# Multi‐omics–driven precision medicine

**DOI:** 10.1002/imt2.70165

**Published:** 2026-08-25

**Authors:** Huibo Li, Zhe Zhao, Yifan Zhang, Yao Ma, Gaofei Hu, Min Zeng, Zhanqun Yang, Zixuan Zhao, Xin Zhou, Wei Hu, Yuxuan Sun, Meng Su, Jun Li, Matthew Whiteman, Wei Fu, Chao Zhong, Lemin Zheng, Long Chen, Hairong Lv, Rongsheng Zhao, Yi Zhun Zhu

**Affiliations:** ^1^ School of Pharmacy, Faculty of Medicine, Laboratory of Drug Discovery from Natural Resources and Industrialization, Macau University of Science and Technology Macau SAR China; ^2^ Department of Pharmacy Peking University Third Hospital; Peking University Therapeutic Drug Monitoring and Clinical Toxicology Center; Institute for Drug Evaluation, Peking University Health Science Center Beijing China; ^3^ State Key Laboratory of Natural and Biomimetic Drugs, Peking University Beijing China; ^4^ Department of General Surgery Cancer Center, Peking University Third Hospital Beijing China; ^5^ Beijing Key Laboratory for Interdisciplinary Research in Gastrointestinal Oncology, Peking University Third Hospital Beijing China; ^6^ The Institute of Cardiovascular Sciences and Institute of Systems Biomedicine, School of Basic Medical Sciences, State Key Laboratory of Vascular Homeostasis and Remodeling, NHC Key Laboratory of Cardiovascular Molecular Biology and Regulatory Peptides, Beijing Key Laboratory of Cardiovascular Receptors Research, Health Science Center, Peking University Beijing China; ^7^ Department of Immunology School of Basic Medical Sciences, NHC Key Laboratory of Medical Immunology, Medicine Innovation Center for Fundamental Researches on Major Immunology‐Related Diseases, Peking University Beijing China; ^8^ Institute of Advanced Clinical Medicine, Peking University Beijing China; ^9^ Ministry of Education Key Laboratory of Bioinformatics, Bioinformatics Division at the Beijing National Research Center for Information Science and Technology, Center for Synthetic and Systems Biology, Department of Automation Tsinghua University Beijing China; ^10^ Department of Pharmacy Children's Hospital, Tianjin University Tianjin China; ^11^ College of Pharmacy, University of Louisiana at Monroe Monroe Louisiana USA; ^12^ Department of Gastroenterology Peking University Third Hospital Beijing China; ^13^ University of Exeter Medical School Exeter UK; ^14^ Department of Rheumatology and Immunology Peking University Third Hospital Beijing China; ^15^ Department of Gastroenterology Peking University First Hospital Beijing China; ^16^ Shanghai Key Laboratory of Bioactive Small Molecules and Department of Pharmacology School of Pharmacy, Fudan University Shanghai China; ^17^ Department of Cardiology, Xiangshan International Medical Center Zhuhai People's Hospital, Macau University of Science and Technology Zhuhai Guangdong China

**Keywords:** artificial intelligence, clinical decision‐making, multi‐omics, precision medicine, translational medicine

## Abstract

Precision medicine is increasingly constrained not by a lack of molecular data but by the absence of frameworks that can translate multidimensional biological information into actionable clinical decisions. Multi‐omics‐driven precision medicine (MODPM) addresses this lack by integrating genomics, epigenomics, transcriptomics, proteomics, metabolomics, microbiome, and clinical context into a multiscale framework that links molecular mechanisms, tissue organization, and patient trajectories. In this review, we propose a conceptual framework for MODPM and examine how advances in multi‐omics technologies, artificial intelligence (AI), and foundation models are reshaping disease modeling, drug development, and precision intervention. We summarize the biological contributions of major omics layers and discuss how AI supports cross‐modal representation learning, contextual modeling, and perturbation‐aware prediction. We highlight drug development as a key translational application of MODPM and further discuss its clinical relevance across three major disease contexts: cancer, autoimmune diseases, and metabolic disorders, including cardiometabolic and renal–metabolic diseases. These examples illustrate how MODPM can support target discovery, disease endotyping, treatment response prediction, and clinical monitoring by analyzing shared mechanisms such as immune dysregulation, metabolic remodeling, chronic inflammation, tissue microenvironmental changes, and gene–environment interactions. Across these settings, MODPM enables finer molecular stratification, the identification of pathway‐dominant disease states, improved response prediction, and dynamic treatment monitoring. We also discuss key barriers to implementation, including data heterogeneity, limited cohort diversity, polygenic complexity, workflow constraints, cost, and ethical issues related to privacy, consent, and data ownership. Overall, the value of MODPM lies not in stacking additional data layers but in building a multiscale, continuously learnable framework to link biological heterogeneity to clinically interpretable and actionable decisions.

## Highlights

- Multi‐omics‐driven precision medicine (MODPM) is best understood as a multiscale decision architecture that links molecular heterogeneity, tissue context, and patient‐level trajectories.
- Artificial intelligence provides an inference layer for representation learning, cross‐modal integration, and perturbation‐oriented modeling across omics and clinical data.
- Multi‐omics is reshaping translational medicine by improving target prioritization, mechanistic validation, patient enrichment, treatment‐response assessment, and mechanism‐based intervention across oncological, autoimmune, cardiometabolic, and renal–metabolic diseases.
- The future of MODPM depends on overcoming data heterogeneity, biological complexity, implementation bottlenecks, and unresolved challenges in equity, privacy, and data governance.

## Graphical abstract

Multi‐omics‐driven precision medicine (MODPM) provides a multiscale, continuously learnable framework that integrates genomics, epigenomics, transcriptomics, proteomics, metabolomics, microbiome profiles, and clinical data. Powered by artificial intelligence and foundation models, MODPM enables cross‐modal representation learning, contextual modeling, molecular network inference, and perturbation‐oriented prediction. This framework transforms multidimensional biological information into actionable clinical decisions by advancing target identification, drug development, biomarker discovery, disease stratification, precision oncology, and individualized management across disease areas such as autoimmune, cardiometabolic, and renal–metabolic diseases. Continuous feedback from clinical implementation further refines models, supports dynamic monitoring, and addresses barriers related to heterogeneity, cohort diversity, workflow, cost, and ethics.

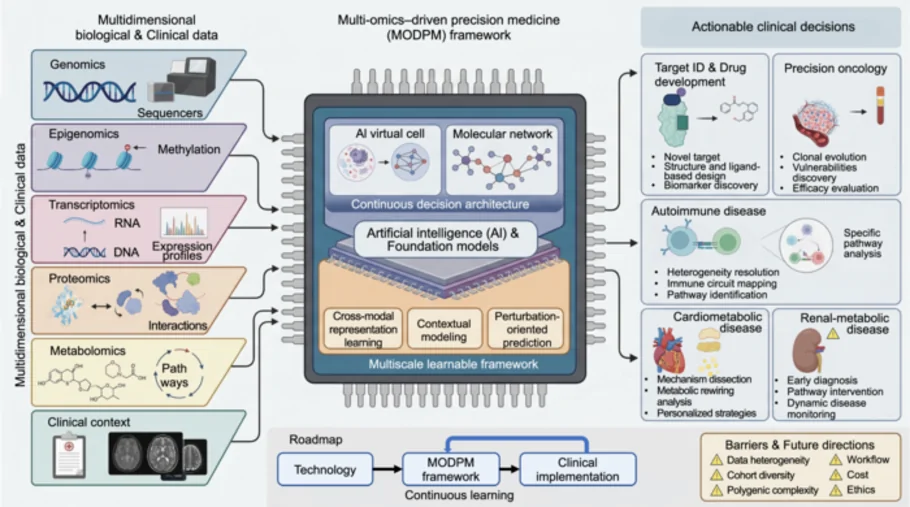

## INTRODUCTION

Precision medicine integrates an individual's genomic, environmental, and lifestyle profiles to guide medical decisions, thereby refining the framework for disease prevention, diagnosis, and treatment [1]. Early observations of disease heterogeneity across different medical traditions laid a conceptual foundation for individualized treatment [2, 3], yet the field gained global traction only after the launch of the *Precision Medicine Initiative* in 2015 [4]. Today, precision medicine is driving a paradigm shift from population‐based approaches towards therapeutic strategies tailored to each patient's unique molecular profile [5, 6, 7]. This transformation is rooted in the recognition that disease arises not from isolated molecular events but from multilayered interactions across the genome, epigenome, transcriptome, proteome, and metabolome and also their dynamic interplay with the microenvironment [8, 9]. Single‐cell and spatial technologies have further revealed that cellular heterogeneity, clonal architecture, and tissue‐level organization collectively shape disease phenotypes and treatment responses [6, 10]. Consequently, multi‐omics integration lies at the core of precision medicine, offering a multidimensional view of how genetic, regulatory, and metabolic factors collectively drive pathophysiology [7, 11, 12]. Unlike single‐omics analyses, which often capture associations rather than causal mechanisms, multi‐omics approaches triangulate evidence across biological hierarchies to uncover emergent properties otherwise obscured within any single data layer [9, 13]. This capability is particularly critical for addressing interpatient variability, intratumoral clonal diversity, and the dynamic evolution of disease under therapeutic pressure. Each of these factors poses a major obstacle to effective precision treatment [14].

However, the central challenge of precision medicine is no longer data scarcity. High‐throughput omics technologies now generate extensive molecular profiles across large populations, as illustrated by the UK Biobank and emerging multi‐omics atlases [15, 16]. However, robust frameworks that can translate this multidimensional information into actionable clinical decisions remain limited [17]. Integrating heterogeneous data types, controlling technical variability such as batch effects, and extracting clinically meaningful signals from high‐dimensional data sets remain major barriers to translation [13, 14]. Moreover, each additional omics layer increases the sparsity and computational burden, intensifying the curse of dimensionality and rendering many conventional analytical approaches inadequate [9, 18]. Combining multi‐omics integration with artificial intelligence (AI) offers a pathway to overcome these barriers by providing the framework necessary to convert molecular complexity into clinical utility. AI‐powered multi‐omics frameworks now enable the continuous refinement of intervention strategies by integrating real‐world clinical outcomes [13, 14]. Recent studies have demonstrated that integrating multi‐omics with AI can identify complementary contributions from different molecular layers to improve the personalized prediction of complex diseases such as cardiovascular disorders. This approach also accelerates target identification and drug discovery by providing causal evidence for disease‐relevant pathways [13, 19]. These advances illustrate that multi‐omics is not merely a supplementary data source but also a foundational infrastructure for reimagining how biomedical knowledge is generated, synthesized, and applied at the point of care.

Within this review, we conceptualize multi‐omics‐driven precision medicine (MODPM) not as a passive compilation of molecular layers but as a hypothesis‐driven framework that traces how genomic and epigenomic variations propagate through pathway networks, reshape the tissue microenvironment, define disease endotypes, and modulate therapeutic responses, then ultimately informs patient‐specific clinical decisions. This perspective emphasizes that the strength of multi‐omics does not simply come from adding more data layers, but from linking molecular changes across biological levels in a way that clarifies disease mechanisms and informs precision interventions. Nevertheless, translating this mechanism‐oriented vision into clinical reality faces nontrivial obstacles when artificial intelligence is introduced. Multi‐omics data sets are typically high‐dimensional, sparse, and affected by batch effects, missing modalities, and interpopulation heterogeneity. These inherent complexities predispose AI models to overfitting and poor external generalizability. Moreover, many state‐of‐the‐art models are opaque in nature, and while they often produce accurate results, they provide little mechanistic insight, thereby hindering clinician trust and biological validation. On the implementation side, precision medicine further demands standardized sample handling, clinically acceptable turnaround times, cost‐effectiveness, regulatory clearance, data privacy safeguards, clinician training, and seamless integration with electronic health records. These intertwined technical and practical challenges must be addressed before AI‐powered multi‐omics can reliably enter routine care. This review presents a multi‐omics‐centric framework for precision medicine, examining how integrated omics approaches illuminate molecular heterogeneity across major disease domains, including oncology, autoimmune disorders, cardiometabolic diseases, and renal–metabolic diseases. We explore the methodological infrastructure that enables these applications, encompassing AI‐powered integration strategies and foundation models, alongside the role of multi‐omics throughout the drug development pipeline. By synthesizing advances across these domains, this review delineates both the transformative potential of mechanism‐oriented multi‐omics integration and the persistent technical and practical challenges that must be addressed to accelerate clinical translation.

## AN INTEGRATED CONCEPTUAL FRAMEWORK FOR MULTI‐OMICS‐DRIVEN PRECISION MEDICINE

### Beyond the definition: The paradigm shift to systems biology

The conceptual evolution of precision medicine has transitioned from a visionary ideal to a rigorous, data‐driven reality. At its core, precision medicine seeks to guide medical decisions by integrating an individual's genomic, environmental, and lifestyle characteristics, thereby establishing a more accurate framework for disease prevention, diagnosis, and treatment [20, 21]. Observations of human biological heterogeneity date back to ancient medical traditions, such as syndrome differentiation in traditional Chinese medicine and medieval Persian insights into individualized drug responses. Following the 2015 announcement of the *Precision Medicine Initiative*, the field underwent marked global expansion [4, 22, 23, 24]. Currently, a fundamental paradigm shift is underway, from population‑based, generalized medical practices toward personalized treatment strategies rooted in individual molecular profiles. This shift reflects the recognition that disease arises not from isolated molecular events but from coordinated perturbations across the genome, epigenome, transcriptome, proteome, metabolome, microbiome, and tissue microenvironment [11, 25]. Compared with single‐omics approaches, which often provide a narrow and correlative view, multi‐omics integration enables the cross‐layer triangulation of biological evidence. It can help distinguish driver events from secondary alterations, reveal system‐level disease mechanisms, and support a more precise interpretation of human health and disease [26, 27].

### The integrated decision architecture: Connecting molecules to patients

The MODPM framework integrates molecular, tissue, and patient layers as a continuous decision‐making system, shifting the traditional paradigm from static diagnostic indicators to dynamic, evolving clinical intervention [28]. This integration operates vertically across molecular layers and organizational levels within the same human biological system, linking genomic variation, epigenetic regulation, transcriptomic activity, proteomic signaling, metabolic phenotypes, the tissue microenvironment, and patient‐level clinical outcomes. At the molecular level, data on genetic variants, epigenetic modifications, and metabolite profiles enable the identification of drivers, the discovery of drug targets, and the resolution of molecular heterogeneity [9, 29]. These molecular insights are then contextualized within the tissue layer, where spatial transcriptomics and histopathology reveal the cellular ecosystem, intercellular interactions, and influence of the microenvironment on drug efficacy [30, 31]. Finally, this information is synthesized at the patient layer, incorporating electronic health records (EHR), radiomics, and longitudinal treatment responses to facilitate patient stratification, treatment selection, and long‐term prognosis [32, 33, 34]. This cross‐layer connectivity ensures that molecular changes are understood within their structural context, ultimately leading to precise clinical interventions. Central to this framework is a dynamic learning capability to transform clinical experience into algorithmic insight through a closed‑loop feedback mechanism powered by real‑world data and continuous model updating. By integrating clinical outcomes, such as drug sensitivity or resistance emergence, the system continuously refines intervention strategies, enabling personalization of therapeutic regimens on the basis of evolving disease biology [35, 36]. This shift from static observation to dynamic feedback represents a critical step toward realizing *“the right treatment to the right person at the right time*” [4, 37, 38].

In clinical workflows, MODPM can be implemented as a stepwise decision‐support process. First, patients were selected on the basis of clinical indication, disease stage, family history, treatment history, or unexplained heterogeneity. Second, appropriate biospecimens are collected and profiled using selected omics technologies. Third, molecular data are integrated with pathology, imaging, laboratory tests, medication history, and clinical outcomes. Fourth, computational models generate clinically interpretable outputs, such as risk groups, molecular subtypes, actionable targets, or predicted treatment responses. Finally, the results are reviewed by a multidisciplinary team and, when appropriate, translated into treatment selection, monitoring strategies, or clinical trial enrollment.

### The intelligence layer: AI as the engine of predictive modeling

Multi‐omics modeling can serve several distinct but complementary purposes in MODPM. Descriptive modeling aims to characterize biological patterns, identify molecular subtypes, and reveal patient or cell‐state heterogeneity. Predictive modeling uses multi‐omics and clinical features to estimate future or unobserved outcomes, such as disease risk, molecular subtype, prognosis, treatment response, drug toxicity, or relapse. Mechanistic modeling seeks to explain causal, regulatory, or pathway‐level relationships among molecular layers. Perturbation modeling simulates how genetic, pharmacological, immune, or environmental interventions alter molecular states and phenotypes. Generative and foundation‐model‐based approaches aim to learn transferable representations, impute missing modalities, and support cross‐context prediction. Clarifying these modeling goals is essential because each requires different data structures, validation strategies, interpretability standards, and clinical evidence.

AI functions as the intelligence layer of MODPM by converting high‐dimensional molecular data into transferable biological representations. The field is shifting from task‐specific models based on manually engineered features toward reusable representations learned through foundation models [39]. This progression extends from latent‐variable and graph‐based integration methods to large pretrained models such as DNABERT, scGPT, and AlphaFold, which learn molecular or cellular regularities from large‐scale data sets [40, 41]. At the frontier, the AI Virtual Cell (AIVC) should be understood less as a single clinically deployed model than as an emerging framework for simulating biological states across molecular, subcellular, and tissue scales [42, 43, 44, 45]. In principle, such systems could enable the in silico testing of genetic or pharmacological perturbations before experimental or clinical validation [46]. Thus, AI is not merely an auxiliary analytic tool; it is becoming the computational substrate that links molecular profiling to prediction, simulation, and, eventually, more adaptive clinical decision‐making [47].

### A roadmap of the chapter: From technology to clinical implementation

**Figure 1:**
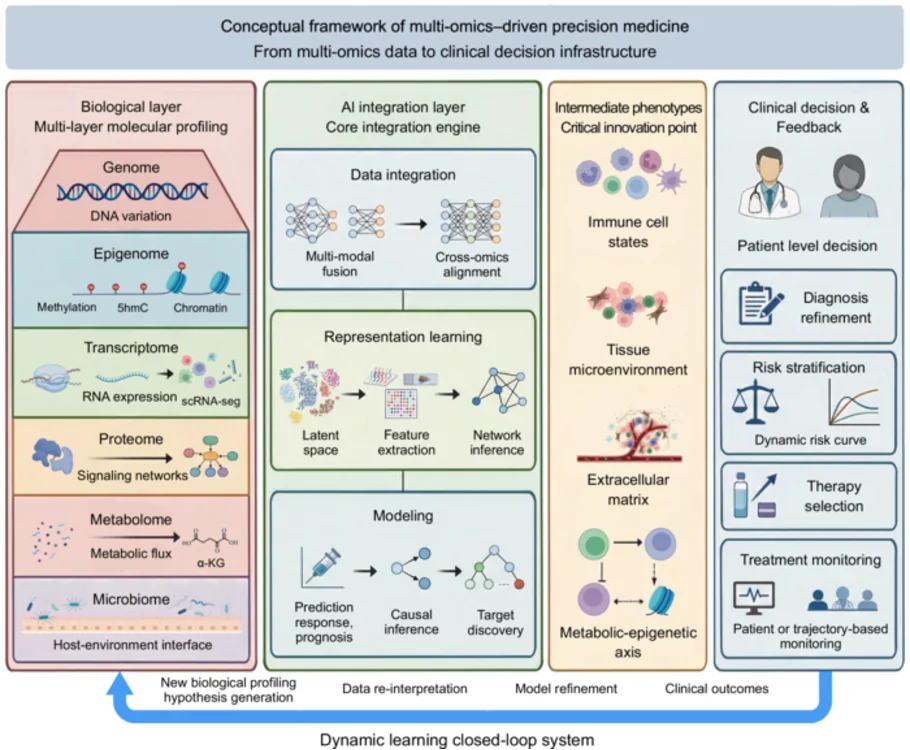
Conceptual framework of multi‐omics‐driven precision medicine. Multi‐omics‐driven precision medicine is depicted as a multiscale and continuously learnable decision architecture that links molecular heterogeneity to clinically actionable decisions. The framework comprises four interconnected layers: a biological data layer integrating genomic, epigenomic, transcriptomic, proteomic, metabolomic, and clinical information; an AI integration layer supporting multimodal fusion, representation learning, network inference, contextual modeling, and predictive analytics; an intermediate phenotype layer that converts high‐dimensional molecular signals into interpretable biological states, such as immune polarization, tissue microenvironmental organization, metabolic remodeling, and metabolic–epigenetic coupling; and a clinical decision and feedback layer that enables diagnosis refinement, risk stratification, therapy selection, response prediction, and longitudinal monitoring. Iterative feedback from real‐world outcomes supports continuous refinement of the framework and facilitates more adaptive, mechanistic, and patient‐specific precision medicine.

The remainder of this review summarizes the technologies, analytical models, and clinical applications that support multi‐omics‐driven precision medicine. We first review the evolving landscape of multi‐omics technologies, highlighting the transition from bulk profiling to single‐cell and spatially resolved analyses. This section covers genomic, epigenomic, and transcriptomic approaches, including emerging insights from tRNA‐related research, as well as proteomic and metabolomic strategies that capture downstream functional changes [48]. This technological foundation enables the capture of biological information across multiple scales, from molecular events to tissue architecture. The next section explores AI‐driven modeling, examining how foundation models and virtual cells are reshaping our ability to predict biological perturbations. The focus then shifts to the revolution in drug development: multi‐omics is countering “Eroom's Law” by providing reliable evidence across the drug discovery pipeline [49]. Furthermore, this review examines the roles of multi‐omics in precision treatment for specific diseases, such as the resolution of heterogeneity and drug resistance in oncology, the system‑level deconstruction of immune programs and stromal niches in autoimmune diseases, and the implementation of digital twins in cardiometabolic disease and renal–metabolic disorders [50]. Finally, the review addresses challenges and future trends, including technical bottlenecks, barriers to clinical implementation, and ethical and privacy concerns surrounding identifiable omics data. Together, these sections illustrate that multi‐omics is not merely an additional data source but represents a fundamental transition toward a more holistic, human‐centric model of medicine (Figure 1).

## A LANDSCAPE OF MULTI‐OMICS‐DRIVEN PRECISION MEDICINE

**Figure 2:**
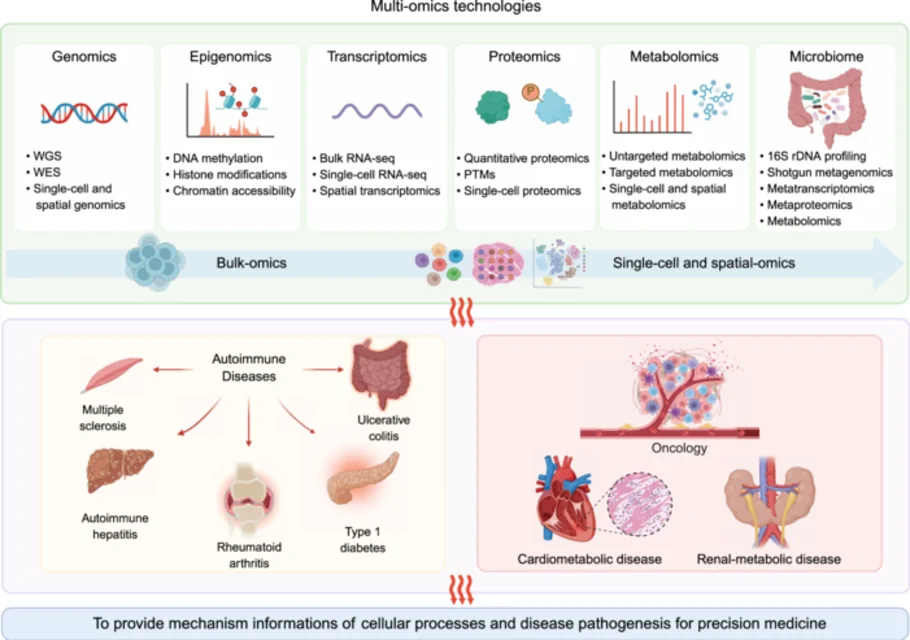
Major multi‐omics technologies in precision medicine. Multi‐omics technologies provide complementary molecular information across genomics, epigenomics, transcriptomics, proteomics, metabolomics, and the microbiome. These approaches cover bulk‐level profiling as well as single‐cell and spatially resolved analyses, enabling the systematic characterization of genetic variation, regulatory states, gene expression, protein abundance and modifications, metabolic pathways, and host–microbiome interactions. By integrating these molecular layers, multi‐omics profiling helps elucidate cellular processes and disease pathogenesis, supporting patient stratification, biomarker discovery, therapeutic target identification, and individualized clinical decision‐making. PTMs, posttranslational modifications; WES, whole‐exome sequencing; WGS, whole‐genome sequencing.

Multi‐omics technologies have become a cornerstone of biological research and precision medicine, enabling the systematic interpretation of biological information from DNA, RNA, proteins, metabolites, and epigenetic modifications [24]. In recent decades, advances in high‐throughput instruments, novel chemistries, data‐processing methods, and reference resources have transformed multi‐omics from basic science to translational tools for patient stratification, biomarker and drug target discovery, and treatment monitoring [51] (Table 1). This section summarizes the recent landscape of multi‐omics technologies, focusing mainly on genomics, epigenomics, transcriptomics, proteomics, metabolomics, and the microbiome (Figure 2).

**Table 1:** Key resources for multi‐omics data and analytical tools.

Category	Resource	Omics	Key Features	Access
Genomics Multi‐omics	TCGA	Multi‐omics	Comprehensive cancer multi‐omics data for >30 tumor types; includes DNA mutations, CNV, mRNA/miRNA expression, DNA methylation, protein expression, and clinical data	https://portal.gdc.cancer.gov/
Transcriptomics Genomics	GTEx	Transcriptomics, Genomics	Tissue‐specific gene expression and regulation data across 54 nondiseased tissues from ~1000 individuals; includes WGS, RNA‐Seq, and eQTL data for studying genetic effects on expression	https://gtexportal.org/home/
Multi‐omics	UCSC Xena	Multi‐omics Integration Platform	Web‐based visual integration and exploration tool; hosts TCGA, GTEx, ICGC, CCLE, PCAWG data sets (>1500 data sets across 50+ cancer types); supports survival analysis, genomic signatures, and multi‐omics visualization	https://xena.ucsc.edu/
Multi‐omics	cBioPortal	Multi‐omics Integration Platform	Integrates and visualizes TCGA and other published cancer studies; supports multi‐omics queries, survival analysis, and pathway visualization	https://www.cbioportal.org/
Genomics	GSA‐Human	Genomics	Controlled‐access archive for human raw sequencing data (WGS/WES), enhanced quality control and privacy protection (2025 version)	https://ngdc.cncb.ac.cn/gsa-human/
Genomics	gnomAD	Genomics	Large‐scale population standard variant database for filtering benign variants and annotating rare pathogenic variants	https://gnomad.broadinstitute.org/
Genomics	PharmGKB	Pharmacogenomics	Knowledge base for pharmacogenomic variants, CYPs, and drug response phenotypes	https://www.pharmgkb.org/
Epigenomics	MethBank	DNA Methylation	High‐quality DNA methylome database integrating WGBS, EM‐seq data; single‐base resolution methylation annotation	https://ngdc.cncb.ac.cn/methbank/
Epigenomics	ENCODE	Epigenomics	Comprehensive functional genomics encyclopedia; includes ChIP‐seq, ATAC‐seq, Hi‐C data for epigenetic regulation	https://www.encodeproject.org/
Epigenomics	CUT&Tag Resource	Epigenomics	Technical resource for low‐input epigenomic profiling; provides CUT&Tag/CUT&RUN protocols and analysis guides	https://www.epicypher.com/resources/
Transcriptomics	CELLxGENE	Single‐cell RNA‐seq	Standardized single‐cell transcriptome platform; includes hundreds of millions of cells with cross‐dataset comparison	https://cellxgene.cziscience.com/
Transcriptomics	SpatialRef	Spatial Transcriptomics	Manually curated spatial transcriptome database; integrates ST, Slide‐seq data; supports cell–cell communication analysis	http://www.liclab.net/spatialref/
Transcriptomics	SG‐RNASeq	tRNA‐seq	Specialized database for tRNA and tsRNA sequencing; includes modification sites, expression levels, and isoform information	https://sg-rnaseq.com/
Proteomics	PRIDE	Proteomics	Largest global proteomics data repository; stores MS‐based DDA/DIA data for reanalysis	https://www.ebi.ac.uk/pride/
Proteomics	Olink	Targeted Proteomics	High‐throughput targeted proteomics platform based on PEA technology; quantifies >3000 proteins at fM sensitivity (commercial)	https://www.olink.com/
Proteomics	CPTAC	Proteomics	Proteomic (protein/phosphoprotein) data linked to TCGA tumor samples; enables integrated cancer proteogenomic analysis	https://proteomics.cancer.gov/programs/cptac
Proteomics	BioGRID	Protein–Protein Interaction	Manually curated database of protein–protein and genetic interactions; includes BioID, APEX proximity labeling data	https://thebiogrid.org/
Metabolomics	HMDB	Metabolomics	Most comprehensive human metabolome database; includes >200,000 metabolite structures, pathways, and disease associations	https://hmdb.ca/
Metabolomics	GNPS	Metabolomics	Molecular networking‐based mass spectrometry data‐sharing platform; supports unknown metabolite annotation	https://gnps.ucsd.edu
Microbiome	GutMetaNet	Metagenomics	Database integrating horizontal gene transfer and functional redundancy in human gut microbiome; supports functional module analysis	https://gutmetanet.deepomics.org/
Microbiome	iHMP	Multi‐omics Microbiome	Integrative Human Microbiome Project database; includes host‐microbe multi‐omics data for IBD, pregnancy, and other conditions	https://hmpdacc.org/ihmp/
Single‐cell Multi‐omics	scCancerExplorer	Single‐cell Multi‐omics	Single‐cell multi‐omics database for human pan‐cancer; integrates scRNA‐seq, scATAC‐seq, scDNA methylation, and CNV data across 50 cancer types; includes TCGA survival analysis module	https://bianlab.cn/scCancerExplorer
Single‐cell Multi‐omics	Tabula Sapiens	Single‐cell Multi‐omics	Human single‐cell multi‐omics atlas; includes transcriptome, proteome, and immune repertoire data with cell–cell interaction tools	https://tabula-sapiens.sf.czbiohub.org/
Spatial Omics	HuBMAP	Spatial Multi‐omics	Human BioMolecular Atlas Program; integrates spatial transcriptomics and proteomics to build 3D tissue molecular maps	https://portal.hubmapconsortium.org/
Multi‐omics Integration Tools	UCSCXenaShiny	Integrative Tool	R package and web application for interactive analysis of UCSC Xena data sets; supports correlation, comparison, and survival analysis in individual, pan‐cancer, and batch screen modes; includes pharmacogenomics functionalities	https://github.com/openbiox/UCSCXenaShiny
Multi‐omics Integration Tools	Flexynesis	Integrative Tool	Deep learning–based multi‐omics integration toolkit (2025); supports transcriptome‐genome‐proteome joint modeling for survival and drug response prediction	https://github.com/BIMSBbioinfo/flexynesis
Multi‐omics Integration Tools	BIG Search	Integrative Platform	Cross‐database search engine (2025); enables unified search and data retrieval across CNCB, NCBI, and EBI	https://ngdc.cncb.ac.cn/

Abbreviations: CYP, cytochrome P450 enzyme; GTEx, Genotype‐Tissue Expression; IBD, inflammatory bowel disease; TCGA, The Cancer Genome Atlas.

### Genomics

Genomics, one of the earliest and most mature high‐throughput technologies for precision medicine, has evolved from single‑candidate‑gene sequencing to genome‑wide profiling [11]. The Human Genome Project and subsequent technological advances drove the transition from microarray‑based genotyping and comparative genomic hybridization to next‑generation sequencing (NGS), which enables the genome‑wide detection of single‑nucleotide variants (SNVs), small insertions/deletions, copy‑number alterations, and structural variants [52, 53]. Whole‑genome sequencing (WGS) provides the most comprehensive view of genomic characteristics, including coding and non‑coding variations across the entire human genome [54], whereas whole‑exome sequencing (WES) is more cost‑efficient owing to its exclusive capture of protein‑coding regions [55]. Targeted gene panels are widely adopted clinically because of their optimal balance of sequencing depth, turnaround time, and interpretability [56]. Advances in variant calling pipelines, standardized reference genomes, and large population databases have greatly improved the interpretation accuracy of pathogenic variations, although challenges remain in interpreting rare variants and complex structural rearrangements [57, 58]. Improvements in library preparation and error suppression have expanded the detection of low‐frequency variants [59, 60]. Advanced long‑read sequencing (represented by Pacific Biosciences HiFi and Oxford Nanopore Technologies) enables the effective analysis of complex structural variants often missed by short‑read methods [61, 62].

Genomic technologies also vary by sample type and analytical aim. For example, tumor‐normal sequencing enables the identification of somatic driver mutations and mutational signatures [63, 64], whereas germline genomics provides information on inherited risk, pharmacogenomics, and familial disease [65]. Notably, the use of pharmacogenomics (which focuses on genes encoding drug‑metabolizing enzymes, transporters, and pharmacodynamic targets, most notably cytochrome P450 enzymes) has translated numerous genomic variations into reliable predictors of drug efficacy and adverse events [66, 67]. Liquid biopsy, typically represented by cell‑free DNA (cfDNA) analysis, has shifted genetic monitoring from invasive tissue samples toward blood or other body fluids, enabling the noninvasive or minimally invasive detection of minimal residual disease (MRD), clonal evolution, and emerging resistance mutations [68, 69, 70]. Single‑cell and spatial genomics are emerging technologies capable of interpreting cellular genomic heterogeneity within tissues and reconstructing clonal architectures and evolutionary paths in cancer and other diseases, although they still face technical challenges such as amplification biases and uneven coverage [71].

### Epigenomics

Epigenomics refers to the study of heritable or nonheritable modifications to DNA or DNA‑associated proteins that modulate gene expression without altering the DNA sequence [72]. Epigenetic alterations play critical roles in diseases [73, 74]. The best‑known epigenetic marker is DNA methylation, which predominantly occurs as cytosine modification (e.g., 5‑methylcytosine, 5mC) within CpG islands [75]. DNA methylation controls cellular processes via the modulation of gene expression and is strongly associated with disease [73, 76]. Multiple strategies have been developed to profile DNA methylation signatures. Whole‑genome bisulfite sequencing (WGBS) is the gold standard method for detecting DNA methylation sites at single‐base resolution [77]. However, this method has several drawbacks, including severe DNA damage and low conversion efficiency. Bisulfite‑free enzyme‑based methods, such as enzymatic methyl sequencing (EM‑seq) and TET‑assisted pyridine borane sequencing (TAPS) [78, 79], and optimized bisulfite‐based methods, such as ultrafast BS‑seq (UBS‑seq) and ultramild bisulfite sequencing (UMBS‑seq) [80, 81], have been developed to address these limitations. In addition to 5‑methylcytosine, a series of stable cytosine modifications, including 5‑hydroxymethylcytosine (5hmC), 5‑formylcytosine (5fC), and 5‑carboxylcytosine (5caC), occur during the demethylation process [82]. These modifications are correlated with gene expression and modulate cellular processes, and methods have been developed to decipher these modifications genome‑wide [82, 83, 84, 85]. Single‑cell and multimodal mapping of these DNA modifications represent a cutting‑edge direction in this field [86, 87]. In recent years, invasive and noninvasive profiling of DNA epigenetic marks has shown great potential for early diagnosis of diseases and monitoring of drug responses [88, 89, 90, 91].

Posttranslational histone modifications are another major form of epigenetic modification with critical roles in controlling transcriptional activity through the recruitment of transcription factors and repressive proteins, activation of transcriptional enhancers, and interactions with the DNA methylation machinery [72, 92]. Histone modifications include methylation, acetylation, and a series of newly discovered modifications [92, 93, 94, 95]. The third major form of epigenetic information is chromatin accessibility and higher‐order chromatin structure [92]. Chromatin immunoprecipitation sequencing (ChIP‐seq) is typically used to profile histone modifications and chromatin‐binding proteins to elucidate active and repressive regulatory marks [96]. Technical advances include the CUT&RUN and CUT&Tag methods, which improve sensitivity and reduce input requirements [97, 98]. ATAC‑seq (assay for transposase‑accessible chromatin using sequencing) is applied to map chromatin accessibility [99]. Like other omics, the single‑cell resolution profiling of histone modifications, chromatin accessibility, and higher‑order chromatin structure represents a frontier in the field [100, 101].

### Transcriptomics

Transcriptomics was initially established through expressed sequence tag analysis and microarray‐based profiling and was subsequently revolutionized by RNA sequencing (RNA‐seq) [102, 103]. This technology provides an unbiased, quantitative characterization of the transcriptome, encompassing gene expression levels, alternative splicing events, allele‑specific expression, and non‑coding RNA species [102, 103]. Bulk RNA‑seq has become a routine method for identifying differentially expressed genes and pathway changes, as well as for discovering aberrant transcript isoforms and gene fusions across various diseases [102, 103]. The development of strand‐specific library preparation, ribosomal RNA depletion, and improved alignment and quantification algorithms has enhanced detection sensitivity, with particularly pronounced benefits for low‐abundance transcripts [104, 105, 106]. Notably, transcriptomics enables the dynamic capture of cellular responses to stimulation and treatment, providing information on disease progression and pharmacodynamics [107, 108, 109].

The most recent advances in transcriptomics are single‑cell RNA‑seq (scRNA‑seq), spatial transcriptomics, and long‑read RNA‑seq (LRS). scRNA‑seq has revolutionized our understanding of tissue heterogeneity, enabling the identification of rare cell populations and comprehensive profiling of the immune microenvironment in cancer, autoimmune disorders, and virtually any disease context [110, 111, 112, 113]. Spatial transcriptomics preserves histological architecture and spatial resolution while measuring gene expression, thus offering insights into intercellular communication and immune infiltration [114, 115, 116, 117, 118]. These approaches are primarily classified into two categories. The first category comprises NGS‐based approaches, where spatial information of the transcripts is defined prior to NGS; the second comprises imaging‑based approaches, which rely on in situ sequencing or in situ hybridization combined with imaging‑based methods [119, 120, 121]. LRS is an emerging and powerful method for transcriptomic studies [61, 122]. Owing to the short read‑length window of short‑read RNA‑seq (typically 50–300 bp, substantially shorter than the average length of mRNA transcripts), many noncanonical transcript isoforms remain undetectable [122, 123]. Conversely, LRS can generate reads longer than 10 kb (up to 4 Mb), enabling the direct sequencing of full‑length mRNA transcripts and providing more comprehensive information [61, 122].

Beyond conventional mRNA, the tRNA transcriptome represents a frontier in transcriptomics research. tRNAs are a highly modified and dynamically regulated class of RNAs that link genome variation, transcriptome regulation, and proteome output. During disease progression, alterations in the tRNA pool may reshape proteome output and cellular phenotypes [124]. Among all RNA species, tRNAs have the highest density and diversity of noncanonical nucleotides because of extensive posttranscriptional modifications. The chemical identity and positional distribution of modified nucleotides, as well as the abundance and sequence composition of individual tRNA isodecoders, vary across cell types, tissues, and individuals in a spatiotemporally regulated manner. Although this complexity presents substantial technical challenges for single‐cell tRNA sequencing, it also creates opportunities to leverage tRNA‐seq information for early diagnosis and precision therapeutic stratification.

tRNA transcriptome profiling relies on high‐throughput sequencing and faces distinct technical challenges [125]: inefficient adapter ligation, reverse transcriptase stalling due to nucleotide modifications, and interference from highly stable secondary structures. The accurate identification and alignment of modified nucleotides are necessary to ensure cross‐study comparability, but they introduce computational complexity and protocol variability. Recent advances aim to address these issues, including thermostable reverse transcriptases [126], cutting‐edge sequencing workflows, such as ARM‐seq and Nano‐tRNAseq [127, 128], specialized analytical pipelines [129, 130, 131, 132], and continuously updated modification databases [133].

In contrast to bulk approaches, single‐cell and spatial tRNA sequencing remain technically immature. The lack of poly(A) tails and defined capture motifs complicates adapter ligation, resulting in heterogeneous libraries and increased mapping ambiguity. Simplified library preparation strategies are therefore needed. Single‐molecule platforms such as SMRT may help capture full‐length tRNAs and modification signatures [134, 135]. However, most current methods remain limited to bulk‐level analysis [136].

tRNA transcriptomes are being increasingly leveraged for biomarker discovery, disease diagnosis, and personalized therapeutic strategies. Sequencing analyses of breast cancer cells have revealed the significant upregulation of specific tRNAs associated with proliferation and metastasis [137, 138], providing a mechanistic rationale for developing anticancer agents targeting these key tRNA species. In Parkinson's disease, altered modification patterns of specific tRNAs have been linked to neuronal dysfunction and cell death [139, 140], highlighting their potential utility in early diagnosis and therapeutic intervention. Abnormal tRNA‐derived fragment profiles have also been detected in patients with diabetes [141, 142]. Collectively, these findings deepen our understanding of disease pathogenesis and support the development of more precise, individualized treatment strategies.

### Proteomics

Proteomics directly reflects the functional phenotype, as proteins execute most biological processes and undergo extensive post‑translational modifications (PTMs) that cannot be readily inferred from genomics or transcriptomics alone [143]. Early proteomics depended on 2D gel electrophoresis and Edman sequencing [144]. However, the field was revolutionized by the development of soft ionization (MALDI, ESI) and tandem mass spectrometry (MS/MS). Shotgun proteomics, combining LC–MS/MS, peptide fragmentation, and database searching, enables the large‐scale identification and quantification of thousands of proteins from complex biological systems. Quantification strategies have evolved from label‑based methods, including stable isotope labeling by amino acids in cell culture (SILAC), isobaric tags for relative and absolute quantitation (iTRAQ), and tandem mass tags (TMT), to label‑free quantification approaches, with different methods offering distinct advantages in throughput, accuracy, and experimental design [145, 146, 147, 148]. Advances in chromatographic separation, mass analyzers, acquisition strategies, and data‐processing workflows have significantly increased the analysis depth and reproducibility of proteomics [149, 150]. Data‑independent acquisition (DIA), which captures all ions within selected m/z ranges regardless of signal intensity, provides more comprehensive proteome profiles, with better quantification accuracy, repeatability, and coverage of low‑abundance proteins, than data‑dependent acquisition (DDA) can [151]. In parallel with mass spectrometry‐based discovery platforms, affinity‐based targeted proteomics has gained substantial traction for biomarker validation and clinical implementation. The proximity extension assay (PEA), commercialized under the Olink platform, utilizes matched antibody pairs directed against distinct epitopes on a target protein; each antibody is conjugated to a unique single‐stranded DNA oligonucleotide [152]. The simultaneous binding of both antibodies positions the DNA tags within nanometer proximity, enabling hybridization and polymerase‐mediated extension to generate a protein‐specific DNA amplicon that is subsequently quantified by quantitative PCR or next‐generation sequencing [153]. This dual‐recognition architecture confers markedly enhanced specificity relative to conventional sandwich immunoassays, while PCR amplification provides sensitivity down to femtomolar concentrations [154]. Current PEA iterations support the multiplexed quantification of more than 3000 proteins from as little as 1 µL of plasma or serum, with a dynamic range spanning 10 orders of magnitude [155]. Single‑cell proteomics is another frontier area that seeks to capture single‑cell functional heterogeneity at the proteome level [156, 157]. Development approaches include mass spectrometry‐based single‐cell proteomics (scMS) and next‐generation sequencing (NGS)‐based single‐cell proteomics [156]. To date, scMS approaches have been the most successful single‑cell proteomics strategies, typically identifying and quantifying up to 1500 proteins in a single cell [156]. Using scMS strategies, researchers have successfully identified distinct cancer cell clusters that are resistant to drug treatment [158]. As an emerging technology, single‑cell proteomics still faces critical challenges, including extreme sample scarcity, inadequate protein coverage, limited throughput, and quantitative variability [156, 157, 159].

Recent proteomics also emphasizes PTMs, protein–protein interactions, and subcellular locations, which are key drivers of protein functions. Typical PTMs include histone modifications and kinase phosphorylation, which are modulators of gene expression and kinase activity [93, 94, 160]. Extensive proteomic strategies have been developed to interpret diverse protein PTMs [93, 94, 160]. BioID, TurboID, APEX2, and genetically encoded crosslinker‐based strategies have been widely used to identify protein interactomes [161, 162, 163, 164]. Enzyme‑based and photocatalyst‑based proximity labeling methods have been extensively developed to identify organelle‑specific proteomes [161, 165, 166].

### Metabolomics

Metabolomics captures small‑molecule repertoires representing either the terminal outputs or the transient intermediates of metabolic pathways, integrating endogenous physiological processes with exogenous inputs from the diet, gut microbiota, pharmaceuticals, and toxins [167, 168]. The analytical landscape of metabolomics has transformed from classical targeted assays of individual compounds to contemporary untargeted and targeted strategies employing mass spectrometry (MS) and nuclear magnetic resonance (NMR) spectroscopy [169, 170]. Untargeted metabolomics aims at broad coverage and the discovery of unknown metabolites, whereas targeted metabolomics provides precise quantification for defined metabolite classes and pathways [169, 170]. Chromatographic separation modalities are selected on the basis of analyte properties: gas chromatography–MS excels for volatile and derivatized metabolites, whereas liquid chromatography–MS is broadly used for lipids, polar metabolites, and diverse compound classes [171]. NMR spectroscopy complements these platforms with superior quantitative accuracy and interlaboratory reproducibility, although its applicability is constrained to more abundant metabolites, whereas MS‐based methods can analyze metabolites with lower abundance [172].

Technological advances have focused on expanding metabolite coverage, improving identification confidence, enhancing quantitative robustness, and increasing throughput. High‐resolution MS‐refined chromatographic methods (hydrophilic interaction chromatography for polar metabolites and reversed‐phase methods for lipids), ion mobility separation, and expanded spectral databases collectively improve annotation accuracy [173, 174]. Nevertheless, metabolite identification remains a critical bottleneck, and many detected features lack definitive structural assignment [173, 174]. Like other omics technologies, single‐cell and spatial metabolomics are emerging fields, with new and fundamental methods emerging rapidly to facilitate metabolic decoding of complex diseases at single‐cell resolution [175, 176].

Metabolomics has strong clinical and disease relevance, as metabolic reprogramming is both a cause and a consequence of pathophysiology [177, 178]. For example, in cancer, many metabolites have been identified as critical drivers of immune evasion [179, 180, 181]. Metabolites can also serve as diagnostic biomarkers and mechanistic evidence for many diseases, including diabetes, kidney disease, cardiovascular disease, and cancer [182, 183, 184, 185]. Another key application of metabolomics is therapeutic drug monitoring, which can enable the dynamic quantification of drug metabolites and endogenous pathway alterations, facilitate the detection of off‑target toxicity, and provide early indicators of therapeutic efficacy [185].

### Microbiome

The human microbiome encompasses the collective genomes of microorganisms colonizing diverse body niches, representing an additional dimension of biological information beyond host‐encoded omics layers [186]. The gut microbiome, harboring approximately trillions of microorganisms, operates as a metabolically active organ that modulates nutrient assimilation, immune education, and xenobiotic transformation through extensive bidirectional communication with host systems [187]. Technological innovations in sequencing and mass spectrometry have catalyzed the transition from cultivation‐dependent microbiology to culture‐independent multi‐omics characterization, enabling the comprehensive profiling of microbial taxonomy, functional genes, transcripts, proteins, and metabolic outputs [188, 189].

Shotgun metagenomic sequencing has superseded 16S rRNA amplicon‐based approaches as the principal methodology for microbial community analysis, resulting in enhanced taxonomic resolution at the species and strain levels while simultaneously capturing functional genomic potential [190, 191]. Large‐scale initiatives, notably the Human Microbiome Project and theIntegrative Human Microbiome Project (iHMP), have generated expansive reference data sets that underpin the identification of microbiome perturbations associated with inflammatory bowel disease, metabolic syndrome, and oncological malignancies [192, 193]. Third‐generation sequencing platforms now generate reads exceeding 10 kb with high accuracy, enabling improved metagenome‐assembled genome construction and the discovery of biosynthesis‐related gene clusters in previously uncultivated lineages [194, 195].

While metagenomics delineates the genomic blueprint of microbial communities, it provides limited insight into dynamically regulated processes. The incorporation of metatranscriptomics, metaproteomics, and metabolomics has therefore become indispensable for interrogating real‐time microbial activity and host–microbe metabolic crosstalk [196, 197]. Metatranscriptomics captures actively transcribed genes under defined conditions, whereas metabolomics profiles small‐molecule effectors, including short‐chain fatty acids, secondary bile acids, and tryptophan derivatives, that function as signaling mediators between microbes and host receptors [198, 199]. The systematic integration of microbiome data with host‐derived multi‐omics data sets presents substantial analytical complexities. Multi‐omics frameworks combining metagenomic, genomic, and metabolomic measurements have begun to resolve intricate interaction networks governing disease pathophysiology [200]. Computational methodologies, encompassing graph neural networks and ensemble learning algorithms, are being increasingly deployed to navigate the high‐dimensional, compositional nature of microbiome data [201]. Nevertheless, persistent challenges include batch effects across platforms, limited reference database coverage, and the difficulty of establishing causality from observational associations [202, 203].

Emerging technological frontiers include spatially resolved microbiome profiling and single‐cell metagenomics, which hold promise to dissect cellular heterogeneity and spatial organization within microbial communities [204, 205]. As a complement to sequencing‐based approaches, advanced culturomics strategies incorporating microfluidic droplet encapsulation and machine learning‐guided medium optimization have expanded the repertoire of isolatable microorganisms, leading to the recovery of taxa previously considered unculturable [206, 207, 208, 209]. The continued maturation of integrative computational pipelines will be pivotal for translating microbiome discoveries into clinically implementable precision medicine applications.

## ARTIFICIAL INTELLIGENCE‐DRIVEN MODELING OF MULTI‐OMICS DATA

Multi‐omics technologies have expanded biological measurements from single molecular layers toward integrated profiles that include genotype, chromatin state, transcriptional activity, protein abundance, metabolic output, tissue architecture, and increasingly, patient‐level clinical information [11, 210]. At the same time, AI has provided new approaches for analyzing these complex data sets. Early studies mainly focused on combining heterogeneous feature matrices from different omics platforms. More recent work has moved toward learning shared representations from molecular and clinical data, allowing information from different assays and cohorts to be compared and applied to multiple downstream tasks [210, 211]. Thus, the contribution of AI to multi‐omics lies not only in improving predictive performance but also in organizing fragmented molecular measurements into interpretable representations of biological states.

This shift matters because precision medicine is rarely driven by a single modality. Molecular assays capture regulatory potential, cellular state, and pathway activity, but diagnosis and treatment rely on tissue architecture, imaging phenotype, clinical course, and therapeutic response. A useful multi‐omics model must therefore extend beyond simple measurement fusion. It must preserve the meaning of each layer while allowing the layers to inform one another. This section first introduces data resolution, ranging from molecular to multiscale inputs, and traces the evolution from early integration models to foundation models and subsequently to the recent AIVC perspective. It then deconstructs these multi‐omics AI systems into a layered architectural framework. Finally, the discussion focuses on molecular foundation models, cross‐modal integration, and multimodal extensions beyond molecular omics, along with perturbation‐oriented modeling and its implications for precision medicine, and concludes with the critical role of interpretable and explainable AI (XAI) in overcoming black‐box limitations.

### Data resolution: From molecular to multiscale inputs

The resolution of model inputs dictates the depth of actionable pathological information that can be extracted to elucidate the true mechanisms underlying diseases. As the paradigm of oncology and complex disease management shifts from one‐size‐fits‐all approach to individualized care, the granularity of data establishes the limits of precision in therapeutic interventions. On the basis of current methodological frameworks, data resolution can be stratified into five scales, each driving specific advancements in precision medicine:

#### Molecular and atomic resolution

Models operating at this tier utilize nucleotides, amino acids, or atomic coordinates as their primary inputs. Approaches such as DNABERT‐2, Evo2, RNABERT, the ESM series, and AlphaFold process vast corpora of DNA, RNA, or protein sequences to capture evolutionary conservation and biophysical constraints [41, 212, 213, 214, 215]. This foundational resolution is critical for target identification and drug discovery. By accurately predicting the functional consequences of genetic variants (variant effect prediction) and determining 3D protein structures, these models enable the rational design of targeted therapeutics and the identification of patient‐specific pathogenic mutations that drive disease susceptibility.

#### Bulk and population‐level resolution

Bulk resolution captures the average molecular state of a tissue. Historically, this physical averaging of cell populations has been the primary bottleneck in clinical omics integration. Early multi‐omics integrations heavily relied on macroscopic bulk profiling, which mathematically averages the mixed signals within a tissue, completely obliterating cellular heterogeneity [210]. Consequently, such macroscopic resolution fails to delineate precise pathogenic states [216, 217]. However, owing to its cost‐effectiveness and the widespread availability of archived data sets, bulk data remain a pragmatic and prevalent resource for computational modeling and biomarker discovery [218]. Models trained on bulk resolution are heavily utilized for clinical risk stratification and the derivation of population‐level prognostic signatures, bridging transcriptomic profiles with long‐term patient survival outcomes.

#### Single‐cell resolution

To overcome the limitations of physical averaging, the advent of single‐cell analysis techniques such as scRNA‐seq forced a paradigm shift [210]. Models such as scGPT can now conceptualize gene expression profiles as high‐dimensional cell embeddings, empowering deep generative models to capture fine‐grained cellular heterogeneity across millions of individual cells [219]. This improvement in resolution from bulk to single‐cell is the indispensable foundation for discovering rare, drug‐resistant pathogenic subpopulations that bulk sequencing would otherwise mask [217]. Furthermore, these models support in silico perturbation simulations, predicting how an individual patient's specific cell types will respond to targeted chemotherapies or genetic interventions, thereby guiding personalized drug selection [43, 220].

#### Spatial and microenvironmental resolution

Fundamentally, spatially resolved transcriptomics (SRT) data constitute a multimodal composite that integrates high‐resolution histology images, two‐dimensional (x, y) physical coordinates, and a high‐dimensional gene expression matrix for each localized spot or cell [208]. By preserving the physical coordinates of molecular measurements, some models use a topological dimension that captures tissue‐level organization [221, 222, 223]. Spatial context is paramount for understanding the tissue microenvironment (TME) and cell–cell communication [222, 223]. Furthermore, by mapping the spatial distribution of immune infiltration and malignant cells, advanced spatial multimodal frameworks can decode spatial organizations that encode critical information relevant to patient prognosis and treatment response [224]. It enables the shift from merely knowing what cells are present to understanding how their spatial organization dictates disease progression.

#### Multiscale resolution and joint representation learning

The data ingestion strategies of models are shifting from single‐resolution learning to cross‐resolution joint modeling. For instance, scTranslator adopts a two‐stage generative approach: it pretrains on paired bulk data to capture broad regulatory patterns and then fine‐tunes on single‐cell profiles to directly infer missing single‐cell proteomes from transcriptomes [225]. Similarly, rather than relying on a single modality, the OmniCell framework integrates single‐cell and spatial resolution data to simultaneously capture intracellular expression and spatial tissue topology [226]. Notably, cross‐scale integration can also be hardwired structurally. Rather than relying on multi‐assay inputs, PULSAR anchors single‐cell transcriptomes with molecular‐level protein sequence embeddings; its hierarchical design then funnels these signals from individual genes up to multicellular systems, thereby yielding donor‐level representations for disease classification [227].

This cross‐resolution integration is a critical methodology in precision oncology. By bridging multilevel data, AI lays the groundwork for constructing mechanistic simulators. This significantly enhances our ability to perform in silico prediction of cellular perturbation responses, thereby bridging the gap between molecular profiling and clinical decision‐making.

### Historical evolution: From pre‐foundation models to the artificial intelligence virtual cell

From an academic research perspective, this subsection chronologically traces the evolution of the domain on the basis of the publication timelines of key methods. Prior to the deep‐learning era, the field relied on traditional statistical methods to infer shared latent structures. The central question at that stage was whether integrating multiple assays, each providing a partial and noisy view of the system, could recover a shared biological structure more faithfully than relying on any single modality alone. iCluster addressed this problem using a joint latent variable model [228]. SNF approached it by fusing similarity networks rather than feature matrices [229]. MOFA and MOFA+ extended the latent‐factor view to increasingly complex multimodal and single‐cell settings [230]. DIABLO added a supervised variant that constrained the multi‐omics structure with phenotype prediction [231]. Although these methods differ in formulation, they share the same core objective: recasting multi‐omics integration as the recovery of shared biological organization.

A second generation emerged when deep learning and graph‐based modeling entered the field. At this stage, the problem was not only what was shared but also how different modalities contributed to the definition of the biological state. MOGONET made this explicit by assigning each omics layer its own graph structure and learning cross‐view representations through graph convolution [232]. totalVI addressed the same issue in a probabilistic framework for paired RNA and protein data, demonstrating that a multimodal cellular state could be modeled while accounting for assay‐specific technical effects [233]. WNN analysis introduced an influential insight: the contribution of each modality does not need to be fixed across all cells but can itself be learned [234]. By establishing the principle that modalities would retain their own geometry before being brought into a common latent system, these early frameworks laid a robust foundation.

A third generation emerged as shared latent spaces became expressive enough to support missing‐modality recovery, cross‐modal translation, and perturbation‐aware inference. MultiVI, GLUE, multiDGD, and TMO‐Net are representative of this phase, although they differ substantially in architecture and supervision [235, 236, 237, 238]. Some emphasize robust alignment under incomplete pairing; others incorporate prior regulatory structure or pretraining‐style objectives. In parallel, BABEL and scButterfly turned cross‐modal translation into a central problem rather than a byproduct [239, 240]. During the same period, perturbation models such as scGen, CellOT, GEARS, and CINEMA‐OT shifted the focus from static state description to state transition under intervention [220, 241, 242, 243]. Although the term “virtual cell” was introduced later, earlier computational studies had already explored related ideas, including cellular state representation, cross‐modal prediction, and in silico simulation of responses to perturbations.

**Figure 3:**
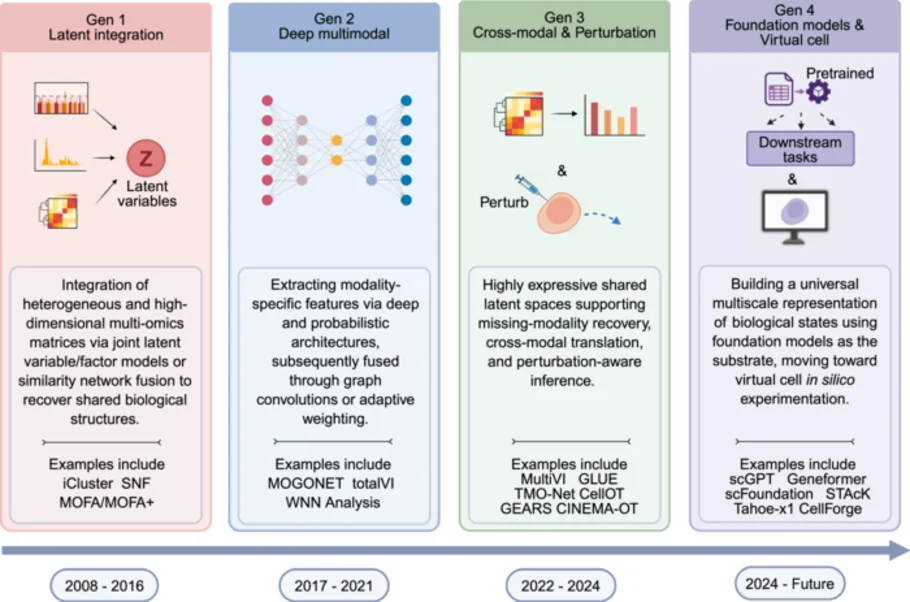
Evolution of artificial intelligence approaches for multi‐omics modeling. Gen 1 multi‐omics studies relied on statistical integration methods to infer shared latent structures across molecular layers. Gen 2 introduced deep multimodal learning architectures that modeled cross‐omics relationships via neural networks and graph‐based methods. Gen 3 expanded to highly expressive latent spaces for cross‐modal translation, missing‐modality recovery, and perturbation‐aware state transitions under intervention. More recently, the Gen 4 paradigm builds on large‐scale biological foundation models as a representational substrate for the virtual cell framework, moving toward transferable molecular representations and *in silico* experimentation across biological contexts.

The current phase is defined by two related developments. The first is the rise of foundation models spanning molecular omics (genomics, transcriptomics, epigenomics, proteomics, metabolomics, and microbiome), spatial‐omics, pathology, and multimodal biomedical data. The second is the emergence of the AIVC perspective, which reframes the broader aim of the field. In the AIVC formulation, the objective is no longer simply to integrate more modalities or to improve a single benchmark. It builds a universal multiscale representation of biological states and virtual instruments that can query, perturb, and simulate that state in silico [46]. From this angle, foundation models are the representational substrate on which a broader program of virtual experimentation may be built (Figure 3).

However, it is crucial to recognize that the dawn of the AIVC era does not render earlier generations obsolete. Rather than representing a complete replacement of established multi‐omics integration methods, foundation models should be viewed as an emerging paradigm that complements current statistically grounded and classical machine‐learning approaches. For instance, MOFA and DIABLO are widely employed to discover shared latent covariance and effectively mitigate the curse of dimensionality, while tree‐based ensembles remain highly valued for their data efficiency and mechanistic interpretability in limited‐sample, low‐dimensional regimes [244]. These classical approaches are constrained primarily by the supervised learning paradigm and strict data alignment requirements. In contrast, multimodal foundation models offer the potential to learn transferable representations across omics layers, spatial contexts, and disease states. However, their clinical translation depends strongly on overcoming data heterogeneity, benchmark standardization, computational demands, and the black‐box interpretability deficit. Therefore, the current landscape of MODPM is a dual paradigm: Established methods remain central as interpretable components, whereas foundation models represent a powerful but still maturing translational direction.

### Architectural framework of multi‐omics artificial intelligence

Despite the broad variation in architecture and application, most recent multi‐omics AI systems can be deconstructed into a layered framework. The foundation of this system is the multi‐omics and multimodal data substrate. Large models need more than large data sets; they require data that are sufficiently standardized, annotated, and diverse to support transfer. This foundational layer integrates diverse data types ranging from molecular measurements (genome, epigenome, transcriptome, proteome, metabolome, and microbiome) to macroscopic and phenotypic modalities (pathology, radiology, and clinical records). As discussed in the “Spatial and Microenvironmental Resolution” section, spatial omics naturally spans both of these dimensions. Therefore, atlas‐building efforts such as hECA v1.0 and hECA v2.0 are methodologically significant and not merely infrastructural [245, 246]. Their importance lies not simply in scale but in placing cells collected across tissues, studies, and modalities into a common frame, thereby enabling cross‐study pretraining and benchmarking.

The central layer focuses on establishing a unified biological representation, which is the core of AI modeling and simulation. Raw assays differ in scale, sparsity, resolution, and semantics; however, compared with raw measurement tables, they can still be projected into latent spaces that stabilize biological states more effectively. Earlier models usually learned such spaces for one task on one dataset. Foundation models change the purpose of this layer by treating representation itself as a reusable object, learned once on broad corpora and adapted repeatedly across downstream settings [211, 247, 248, 249]. This step turns a model from a specialized predictor into a biological interface.

This unified representation is enriched by integrating two complementary dimensions of representation learning. Along the molecular‐semantic dimension is cross‐modal relation modeling. Once informative latent spaces are available, the next question is how different layers should be related. Two broad strategies have been used throughout the field. One is alignment, in which modalities are mapped into a shared space that supports a unified notion of cell or sample state under incomplete observation [235, 236, 237, 238]. The other is translation, in which one molecular layer is modeled as a directional transformation of another, such as chromatin to transcription or transcription to protein [225, 239, 240, 250]. Alignment is most useful when the aim is a robust state definition across partially observed data, whereas translation is most informative when the problem itself is directional.

Context modeling addresses the macroscopic and topological dimension. Molecular measurements alone seldom explain tissue behavior or patient outcome. The biological state is context dependent: Cells are shaped by the spatial neighborhood, tissue architecture, histological phenotype, organ‐level imaging, and longitudinal clinical trajectory. Spatial omics models, pathology foundation models, radiological integration, and EHR‐linked multimodal systems all fall under this category, as they enrich molecular states with tissue‐ and patient‐scale information [208, 209, 216, 218, 222, 223, 226, 251, 252, 253, 254, 255, 256, 257, 258, 259, 260, 261, 262, 263, 264, 265, 266, 267, 268, 269, 270]. This integration therefore changes the object of analysis from isolated molecular states to situated biological states.

Building upwards from the central hub is intervention‐oriented modeling, which extends to the analysis of state transitions and responses to systemic perturbations. These capabilities are enabled by causal transport, perturbation prediction, and flow‐ and diffusion‐based transition models, eventually culminating in the AI Virtual Cell for in silico experimentation [43, 44, 45, 46, 220, 241, 242, 243, 271, 272, 273].

Finally, this entire simulation core is designed to drive precision medicine applications, translating computational insights into patient stratification, therapy selection, response prediction, and clinical decision support. In this architecture, the data infrastructure provides the foundational substrate, and cross‐modal and contextual modeling shape the unified representation, which in turn drives intervention‐oriented modeling to establish the AI Virtual Cell for downstream clinical applications.

**Figure 4:**
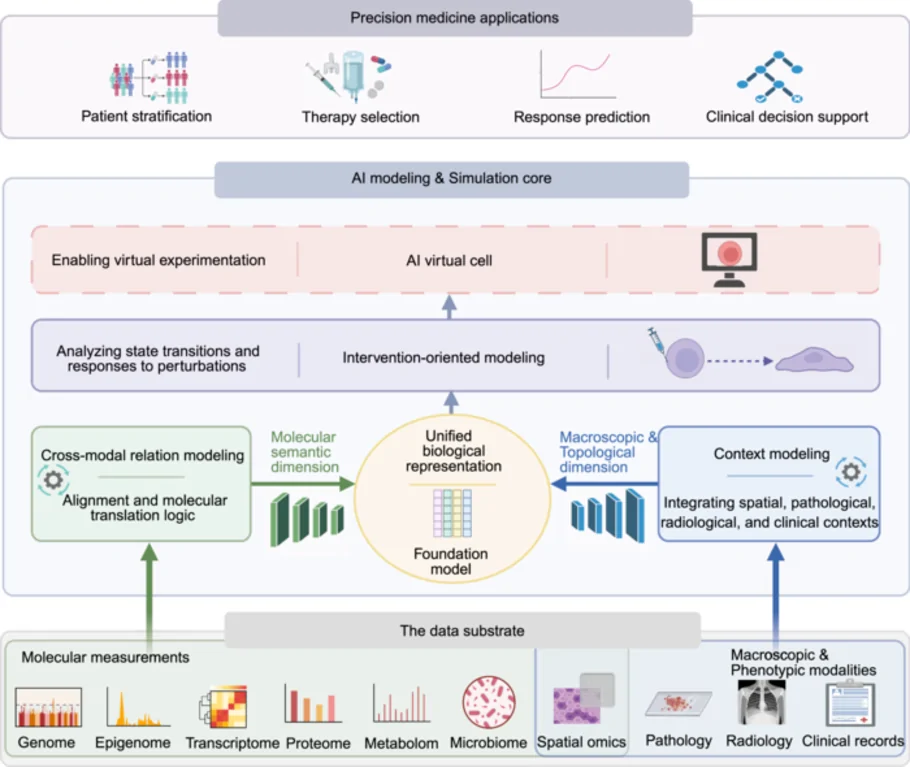
Architectural framework of AI‐driven multi‐omics modeling. The framework consists of three layers. The data substrate integrates diverse inputs across two scales: molecular measurements (genome, epigenome, transcriptome, proteome, metabolome, and microbiome) as well as macroscopic and phenotypic modalities (pathology, radiology, clinical records). Spatial omics spans both domains. Within the AI Modeling & Simulation Core, a unified biological representation (foundation model) is enriched by integrating two complementary dimensions: cross‐modal relation modeling performs alignment and translation of molecular layers, whereas context modeling integrates spatial and clinical topologies. This unified representation functions as a reusable substrate to enable the intervention‐oriented modeling of state transitions and perturbations, establishing an AI Virtual Cell for in silico experimentation. The top layer outlines subsequent precision medicine applications, including patient stratification, therapy selection, response prediction, and clinical decision support.

This framework also explains why foundation models have become an important emerging substrate. While earlier methods often solved one task on one dataset, foundation models aim to provide a reusable substrate that supports many downstream analyses without rebuilding the representation each time [211, 247, 248]. They improve transfer across assays and cohorts, make missing‐modality analysis less brittle, and allow reuse of the same latent space for descriptive tasks, cross‐modal inference, and perturbation‐oriented reasoning. This is also the point at which multi‐omics AI has begun to converge with broader multimodal AI in medicine because pathology, radiology, and clinical records become part of the representational system (Figure 4).

### Molecular foundation models across omics layers

#### Genomic foundation models

Functionally meaningful variation can occur at single‐nucleotide resolution, yet regulatory dependencies can span long genomic intervals. Genomic foundation models have emerged to address this problem. Early models such as DNABERT adopted the Transformer architecture with k‐mer tokenization and showed that local sequence semantics could be captured through pretraining [40]. This was an important proof of concept, but it also revealed the limitations of standard self‐attention when very long genomic contexts were involved. DNABERT‐2 and the Nucleotide Transformer improved the practicality and breadth of genomic pretraining through more efficient tokenization and large multispecies corpora [212, 274].

Other models confronted the long‐range problem more directly. Enformer combined convolutional compression with attention to increase the receptive field and improve regulatory prediction over long sequence ranges [275]. HyenaDNA approached the same issue from the computational side, replacing standard attention with subquadratic sequence operators to make million‐token contexts feasible while retaining nucleotide‐level inputs [276]. Evo extended this logic further, showing that sequence modeling and design could be pushed from molecular to genome‐scale settings [277]. More recently, AlphaGenome and Genos have shifted their attention toward more explicitly human‐centered regulatory modeling, aiming to combine long‐range sequence context with clinically relevant variant interpretation [278, 279]. Although these models are not multi‐omics methods in the narrow sense, they are highly relevant because they turn regulatory sequences into a reusable representational layer that can later be connected to chromatin and transcriptional states.

#### Epigenomic foundation models

Epigenomic foundation models address a related yet distinct problem. Whereas transcriptomics reflects downstream cellular activity, epigenomic assays often capture upstream regulatory potential in a highly sparse form. Therefore, early deep‐learning work in this area focused heavily on imputation. DeepCpG is a pioneering model for single‐cell DNA methylation state prediction [280]. GraphCpG introduced the graph structure into methylome imputation [281]. scMeFormer extended this direction with Transformer‐based modeling that combined sparse methylation observations and sequence context [282]. These models were still largely task specific, but they established the feasibility of learning structured regulatory representations under severe sparsity.

EpiGePT has broadened the field by integrating sequence context, trans‐acting factors, and chromatin interactions to model context‐specific epigenomic outputs [283]. EpiAgent marked a further step toward a genuine single‐cell epigenomic foundation model by representing chromatin accessibility patterns as “cell sentences” and learning reusable regulatory‐state representations that support annotation, imputation, and in silico perturbation‐related analyses [249]. GET complements this perspective by linking chromatin accessibility and sequence features to transcriptional output across human cell types [284]. Consequently, epigenomic foundation modeling is becoming an upstream representational layer in multi‐omics AI rather than solely a tool for sparse‐matrix repair.

#### Transcriptomic foundation models

Transcriptomic foundation models are currently the most mature molecular foundation models in biomedicine. Single‐cell transcriptomic data pose a different set of challenges from genomic sequences: cells are sparse and noisy, batch effects are substantial, and gene expression is quantitative rather than symbolic [219, 285]. Early self‐supervised approaches such as scPretrain have already suggested that cell‐level pretraining could improve annotation and transfer [286]. The field changed more decisively when language‐model‐inspired frameworks began to treat cells themselves as pretrainable objects.

scBERT showed that very large scRNA‐seq corpora could support the learning of transferable cellular embeddings [287]. Geneformer built on similar intuitions but emphasized contextualized gene embeddings and demonstrated utility for regulatory and disease‐related tasks [288]. tGPT explored large‐scale generative pretraining from transcriptomes [247]. scGPT designs a generative framework that supports multiple downstream analyses within a shared representation [219]. However, these early milestones relied on lossy discretization or rank ordering, thereby failing to preserve the full resolution of continuous transcriptomic signals [289].

A key step came with the scFoundation. After training on more than 50 million human single‐cell transcriptomes, the scFoundation revealed that one pretrained representation could be reused across annotation, gene expression enhancement, perturbation prediction, and tissue‐level drug response prediction [248]. CellFM extended transcriptomic pretraining to 100 million human cells [289]. In addition to scaling data, architectures evolved to capture richer biological contexts and generative capabilities. The CellPLM added a complementary idea by emphasizing the population context rather than treating each cell as fully isolated [290]. scMulan introduced unified “c‐sentences” to enable multitask generative pretraining from scratch [291]. STAcK suggested that some biological reasoning tasks may eventually be performed in context, without retraining parameters for each new problem [292]. A parallel line of work has attempted to interface omics data more directly with general‐purpose language models. Cell2Sentence, larger‐scale Cell2Sentence variants, and CellHermes reformulate cellular information in text‐like or instruction‐like forms to allow language‐model machinery to be reused in omics analysis [293, 294, 295]. Other approaches, such as GenePert, use large language models more lightly as sources of semantic priors rather than as direct processors of molecular matrices [296]. Together, these studies show that transcriptomic foundation modeling is increasingly concerned with building reusable cell representations that can be transferred, queried, and integrated across analytical settings.

#### Proteomic foundation models

Proteomic foundation modeling is developing along two partially distinct paths. The first, currently more mature, is protein sequence and structure modeling. Large protein language models, represented by the ESM series and ProtTrans, have shown that evolutionary‐scale pretraining on amino acid sequences can recover representations that are informative about structure and function [213, 297, 298]. This has enabled the paradigm in this field to evolve from methods heavily relying on multiple sequence alignments (MSAs), such as AlphaFold2 and RoseTTAFold, to approaches that directly perform end‐to‐end prediction from single sequences, exemplified by ESMFold [41, 213, 299]. Building on this, generative architectures such as ProtGPT2 and the multimodal model ESM3 have extended this capability to de novo protein design [300, 301]. To address the semantic blind spots associated with the complex biological functions of pure sequence models, OntoProtein explicitly injects structured knowledge graphs into pretraining [302]. In addition, instead of being confined by predefined ontologies, PROCYON interleaves free‐text phenotype descriptions with protein sequences, protein structures, and small molecular structures [303]. However, as zero‐shot prediction methods often struggle to map to protein activity, frameworks such as EVOLVEpro combine a top‐layer regression model with a protein language model. By utilizing few‐shot active learning, they can successfully navigate the protein activity landscape [304]. This line of work does not operate on measured proteomic abundance directly, but it provides an important functional layer for interpreting how sequence constrains molecular behavior.

The second path is cell‐state proteomics, which is more directly connected to multi‐omics integration. Compared with transcriptomics and epigenomics, measured proteomics remains more heterogeneous across platforms, and foundation‐model ecosystems are correspondingly less mature. Nonetheless, protein‐level inference is becoming increasingly important in multimodal settings. For example, scTranslator shows that protein abundance can be treated as a learnable endpoint of single‐cell representation learning via structured decoding from upstream transcriptomes [225]. Conversely, scPROTEIN demonstrates that measured proteomic abundance can be directly modeled using multitask heteroscedastic regression and graph contrastive learning to resolve peptide uncertainty and batch effects [305]. This does not yet amount to a mature proteomic foundation ecosystem, but it indicates how proteomics may enter multi‐omics AI both through direct protein modeling and through structured decoding from upstream molecular representations.

#### Metabolomic foundation models

Metabolomic foundation models are currently at an even earlier stage of development, but they may prove especially important for precision medicine because metabolite profiles are closely linked to phenotype, physiology, and treatment response. MetaboLM provides an early example of Transformer‐style pretraining on large‐scale plasma metabolomics data for disease prediction and risk stratification [306]. Building upon this, recent frameworks extend isolated metabolic profiles by integrating complementary data structures. PanMETAI incorporates tabular clinical parameters, and MetaKG leverages structured knowledge graphs [307, 308]. Current model ecosystems remain relatively small, yet metabolomics adds a more immediate view of host state and system output that upstream molecular layers do not provide. Thus, the learning of metabolomic representations may become increasingly important in settings where risk monitoring, treatment toxicity, and disease progression matter as much as the diagnosis itself does.

#### Microbiome foundation models

Microbiome foundation models extend representation learning beyond host‐derived molecular layers to the microbial communities that influence host phenotype. Microbiome profiles are compositional and record relative rather than absolute abundances. Early models such as the MGM used rank‐based, genus‐level encodings that discarded abundance information [309, 310]. BiomeGPT is instead pretrained on species‐level profiles with abundance‐aware tokenization, capturing cross‐species dependencies for host health prediction [310]. A self‐supervised Transformer approach learns contextualized taxon embeddings, representing the same taxon differently according to its community context and thus recovering taxonomic and microbial metabolic pathway structure without explicit supervision [311].

Because profile‐level models still treat taxa as discrete tokens, recent work has turned to the underlying nucleotide sequences. At the unassembled sequence level, FINGERS‐7B operates on quality‐aware tokens from raw reads to predict metabolic pathways without taxonomic aggregation [312]. Similarly, Genos‐m, a microbiome‐oriented mixture‐of‐Experts model distinct from the host‐regulatory Genos above, provides reusable genome embeddings from as few as 10,000 reads and improves disease classification over abundance baselines [313]. Moving beyond raw reads to capture long‐range genomic architectures at a larger scale, the EDEN family (28B parameters, 9.7 trillion tokens) shows that autoregressive transformers retain better scaling efficiency than linear‐time alternatives do, with the scaling exponent depending on assembly quality rather than parameter count alone [314]. These models hold up better than traditional machine learning when the data distribution shifts, and they can be generalized across independent cohorts. Their advantage over well‐tuned baselines is nonetheless limited, and accuracy can decrease sharply on external cohorts that differ substantially from the training data [310, 311].

### Multi‐omics integration paradigms in the foundation‐model era

Current multi‐omics integration methods are generally divided into two complementary paradigms: alignment and translation.

Alignment‐based methods ask how a stable representation of a biological state can be constructed across modalities. MultiVI, GLUE, and multiDGD are representative examples, although they differ in how they treat pairing, priors, and probabilistic structure [235, 236, 237]. Their shared goal is to define a cell or sample representation that remains meaningful even when one or more modalities are absent. This is especially important in clinical settings, where complete multimodal pairing remains uncommon. TMO‐Net extends the same logic toward oncology‐oriented multitask settings [238]. SCARF pushes it further into a foundation‐model regime for RNA and ATAC integration [250].

Translation‐based methods begin from a different question: How does one molecular layer give rise to another? BABEL was an early demonstration that transcriptomic and chromatin states can be translated at single‐cell resolution [239]. scButterfly generalized the same idea in a variational setting while emphasizing the retention of cellular heterogeneity [240]. As a pretrained generative foundation model, scTranslator carried this direction into RNA‐to‐protein prediction [225].

In conceptual terms, alignment and translation do not compete; they answer different biological questions. Alignment is most useful when the aim is robust state definition. Translation is most useful when the aim is directional or mechanistic reasoning. Foundation models do not replace either paradigm. Rather, they strengthen both. Shared pretrained representations make alignment less brittle across data sets, whereas broader pretraining can stabilize translation functions that would otherwise remain highly cohort specific. In the foundation‐model era, therefore, both become more reusable.

### Multimodal extensions beyond conventional omics

#### Spatial multi‐omics and tissue context

The expansion from dissociated omics to spatial and tissue‐level context has been among the most significant recent changes in the field. NicheFormer framed spatial and single‐cell omics from a foundation‐model perspective, emphasizing neighborhoods and tissue context rather than isolated cells [251]. Earlier spatial AI methods, such as SpaGCN, STAGATE, GraphST, and SpaceFlow, have already shown that graph‐based approaches can incorporate location into gene expression analysis [208, 209, 252, 253]. SpatialGlue and COSMOS extended this logic to spatial multi‐omics [254, 255]. GROVER further explored adaptive fusion between spatial omics and vision [256]. More recent spatial foundation‐model efforts (including Novae, scGPT‐spatial, OmniCell, SToFM, stFormer, SpatialFormer, and CELLama) go beyond domain identification and begin to learn transferable tissue‐context representations across platforms and resolutions [222, 226, 257, 258, 259, 260, 261]. A conceptual change is underway: Spatial context is increasingly treated as part of state representation rather than as an annotation layered on it.

#### Histopathology and computational pathology foundation models

In many diseases, especially cancer, tissue architecture and morphology convey information that cannot be reconstructed from dissociated molecular measurements alone. Computational pathology has become a major branch of multimodal biomedical AI. CONCH established a vision‐language foundation model for pathology, showing that histopathology images and biomedical text can be co‐embedded in a transferable representational space [262]. TITAN moved further by learning multimodal whole‐slide representations, shifting the field from patch‐level recognition to patient‐ and slide‐level embedding [263].

In addition, ROAM advances weakly supervised glioma diagnosis by optimizing task‐specific whole‐slide feature representation under a joint slide‐ and instance‐level (ROI) supervision framework, highlighting diagnostically meaningful morphology while elucidating its correlation with associated molecular markers [264]. In glioma, where the histological pattern and molecular classification are tightly coupled, this is particularly instrumental. ROAM illustrates how pathology representations are becoming simultaneously more clinically usable and more biologically informative.

#### Visual–omics coupling

Another important multimodal direction is the direct integration of histology and omics data. For instance, OmiCLIP learns a visual–omics foundation model that links histopathology with spatial transcriptomics [265]. This reflects a broader shift in image–omics analysis, from simple cross‐modal prediction, such as inferring gene expression from images, toward joint representation learning in which morphological and molecular information can be compared and interpreted together. Related methods, including THREADS and ST‐Align, extend this strategy by aligning pathological features with spatially resolved molecular states [223, 266]. Beyond cross‐modal alignment, vision transformers now directly model spatial omics signals as segmentation‐free visual inputs. For instance, SubCell and KRONOS encode subcellular organization and multiplexed architecture from fluorescence patches, whereas MetaboFM extracts spatial–spectral tensors directly from mass spectrometry imaging data [224, 315, 316].

Other methods in this space remain more task focused. PAMT integrates pathological images and gene expression with an emphasis on pathway‐aware interpretability in cancer survival analysis [267]. CMRCNet uses cross‐modal mask reconstruction and contrastive learning to predict spatial transcriptomics from histopathology [268]. Thor extends the bridge between histology and molecular spatial information toward cell‐level investigations [269]. The reverse direction is also developing. Gene‐to‐image generative models such as RNA‐conditioned WSI synthesis and GE2Hist suggest that molecular states can be translated back into tissue‐level appearance, opening a complementary route to studying the relationships between the microenvironment, morphology, and underlying molecular state [216, 218, 270].

#### Clinical and radiological context

When moving from tissue to patient, clinical records and radiological data act as natural contextual layers rather than supplementary non‐omics metadata. COMET uses transfer learning from large EHR corpora to strengthen multimodal analyses in smaller omics studies, showing that longitudinal clinical information can act as a prior for omics‐based modeling [317]. Radiology plays a similar role at a different scale. It provides lesion‐, organ‐, and whole‐body information that is often missing from biopsy‐derived molecular data, making it an important candidate for multiscale integration in precision oncology [318].

More broadly, recent oncology reviews have argued that multimodal AI moves beyond mere data accumulation to model disease simultaneously at molecular, histological, imaging, and clinical scales [319, 320]. Consequently, pathology, radiology, and EHR serve as patient‐scale extensions of multi‐omics modeling.

### Perturbation modeling and the artificial intelligence virtual cell horizon

Perturbation modeling marks the transition from descriptive integration to intervention‐oriented inference. Early approaches established the foundation for single‐modality perturbation prediction: scGen showed that latent representations could be used not only to encode observed states but also to estimate how those states change under perturbation [241]. CellOT reframed the problem in terms of optimal transport between control and treated populations [220]. GEARS introduced graph priors to improve generalization to previously unmeasured multigene perturbations [242]. CINEMA‐OT added a stronger causal orientation by attempting to separate perturbation effects from nuisance variation [243].

However, these models are confined primarily to single‐modality transcriptomic data. Because distinct omics modalities capture cellular states at fundamentally different molecular hierarchies, integrating these complementary modalities is crucial for overcoming single‐modality constraints and comprehensively characterizing cross‐layer molecular effects [321]. Perturbations generate profound cascade effects across different molecular modalities, simultaneously reshaping transcriptional programs and protein abundance—and proteins, acting as direct functional executors, ultimately dictate cellular phenotypes [321]. Compared with transcriptome‐only models, multi‐omics approaches provide a mechanistic view of how upstream regulatory changes propagate into downstream functional and clinical outcomes. By learning these cross‐layer cascade responses, AI‐based multi‐omics perturbation models can prioritize drug targets, predict resistance mechanisms, and simulate cell‐type‐specific responses before experimental testing [322].

More recent models such as PerturbNet, STAMP, CellFlow, and Squidiff have expanded this space further, moving toward generalizable perturbation prediction, subtask‐aware decomposition, continuous flow‐based transitions, and diffusion‐style state propagation [43, 44, 45, 271]. Scouter reported that language‐derived priors can also be integrated into perturbation prediction [272]. The latest foundation models significantly extend the reach of generalization, with Tahoe‐x1 leveraging billion‐parameter pretraining for zero‐shot transfer and STAcK entirely bypassing task‐specific fine‐tuning via in‐context learning [292, 323].

These methods reflect a shift in modeling goals. The biological state is no longer treated as a descriptive target. It is treated as a state that can be transformed within a learned space under hypothetical intervention. Thus, perturbation modeling marks the stage where multi‐omics AI begins to approach virtual experimentation.

In that context, the AIVC framework offers a solution. The cell perspective on AIVC argues that a useful virtual cell should combine a universal multimodal and multiscale representation of biological states with virtual instruments capable of querying, perturbing, and simulating that state in silico [46]. Under this view, foundation models are essential prerequisites whose value lies in making biological state representations broad and stable enough that intervention, simulation, and mechanistic reasoning become feasible across modalities and contexts.

The emergence of agentic systems, such as CellForge, hints at a further progression from static virtual prediction to the autonomous agentic design and synthesis of tailored neural network architectures [273]. Whether such systems become scientifically useful depends less on the novelty of the agent itself than on the quality and biological faithfulness of the underlying state representations and transition models.

Despite its promise, the AI Virtual Cell remains an aspirational framework rather than a mature clinical tool. Current AIVC development is constrained by incomplete multi‐omics coverage [324], the limited availability of paired perturbation data sets [43], inconsistent temporal and spatial resolution [325, 326], and substantial computational cost [279]. Moreover, current predictive models often operate as black boxes and lack the capacity to provide clear, verifiable mechanistic explanations before they can safely guide therapeutic decisions [294]. Ethical, privacy, accessibility, and regulatory issues also need to be addressed, particularly when large‐scale patient‐derived data are used for model training and deployment.

### From representations to precision medicine

The clinical relevance of these developments lies in their translational impact at the patient level. First, shared and multimodal representations support patient stratification, especially when clinically meaningful subtypes cannot be resolved from a single assay. In oncology, subtype definition depends increasingly on the molecular state, histological phenotype, and treatment history, considered together rather than separately [317, 318, 319, 320].

Second, these models support therapy selection. Treatment decisions rarely follow from one molecular layer alone. A transcriptomic signature may indicate pathway activity; pathology may reveal tissue architecture and immune context; radiological information may capture lesion burden and spatial extent; and clinical records may encode prior treatment response and toxicity. The value of multimodal AI lies in bringing these layers together without collapsing them into one undifferentiated feature table. Foundation‐style representations are especially promising here because they provide a shared substrate on which these different evidentiary layers can be compared or integrated.

Third, perturbation‐aware models support response and resistance prediction. This represents the emergence of a framework where treatment hypotheses can be explored in silico before, or alongside, experimental validation. In that sense, perturbation modeling, AIVC, and precision medicine are successive levels of the same problem: how to move from molecular description to actionable therapeutic reasoning.

Finally, EHR‐linked and multimodal systems open the possibility of longitudinal monitoring and closed‐loop learning. If patient outcomes can be fed back into the same representational system that was used for initial modeling, then prediction, treatment choice, and real‐world evidence no longer belong to separate analytic stages. This approach remains aspirational in many settings, but it is already shaping the direction of multimodal AI in oncology and related fields [317, 318, 319, 320].

### Interpretable and explainable AI for multi‐omics precision medicine

Interpretability is a critical prerequisite for the clinical adoption of AI‐driven MODPM. As multi‐omics models increasingly employ complex architectures, such as deep neural networks and giga‐scale multimodal foundation models, to integrate millions of features across heterogeneous molecular layers, they inherently face severe black‐box limitations [291]. Although these highly parameterized models achieve superior predictive performance, their structural opacity fundamentally limits biological validation, clinician trust, and regulatory acceptance. For instance, recent cell language models and single‐cell transformers often struggle to interpret the complex cellular syntax they capture, operating as black boxes that fail to provide verifiable mechanistic explanations for their predictions. Furthermore, the standard attention mechanisms in these foundation models can be limited in capturing gene relationships grounded in static or global biological knowledge unless explicitly guided [289], thereby functioning as opaque black boxes that lack the transparent explanations required for deep mechanistic understanding [327].

To systematically address these limitations, interpretable and explainable AI (XAI) approaches have become indispensable. Current XAI strategies for multi‐omics generally fall into two broad categories: post hoc explanations and inherently transparent models [328]. Post hoc methods employ feature‐attribution techniques such as integrated gradients (IG) to retrospectively map and characterize the effects of individual molecular analytes on clinical outcomes, alongside attention weight visualization and counterfactual analysis to identify key driving variables [238, 243, 306]. Alternatively, to mitigate the risks of deploying opaque architectures in high‐stakes clinical scenarios, transparent models integrate prior biological knowledge directly into the network design, deciphering both the static structural hierarchy and the dynamic temporal mechanisms of biological systems. On the static front, biologically informed visible neural networks (VNNs), such as DrugCell, structure their internal architectures using literature‐curated frameworks such as Gene Ontology, directly linking neural activation to established signaling pathways to ensure human accountability [329]. Complementing this with a logic‐driven approach, emerging paradigms such as biological world models advance white‐box modeling by integrating structured biological knowledge with the iterative reasoning capabilities of large language models. Instead of opaque end‐to‐end mapping, these models generate traceable, step‐by‐step mechanistic explanations for cellular state transitions [330].

In the context of MODPM, effective explanations must move beyond simple feature ranking. They should explicitly connect model outputs to underlying disease mechanisms, cellular contexts, and therapeutic targets to formulate actionable clinical hypotheses [329, 330]. Furthermore, future multi‐omics AI systems must seamlessly combine prediction accuracy with robust uncertainty estimation, biological plausibility, and independent external validation. By transforming black‐box predictions into clinician‐understandable insights, XAI effectively bridges the gap between massive computational models and reliable clinical decision‐making.

### Section summary

This section reviews the landscape of artificial intelligence‐driven multi‐omics modeling, tracing its evolution from early latent integration to advanced foundation models and the artificial intelligence virtual cell paradigm. However, several challenges remain, and they are not merely technical. Paired multimodal data sets are still scarce, especially in clinically well‐annotated cohorts [9]. Cross‐platform and cross‐cohort generalization remains fragile. Although highly relevant to treatment response and host state, protein and metabolite layers still lag genomics and transcriptomics in terms of standardized corpora and mature foundation‐model ecosystems. Microbiome‐level foundation models also remain relatively underexplored. Pathology, radiology, and clinical records add indispensable context, but they also introduce annotation bottlenecks, scale mismatches, and new sources of confounding.

A deeper challenge concerns the meaning of representation itself. The ultimate utility of AI in multi‐omics lies in its ability to transform heterogeneous data into transferable, predictable, and intervenable biological state representations. The value of a latent space depends on its biological accuracy across molecular modalities, tissue contexts, and patient populations rather than on scale alone. This requirement becomes more demanding as the field moves from integration to virtual experimentation. A model that performs well on a benchmark may nevertheless be biologically misleading when it is asked to support perturbation simulation, therapeutic prioritization, or extrapolation across populations [331].

Moreover, the inherent black‐box nature of transformer‐based architectures continues to limit mechanistic interpretability [332]. Overcoming this opacity requires not only high‐quality data and robust training strategies but also strict critical appraisal by domain experts and specialized frameworks to detect algorithmic errors [333].

The next phase of the field will therefore be shaped less by whether models can become larger and more by whether they can remain meaningful as they become broader. The central problem is no longer limited to how to integrate modalities but rather how to learn biological representations that remain coherent across scales and stable enough to support intervention‐oriented reasoning. If this standard is met, multi‐omics AI will evolve beyond a collection of integration tools to become part of the computational substrate for precision medicine.

## MULTI‐OMICS IN DRUG DEVELOPMENT

Despite decades of biomedical advances, drug discovery remains prohibitively costly and inefficient: The average cost to translate a new agent to market approaches $2.23 billion, and clinical success rates are below 10% [334, 335]. The increasing reliance on the Orphan Drug Designation framework, a cost‐effective mechanism that grants market exclusivity and financial incentives for more than one‐third of new FDA approvals in the 2010s [336, 337], further underscores the productivity challenge in the pharmaceutical industry. This phenomenon reflects “Eroom's Law,” the inverse of Moore's law, which describes the consistently decreasing R&D efficiency [49]. While the decline has recently plateaued [338], the underlying disconnection between scientific progress and therapeutic yield persists. The contributing factors include safety concerns and poor efficacy, with many new agents failing to outperform generics [339]. Fundamentally, this stems from an incomplete understanding of disease biology: oversimplified models may lack precision for targeted intervention.

To understand how multi‐omics addresses these challenges, it is essential to consider the pharmacokinetic and pharmacodynamic framework. Pharmacokinetics describes absorption, distribution, metabolism, and excretion, namely, how the body affects a drug. Pharmacodynamics describes how a drug affects the body, including target engagement, pathway modulation, and downstream therapeutic outcomes. Conventional pharmacokinetic/pharmacodynamic modeling typically relies on limited endpoints, which may fail to capture system‐wide drug actions or interindividual variability. Multi‐omics fills this gap by providing high‐dimensional molecular readouts across tissues and time points. For instance, transcriptomic and proteomic data reveal target abundance and engagement, whereas metabolomics profiles metabolizing enzymes and consequent tissue exposure. Embedding pharmacokinetics and pharmacodynamics into a multi‐omics landscape enables more reliable and personalized drug discovery, offering a strategy to address these challenges.

**Figure 5:**
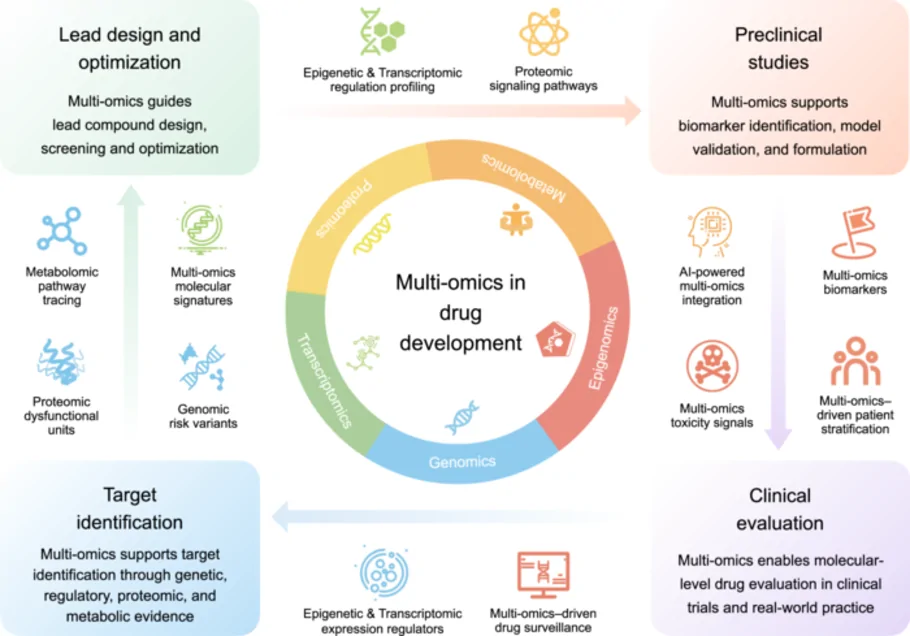
Multi‐omics in drug development. Multi‐omics is reshaping the entire drug development pipeline. From target identification, lead design and optimization, to preclinical validation and clinical evaluation, AI‐driven multi‐omics approaches provide a comprehensive molecular‐level view across all stages of drug development. This integrated perspective thereby lays the foundation for the clinical practice of precision medicine. This diagram is illustrated with Microsoft PowerPoint.

Moreover, unlike conventional pathophysiological views, AI‐powered multi‐omics analysis enables the comprehensive characterization of disease mechanisms. Such integration is synergistic, not merely additive: It uncovers causality, distinguishes driver alterations from passenger events, and maps network‐level consequences of therapeutic interventions [340]. At present, multi‐omics is systematically reshaping the drug development pipeline. It guides multidimensional variant identification and target‐to‐lead optimization [341, 342, 343], refines preclinical models and pharmaceutical formulation [344, 345, 346], and provides analytical frameworks that can inform trial design and postmarket surveillance [347], paving the way for a more reliable foundation of precision medicine from bench to bedside (Figure 5).

### Multi‐omics in target identification

Rational drug design (RDD) is based on the precise modulation of disease‐associated biomolecules, known as targets [348]. For decades, target identification has relied on phenotypic screening and target deconvolution in animal models [349], frequently yielding targets with uncertain pathological relevance [350]. Multi‐omics is overcoming this ambiguity, accelerating target identification across genetic, regulatory, functional, and metabolic dimensions.

#### Genomics in target identification

Among multi‐omics molecular layers, genomics holds prominence. Retrospective analyses of approved therapeutics have demonstrated that targets supported by genetic evidence are 2.6 times more likely to achieve market approval [351], highlighting the value of genomics in target identification.

Genome‐wide association studies (GWASs) have played a pivotal role in detecting genetic variants linked to complex diseases, offering etiological insights unconstrained by conventional pathophysiological assumptions. For instance, large‐scale GWASs have implicated rare mutations in *BAG3* as a cause of dilated cardiomyopathy [352], leading to the development of RP‐A701, a gene therapy candidate that has recently received FDA clearance for clinical investigation [353]. Despite these advances, several limitations persist: risk loci are frequently located in noncoding regions, and linkage disequilibrium complicates causal inference [354]. QTL analysis and Mendelian randomization are typically employed to refine causal inference and address these challenges.

Nevertheless, genomics alone provides only a static blueprint of disease pathogenesis, offering limited insight into dynamic functional changes. Its utility in target identification must therefore be validated by using downstream data that reflect transcriptional and functional alterations.

#### Epigenomics and transcriptomics in target identification

By illuminating the dynamic regulation of gene expression, epigenomics and transcriptomics provide critical evidence for target identification, particularly in complex diseases such as cancer and rheumatoid arthritis, where environmental and lifestyle factors exert significant influence [355, 356].

Epigenetic modifications (e.g., DNA methylation and histone acetylation) not only constitute therapeutic targets but also reflect pathophysiological states or pharmacological responses [343, 357]. Transcriptomics enhances the resolution of driver gene and subtype identification. This is illustrated by the BIOSTAT‐CHF study, which revealed 1153 differentially expressed genes in fatal heart failure [358]. Such inferences, however, are susceptible to false positives, as accumulated noise in large‐scale RNA‐seq may be misidentified as signals [359]. One approach to mitigate this limitation is the introduction of temporal information, which facilitates causal inference by ordering regulatory events [360]. Further integrative strategies leveraging multi‐omics are discussed in Section Multi‐omics‐driven target validation.

However, transcript abundance does not invariably correlate with protein levels because of posttranscriptional and translational regulation. Proteomic validation is therefore essential for establishing functional consequences and elucidating underlying mechanisms, thus bridging the gap between transcriptional signatures and therapeutic relevance.

#### Proteomics in target identification

As primary executors of biological function, proteins predominate among current drug targets. Proteomics thus offers a direct lens into the involvement of these functional molecules in disease pathogenesis. In addition, profiling protein expression and subcellular localization is crucial for assessing protein accessibility, which is a critical step in target validation. This section covers structural and functional proteomics.

Structural proteomics approaches, such as density gradient centrifugation and X‐ray crystallography, resolve protein subcellular localization and structures [361, 362], thereby identifying potential therapeutic targets. However, the structural heterogeneity of macromolecules may lead to nonphysiological conformations in isolated proteins [361]. Although cryo‐EM offers a solution, its cost limits accessibility. An alternative strategy is activity‐based protein profiling, which uses chemical probes to identify functional proteins or their subunits that interact with specific ligands. This technique has proven valuable in target discovery for natural products with unknown pharmacological mechanisms [363].

Functional proteomics identifies targets within protein–protein interaction networks and signaling pathways. For example, the use of Akt, a signaling hub in multiple diseases, has achieved considerable translational success [364], emphasizing the value of network‐centric approaches for target prioritization. However, the precise quantification of key signaling and regulatory proteins remains challenging because of their typically low abundance and the specific posttranslational modifications that govern their functional states. Enrichment strategies, including immunoprecipitation [365] and affinity chromatography [366], have been employed to increase analytical sensitivity and accuracy [367].

#### Metabolomics in target identification

Metabolomics captures terminal products of biological regulation, reflecting how upstream omics layers respond to stimuli. Consequently, this capacity is particularly effective for identifying dysregulated pathways and, in turn, tracing their key components as candidate targets. For instance, enhanced glycolysis in cancer cells suggests hexokinase as a potential therapeutic opportunity [368]. Similarly, metabolomics has proven especially insightful for studying tissues with distinctive metabolic characteristics, such as the high energy demand of the heart. Marked aberrations in acylcarnitine metabolism have been reported in heart failure patients, revealing that mitochondrial dysfunction is an actionable therapeutic target [369]. Integrative approaches combining metabolomics with transcriptomics have also revealed novel therapeutic mechanisms (see Section 5.1.5).

Despite these achievements, a major challenge in metabolomics stems from the complexity of metabolic pathways. This complexity renders conventional analytical platforms inadequate for the comprehensive and precise capture of all relevant metabolites. Moreover, many metabolites undergo extensive degradation *in vivo*, potentially leading to the loss of critical information. To overcome such obstacles, dynamic isotopic tracing, following metabolic flux through interconnected pathways, has emerged as a solution [370].

#### Integrative multi‐omics strategies for target identification

While individual omics layers provide valuable clues, the convergence of multi‐omics data offers a robust strategy for revealing therapeutic targets. A foundational approach is mapping regulatory, expression, and metabolomic data onto the genome, which enables the identification of causal genes that lie beyond the reach of conventional association studies. A representative and rapidly developing method in this direction is the integration of single‐cell RNA sequencing (scRNA‐seq) with expression quantitative trait loci (eQTLs). scRNA‐seq resolves disease‐associated transcriptional programs at single‐cell resolution. When paired with eQTL data, this integration links cell type‐specific genetic signals to the effector genes and the exact cell populations from which pathogenic phenotypes originate. For instance, this convergence identified IRF1 and MAPRE1 in specific T‐cell subsets as potential therapeutic targets in polycystic ovary syndrome, which was obscured in bulk eQTLs [371]. Nevertheless, scRNA‐seq is expensive and requires specific noise‐reduction methods, factors that currently limit its broad adoption [372].

Beyond genomic–transcriptomic integration, network‐based multi‐omics models have revealed potential disease mechanisms and therapeutic targets. An illustrative case is the multi‐omics strategy termed sCCA‐ACAT, which identified *GMPPB* as a causal gene and potential therapeutic target in depression [373]. In a complementary approach, combining transcriptomic and metabolomic data revealed that gemcitabine induces the accumulation of AMP and cAMP, thereby activating AMPK and PKA signaling pathways to suppress pancreatic cancer [374]. More recently, spatial multi‐omics approaches have gained traction for target identification. Spatial transcriptomics preserves information on cell and subcellular distributions during gene expression analysis, supporting target identification within the MODPM framework [375]. Collectively, these integrative strategies have the potential to filter out false‑positive associations and resolve ambiguities, delivering refined candidate targets for downstream validation.

#### Multi‐omics‐driven target validation

Reliable targets are typically central nodes in pathophysiological networks, with consistent effects across genomic, transcriptional, functional, and metabolic dimensions. Validating identified targets prior to preclinical studies is essential—a lesson from the controversy surrounding the use of amyloid‐β as a therapeutic target in Alzheimer's disease [376]. Multi‐omics, empowered by AI, provides a framework for converging multidimensional evidence, although the integrative strategy introduces the curse of dimensionality, which demands advanced hardware and algorithms [360].

A promising target for further development must demonstrate causality, safety, and accessibility. Causal validation of targets has been strengthened by experimental approaches, such as gene knockout, RNA interference, and selective antagonists [377], which collectively constitute a rigorous framework for establishing target credibility. Safety assessments also benefit from multi‐omics: genomics underscores the risk of targets in genetically susceptible organs; transcriptomics and proteomics identify potential off‐target effects in healthy tissues; and metabolomics reveals unintended consequences of intervention [354]. Accessibility, the susceptibility of a target to modulation by exogenous agents, represents another critical determinant. While easily accessible sites (e.g., exposed protein domains) are generally amenable, functional cores of macromolecules are often structurally concealed, as exemplified by the buried active site of the SARS‐CoV‐2 main protease [378]. This inaccessibility poses substantial hurdles for lead compound development, yet it has paradoxically accelerated the deployment of advanced prediction tools such as AlphaFold [41]. When combined with pocket‐detection algorithms, such tools may identify cryptic binding sites that elude conventional methods.

### Multi‐omics‐driven lead design and optimization

Lead compounds, which are molecules with defined bioactivity against a target [348], serve as the bridge between target validation and drug candidates, although further optimization of their pharmacokinetics, safety, and efficacy is typically needed. Multi‐omics technologies influence multiple stages of lead development, including molecular design, compound screening, and iterative optimization.

#### Multi‐omics‐driven structure‐based drug design

Structure‐based drug design (SBDD) aims to generate corresponding ligands on the basis of the spatial structure of the target. Nevertheless, the de novo design of a safe and effective lead remains exceedingly challenging without sufficient clues. Multi‐omics embeds structural information within the broader context of dynamic pathophysiological systems, thereby advancing SBDD.

Proteomics and structural biology elucidate protein structures, particularly functional domains and disease‐associated conformations, and provide critical information on protein–protein interaction networks and posttranslational modifications. These insights offer essential data for the design of leads targeting specific proteins. For instance, phosphorylation at key sites may alter the charge distribution of ligand‐binding pockets [379], a finding that suggests the introduction of affinity‐enhancing groups or moieties with steric hindrance to exclude competitors [380]. Through exon sequencing and RNA‐seq, genomics and transcriptomics provide the primary sequences of protein targets while elucidating expression profiles and genetic polymorphisms [381]. This integrated view allows the rational design of leads tailored not only to the target structure but also to its genetic and regulatory heterogeneity, facilitating effective validation of target engagement.

Multi‐omics‐driven algorithms and AI are further accelerating the progress of SBDD. Models such as PandaOmics leverage omics data to predict protein structures and conformations [382]. Constrained by the current understanding of proteomics, such predictions are not always reliable, especially for proteins that remain understudied and have been captured only in static snapshots [383]. The orphan receptor GPR139 illustrates this point: AlphaFold accurately predicts its overall fold but fails to correctly model the dynamic conformation of its ligand binding pocket [384]. For such targets, synergistic integration of cryo‐EM experimentation and AI‐powered SBDD is therefore recommended.

#### Multi‐omics‐driven ligand‐based drug design

In contrast to SBDD, ligand‐based drug design (LBDD) employs a template‐based strategy in which existing active ligands of the target serve as templates for modification. This highlights a fundamental trade‐off: Although it remains applicable for targets with unknown structure or poor accessibility, LBDD is highly dependent on the thorough elucidation of ligand–target interactions and the *in vivo* behavior of reference ligands, precisely the domains in which multi‐omics excels. Multi‐omics addresses this issue by integrating multidimensional features, including proteomic response patterns and metabolomic perturbation readouts, and assembling fragmented biological clues into key pharmacophores for target modulation. LBDD can be further advanced by identifying endogenous ligands.

Despite its promise, multi‐omics‐driven LBDD faces substantial challenges, particularly data heterogeneity: Omics data sets from disparate spatial and temporal dimensions typically carry distinct pathophysiological meanings, potentially rendering known ligands therapeutically irrelevant. Paradoxically, these inconsistencies can, in some cases, inspire superior leads. A recent study demonstrated that rifampicin rapidly increases the level of CYP3A4 mRNA in hepatocytes, as does its derivative rifabutin. However, the latter induces upregulation of protein expression and enzyme activity at a markedly slower rate, thereby mitigating drug resistance and promoting its clinical application [385]. A more fundamental challenge lies in the fact that, owing to the complexity of disease pathophysiology, current multi‐omics integration strategies often accumulate parameters without appropriate weighting under specific disease conditions. This may exacerbate the curse of dimensionality and lead to overfitting in AI models [386].

#### Multi‐omics‐driven lead screening

The screening of compound libraries for active leads offers a more efficient alternative to de novo drug design and is widely applied in the pharmaceutical industry. This approach, a precursor to modern high‐throughput screening (HTS), evolved from classical *in vitro* dose–response studies using isolated or recombinant targets, facilitating the rapid identification of candidate molecules. High‐content screening (HCS), supported by multi‐omics technologies, now represents a substantial advance beyond conventional HTS, as it enables lead screening in living cells. By integrating data encompassing pharmacodynamic actions, pathway perturbations, and cytotoxicity, multi‐omics comprehensively assesses target engagement and metabolic fate in living systems [387] to prioritize potential leads and generate rich data sets for downstream studies. Notably, owing to its swift response dynamics, the transcriptome often serves as an early indicator of compound activity, with other omics layers frequently employed in mechanistic backtracking. Nevertheless, the higher costs of HCS owing to its complex instrumentation and data‐rich nature limit its applicability, positioning it primarily for the in‐depth validation of SBDD and LBDD outputs.

Multi‐omics is also advancing virtual screening. Omics data provide robust clues for targets and ligands, overcoming their historical reliability issues [388]. AI‐accelerated models such as RosettaVS, a physics‐based virtual screening platform capable of screening multibillion‐compound libraries [389], are being increasingly deployed, expanding the scope and throughput of lead development.

#### Multi‐omics‐driven lead optimization

Prior to entering preclinical studies, leads must demonstrate optimal pharmacokinetics, safety, and efficacy through nonclinical validations conducted *in vitro* or *ex vivo*. Satisfying these criteria typically necessitates systematic optimization, an endeavor that multi‐omics technologies accelerate and refine. The outcome extends beyond the candidate molecule itself, encompassing a rich body of pharmacological profiles that inform its behavior in complex biological systems.

In pharmacokinetic assessment, multi‐omics‐based single‐cell or tissue‐level analyses surpass conventional methods in predicting the *in vivo* process of leads. Metabolomic approaches quantify drug‐metabolizing enzymes and transporters across relevant tissues to predict ADME profiles [390], offering insights for lead optimization. Moreover, although Lipinski's Rule of Five remains foundational for oral bioavailability prediction [391], the concept of druggability has been re‐examined within the multi‐omics framework. Proteolysis‐targeting chimeras (PROTACs) represent a notable exception to Lipinski's Rule of Five [392]. Membrane proteomics has revealed that specific PROTACs enter cells through CD36‐mediated endocytosis [393], a transport mechanism that is independent of passive diffusion. These findings underscore a new strategy for lead optimization.

With respect to efficacy and safety, multi‐omics offers multidimensional support. Proteomic techniques, for example, enable the assessment of target engagement, pathway regulation, and conformational profiling to evaluate lead–receptor interactions and establish dose–response relationships. A key parameter derived from such analyses is binding affinity, along with the relative binding affinity of the lead compared to endogenous ligands [394]. Moreover, omics parameters linked to normal physiology offer insights into their safety profiles, revealing potential off‐target effects or homeostasis perturbations. Beyond acute effects, multi‐omics can further reveal long‐term outcomes. A case in point is prenatal exposure to valproic acid, which has been shown to induce DNA methylation that upregulates Wnt signaling pathways in specific cells, thereby increasing the risk of autism spectrum disorder in offspring [395].

Lead optimization strategies (e.g., chemical modification) are being increasingly refined by multi‐omics: Proteomics elucidates disease‐associated conformational alterations and posttranslational modifications, suggesting potential moieties for chemical introduction; metabolomics detects pathway perturbations, thereby excluding modifications with unacceptable safety risks. Other optimization strategies are also being increasingly informed by multi‐omics. Prodrug design, for example, aims to circumvent first‐pass elimination, a pharmacokinetic challenge clarified through metabolomic studies. Chiral optimization is exemplified by l‐DOPA, an antiparkinsonian agent that crosses the blood–brain barrier with the assistance of LAT1 [396], whereas d‐DOPA results in less than 5% brain exposure because of transporter stereoselectivity [397, 398]. This classic case highlights the importance of integrating transporter proteomics into lead optimization.

A critical consideration in lead optimization is that modifications may have opposing effects, some of which may be unacceptable. To illustrate, while the introduction of a nitro group enhances the target affinity of tolcapone, it is also associated with mitochondrial toxicity, which once led to its withdrawal from the market [399]. Such scenarios necessitate iterative validation and further optimization, forming a closed loop of “design–validate–redesign.”

### Multi‐omics in preclinical studies

Preclinical studies bridge laboratory discovery and clinical translation. Conventionally, this process heavily relies on phenotypic endpoints (e.g., animal behavior and histopathology), which may provide limited insight into underlying molecular mechanisms. Multi‐omics technologies now contribute to several aspects of preclinical research, including model development, efficacy and safety evaluation, biomarker identification, and formulation optimization.

#### Multi‐omics in the development and validation of preclinical models

Successful preclinical research depends on disease models that accurately emulate human pathophysiology. Traditional model validation, which is primarily based on phenotypic similarity or a few biomarkers, often underestimates interspecies differences and modeling issues, potentially obscuring adverse events. This limitation has contributed to the clinical failure of promising drug candidates [400], such as the CDC7 inhibitor SGR‐292. Despite demonstrating efficacy and safety in a murine xenograft model of acute myeloid leukemia [401], it caused severe immunosuppression in humans, ultimately leading to its discontinuation [402].

Multi‐omics facilitates systematic model validation through pathway‐level comparisons with human disease signatures. In a recent study on Alzheimer's disease, the transcriptomic, proteomic, and metabolomic profiling of plasma and cerebrospinal fluid validated whether a type 2 diabetes macaque model recapitulates early human molecular events [403]. In oncology, proteomic comparisons of patient‐derived xenograft models with primary tumors can be used to precisely identify pathway alterations. A study of ovarian cancer revealed shifts in both the extracellular matrix and immune function, offering warnings for therapeutic development [404]. Furthermore, multi‐omics helps determine the off‐target effects of gene‐editing tools such as CRISPR, which can compromise model credibility [405]. In cases where existing models underperform, multi‐omics approaches may guide their optimization or the selection of alternatives. For instance, screening cell lines from a repository can identify those whose genetic mutations and transcriptional profiles are consistent with those of pathological samples [406].

Driven by the increasing emphasis on the 3Rs principle (replacement, reduction, and refinement) and the tightening ethical restrictions on nonhuman primate research [407, 408], alternative models, particularly organoids, are gaining importance. Nevertheless, the credibility of such models in recapitulating human pathophysiology remains to be validated. Multi‐omics addresses this by integrating single‐cell transcriptomics, proteomics, and spatial metabolomics, thereby assessing the cellular differentiation and functional maturity of organoids [409, 410]. Moreover, by combining patient‐derived organoids with real‐world data, multi‐omics can validate associations between molecular signatures identified in organoids and therapy selection. For instance, upregulation of the KRAS pathway in colorectal cancer liver metastasis may suggest sensitivity to regorafenib, an inhibitor of the downstream protein BRAF in this pathway [411]. Notably, the analytical power of multi‐omics directly aligns with the 3Rs principle by replacing traditional large‐scale studies with data‐rich analyses on fewer animals.

#### Multi‐omics‐driven preclinical evaluation and biomarker identification

Preclinical efficacy and safety evaluation constitute the key determinants of whether a drug candidate advances to clinical trials. Conventional strategies, constrained by a narrow panel of endpoints, frequently yield incomplete assessments. Multi‐omics overcomes this by enabling integrated, multi‐timepoint analysis of molecular changes associated with drug response, thereby supporting biomarker discovery and informing early benefit‐risk assessment.

Preclinical evaluation must confirm the intended pharmacological effects and validate safety. Spatial multi‐omics enables the in situ profiling of drug effects on specific cellular subsets, thereby providing a more precise representation of drug activity in complex tissues, such as when evaluating drug efficacy within the tumor microenvironment [412]. Single‐cell multi‐omics further extends efficacy evaluation down to the level of cellular subsets. When applied to cancer immunotherapy, this technique can precisely identify the T‐cell subsets activated by a given drug, revealing their distinct roles in the therapeutic response [413].

In toxicological evaluation, multi‐omics, particularly metabolomics, excels through the sensitive detection of endogenous molecular perturbations, thereby facilitating the identification of toxicity biomarkers. This enables the early analysis of toxicity signatures against reference databases of known toxicants, facilitating predictive toxicology. Tissue damage that is difficult to detect by conventional methods may still result in characteristic metabolic alterations in biofluids. The hepatotoxic impact of *Rhododendron molle* Flos on reactive oxygen species levels serves as an example [414]. The integration of multi‐omics data further provides molecular evidence for determining the no‐observed‐adverse‐effect level. For instance, even in the absence of histopathological abnormalities, the upregulation of key apoptotic or inflammatory pathways indicates early toxicity, providing a foundation for the safe starting dose in first‐in‐human studies. Notably, gut microbiome analysis has emerged as a novel dimension in toxicological assessments, capturing the impact of drug–microbiota interactions on drug efficacy and toxicity [415].

Beyond biomarker identification, multi‐omics enables cross‐validation across biological layers. The reliability of a metabolite as a biomarker, for example, is substantially strengthened when its alterations align with transcriptomic and proteomic changes. Furthermore, projecting differentially abundant molecules onto shared biological pathways may facilitate the identification of broadly applicable biomarkers whose generalizability can be evaluated using public databases [416]. Moreover, cross‐species multi‐omics comparisons may yield evolutionarily conserved biomarkers that reflect model fidelity, such as the adrenal gland genes *CYP11B1* and *CYP11B2* in mice and humans [417]. A study on Crohn's disease exemplifies this multidimensional paradigm. In this study, *S100A8* and *IGFBP5* were identified as key risk genes from transcriptomic databases using machine‐learning algorithms. Their diagnostic value was subsequently validated in patient blood samples, and their therapeutic relevance was confirmed in animal models [418], establishing a complete route from multi‐omics findings to preclinical proof‐of‐concept. In addition, epigenomics offers a distinctive perspective at this stage. Certain drugs act via epigenetic modifiers, inducing epigenetic modifications that precede transcriptional or proteomic alterations. These patterns may serve as both mechanistic evidence and activity biomarkers. Ivosidenib, an IDH1‐targeting antitumor agent that reduces the level of the epigenetic modifier 2‐hydroxyglutarate, illustrates this case [419]. Nevertheless, the relationship between candidate biomarkers and true clinical efficacy requires rigorous validation.

#### Multi‐omics in pharmaceutical formulation

Pharmaceutical formulation is a critical step in translating active pharmaceutical ingredients into usable dosage forms. Multi‐omics technologies offer a molecular perspective on formulation development by systematically assessing how formulations shape drug fate *in vivo*. Transcriptomics, proteomics, and metabolomics detect aberrant perturbations triggered by dosage forms, guiding formulation optimization and quality control, a strategy that is particularly relevant for gene therapy products [420]. For rapidly advancing nanoformulations, proteomic analysis can elucidate how preparations affect interactions with plasma and membrane proteins, as well as the formation of the protein corona, a key determinant of the *in vivo* fate of the formulation [421]. In cases in which a formulation (e.g., nanoparticles) elicits immune responses, transcriptomics and proteomics can profile immune pathway activation, facilitating safety assessment.

### Multi‐omics‐driven clinical evaluation

Building upon preclinical assessment, multi‐omics approaches not only facilitate drug development and repurposing but also reshape the entire clinical evaluation continuum, including patient stratification, clinical trials, and postmarketing re‐evaluations.

#### Multi‐omics‐driven patient stratification

Patient stratification constitutes a core application of multi‐omics in precision medicine. Conventional strategies, which rely primarily on isolated biomarkers or clinicopathological features, may compromise clinical outcomes in diseases with high heterogeneity [422]. Multi‐omics technologies provide a molecular lens for patient stratification, identifying target patient populations and guiding precision medicine through mechanistic insights, biomarkers, and pathway networks. Furthermore, these approaches furnish the rationale for employing enrichment strategies in clinical trials [423].

The essence of multi‐omics‐driven patient stratification lies in distilling common molecular patterns from massive heterogeneous data sets to define clinically relevant subgroups. Non‐negative matrix factorization has emerged as a prevailing algorithm in this context. It projects high‐dimensional multi‐omics data (e.g., gene expression and protein abundance) into a lower‐dimensional space, thereby capturing core molecular signatures that constitute subtype classifications [424]. An alternative strategy, similarity network fusion, involves constructing patient similarity networks for each omics layer individually before integrating them for noise‐robust classification [229, 425], while gene set variation analysis enhances interpretability by converting omics data into pathway activity scores [426], thereby linking results to disease pathophysiology. AI‐powered approaches, such as Subtype‐GAN [427], are also flourishing. Critically, there is no universal patient stratification strategy. The selection of appropriate methods depends on the specific disease and patient characteristics. Furthermore, cross‐validation across multiple approaches can offset single‐algorithm bias. A recent study comparing 12 survival analysis models confirmed that compared with any single model, multimodel integration achieves significantly higher predictive reliability [428]. However, translating these complex outputs into clinically accessible tools remains an essential challenge.

Interindividual variability in drug metabolism and toxicity response represents another core consideration in patient stratification. Multi‐omics enables the analysis of polymorphisms in drug‐metabolizing enzymes and transporters, thereby advancing precision medicine. In addition, drug metabolism is shaped by more than patient genetics and expression variability; other factors, such as the gut microbiota [415], also play a role.

#### Multi‐omics in clinical trials

Clinical practice is the definitive phase for evaluating the clinical benefit of therapeutics. Conventional clinical trials focus on phenotypic endpoints and population‐level average effects, with gold standards such as the survival rate providing reliable evidence for drug approval [429]. Nevertheless, this approach offers limited insight into the molecular mechanisms that govern drug response and may fail to explain interpatient variability [430]. Multi‐omics is now expanding clinical trials from endpoint confirmation to mechanistic resolution.

Phase 0 trials aim to assess the pharmacokinetic consistency between humans and preclinical models using microdose therapy [431]. Multi‐omics has facilitated their realization. Metabolomics, supported by platforms such as HPLC–MS, enables the comprehensive detection of drugs and metabolites [432], facilitating the comparison of human metabolic profiles with animal data. Proteomics can characterize drug–plasma protein binding, thereby providing insights into bioavailability [433].

In phase Ⅰ trials, interindividual pharmacokinetic variability directly influences the reliability of safety assessment and subsequent dose determination, particularly the maximum tolerated dose (MTD). However, the traditional “3 + 3” dose‐escalation schema may yield imprecise MTD estimates because of its inherent methodological limitations [434]. Multi‐omics offers a solution by comprehensively analyzing genetic polymorphisms and protein expression in participants prior to dose escalation, mitigating the risk of severe adverse events. For instance, individuals carrying CYP3A5*3 exhibit poor metabolism of sorafenib, which may lead to hepatotoxicity at high doses [435], whereas the methylation of *SLCO1B3* can limit drug uptake in certain patients [436]. An unstratified MTD thus risks adverse events or suboptimal efficacy. The biologically effective dose (BED), which is based on downstream pathway alterations, has emerged as an alternative to MTD in the era of targeted therapy. This is illustrated by veliparib, a PARP inhibitor that significantly suppresses PAR at doses substantially below its MTD, achieving target engagement without dose‐limiting toxicity [437]. While this strategy mitigates the risks inherent in pursuing MTD, the lack of standardized procedures for BED determination currently constrains its role to supportive evidence in regulatory review.

In terms of efficacy validation, integrating multi‐omics with patient stratification transforms the clinical evaluation framework from a binary judgment to mechanistic elucidation. Through multi‐timepoint analysis of serial biological samples, multi‐omics maps the molecular trajectory of the therapeutic response, capturing target engagement, pathway activation, and metabolic perturbations. This approach not only validates the intended efficacy and mechanism of action but also enables the early detection of drug resistance or long‐term toxicity. ctDNA sequencing, for example, may identify acquired resistance mutations months before radiographic progression or increases in traditional biomarkers, as demonstrated in a study on prostate cancer [438]. Multi‐omics technologies are also advancing research on surrogate endpoints, particularly those causally linked to long‐term clinical outcomes, thereby accelerating regulatory approval. A representative case is lurbinectedin, which received accelerated approval for metastatic small cell lung cancer on the basis of surrogate endpoints, including the inhibition of oncogene transcription, induction of apoptosis, and duration of response [439]. Nevertheless, surrogate endpoints require rigorous causal validation. An analysis of FDA approvals from 2013 to 2017 revealed that only 43% of cancer therapies granted accelerated approval based on surrogate endpoints that demonstrated clinical benefit in confirmatory trials [440].

Safety is a critical determinant in clinical evaluation, often carrying importance comparable to, or even surpassing, that of efficacy. Conventional safety assessment frequently relies on detecting, quantifying, and analyzing adverse events in clinical trials, which may introduce bias. Multi‐omics technologies are shifting clinical toxicology assessments toward individual prediction and mechanistic elucidation. By systematically comparing paired samples collected before and after drug administration, omics profiles enable the precise detection of toxicity signatures. Metabolomics, for instance, can identify characteristic metabolic perturbations, such as alterations in bile acid profiles and early signals of hepatotoxicity [441]. Transcriptomic and proteomic approaches can also reveal early stress or inflammatory pathway activation prior to histopathological manifestations of liver injury [442]. While such molecular‐level toxicity assessment offers a more sensitive safety margin for clinical trials, this heightened sensitivity may also lead to false positives, necessitating rigorous data interpretation. When adverse events arise, multi‐omics can provide in‐depth elucidation of their molecular mechanisms, thereby effectively safeguarding trial participants and informing further investigations. As a case in point, a clinical trial participant developed a severe bleeding tendency following the administration of a PD‐1 inhibitor [443]. Multi‐omics approaches, particularly transcriptomics and proteomics, identified the underlying cause as immune thrombocytopenia resulting from aberrant B‐cell activation coupled with PI3K/Akt pathway suppression. Targeted interventions based on these findings promptly resolved the crisis [443].

#### Multi‐omics in postmarketing surveillance and drug repurposing

Drug approval signals the beginning of real‐world evidence generation, not the end of clinical evaluation. Multi‐omics is playing an increasingly pivotal role in postmarketing surveillance and drug repurposing, transforming the postapproval phase into an ongoing optimization process—a self‐improving framework from bench to bedside and back to the bench for further development.

Following approval, drugs are administered to broader and more diverse populations, including special groups typically excluded from clinical trials. Multi‐omics enables the identification of molecular signatures associated with rare responses or adverse events, refining the pharmacological profile and further substantiating the drug's value [444]. By integrating multi‐omics data with patient‐reported outcomes, investigators may identify core molecular and pathway determinants of quality of life, factors that can be elusive in clinical trials. Such insights can, in turn, guide biomarker refinement and drug development.

In a study of glioblastoma, patients with comparable survival durations exhibited divergent quality‐of‐life outcomes. Multi‐omics linked this variation to mutations and expression levels of non‐survival‐related genes such as *ERBB2* [445]. Multi‐omics is also advancing pharmacovigilance and pharmacoepidemiology, surpassing conventional reliance on spontaneous reports and surveys given their inherent sample and causality limitations. Clinical multi‐omics studies, especially those coupled with electronic health records, enable active postapproval surveillance, uncovering associations between drugs and adverse events. A study based on genomic and transcriptomic evidence, for instance, revealed that lansoprazole may induce or exacerbate juvenile idiopathic arthritis [446], potentially supporting label revision. Nevertheless, clinical evidence faces inherent limitations, including data missingness, sampling bias, and the nonquantitative nature of general medical records, necessitating the use of sophisticated algorithms for signal detection [447]. Multi‐omics further addresses the challenge of polypharmacy, a common scenario in clinical practice that frequently remains underexplored in clinical trials. For instance, the SyndrumNET model evaluates the risks of drug–drug interactions by analyzing global perturbations in metabolic and signaling pathways [448].

Repurposing approved drugs may reduce the cost of drug development. Multi‐omics is elevating this process from a matter of chance to systematic exploration through the identification of negative correlations between disease omics profiles and drug perturbation signatures. In a recent study integrating public omics data with molecular docking, chloramphenicol was identified as a potential candidate for BTK‐related cancers [449]. Drug repurposing also extends to agents that have ‘failed’ in initial development. A case in point is gefitinib, which initially received accelerated approval but was subsequently withdrawn after failure to prolong overall survival in a phase Ⅲ clinical trial. Supported by subsequent omics analysis and targeted clinical trials, it has become an established therapy for *EGFR*‐mutant non‐small cell lung cancer [450]. For effective drugs with severe toxicity, formulation strategies are worth pursuing. Optimization of the dosing regimen, supported by metabolomic studies, led to the reapproval of Mylotarg, an antibody–drug conjugate that was initially withdrawn because of fatal hepatotoxicity [451].

### Section summary

At the macro level, multi‐omics is catalyzing a paradigm shift in pharmaceutical life‐cycle management. By furnishing a high‐resolution, multidimensional view of biological systems, multi‐omics approaches weave an intricate molecular thread throughout the entire drug development pipeline. As technological standardization advances, bioinformatics matures, and real‐world data accumulate, and multi‐omics‐driven drug development is poised to evolve from a cutting‐edge pursuit into mainstream practice. This transformation promises to yield more efficient and reliable solutions for precision medicine, ultimately bridging the gap between scientific progress and therapeutic translation.

## MULTI‐OMICS IN PRECISION ONCOLOGY

**Figure 6:**
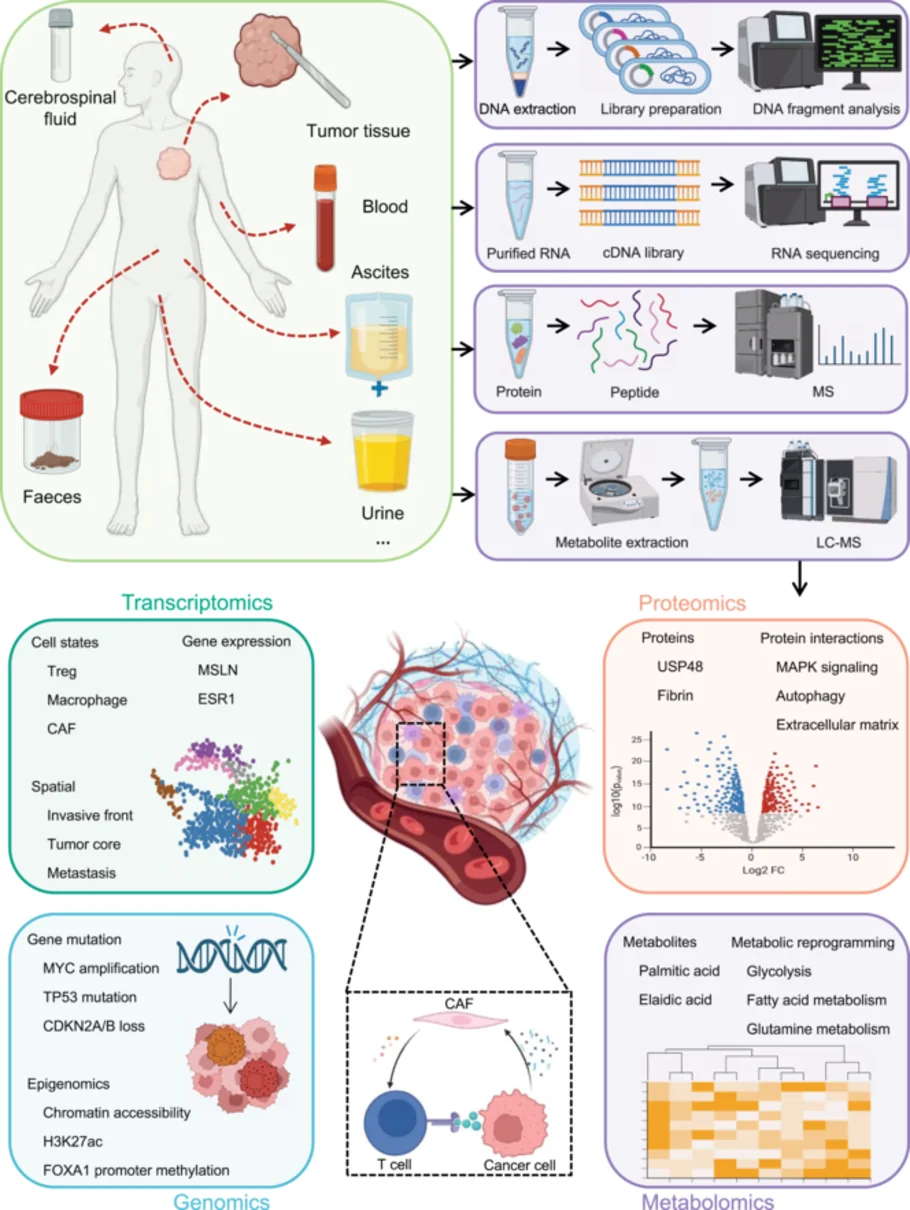
Multi‐omics approaches for elucidating tumor biology. Tumor tissues and body fluids—such as blood, cerebrospinal fluid, urine, and feces—can be analyzed through tissue and liquid biopsies using multi‐omics platforms that integrate genomics, transcriptomics, proteomics, and metabolomics to comprehensively elucidate the molecular mechanisms underlying tumor initiation and progression.

In recent years, the rapid advancement of multi‐omics technologies (including genomics, epigenomics, transcriptomics, proteomics, and metabolomics) has revealed not only the molecular mechanisms underlying tumorigenesis and progression but also tumor heterogeneity. Tissue samples are obtained via tissue biopsy, while body fluids such as blood, effusions, cerebrospinal fluid, and urine are collected through liquid biopsy. These samples are analyzed across multiple platforms to elucidate tumor biology at the genetic, transcriptional, protein, and metabolite levels (Figure 6).

### Multi‐omics approaches reveal the biological mechanisms of cancers

#### Genomic architecture and clonal evolution

Cancer is characterized by dynamic evolution, in which genomic instability generates genetic diversity under selective pressure. The continuous acquisition of new driver mutations further drives genetic heterogeneity across tumor regions and among subtypes [452]. Multi‐omics technologies have enabled comprehensive analysis of the genomic architecture and clonal evolution of cancer, facilitating a shift toward personalized and genotype‐guided treatment approaches.

The advent of genomics and epigenomics has provided crucial insights into tumor heterogeneity, as genomic characteristics vary across tumor sites. A study integrating whole‐genome and DNA methylation sequencing of 570 melanoma samples revealed distinct genomic characteristics across melanoma subtypes. Uveal melanoma exhibited the lowest tumor mutational burden, whereas cutaneous melanoma demonstrated the highest tumor mutational burden. Mucosal and acral lentiginous melanomas frequently harbor focal amplifications of oncogenes, including *CCND1*, *CDK4*, *MDM2*, and *TERT*. These distinct genomic profiles profoundly influence treatment decisions for patients with different melanoma subtypes [453]. In colorectal cancer (CRC), metastatic sites exhibit distinct mitochondrial mutations and copy number variations (CNVs); lymph node and primary tumor core cells harbor chromosome 21 amplification without MT‐RNR2 mutations, whereas liver metastases share CNV patterns with primary tumor margins and exhibit heterozygous MT‐RNR2 mutations [454]. Next‐generation sequencing data from 5526 colorectal cancer samples revealed that patients with brain metastases typically harbor more *KRAS*, *BRAF*, and *SMAD4* mutations. Tumors at brain metastasis sites generally exhibit a high mutation burden, characterized by frequent *TP53*, *SMAD4*, and *MYC* mutations [455]. Integrative ChIP‐seq, ATAC‐seq, and transcriptomic analysis of gastric cancer revealed subtype‐specific enhancers in mesenchymal (Mes‐GC) and nonmesenchymal types. Mes‐GC shows increased chromatin accessibility, with TEAD1 acting as a master transcription factor, and is selectively sensitive to TEAD1 inhibition [456]. Moreover, within the same patient, DNA methylation levels are higher in poorly differentiated gastric cancer cells than in their well‑differentiated counterparts within the primary tumor. Hypermethylated promoters of *TMEM240* and *HAGLROS*, as well as hypomethylated promoters of *TRPM2‐AS* and *HRH1*, represent candidate methylation biomarkers for gastric cancer [457]. ER‐positive HER2‐negative breast cancer is characterized by high frequencies of *TP53* mutations and *MYC* amplification, with greater heterogeneity in the methylation patterns of the *FOXA1* promoter region [458]. Patients with lung cancer brain metastases frequently exhibit deletions in the 15q15 chromosomal region, including *MGA*, which encodes a *MYC* signaling inhibitor. These findings suggest that abnormal *MYC* signaling promotes the occurrence of brain metastases [459]. Whole‐genome sequencing of 378 metastatic prostate cancers revealed distinct mutation frequencies in androgen receptor binding sites across metastatic sites; liver/visceral metastases had higher TMB than bone/lymph node metastases did, which correlated with organ‐specific DNA mismatch repair deficiencies [460].

#### Tumor microenvironment and cell states

The tumor microenvironment (TME) is a highly complex system comprising tumor cells, infiltrating immune cells (e.g., macrophages, dendritic cells, and lymphocytes), and stromal cells such as cancer‑associated fibroblasts, endothelial cells, and adipocytes, together with the extracellular matrix (ECM) and various signaling molecules. In recent years, the TME has garnered increasing attention because of its critical role in tumor immunosuppression, tumor metastasis, and responses to targeted therapies [461]. Advances in multi‐omics technologies have enabled single‑cell resolution identification of the complex cellular composition of tumor tissues, including malignant cells, stromal cells, and immune cells, thereby greatly enriching our understanding of tumor heterogeneity.

Integrative multi‐omics approaches provide valuable insights into the tumor microenvironment and cell states. The single‑cell transcriptomic profiling of metastatic colorectal cancer revealed *MSLN* as a tumor marker associated with tumor budding at the invasive front, whereas tumor core regions exhibited increased stemness [462]. Breast cancer cells exhibit significant heterogeneity across age groups, with estrogen receptor‑positive tumors showing increased *ESR1* expression with advancing age [463]. Hepatocellular carcinoma comprises three tumor cell subtypes: the *ARG1* metabolism subtype (Metab‑subtype), the *TOP2A* proliferation phenotype (Prol‑phenotype), and the *S100A6* prometastatic subtype [464].

In addition to tumor cells, other cells within the tumor microenvironment also exhibit heterogeneity. Analysis of 1670 colorectal cancer samples (single‐cell and spatial transcriptomics) revealed four immune phenotypes (immune desert, B‐cell enriched, T‐cell enriched, and myeloid‐enriched) and an IL‐1‐mediated neutrophil‐fibroblast niche [465]. Single‐cell transcriptomic analysis of primary tumors, metastatic sites, and circulating tumor cells from multiple breast cancer patients revealed the *CBX3*‐*SNX10*‐*ANO6* signaling axis. This axis promotes the generation of fusion cells between tumor cells and lipid‐associated macrophages, thereby enhancing metastasis. These cells exhibit high sensitivity to simvastatin treatment [466]. In pancreatic cancer, extensive tertiary lymphoid structures surround uninvaded nerves, while invaded nerves are enveloped by NLRP3^+^ macrophages and cancer‐associated myofibroblasts [467].

The heterogeneity of the tumor microenvironment is closely associated with the response to immunotherapy or chemotherapy. For example, among lung cancer patients receiving immunotherapy, those with a major pathological response (MPR) presented elevated levels of FGFBP2, NK/NK‐like T cells, memory B cells, and effector T cells, whereas non‐MPR patients presented higher levels of CCR8^+^ Tregs [468]. A chemotherapy‑resistant niche in pancreatic cancer consists of SNCG basal‐like tumor cells, SPP1 tumor‐associated macrophages, and exhausted T cells [469]. THBS2^+^ matrix cancer‐associated fibroblasts are closely associated with immunotherapy resistance in gastric cancer [470].

Therefore, the advancement of multi‐omics approaches has not only deepened our understanding of tumor heterogeneity and the tumor microenvironment but also established a link between this heterogeneity and treatment response.

#### Proteomic/metabolic rewiring and therapeutic vulnerabilities

Proteins in tumors frequently undergo reprogramming in terms of their composition, structure, expression, and modification status, thereby altering protein signaling network equilibria to increase tumor cell survival, proliferation, and invasion. Furthermore, metabolic pathways in tumor cells often undergo reprogramming, simultaneously affecting the host's systemic metabolism. Proteomics and metabolomics provide critical insights into these rewiring processes and facilitate the identification of therapeutic targets and biomarkers.

Proteins serve as functional executors driving tumorigenesis and progression, particularly in the context of molecular signal transduction. Proteomic analysis revealed that overexpression of ubiquitin‐specific protease (USP48) in CRC is closely associated with autophagy inhibition. Further studies revealed that USP48 interacts with SQSTM1, inducing ubiquitination and thereby suppressing autophagy [471]. Another proteomics study using liquid chromatography–tandem mass spectrometry on mouse tumors revealed that fibrin depletion increases matrix protein expression, promotes extracellular matrix remodeling within the tumor microenvironment, and inhibits pancreatic cancer cell proliferation [472]. In bladder cancer, proteomics revealed that neutrophil extracellular traps promote tumor immune evasion by upregulating *STC1* through the MAPK–FosL1 pathway [473].

Proteomics has also facilitated the discovery of noninvasive biomarkers of tumor progression. For instance, a proteomic analysis of plasma exosomes from non‐small cell lung cancer patients revealed that *CD147* expression progressively increased with tumor stage. Subsequent studies demonstrated that *CD147*‐driven exosomes significantly increased the proliferation and metastatic capacity of lung cancer cells, identifying a promising therapeutic target [474]. Through *in vivo* proteomic labeling, another study revealed that lactate dehydrogenase A‐like 6A (LDHAL6A) is a persistent exosomal effector that promotes malignant cellular processes. The inhibition of LDHAL6A in combination with paclitaxel treatment significantly suppressed the growth and metastasis of triple‐negative breast cancer. These findings indicate that LDHAL6A serves not only as a noninvasive biomarker for triple‐negative breast cancer but also as a highly promising therapeutic target [475]. Proteomics advances research on tumor heterogeneity. In lung squamous cell carcinoma, low‐keratinization patients showed stronger immune infiltration, whereas high‐keratinization patients exhibited enhanced stemness, activated metabolism, and immune suppression [476]. A spatial proteomics study of CRC revealed the co‐localization of C1QC‐resident tissue macrophages with CD4 T cells in immunotherapy responders, whereas cancer‐associated fibroblasts suppressed this interaction in nonresponders [477]. In hepatocellular carcinoma, tumor cells surrounded by a fibrotic ring (FR) exhibit elevated levels of basement membrane proteins and fibroblast lineage markers (COL4A1, FBLN5, DCN, and DPT). In contrast, the tumor stroma of FR‐ tumors displays increased levels of immunoregulatory and ECM degradation proteins (IL‐18, LGALS9, ISG20, and CTSB) [478].

Metabolomics plays a crucial role in elucidating metabolic reprogramming during tumor progression and in identifying biomarkers and therapeutic targets. For instance, a metabolomics analysis of 34 non‐small cell lung cancer patients with or without brain metastases revealed elevated palmitic acid levels in the metastatic niche as a key driver of brain metastasis. The fatty acid synthase inhibitor TVB‐2640 was identified as a potential therapeutic agent that disrupts metabolic vulnerability and inhibits brain metastasis in non‐small cell lung cancer [479]. Metabolomic analysis of plasma from hepatocellular carcinoma patients revealed a significant decrease in elaidic acid levels. Subsequent metabolomic studies in mice further demonstrated that elaidic acid promotes spermidine production by intestinal *Lactobacillus murinus*, thereby inhibiting hepatocellular carcinoma growth [480]. Additionally, metabolomics revealed that de novo guanine nucleotide biosynthesis is vulnerable in *KRAS*‐mutated pancreatic ductal adenocarcinoma, with *IMPDH2* serving as a key target. The inhibition of *IMPDH2* induces irreversible guanine nucleotide depletion and increases the efficacy of KRAS inhibitors both *in vitro* and *in vivo* [481]. In colorectal cancer, metabolomics revealed that the key tumor‐associated bacterium *Prevotella copri* consumes glycerophosphocholine to reprogram MARCO^+^ macrophages, creating an immunosuppressive niche. Supplementation with glycerophosphocholine is considered a promising immunometabolic intervention strategy [482].

Metabolomics provides a comprehensive understanding of tumor‐specific metabolic profiles, elucidating the metabolic heterogeneity of tumors. For example, patients with pancreatic ductal adenocarcinoma exhibit heterogeneous glycolytic activity, with low glycolytic activity associated with better clinical outcomes and treatment response. Metabolic inhibitors targeting lactate dehydrogenase A demonstrate differential sensitivity, confirming the potential therapeutic value of glycolysis‐targeting therapies [483]. Spatial metabolomics further revealed that human hepatocellular carcinoma is characterized by the presence of metabolically heterogeneous subregions. Within tumor regions, glutamine metabolism serves as a unique metabolic signature of Treg‐enriched subregions; conversely, in normal regions, this metabolic feature shows no specific correlation with Treg distribution [484]. Another study compared the clinical heterogeneity between left‐sided and right‐sided colorectal cancers. Metabolomic analysis of fecal samples revealed that right‑sided colorectal cancer patients accumulated L‑phenylalanine, whereas left‑sided patients accumulated D‑ornithine and L‑citrulline [485]. Collectively, metabolomics provides valuable insights into tumor‑specific metabolic signatures, offering critical information for cancer diagnosis, prognosis, and the development of novel therapeutic targets.

### Applications of multi‐omics approaches in precision cancer therapy

**Figure 7:**
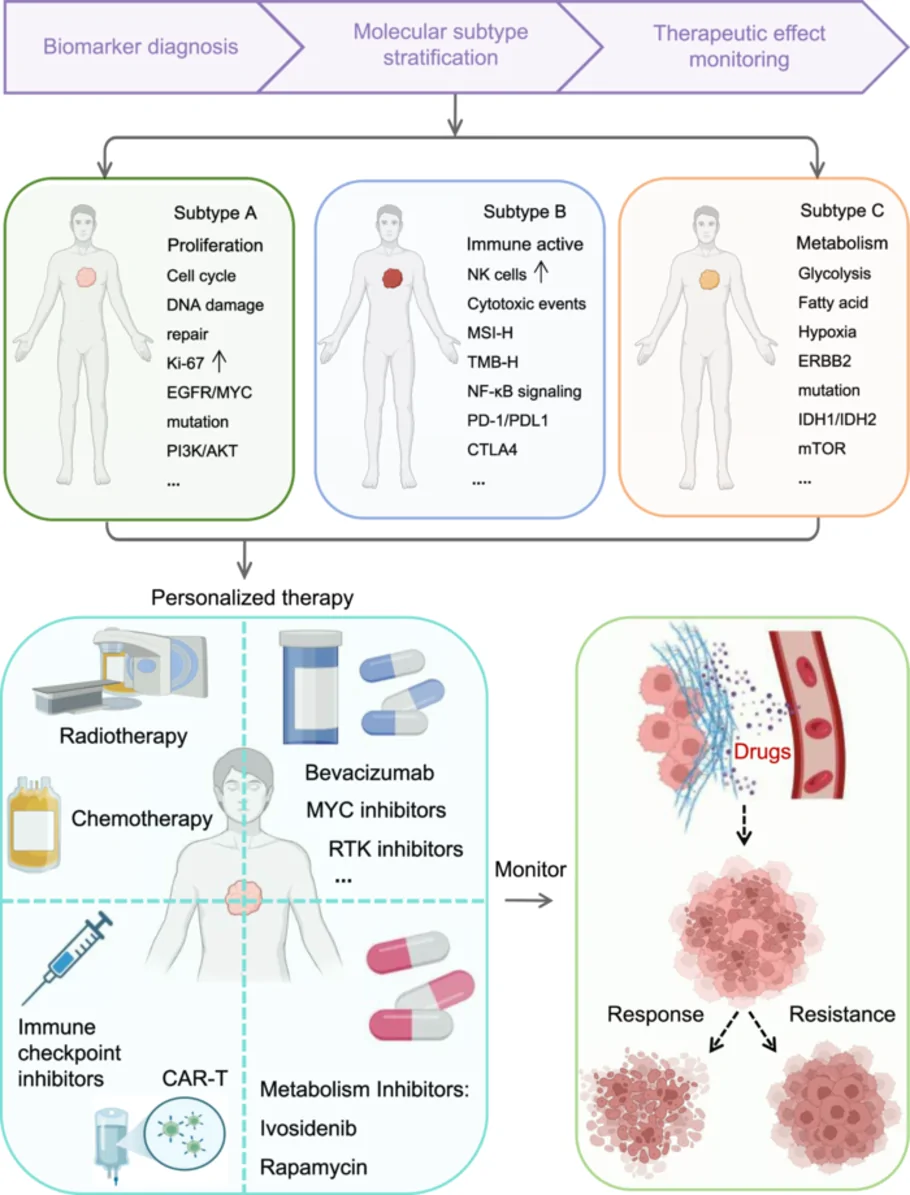
Role of integrated multi‐omics data analysis in precision oncology. Multi‐omics analysis has three primary applications in precision cancer therapy: biomarker‐based cancer diagnosis, molecular subtyping of tumors, and prediction and monitoring of therapeutic efficacy. Integrated multi‐omics data analysis can effectively identify biomarkers for cancer diagnosis and risk prediction, enable precise patient stratification into molecular subtypes, and facilitate the selection of optimal treatment strategies. In this schematic, representative molecular subtypes include proliferation‐dominant subtype A, angiogenesis‐dominant subtype B, immune‐active subtype C, and metabolism‐dominant subtype D. Multi‐omics analysis also supports monitoring of patient treatment response and tumor evolution.

Precision oncology is among the most mature applications of MODPM. By integrating tumor genomics, transcriptomics, epigenomics, proteomics, metabolomics, single‐cell profiles, spatial information, and clinical data, MODPM can improve cancer diagnosis, risk prediction, molecular subtyping, and personalized treatment design (Figure 7).

#### Diagnosis and risk prediction of tumors

The rapid advancement of multi‐omics technologies has enabled in‐depth exploration of tumorigenesis and tumor progression. In the era of precision medicine, integrated multi‑omics analysis provides biomarkers for both tumor diagnosis and actionable therapeutic targets. For example, a cohort study of 251 participants developed a machine‐ learning model by integrating proteomics and metabolomics data. This model included four proteins (LRG1, ITIH3, PDIA4, and PON1) and three metabolites (kynurenine, indole, and 3‐hydroxybutyrate) and effectively distinguished epithelial ovarian cancer patients from healthy controls, with an AUC of 0.975 [486]. Another study integrated transcriptomic, proteomic, and metabolomic data to establish a robust collagen‑based machine‐learning model for identifying clinically significant prostate cancer, which demonstrated superior performance to that of PSA and age [487]. Furthermore, the integration of genomic and transcriptomic data from blood samples of gastrointestinal tumor patients enabled the detection of genetic mutations in cell‑free DNA and RNA, with the detection efficacy significantly outperforming single‑omics analysis. Mutations in nine genes, including *TP53* and *SMAD2*, were detected in more than 75% of patients [488].

The increasing availability of multi‑omics data sets enables better characterization of tumor heterogeneity, facilitating risk prediction and prognosis assessment. One study developed a machine learning‐based model, CIMPTGV, to predict recurrence risk in HR + /HER2− breast cancer by integrating metabolomics, transcriptomics, and genomics data from 579 patients. The model achieved a concordance index of 0.871 on the training set and 0.869 on the test set [489]. Another multi‐omics integrated analysis encompassing proteomics, genomics, and transcriptomics data from 154 gastric cancer patients revealed distinct molecular subtypes. The constructed decision tree model optimized the prediction of recurrence and survival after staging [490]. Additionally, a study screened proteomic and transcriptomic data from intrahepatic cholangiocarcinoma (ICC) to develop a novel framework integrating Cox regression analysis with five machine‐learning algorithms. Compared with the traditional serum prognostic marker CA19‐9, the resulting risk score demonstrated superior prognostic performance [491]. Genomic, epigenomic, and transcriptomic sequencing of 220 patients with metastatic prostate cancer revealed cell‑free androgen receptor/enhancer alterations as prognostic biomarkers in lethal metastatic castration‑resistant prostate cancer. These alterations are associated with significantly poorer progression‑free survival [492].

#### Molecular subtypes of tumors

The integration of multi‐omics data plays a critical role in identifying molecular subtypes of tumors, enabling the development of subtype‑specific personalized treatment strategies to improve therapeutic outcomes.

In cholangiocarcinoma, an integrative analysis of genomic and transcriptomic data revealed four intrinsic molecular subtypes: the cholangiocellular‑like subtype (high expression of normal cholangiocyte epithelial genes), the stromal subtype (enrichment of endothelial and fibroblasts), the metabolic subtype (upregulation of bile acid metabolism pathways), and the invasive inflammatory‑proliferative subtype (activation of the cell cycle and inflammatory pathways). The cholangiocellular‐like subtype demonstrated the best survival outcomes with nab‐paclitaxel plus gemcitabine–cisplatin therapy, whereas the aggressive inflammatory‐proliferative subtype, which has the poorest prognosis, may benefit from a treatment strategy combining PD‑1 inhibitors with gemcitabine/cisplatin and low‑dose nab‑paclitaxel [493]. An integrated analysis of genomic, transcriptomic, proteomic, and metabolomic data from gallbladder cancer revealed four multi‐omics subtypes (MO1–MO4). MO1 may benefit from combination chemotherapy with cell cycle inhibitors or immunomodulators because of its immune‐activated phenotype. The activation of oncogenic signaling pathways in MO2 indicates sensitivity to RTK inhibitors. MO3 can be targeted for its metabolic dependency using metabolic inhibitors or MYC inhibitors. The immunosuppressive phenotype of MO4 suggests the potential efficacy of ErbB2‐targeted therapy combined with immune checkpoint inhibitors or immunomodulators [494]. Additionally, a multi‐omics map of glioblastoma encompassing genomic, transcriptomic, and proteomic data revealed five distinct molecular subtypes (neural‐like, tumor‐driving, premature tumor evolution, hot tumor, and classical), and the omics characteristics and therapeutic strategies for each subtype were systematically elucidated [495]. A multi‐omics study integrating transcriptomics, metabolomics, and proteomics analyzed tumors and serum from 41 hepatocellular carcinoma patients. Three subtypes of fatty acid degradation (FAD) have been identified: Subtype F1 is characterized by low FAD activity, an immunosuppressive microenvironment, and responsiveness to sorafenib treatment. Subtype F2 occupies an intermediate position and represents a potential beneficiary of atezolizumab plus bevacizumab (T + A) therapy. Subtype F3 demonstrates high FAD activity and sensitivity to transarterial chemoembolization (TACE) [496]. An integrative multi‐omics analysis of genomics, transcriptomics, proteomics, and metabolomics classified HER2‑negative breast cancer into three subtypes. Subtype 1 is characterized by active estrogen signaling pathways and is sensitive to endocrine therapies such as tamoxifen. Subtype 2 is characterized by high angiogenesis and may benefit from antiangiogenic treatments such as bevacizumab. Subtype 3 is characterized by high proliferation, resembling that of HER2‐positive breast cancer, and is likely to benefit from anti‐HER2 therapies such as trastuzumab [497]. Additionally, a multi‐omics analysis of proteomics, genomics, and transcriptomics data from 112 small cell lung cancer patients revealed four molecular subtypes and elucidated the multi‑omics characteristics of each. Subtype 1 is associated with high proliferation and E2F activity and strongly responds to ATR and TOP1 inhibition. Subtype 2 is characterized by high DLL3 expression and may benefit from DLL3‐targeted therapy. Subtype 3 has elevated RNK signaling activity and is sensitive to multitargeted tyrosine kinase inhibitors such as anlotinib. Subtype 4 has high *MYC* expression, suggesting the potential for targeting AURKA/B [498].

In summary, the complexity of tumors means that single‐omics data cannot fully capture their molecular characteristics. Integrating multi‐omics data significantly improves the accuracy of subtype classification. By assigning patients to subtypes with distinct molecular profiles, clinicians can more accurately predict disease progression and responses to specific drugs, thereby developing more targeted treatment strategies.

#### Prediction and evaluation of tumor treatment efficacy

Multi‐omics‐based predictive models can integrate somatic mutations, copy number alterations, transcriptomic programs, epigenetic states, proteomic signaling, immune cell composition, tumor microenvironment features, and clinical variables to predict the response to immunotherapy, targeted therapy, chemotherapy, or combination regimens. For example, Xiao et al. analyzed proteomic and transcriptomic data from plasma samples of triple‐negative breast cancer patients and reported that the plasma levels of ARG1, NOS3, and CD28 are significantly associated with the response to neoadjuvant immunotherapy. The plasma‐based immunotherapy predictive scoring model they developed demonstrated effective predictive capability for immunotherapy response [499]. scRNA‐seq‐based frameworks such as scCURE can identify cell types associated with immunotherapy response and integrate mechanism analysis with outcome prediction [500]. Additionally, metagenomic and metabolomic analyses of plasma and fecal samples from 44 non‐small cell lung cancer patients revealed that a model incorporating differentially abundant microbial species (*Clostridium* sp. M62/1 and *Eisenbergiella tayi*) and metabolic features (linoleic acid, oxindole‐3‐acetic acid, and quinolinic acid) effectively predicted the response to neoadjuvant immunotherapy, with AUCs > 0.85 [501].

The integrated analysis of multi‐omics data facilitates dynamic monitoring during tumor treatment, providing a scientific basis for adjusting treatment plans in real time and enabling personalized precision medicine. For example, Wang et al. analyzed the proteomics and single‐cell spatial transcriptomics of urine and tumor biopsy samples from 97 patients with biliary tract cancer and developed a machine‐learning model based on four urinary proteins to predict and monitor responsiveness to immune checkpoint inhibitors [502]. Zheng et al. conducted a combined metabolomic, lipidomic, and proteomic analysis of serum samples from small cell lung cancer patients receiving immunotherapy in combination with chemotherapy. They developed and validated a noninvasive, dynamic monitoring model comprising five metabolites, six lipids, and three proteins [503]. In addition, by integrating genomic, transcriptomic, and proteomic analyses of a pancancer cohort, another study identified RNA methylation regulators as predictive biomarkers for monitoring the tumor microenvironment, patient prognosis, and treatment response [504].

Despite significant advances in chemotherapy, targeted therapy, and immunotherapy, the efficacy of these treatments remains limited by treatment resistance. Multi‐omics approaches provide detailed insights into treatment response and resistance mechanisms. A study using proteomics and transcriptomics analyses revealed that wild‑type IDH1 reprograms nucleotide metabolism during chemotherapy, linking DNA damage repair to ferroptosis resistance via DHODH and thereby promoting chemotherapy resistance in nasopharyngeal carcinoma [505]. Through transcriptomic and metabolomic analyses of pancreatic cancer mouse models and cell metabolites, another study revealed that extracellular matrix‑induced guanosine accumulation alleviated oxaliplatin‑induced DNA damage [506]. Additionally, a comprehensive analysis of the proteome, transcriptome, and genome of 60 muscle‐invasive bladder cancers revealed the abundance of ATAD1 and RAF family protein short isoforms as biomarkers for chemotherapy resistance. Wnt signaling via GSK3B‐S9 phosphorylation and the JAK/STAT pathway are potential targets for overcoming chemotherapy resistance [507]. An integrated analysis of proteomics and genomic sequencing data from peripheral blood and tumor samples from 75 endometrial carcinoma patients revealed that the levels of CCL23 and CSF1/M‐CSF in plasma monocyte/macrophage mobilization correlate with resistance to nivolumab plus cabozantinib [508].

### Section summary

Multi‐omics technologies hold substantial potential for precision cancer therapy. Cancer is a complex ecosystem of cells with diverse genetic and phenotypic characteristics, involving synergistic or antagonistic changes across the genome, epigenome, transcriptome, proteome, and metabolome. Although conventional single‑omics approaches have provided valuable insights into tumor biology, they focus on a single level and fail to provide a complete biological picture. In contrast, multi‑omics technologies have enhanced our understanding of key aspects of tumor biology, including clonal evolution, the tumor microenvironment, and tumor heterogeneity. The integration of multi‑omics data reveals the complex molecular mechanisms of tumors and provides clinicians with informative molecular targets for tumor diagnosis and prognosis prediction. Additionally, it offers a more comprehensive and refined classification of cancer molecular subtypes, identifies high‐risk patients, and provides personalized treatment options. It also supports the prediction and monitoring of treatment responses in real time, facilitating timely treatment adjustment. Multi‐omics integration across molecular levels has advanced modern oncology research, driving a paradigm shift in cancer treatment from a population‑based standardized workflow toward an individualized model.

## MULTI‐OMICS IN AUTOIMMUNE DISEASE STRATIFICATION AND THERAPY

**Figure 8:**
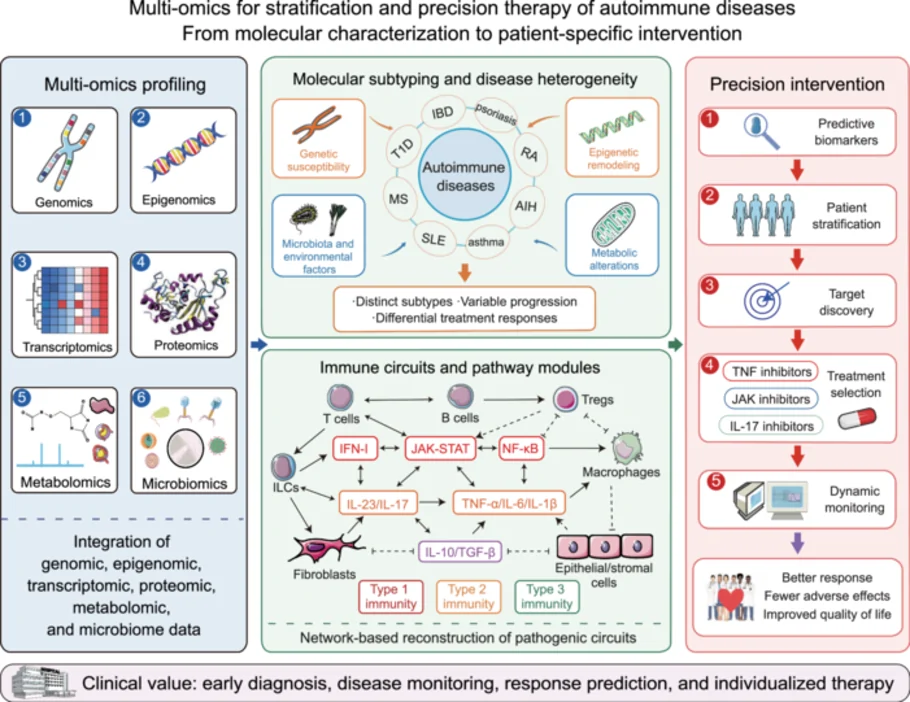
Multi‐omics technologies for decoding immune‐mediated inflammatory diseases and guiding precision intervention. Multi‐omics technologies—encompassing genomics, epigenomics, transcriptomics, microbiomics, proteomics, and metabolomics—enable the dissection of immune cell heterogeneity and metabolic programming in immune‐mediated inflammatory diseases (IMIDs). These approaches facilitate the elucidation of core pathogenic mechanisms across IMIDs, such as inflammatory bowel disease (IBD), rheumatoid arthritis (RA), psoriasis, systemic lupus erythematosus (SLE), autoimmune hepatitis (AIH), asthma, multiple sclerosis (MS), and type 1 diabetes (T1D), thereby supporting the identification of therapeutic targets associated with type 1, type 2, and type 3 immunity, patient stratification, and precision intervention.

Traditional biomarkers provide useful clinical information in autoimmune disease, but their limited sensitivity and specificity limit early diagnosis, disease monitoring, and prediction of treatment response. Because autoimmune disorders are highly heterogeneous, clinically similar presentations may reflect very different underlying immune programs. Multi‐omics technologies offer a route to resolve this heterogeneity by integrating genomics, transcriptomics, proteomics, metabolomics, epigenomics, and microbiome‐related data. In this section, we focus on how these approaches support molecular subtyping, the delineation of immune circuits and pathway modules, and the prediction of therapeutic response in autoimmune disease (Figure 8).

### Molecular subtyping and disease heterogeneity of autoimmune diseases

Molecular subtyping is essential for autoimmune diseases owing to their pronounced clinical heterogeneity, as reflected in their divergent symptoms, disease trajectories, and treatment responses. Conventional diagnostic and therapeutic methods often cannot adequately address these variations, making multi‐omics analysis a valuable tool for identifying distinct molecular markers that underpin more precise, individualized disease classification. Environmental influences, including microbiota composition, lifestyle choices, and epigenetic modifications, contribute critically to the development of autoimmune diseases [509]. This is particularly significant, as genetic factors, while relevant, explain less than 20% of disease variability [510]. Even within a single autoimmune disease, substantial differences in onset age, severity, and disease progression rate are common, underscoring the limitations of traditional classification frameworks. Integrating genetic, environmental, and phenotypic data through molecular approaches not only increases diagnostic precision but also promotes personalized treatment regimens.

Advances in genomics and multi‐omics approaches have enabled the identification of not only genetic variations associated with autoimmune diseases but also key factors such as microbiota composition and metabolic alterations [511]. Integrating data from genomics, transcriptomics, metabolomics, and microbiomics provides deeper insights into the complex interactions driving autoimmune diseases, paving the way for more precise disease subtyping and personalized treatment strategies [50]. The significant variability in clinical presentations and genetic profiles across autoimmune diseases underscores the importance of molecular classification for unraveling their complexity. More than 80 autoimmune diseases have been identified, and they are commonly classified by the primary organs they target, such as joints in rheumatoid arthritis, skin in psoriasis, the central nervous system in multiple sclerosis, the intestines in inflammatory bowel disease, and the pancreas in type 1 diabetes [512], as well as by factors such as disease onset timing, tissue damage extent, and genetic predispositions.

GWASs have identified thousands of susceptibility loci linked to immune‐mediated disorders [513, 514]. Despite distinct loci, these diseases share disruptions in immune pathways, such as T‐cell activation, with genes, such as *MHC* and *CTLA‐4,* implicated in conditions, such as rheumatoid arthritis and type 1 diabetes [515]. This genetic overlap suggests common mechanisms and potential for targeted therapies [516]. GWAS‐based disease subtyping of Crohn's disease and ulcerative colitis identified 163 loci, many of which are shared with other immune disorders and mycobacterial infections, including 71 new associations [517]. These findings highlight the potential for disease subtyping and targeted treatments based on shared genetic mechanisms. While previous studies have focused on individual diseases, these large‐scale analyses emphasize commonalities across autoimmune and inflammatory disorders. A study analyzing 129,058 case and control samples revealed that approximately 40% of the genetic overlap in these diseases arises from the same alleles. By fine‐mapping the shared alleles, additional expression quantitative trait loci (eQTLs) driven by these genes were identified, revealing shared pathogenic mechanisms. An association at the *RGS1* locus is also observed in celiac disease and multiple sclerosis, suggesting potential common mechanisms [518].

DNA hypomethylation plays a key role in the pathogenesis of autoimmune diseases. In systemic lupus erythematosus (SLE), CD4^+^ T cells exhibit hypomethylation of the *LFA‐1* promoter region, leading to the overexpression of *LFA‐1* and autoreactive T‐cell activity [519]. SLE B cells also exhibit hypomethylation, which contributes to abnormal autoantibody production. In psoriasis and rheumatoid arthritis, altered DNA methylation patterns are linked to immune dysregulation and chronic inflammation [520]. These epigenetic changes are crucial for disease progression and may provide targets for disease subtyping and future therapies.

Single‐cell spatial transcriptomics reveals key immune niches in chronic active multiple sclerosis (MS) lesions, highlighting dysfunctional microglia and lipid metabolism as drivers of inflammation [521]. In SLE, single‐cell RNA sequencing has been used to identify distinct CD4^+^ T‐cell subsets, including expanded memory and dysfunctional Treg cells, that contribute to aberrant immune responses and disease progression [522]. Using omics approaches to identify oligodendrocyte heterogeneity in MS has revealed distinct subpopulations within MS lesions and normal‐appearing white matter, offering valuable insights for disease subtyping and therapeutic development [523]. These findings underscore the potential of multi‐omics for identifying disease subtypes and mechanisms.

Metabolomics enables the identification of plasma biomarkers for predicting rheumatoid arthritis (RA) onset, assessing disease activity, and evaluating treatment outcomes. Analysis of 209 RA patients revealed that metabolites linked to methylation and redox imbalance, such as *S*‐adenosylmethionine and creatinine, are associated with RA progression. This highlights metabolomics as a valuable tool for early diagnosis and treatment evaluation in patients with RA [524]. Metabolomics has revealed that spermine is a regulator of JAK signaling and suppresses autoimmune pathways in diseases such as SLE, underscoring its potential as an immunosuppressive therapeutic target [525, 526]. In addition, lung microbiota dysbiosis influences susceptibility to central nervous system autoimmune diseases, such as multiple sclerosis, by modulating immune responses through a lung–brain axis. This highlights the role of the microbiota in disease subtyping and autoimmune mechanisms [527, 528].

### Immune circuits, pathway modules, and therapeutic implications in autoimmune diseases

Multi‐omics technologies enable the comprehensive profiling of immune pathways at the levels of gene expression, proteins, and metabolites [529]. The integration of disease‐associated immune cells and their target pathways into a unified network provides a systemic view of immune mechanisms underlying autoimmune diseases, facilitating both therapeutic target identification and deeper insights into immune dysregulation.

Dysregulated immune activation in autoimmune diseases involves major immune cell types, such as T cells, B cells, Tregs, and ILCs [530], which interact through key signaling pathways, including the NF‐κB, JAK‐STAT, and type I interferon (IFN‐I) pathways [531]. Multi‐omics analyses have linked CD4^+^ T‐cell hyperactivation via IFN‐I and JAK‐STAT signaling to SLE [532]. Single‐cell multi‐omics analysis revealed IFN‐driven alterations in T lymphocytes and NK cells in systemic lupus erythematosus, revealing rare immune subsets and altered phenotypes. This approach provides novel peripheral biomarkers of disease activity and enhances sensitivity in immune profiling [533]. T‐cell and B‐cell dysregulation contribute to the pathogenesis of RA, resulting in abnormal immune responses. Peripheral helper T cells (TPHs), which are composed of stem cell‐like TPHs (S‐TPHs) and effector TPHs (E‐TPHs), play crucial roles in promoting synovial tissue inflammation and activating B cells. S‐TPH cells are distributed mainly in tertiary lymphoid structures (TLSs), which possess self‐renewal capacity and strong B‐cell‐helper activity. Conversely, E‐TPH cells are located near macrophages and CD8^+^ T cells, where they secrete effector molecules and exacerbate the inflammatory response [534]. Furthermore, single‐cell RNA sequencing of synovial fluid revealed pathogenic cell populations, including *SPP1* ^+^ macrophages and *CXCL13* ^+^ CD4^+^ T cells. TNF/JAK inhibitors can block the interactions between these pathogenic cells, a factor in the therapeutic efficacy of such drugs [535]. In addition, the six distinct cell type abundance phenotypes (CTAPs) found in the synovial tissue of patients with RA highlight the heterogeneous nature of the disease. These CTAPs possess unique molecular characteristics that can predict treatment response and guide the development of targeted therapies [536]. Furthermore, multi‐omics analysis suggests that aging drives the expansion of age‐related T helper cells, influencing disease activity and treatment response in diseases such as RA and SLE [537]. This highlights how multi‐omics approaches reveal disease‐specific immune dysregulation in autoimmune diseases such as IBD and MS, identifying key immune cell functions and interactions that offer potential therapeutic targets [538].

These multi‐omics studies systematically identified key inflammatory signaling pathways (e.g., NF‐κB, JAK‐STAT, and the IL‐23/IL‐17 axis), along with disease‐specific molecular signatures [525, 539, 540, 541, 542, 543]. This integrative approach deepens the understanding of autoimmune pathogenesis and accelerates the identification of potential therapeutic targets, thereby advancing precision medicine. By classifying patients on the basis of distinct molecular and cellular characteristics, multi‐omics strategies facilitate the development of personalized treatments aimed at restoring immune tolerance, providing a promising approach for complex and challenging autoimmune diseases.

Multi‐omics strategies have revealed associations between cytokine signatures and autoimmune disease pathogenesis, providing insights into disease‐specific processes and identifying promising therapeutic targets. Type 1 immunity is closely associated with diseases such as RA and type 1 diabetes (T1D). These cytokines drive Th1 cell differentiation and activate macrophages and CD8^+^ T cells, thereby mediating tissue inflammation and autoimmunity via activation of the NF‐κB and JAK–STAT signaling pathways. Multi‐omics approaches have identified the IL17REL as a decoy receptor for IL‐17, with IBD‐associated variants failing to suppress inflammation, suggesting its potential as a therapeutic target [544, 545]. Multi‐omics analysis, including single‐cell RNA sequencing and metabolic profiling, revealed that rapamycin reprograms *Cxcl9* ^+^ macrophages through the mTORC1‐C/EBPβ‐OSM axis, suggesting a potential therapeutic strategy for myocarditis and chronic inflammatory cardiomyopathy [546].

Type 2 immunity is involved in the pathogenesis of various diseases, including asthma, allergic rhinitis, and SLE. IL‐4 and IL‐13 play central roles in promoting Th2 cell differentiation, B‐cell activation, and IgE production, processes that underlie chronic inflammation and tissue damage in these diseases [547]. In SLE, the overactivation of eosinophils and mast cells downstream of type 2 immunity further exacerbates the systemic inflammatory response [548]. Multi‐omics analysis revealed that IL‐25‐driven ILC2 activation enhances mucosal barrier protection and resistance to pathogens, revealing an immune resilience mechanism that may inform therapeutic strategies for autoimmune diseases that do not induce chronic inflammation [549].

Type 3 immunity is crucial in diseases such as IBD, psoriasis, and ankylosing spondylitis. IL‐17A and IL‐23 drive Th17 cell differentiation, thereby promoting chronic inflammation and epithelial damage in the gut and skin [550]. The IL‐17/IL‐23 axis is a key driver in the pathogenesis of IBD; these cytokines can induce mucosal inflammation and disrupt epithelial barrier integrity [551]. IL‐22 contributes to tissue repair and regeneration, but its dysregulation can promote pathological fibrosis [552]. Single‐cell RNA‐seq analysis revealed that *CTLA‐4* upregulation on ILC3s is a critical checkpoint that regulates IL‐23‐mediated immune responses, revealing a mechanism that balances tissue protection and inflammation in IBD [553]. In addition, multi‐omics and MERFISH profiling of colitis have revealed dynamic immune and nonimmune cell interactions, including fibroblast polarization and recruitment, providing insights into inflammation‐induced tissue remodeling and identifying conserved processes in ulcerative colitis [554]. The multi‐omics analysis of immune pathways in autoimmune diseases enables a comprehensive understanding of disease mechanisms, facilitates the discovery of new molecular targets, and offers insights for personalized treatment strategies.

Multi‐omics analysis of cytokine networks reveals the complex interplay between immune cell subtypes and signaling pathways, such as the IL‐17, JAK–STAT, and NF‐κB pathways, highlighting their differential roles in the pathogenesis of autoimmune diseases such as RA and MS while providing targeted therapeutic insights [555]. Furthermore, multi‐omics integration elucidates immune networks, spanning cytokine signaling, cell interaction networks, and disease‐specific pathways, offering crucial insights for targeted therapies and advancing precision medicine [556].

Autoimmune pathology reflects a coordinated dysfunction of multiple immune and stromal compartments rather than an isolated aberration within a single cell type or pathway. Within inflamed tissues, immune cells engage in dynamic intercellular signaling, integrating cytokine networks, metabolic reprogramming, and receptor‐mediated activation cascades, thereby collectively orchestrating tissue‐level inflammatory programs [557].

The advancement of multi‐omics technologies has enabled high‐resolution reconstruction of these cellular communication networks. By integrating single‐cell transcriptomics, spatial transcriptomics, and proteomics, researchers can now map the spatial distribution of immune cell populations and uncover interaction circuits that are difficult to detect through bulk sequencing approaches. This systems‐level strategy facilitates a deeper understanding of how specific cellular subsets coordinate local immune amplification [558].

In RA, synovial inflammation represents a complex immune–stromal ecosystem involving T cells, B cells, macrophages, and fibroblasts. Activated T helper cell subsets produce proinflammatory mediators that, through downstream signaling cascades such as the NF‐κB and STAT pathways, drive macrophage activation. In turn, macrophages secrete TNF‐α, IL‐6, and chemokines, establishing a self‐reinforcing feedback loop that amplifies joint inflammation and promotes tissue remodeling. Multi‐omics‐based stratification analyses further demonstrated that the dominant interaction hubs differ across molecular subtypes of RA [559]. Certain subgroups exhibit enhanced T‐B‐cell communication, whereas others are characterized by macrophage–fibroblast–centered inflammatory circuits. These distinct cellular interaction architectures provide mechanistic insight into clinical heterogeneity and differential therapeutic responses.

The tissue microenvironment profoundly shapes these interaction networks in organ‐specific autoimmune diseases. In MS, single‐cell and proteomic analyses of central nervous system lesions have revealed disrupted signaling between microglia and oligodendrocytes that impairs remyelination and exacerbating neuroinflammation [560]. In IBD, spatial analyses of the intestinal mucosa have demonstrated perturbed communication among innate lymphoid cells, epithelial cells, and stromal components, leading to compromised barrier integrity and persistent mucosal inflammation [561]. Contemporary computational frameworks have progressed beyond simple ligand–receptor pairing networks by integrating interaction strength modeling and network topology analysis. Such approaches facilitate the identification of critical pathogenic communication and potential therapeutic intervention points [562].

Multi‐omics approaches are being increasingly applied to allergic diseases, including asthma, respiratory diseases, and food or drug allergies, to elucidate patient‐specific mechanisms and identify potential biomarkers. Single‐cell and spatial transcriptomics revealed proinflammatory cellular ecosystems in the airway wall of asthma patients, highlighting spatially defined targets for precision intervention [563]. Single‐cell and spatial analyses demonstrated that butyrate alleviates food allergy by restoring epithelial barrier integrity and suppressing oxidative stress‐mediated Notch signaling, suggesting potential therapeutic strategies [564]. Multi‐omics approaches, including single‐cell profiling, revealed that TSC22D3‐regulated NK cell heterogeneity underlies drug hypersensitivity reactions, providing potential targets for precision intervention in severe cutaneous adverse drug reactions [565]. Together, these studies demonstrate that multi‐omics approaches provide a powerful framework for dissecting the heterogeneity of allergic diseases and guiding precision diagnostics and therapeutic strategies.

Cytokines are principal regulators of intercellular communication within the autoimmune microenvironment. Their biological effects are determined not only by expression levels but also by the interplay of temporal dynamics, receptor distribution, and the integration of downstream signaling pathways. Rather than acting independently, cytokines function within interconnected regulatory networks that collectively shape inflammatory phenotypes. Integrated transcriptomic, proteomic, and metabolomic analyses revealed that cytokine systems are frequently organized into coordinated immune modules broadly aligned with immune programs. These modules exhibit internal synergy while maintaining cross‐regulatory constraints with other inflammatory axes. The configuration and dominance of specific modules influence tissue tropism, disease severity, and clinical presentation.

Multi‐omics analyses have enabled the systematic characterization of cytokine network architecture across autoimmune diseases. In patients with SLE, integrated data sets revealed heightened type I interferon signaling in conjunction with IL‐6 and TNF‐α activation, forming a central inflammatory hub associated with disease activity and organ damage. Molecular stratification further revealed that renal involvement is correlated with stronger IFN‐I–IL‐6 coupling, whereas cutaneous manifestations are more closely linked to IL‐17–associated signaling. Similar modular patterns are observed in other disorders: psoriasis and ankylosing spondylitis are often dominated by the IL‐23/IL‐17 axis, whereas RA is characterized by coordinated increases in TNF‐α, IL‐6, and IL‐1β levels, which form a proinflammatory cluster that partially predicts responsiveness to TNF‐targeted therapies [566]. In RA, multi‐omics studies have indicated insufficient activation of regulatory mediators such as IL‐10 and TGF‐β, suggesting that defective counterregulatory circuits represent an additional layer driving persistent immune imbalance [567].

Autoimmune diseases can be conceptualized as multilayer regulatory systems involving genetic susceptibility, epigenetic remodeling, cellular dysfunction, and pathway crosstalk. Through the network‐based integration of multi‐omics data, researchers can reconstruct disease‐associated molecular networks to identify key genes, driver pathways, and core regulatory modules. Multiple autoimmune disorders share common inflammatory pathways, including NF‐κB activation, JAK‐STAT dysregulation, impaired apoptosis, and immune tolerance breakdown [568]. These shared modules provide a mechanistic basis for the cross‐disease efficacy of certain targeted therapies. Moreover, disease‐specific subnetworks determine organ specificity and distinct clinical phenotypes.

Network‐informed stratification further refines prognosis and disease classification. By quantitatively scoring network perturbation patterns through multi‐omics integration, researchers can subdivide clinically similar patients into distinct subtypes characterized by different pathway activation profiles. In IBD, molecular stratification into inflammation‐dominant, microbiota‐associated, or metabolism‐related subtypes is correlated with therapeutic outcomes. In RA, clustering on the basis of inflammatory burden supports the selection of individualized treatment strategies. Moreover, the dynamic assessment of network states can provide specific indicators of disease progression, often outperforming single biomarker measurements.

### Predictive biomarkers and treatment in autoimmune diseases

Therapeutic responses in autoimmune diseases vary substantially, reflecting their underlying molecular heterogeneity. Conventional treatment strategies often lack predictive precision. Multi‐omics analyses enable the identification of biomarkers associated with drug sensitivity and resistance prior to treatment. By integrating multilayer molecular data, researchers can develop predictive models for targeted therapies such as TNF inhibitors, JAK inhibitors, and IL‐17 antagonists. For example, in IBD, integrated genomic and mucosal transcriptomic analyses have indicated that genetic susceptibility variants, baseline inflammatory gene expression, and systemic metabolic features are associated with responsiveness to TNF blockade [569]. Persistent NF‐κB activation or the dysregulation of drug transporter expression may predict primary resistance. Furthermore, posttreatment multi‐omics monitoring enables early evaluation of therapeutic efficacy through changes in pathway activity and cytokine profiles.

Multilayer molecular profiling expands the scope of clinically relevant biomarkers beyond classical autoantibodies and inflammatory indicators. Integrative biomarker systems that combine multi‐omics data can substantially improve diagnostic and prognostic accuracy. Early metabolic alterations and circulating noncoding RNAs may precede clinical onset in certain diseases, enabling preclinical risk identification [570]. In active disease, immune cell subset signatures and cytokine profiles often reflect inflammatory status more accurately than conventional markers do.

The same multi‐omics framework also facilitates the expansion of therapeutic targets in autoimmune diseases. Rather than focusing solely on individual cytokines, researchers are increasingly directing attention toward pathway‐level dysregulation, metabolic dependencies, and epigenetic regulatory factors. The integration of computational approaches with artificial intelligence further enables the prioritization of candidate targets and the optimization of combination therapies, particularly in the context of treatment‐resistant disease.

### Section summary

Comprehensive multi‐omics has redefined autoimmune diseases as dynamic network disorders characterized by coordinated perturbations across cellular, cytokine, and regulatory layers. By resolving disease heterogeneity at multiple biological scales, multi‐omics approaches enable mechanistic stratification, the predictive modeling of therapeutic response, and rational target discovery. Continued methodological refinement and integration into clinical workflows will enhance translational applicability and support the broader implementation of precision immunology.

## MULTI‐OMICS IN CARDIOMETABOLIC DISEASE STRATIFICATION AND INTERVENTION

### The role of multi‐omics in the study of molecular mechanisms of cardiometabolic diseases

Cardiometabolic disease (CMD) encompasses a spectrum of interrelated disorders driven by metabolic dysregulation and involving the cardiovascular system, including diabetic cardiomyopathy, dyslipidemia, and atherosclerotic cardiovascular disease [571]. Despite advances in prevention and treatment, CMD often evolves silently, with molecular and tissue‐level abnormalities preceding overt clinical symptoms. Consequently, conventional biomarkers are often insufficient to enable early detection, mechanism‐based stratification, and individualized intervention [572].

Multi‐omics approaches help address this gap by resolving the gene‐, protein‐, metabolite‐, and microbiome‐level alterations that underlie CMD pathogenesis [573]. In doing so, they provide a molecular framework for earlier risk prediction, more precise phenotyping, and personalized treatment.

#### A multi‐omics study using diabetic cardiomyopathy as an example

Diabetic cardiomyopathy (DCM) is a distinct form of myocardial dysfunction that occurs in diabetic patients in the absence of coronary artery disease, hypertension, or other conventional cardiovascular risk factors. It is characterized by myocardial hypertrophy, fibrosis, and impaired systolic and diastolic function. Over time, it may progress to heart failure, of which the preserved ejection fraction subtype accounts for approximately 50% of cases [574]. The pathogenesis of DCM is rooted in metabolic dysregulation, and previous studies have largely focused on discrete mechanisms such as hyperglycemia‐mediated myocardial injury, mitochondrial dysfunction, lipotoxicity, chronic inflammation, and insulin signaling pathway dysregulation [575]. Nevertheless, the synergistic interactions among these mechanisms remain poorly understood, thus limiting a comprehensive understanding of DCM pathophysiology. To overcome the limitations of single‐mechanism studies and enable systematic analysis of molecular regulatory networks in DCM, systems biology approaches that integrate multi‐omics data are urgently needed. Such strategies may shift the research paradigm from isolated molecular events toward coregulation across multiple mechanisms, facilitating the identification of core targets and key pathways underlying DCM pathogenesis.

Several studies have advanced the understanding of DCM molecular mechanisms through multi‐omics integration strategies. For example, a 2025 study integrating transcriptomics, proteomics, and metabolomic data from a type 2 diabetic mouse model of DCM and cardiac samples from clinical patients. The study not only validated the link between hyperglycemia‐mediated metabolic reprogramming and myocardial fibrosis but also identified ACBP as a key target in DCM pathogenesis. ACBP contributes to myocardial injury in DCM by regulating cardiac lipid metabolism and mitochondrial function [576]. In contrast to traditional fragmented approaches using a single omics layer, this multi‐omics integration advances the analysis from the identification of individual molecules to the elucidation of complete regulatory pathways. This approach not only identifies key drivers of disease pathology but also systematically elucidates the associated molecular regulatory networks and downstream biological consequences. Collectively, these findings provide theoretical support and identify new candidate targets for precise therapeutic intervention in diabetic cardiomyopathy [577].

The application of multi‐omics technologies to investigate DCM mechanisms extends beyond molecular interactions within myocardial tissue, with the research scope expanding to include emerging approaches such as metagenomics. These approaches have established a link between the gut microbiota and disease pathogenesis [573]. As the largest microbial ecosystem in the human body, the gut microbiota regulates host myocardial metabolism, inflammatory responses, and mitochondrial function through the gut–heart axis. This cross‐organ regulatory mechanism is often overlooked in traditional studies, which focus primarily on local molecular changes in the myocardium while neglecting the synergistic interaction between the gut microbiota and host cardiac tissue [578]. The combined application of metagenomics and other omics technologies fills this research gap, providing an avenue to elucidate the complex pathogenesis of DCM at the systems level. Recent studies on the gut–heart axis have shown that microbial metabolites exhibit bidirectional regulation [579]. Short‑chain fatty acids, hydrogen sulfide, and tryptophan‑derived indole metabolites exert cardioprotective effects by modulating myocardial receptor signaling [580, 581, 582, 583], suppressing inflammatory and oxidative stress responses, and restoring mitochondrial function. In contrast, metabolites such as trimethylamine N‐oxide (TMAO) and lipopolysaccharides exacerbate myocardial fibrosis [584, 585], lipotoxicity, and energy metabolism disorders, thereby driving disease progression. Moreover, gut microbiota dysbiosis induces metabolic reprogramming in the myocardium by remodeling glucose, lipid, ketone, and branched‐chain amino acid metabolism [586, 587, 588]; regulates the expression of fibrosis‐related genes via epigenetic modifications; and suppresses inflammation and oxidative stress [589]. Together, these processes mediate the transition from diastolic dysfunction to systolic failure. Compared with single‑tissue omics studies, gut microbiota research broadens the understanding of DCM molecular mechanisms and provides a more comprehensive view of the multiorgan regulatory networks underlying disease pathogenesis.

#### The future of multi‐omics research in cardiometabolic diseases

**Figure 9:**
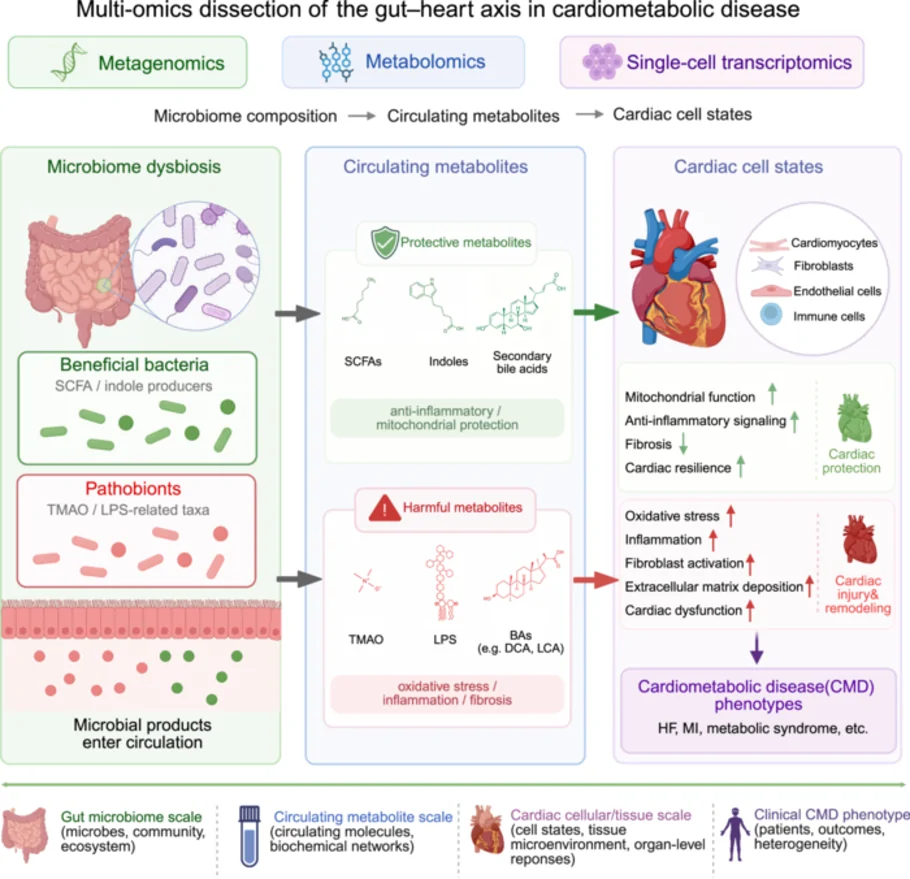
Multi‐omics approaches in precision therapy for cardiometabolic diseases. Metagenomics and targeted metabolomics reveal how beneficial and pathogenic gut microorganisms, together with their circulating metabolites (including short‐chain fatty acids, indole derivatives, TMAO, and branched‐chain amino acids), influence cardiac remodeling. These metabolites affect pathways related to mitochondrial dysfunction, fibrosis, and macrophage infiltration, thereby linking the gut–heart axis to cardiometabolic disease progression.

With the development of multi‐omics technology, increasing precision will become a central focus of CMD research. This contrasts with traditional omics approaches, which typically provide averaged molecular profiles and lack the resolution to capture disease‑specific regulation or spatiotemporal dynamics. Through the integration of diverse and innovative omics technologies, CMD research will advance toward higher resolution and greater systematic coverage. In terms of research content, future efforts will focus on targeted exploration grounded in the pathological characteristics of CMD. Lipid metabolomics and bile acid metabolomics may enable more precise analyses of pathological processes such as lipotoxicity and bile acid metabolism imbalance [590, 591]. Moreover, post‑translational modification omics, such as phosphorylation and acetylation, help overcome the limitation of *“only looking at expression and not looking at function*” [592]. These approaches can facilitate the more efficient screening of effector molecules that play key roles in disease pathogenesis, moving beyond the generalized framework of metabolic disorders. At the research level, the focus will shift from single‑layer characterization to multilayer integration. Metatranscriptomics and metaproteomics can elucidate the functional mechanisms of the gut–heart axis [593], whereas spatial proteomics and spatial metabolomics can resolve local microenvironmental regulatory characteristics across distinct pathological regions of the myocardium. Single‑cell multi‐omics analysis can elucidate the cooperative interactions among diverse cell subtypes and reveal the key roles of rare cell populations [594], reflecting the pathological complexity of CMD, which involves coordinated cellular participation and pronounced spatiotemporal heterogeneity. In the future, the integration and collaborative application of these emerging omics technologies will provide a solid theoretical foundation for identifying early specific molecular markers of CMD, screening targets for precision therapy, and deepening the understanding of disease mechanisms. Collectively, these advances will continue to advance precision prevention and treatment strategies for CMD [595, 596] (Figure 9).

### Application of multi‐omics in risk prediction and personalized treatment of cardiometabolic diseases

As multi‐omics technologies deepen the understanding of the molecular mechanisms of CMD, they are gradually expanding from basic research to clinical translation and have become important tools for the precise prevention and control of CMD [597]. In contrast to traditional clinical detection methods that rely on single biomarkers, multi‐omics approaches integrate multilayer molecular data with clinical phenotypes to capture the molecular signatures of disease occurrence and progression with greater precision. They exhibit unique advantages in early risk prediction, personalized treatment design, and the assessment of efficacy and prognosis, facilitating the transformation of clinical interventions for CMD from empirical medicine toward precision medicine [598].

#### Integrating multi‐omics for disease risk prediction

Traditional risk prediction of CMD mostly relies on routine clinical indicators such as blood glucose levels, blood lipid levels, and blood pressure. Although sufficient for basic screening, such approaches fail to identify individuals who develop CMD despite lacking typical risk factors and cannot capture early disease abnormalities. Multi‐omics enables the construction of more specific and accurate risk prediction models by integrating multilayer molecular features, providing early warning and risk stratification.

At the genomic level, polygenic risk scores (PRSs) have become important tools for genetic risk assessment because they integrate many common single‐nucleotide polymorphisms (SNPs) associated with CMD [599]. Studies indicate that individuals with high PRS have a 2‐ to 4‐fold higher risk of coronary heart disease and type 2 diabetes than the general population does, particularly among those of European ancestry. PRS comprising numerous SNPs identifies high‐risk individuals who are often missed by traditional risk assessment. However, existing PRS models are predominantly based on European data. Owing to differences in linkage disequilibrium patterns and gene–environment interactions across ethnic groups, the prediction accuracy of PRS models in non‑European populations requires further improvement. Diverse cohort studies are needed to optimize the applicability of PRS models [600]. Additionally, single‐gene mutation detection holds unique value in risk prediction for Mendelian genetic diseases such as familial hypercholesterolemia, enabling early diagnosis and intervention through accurate identification of mutations in genes such as *LDLR* and *APOB* [601].

Metabolomics is particularly advanced in profiling branched‑chain amino acids (BCAAs), specific lipids, and gut microbiota metabolites [182]. Multiple prospective cohort studies have confirmed that elevated plasma levels of BCAAs predict the development of type 2 diabetes. A metabolic score combining BCAAs with aromatic amino acids further improves the predictive power of traditional risk models [602]. In cardiovascular diseases, specific lipids such as cholesteryl esters and phosphatidylcholine, along with the gut microbiota metabolite TMAO [603], predict the risk of coronary heart disease and heart failure independently of traditional lipid indicators, particularly in individuals with well‑controlled lipid levels who nevertheless remain at residual risk. These metabolic markers directly reflect metabolic imbalances resulting from the interplay of genetic, environmental, and lifestyle factors and serve as key links between genetic background and clinical phenotype [604].

Recent studies have further confirmed that combined analysis of the gut microbiome and metabolome can reveal the individualized risk characteristics of CMD [605]. Patients with acute coronary syndrome (ACS) have specific abnormalities in the serum metabolome and gut microbiota: The abundance of the unknown species of Clostridiaceae (SGB 4712) in the microbiota is significantly reduced, and the abundance of this bacterium is positively correlated with the abundance of a variety of cardiovascular protective metabolites (such as ergothionine) and negatively correlated with the abundance of pro‐cardiovascular toxic metabolites such as p‐cresol, indoxyl sulfate, and phenylacetylglutamine [606]. Moreover, deviations related to metabolic disorders (such as persistently elevated BCAA levels and abnormal TMAO levels) occur not only in CMD patients but also in healthy people with impaired metabolism, suggesting that microbiome and diet‐related metabolic disorders may be the core causes of early pathological changes in CMD patients. In addition, the body mass index (BMI) prediction model based on the serum metabolome can accurately predict the deviation of “actual BMI and metabolic imbalance” in CMD patients, which is closely related to the severity of diabetes complications and coronary artery disease, providing a new metabolic marker for risk stratification.

Proteomics enhances the accuracy of risk prediction by capturing functional regulation. Protein risk models incorporating biomarkers such as growth differentiation factor‐15, which can be identified through high‐throughput methods, including multiplex antibody microarrays and aptamer technology, improve the predictive accuracy of cardiovascular events in patients with stable coronary artery disease [607]. Such functional protein‑based biomarker combinations reflect abnormal signaling pathways and functional damage in cardiomyocytes at an early stage, thereby providing a more targeted molecular basis for early disease screening [608].

Lipidomics, an emerging type of omics technology, can be used to analyze plasma lipid subtypes. Liquid chromatography–mass spectrometry (LC–MS), direct infusion mass spectrometry and other technologies have confirmed that lipid subtypes such as ceramide, sphingolipid, and glycerophosphatidylcholine are strongly related to the risk of CMD: elevated ceramide levels can independently predict the risk of cardiovascular death, and abnormal sphingolipid profiles are closely related to coronary plaque vulnerability [609]. Large‐scale retrospective studies have shown that after lipidomic markers (such as ceramide scores) and proteomic markers are added to the traditional clinical risk model, the area under the curve (AUC) of the model increases from 0.6–0.7 to 0.7–0.9, and the net reclassification improvement index (NRI) significantly improves, resulting in a steep improvement in risk prediction performance [610]. At present, the high‐throughput protein/lipid detection platform based on Olink proximity extension analysis and SomaScan aptamer technology has the potential for clinical transformation and provides technical support for large‐scale risk screening for CMD [611].

The combined application of multi‐omics is not simply the superposition of individual biomarkers but rather the construction of multidimensional risk prediction models through the integration of genetic risk (PRS), metabolic phenotypes (BCAAs, TMAO, and lipid species), functional regulation (core miRNA–protein networks), and clinical baseline characteristics. Such models not only improve the accuracy and specificity of prediction but also reveal regulatory relationships spanning from genetics to metabolism, providing clear target avenues for subsequent personalized intervention.

#### Multi‐omics‐assisted personalized treatment

The current clinical treatments for CMD remain limited by a “one‐size‐fits‐all” approach. Patients exhibit significant heterogeneity in response rates and adverse reaction risks to the same treatment regimen, which is attributable to variations in genetic background, molecular subtypes, metabolic characteristics, and gut microbiota status. By accurately profiling patient molecular characteristics, multi‐omics technologies support the personalized treatment of CMD across multiple dimensions, including disease molecular subtyping, personalized target screening, treatment response monitoring, and adverse reaction warning [612].

With respect to disease molecular subtyping, multi‐omics‐based classification overcomes the limitations of traditional clinical classification and enables accurate etiology‐oriented classification. For instance, by integrating multi‐omics data, researchers can stratify DCM patients into subtypes characterized by PI3K–AKT–mTOR pathway dysregulation [613], AGE–RAGE pathway activation [614], inflammation predominance, or metabolic reprogramming abnormalities. Patients with PI3K–AKT–mTOR pathway dysregulation often exhibit elevated *AKT1* phosphorylation and are candidates for pathway modulators such as rapamycin analogs [615]. The AGE–RAGE pathway‐activated subtype, characterized by high *AGER* gene expression and elevated oxidative stress markers, may be targeted by RAGE inhibitors and antioxidants [614]. In the metabolic reprogramming subtype, the disruption of BCAA or bile acid metabolism is the core feature, necessitating targeted dietary modification or the use of metabolic modulators [616]. This molecular subtyping approach shifts treatment from symptomatic to etiological intervention, thereby improving the precision and effectiveness of therapy.

Multigrouping of atherosclerosis further revealed the clinical significance of plaque heterogeneity. On the basis of the combined analysis of the single‐cell transcriptome and proteome, atherosclerotic plaques can be divided into inflammation‐unstable and fibrosis‐stable types [617]. The former is enriched in TREM2hi foam macrophages, CSF1‐responsive foam macrophages and CD4^+^ T cells lacking CD28, and the risk of plaque rupture is significantly increased. Precise intervention targeting inflammatory pathways is recommended. The latter is dominated by LTBP2^+^/CRTAC1^+^ smooth muscle cells and repair macrophages, which are more suitable for intensive lipid‐lowering and plaque stabilization therapy. Single‐cell multi‐omics also revealed a vascular smooth muscle cell subset with multidirectional differentiation potential, which interacts with macrophages through TNF and ANGPTL signaling to shape the plaque inflammatory microenvironment and is a key driver subset of unstable plaques [618].

The multigroup classification of hypertension is not limited to the traditional binary classification of primary and secondary hypertension but can be divided into salt‐sensitive, renin dependent, endothelial dysfunction, sympathetic excitation, and other functional subtypes by integrating genomic, metabolome, and microbiome data. In addition to the UMOD variant, MTHFR C677T is homozygous and has other genetic subtypes [619]. Patients with UMOD variants have a significantly better response to loop diuretics such as torasemide than to thiazide diuretics because of enhanced renal tubular sodium reabsorption, and their systolic blood pressure can be reduced by 8.5 mmHg. MTHFR C677T homozygous patients with hyperhomocysteemia need a high dosage of folic acid (≥1.6 mg/day) to effectively reduce the risk of cardiovascular events [620]. For refractory hypertension, a multi‐omics analysis revealed a subgroup of patients with hyperactivated endothelin‐1 (ET‐1) pathway who responded best to the novel dual endothelin receptor antagonist aprocitentan, with a systolic BP reduction of 15.3 mmHg.

Multi‐omics technologies also enable monitoring of treatment response and early warning of adverse reactions. In terms of efficacy monitoring, dynamic assessment of molecular marker changes during treatment enables the real‑time evaluation of intervention effects. For example, in patients with AKT1 activation abnormalities, a decrease in phosphorylated AKT1 levels, an increase in hsa‑miR‑302a‑3p expression, and downregulation of myocardial fibrosis markers (such as TGFB1 and Col1a1) after treatment collectively indicate therapeutic effectiveness. If metabolomic testing reveals persistently elevated BCAAs or no improvement in TMAO levels, treatment may require adjustment. To provide early warning of adverse effects, combined genomic and proteomic analysis can be used to identify individuals who are susceptible to specific drugs. For instance, patients with specific genetic polymorphisms may exhibit abnormal metabolic responses to SGLT2 inhibitors or a higher risk of muscle‑related adverse effects with statins [621].

### Section summary

Multi‐omics technologies have become central to advancing precision diagnosis and treatment for CMD. By integrating multilayer data from transcriptomics, proteomics, and metabolomics, these technologies have achieved breakthroughs not only in elucidating molecular mechanisms but also in translating findings toward clinical applications. These advances include validating the link between hyperglycemia‑mediated metabolic reprogramming and myocardial fibrosis in DCM, identifying core targets such as ACBP, and uncovering cross‑organ regulatory mechanisms of the gut–heart axis through metagenomics [622]. However, practical challenges remain, including insufficient cohort diversity, a lack of technical standardization, and difficulties in data integration. In clinical translation, the application of multi‐omics technologies in risk prediction and personalized treatment aims to achieve accurate risk stratification and tailored treatment through the systematic analysis of disease molecular characteristics. Its core value lies not only in the discovery of new molecular markers and therapeutic targets but also in the construction of complete regulatory networks spanning genes, metabolism, function, and clinical phenotype, providing an integrative framework for the precise prevention and management of diseases.

## MULTI‐OMICS IN RENAL–METABOLIC DISEASE MANAGEMENT

With the increasing incidence of diabetes, obesity, and metabolic syndrome [623, 624, 625], metabolism‐related renal diseases are becoming prevalent worldwide [626], posing a major challenge to public health systems. These renal diseases include a spectrum of kidney disorders arising from systemic perturbations in glucose, lipid, uric acid, and energy metabolism [627, 628, 629]. Their common clinical presentations include diabetic kidney disease, obesity‐ and metabolic syndrome–associated chronic kidney disease, and hyperuricemia‐related nephropathy [630, 631, 632]. Consistent with their various clinical manifestations, these conditions have diverse pathogenic mechanisms. These include altered cellular metabolism [633, 634, 635], chronic low‐grade inflammation [636], mitochondrial dysfunction [637], and progressive renal fibrosis [638, 639, 640]. As leading contributors to chronic kidney disease (CKD) and end‐stage kidney disease, these disorders impose substantial morbidity, mortality, and healthcare burden [641, 642]. However, conventional diagnostic and therapeutic strategies largely rely on estimated glomerular filtration rate, albuminuria, and limited single biomarkers [630, 643, 644, 645], which remain insufficient to capture the profound molecular heterogeneity underlying disease progression and treatment response. This gap has increasingly highlighted the need for systems‐level approaches capable of integrating multidimensional biological information. In this context, multi‐omics technologies, which integrate genomics, transcriptomics, proteomics, and metabolomics and frequently coupled with single‐cell, spatial profiling, and AI‐driven analytics, have emerged as important tools for precision nephrology [15, 217, 646, 647, 648]. By enabling the comprehensive reconstruction of molecular networks, early identification of high‐risk trajectories, stratified therapeutic targeting, and dynamic monitoring of treatment response, multi‐omics provides a unifying framework for advancing precision therapy in renal–metabolic diseases.

### Molecular mechanism dissection

At the level of molecular mechanism dissection, multi‐omics approaches have substantially advanced our understanding of renal injury and progression. Mechanistic understanding of metabolism‐related kidney diseases, particularly diabetic kidney disease (DKD) and CKD, has evolved in parallel with advances in omics technologies. A clear trajectory emerges: from bulk tissue–level profiling that defined core dysregulated pathways to single‐cell approaches that resolved cell‐type–specific injury states and, finally, to spatial multi‐omics that revealed how these states are organized within pathogenic microenvironments. Together, this technological progression has substantially improved the mechanistic insight into how metabolic stress drives kidney dysfunction.

In the bulk omics era, transcriptomics, proteomics, and GWASs have established the foundational framework of renal–metabolic pathology. Bulk transcriptomic analyses consistently reveal the enrichment of inflammatory signaling, extracellular matrix remodeling, oxidative stress, and metabolic reprogramming pathways in DKD and CKD. Integrative genomics further connected noncoding GWAS variants to kidney cis‐eQTLs and cell type–biased regulatory programs, demonstrating that the inherited risk for hypertension and kidney dysfunction converges on gene networks active in tubular and vascular compartments [649]. Multi‐omics integration frameworks that combine GWAS, transcriptomics, proteomics, and metabolomics, further supported by causal inference strategies such as Mendelian randomization, have refined candidate gene prioritization and linked genetic associations to actionable therapeutic targets [650]. Parallel advances in tissue proteomics have emphasized that protein‐level regulation, encompassing posttranslational modification, degradation, and signaling complex formation, often better reflects the functional phenotype than transcript abundance alone does [651]. Databases such as CKDdb further consolidated differential expression signatures across tissues and biofluids, enabling cross‐study pathway convergence analyses and biomarker hypothesis generation [652]. Collectively, these bulk‐level studies established a central disease axis characterized by metabolic overload, chronic inflammation, and progressive fibrotic remodeling. However, these methods are limited by the inability to distinguish the contributions of individual cell types within heterogeneous kidney tissue.

The advent of single‐cell and single‐nucleus technologies marked a critical shift from pathway‐level to cell‐state–level understanding. High‐resolution atlases of healthy and diseased human kidneys revealed that kidney injury is not a uniform process but a dynamic spectrum of epithelial and stromal state transitions. Large‐scale single‐cell profiling revealed adaptive repair, maladaptive repair, cycling, degenerative, and immune‐activated states across nephron segments and the interstitium, linking specific epithelial states to functional decline [653]. The Kidney Precision Medicine Project (KPMP) expanded this paradigm through the multimodal integration of RNA expression and chromatin accessibility to define injury‐associated transcriptional modules that correlate with unresolved damage and progression [654]. Importantly, multimodal single‐nucleus RNA‐seq and ATAC‐seq analyses of DKD kidneys demonstrated that metabolic stress is coupled to epigenetic remodeling. Reduced chromatin accessibility at glucocorticoid receptor binding sites in proximal tubule cells was associated with dysregulated glucose metabolism and inflammatory gene programs, supporting the concept that metabolic memory is encoded through chromatin‐level alterations [655]. These findings advance the field beyond descriptive pathway activation to provide mechanistic insight into how metabolic stress reshapes transcriptional competence in specific nephron cell types.

Single‐cell approaches also enabled the precise mapping of inflammatory and regulated cell death pathways to discrete cellular compartments. In DKD glomeruli, single‐cell RNA sequencing revealed significant upregulation of TNFSF10 (TRAIL) and its receptor TNFRSF10B (DR5), specifically in podocytes. Functional studies have confirmed that activation of this axis induces PANoptosis, a coordinated form of apoptosis, pyroptosis, and necroptosis, leading to podocyte depletion and worsening of albuminuria in diabetic models [656]. This exemplifies how single‐cell discovery can directly inform mechanistic validation and therapeutic targeting. Complementary time‐resolved multi‐omics analyses of TGF‐β‐driven fibrosis have integrated transcriptomics, proteomics, phosphoproteomics, and secretomics to reconstruct sequential signaling cascades culminating in extracellular matrix deposition [657]. Together, these studies revealed that disease progression reflects orchestrated cell‐state transitions and signaling rewiring rather than the static activation of isolated pathways.

The subsequent integration of spatial transcriptomics and spatial metabolomics further transformed the mechanistic understanding by embedding cell states within their anatomical and microenvironmental context. Spatially resolved multi‐omics profiling revealed distinct glomerular, immune, tubular, and fibrotic microenvironments in human kidney tissue, demonstrating that the fibrotic niche constitutes a coherent molecular domain capable of stratifying disease progression more effectively than conventional histopathology does [658]. Large‐scale multimodal mapping confirmed that maladaptive epithelial states cluster within immune‐rich and matrix‐dense neighborhoods, suggesting that spatially organized inflammatory circuits reinforce injury persistence [653, 654]. Spatial metabolomic analyses further revealed that metabolic programs differ across anatomical regions, which underscores the coupling between structural heterogeneity and metabolic vulnerability [659]. These spatial insights reframed CKD and DKD as disorders of disrupted cellular neighborhoods, in which persistent injury arises from maladaptive interactions between epithelial, immune, and stromal compartments.

Taken together, the progressive transition from bulk omics to single‐cell and spatial multi‐omics has led to the development of a hierarchical mechanistic model of metabolism‐related kidney disease. Bulk profiling defined the overarching metabolic–inflammatory–fibrotic axis; single‐cell technologies resolved the specific cell states and epigenetic programs driving injury, and spatial multi‐omics revealed how these states are embedded within structured microenvironments that amplify and stabilize pathogenic signaling. This layered understanding now positions metabolism‐related kidney disease as a dynamic network disorder in which genetic predisposition, metabolic stress, chromatin remodeling, cellular state transitions, and spatial niche organization collectively determine progression toward fibrosis and functional decline.

### Early diagnosis and risk prediction

Although traditional markers such as eGFR and albuminuria remain indispensable, they primarily reflect late stages of nephron loss. Consequently, kidney–metabolic disease research is undergoing a paradigm shift from functional staging to mechanism‐linked molecular stratification. Instead of relying solely on late‐stage clinical markers, recent studies have used systematic multi‐omics frameworks to identify early molecular signatures. These high‐throughput platforms now serve as discovery engines, revealing specific pathogenic pathways that are activated prior to overt functional decline and enabling more biologically informed early detection, molecular stratification, and prognostic modeling [660, 661].

A key advance in recent years is the identification of molecular biomarkers that precede clinically detectable functional decline, thereby extending the window for early diagnosis beyond traditional indicators such as eGFR and albuminuria. The urinary proteomic classifier CKD273, derived from large‐scale capillary electrophoresis–mass spectrometry profiling, captures early extracellular matrix remodeling and tubular injury signals and predicts CKD progression before a measurable reduction in kidney function can be detected [662]. In parallel, metabolomic studies have identified circulating metabolites, including pantothenic acid and lipid intermediates, which reflect early perturbations in mitochondrial function, oxidative stress, and energy metabolism associated with disease initiation [663, 664]. Collectively, these findings indicate a shift in early diagnosis from detecting structural or functional damage to identifying the initial molecular changes that trigger the disease.

Beyond early detection, multi‐omics biomarkers enable the stratification of patients according to both progression risk and dominant pathogenic mechanisms, addressing the substantial heterogeneity within clinically defined CKD stages. Circulating inflammatory markers such as TNFR1 and TNFR2 robustly predict renal outcomes independent of albuminuria and baseline kidney function, highlighting TNF‐mediated inflammation as a key driver of disease progression [665]. In addition, composite molecular signatures integrating proteomic and metabolomic features, including inflammation, mitochondrial dysfunction, and lipid metabolic dysregulation, provide refined risk stratification beyond conventional clinical indices [661, 663, 664]. At a higher level, integrated multi‐omics analyses using unsupervised clustering and network‐based approaches have identified biologically distinct CKD subtypes (endotypes) characterized by the predominant activation of inflammatory, fibrotic, or senescence‐associated pathways [666]. This framework redefines risk stratification from a purely quantitative process into mechanism‐based classification, assigning patients to distinct disease trajectories with differential progression risk and therapeutic responsiveness.

The integration of multi‐omics biomarkers into predictive frameworks has facilitated the development of robust prognostic models for disease progression and adverse renal outcomes. Multimarker panels such as CKD273, as well as metabolomic signatures reflecting systemic metabolic dysregulation, combine signals from multiple pathogenic pathways into parsimonious yet biologically interpretable predictors of CKD progression [663]. These models consistently demonstrate incremental prognostic value beyond traditional clinical variables, indicating that molecular signatures capture progression dynamics not reflected by eGFR or albuminuria alone. Furthermore, biomarkers such as TNFR1/2 not only predict incident end‐stage renal disease and accelerated eGFR decline but also anchor prognostic models within specific pathogenic pathways, particularly inflammation [665]. This integration of prediction and mechanism enhances both the robustness and interpretability of prognostic assessment. Taken together, prognostic modeling in kidney–metabolic diseases is transitioning from reliance on static clinical indicators toward multidimensional, mechanism‐informed prediction frameworks that more accurately reflect disease progression dynamics.

From a clinical perspective, multi‐omics is particularly relevant to risk assessment in patients whose conventional indicators do not fully reflect underlying disease activity. In early diabetic kidney disease, molecular alterations in tubular injury, extracellular matrix remodeling, inflammation, and mitochondrial metabolism may occur before substantial eGFR decline or persistent albuminuria. Urinary proteomic classifiers such as CKD273, circulating inflammatory markers such as TNFR1/2, and metabolomic signatures of lipid and energy metabolism therefore provide examples of how multi‐omics–derived biomarkers may extend risk prediction beyond conventional staging. Similarly, among patients with comparable CKD stages, integrated molecular profiles may distinguish inflammation‐dominant, fibrosis‐dominant, or metabolism‐dominant trajectories, thereby refining prognostic assessment and identifying patients who may require closer monitoring or earlier therapeutic intensification.

Together, early diagnosis and risk prediction in kidney–metabolic diseases form a continuum from early molecular detection (e.g., CKD273, metabolite signatures) through mechanism‐based stratification (e.g., TNFR1/2, multi‐omics endotypes) to integrative prognostic modeling, reflecting a shift from late‐stage functional assessment to precision, pathway‐guided risk prediction and disease management.

### Pathway‐guided individualized intervention

Building upon advances in early diagnosis and risk prediction, multi‐omics in kidney–metabolic diseases is increasingly extending from risk identification to therapeutic guidance. Molecular signatures that define disease onset, progression, and pathway‐dominant subtypes also provide a mechanistic basis for treatment selection, response monitoring, and interpretation of therapeutic variability. This represents a transition from prediction to intervention, positioning multi‐omics as a framework for mechanism‐guided precision therapy.

In recent years, multi‐omics has evolved from a discovery tool into a structural framework for individualized therapy in kidney–metabolic diseases. Rather than classifying patients solely by clinical indices such as eGFR decline or albuminuria severity, systems‐level analyses redefine CKD and DKD as biologically heterogeneous syndromes characterized by variable dominance of metabolic reprogramming, inflammatory activation, mitochondrial dysfunction, oxidative stress, and fibrotic remodeling. Within this framework, multi‐omics enables mechanism‐based patient stratification, allowing therapeutic strategies to be aligned with pathway‐dominant disease subtypes [667]. This paradigm is exemplified by the use of sodium–glucose cotransporter 2 inhibitors (SGLT2i), whose renoprotective efficacy has been established in large‐scale clinical trials, including CREDENCE and DAPA‐CKD [668, 669, 670, 671, 672, 673]. However, interindividual variability in response suggests that the therapeutic benefit is not uniform but depends on the underlying biological context. Multi‐omics evidence suggests that SGLT2 inhibitors exert pleiotropic effects beyond glycemic control, supporting their preferential efficacy in patients with metabolism‐dominant or bioenergetic dysfunction–associated phenotypes. Thus, treatment selection is increasingly guided not only by clinical stage but also by molecular subtype and pathway activity.

Within this emerging framework, therapeutic response is no longer evaluated solely by changes in renal function but by the extent to which treatment modulates dysregulated molecular networks. Multi‐omics provides mechanistic readouts that enable real‐time assessment of pathway engagement and biological response. For instance, in *db/db* mice, integrated renal and serum proteomic–metabolomic profiling demonstrated that empagliflozin induces coordinated metabolic reprogramming and modulates the purine, pyrimidine, tryptophan, and nicotinamide pathways while alleviating oxidative stress and mitochondrial dysfunction [674]. These findings indicate that therapeutic response can be captured at the level of metabolic network normalization and restoration of tubular bioenergetic homeostasis rather than inferred indirectly from functional outcomes alone. Similarly, in emerging biologic therapies such as extracellular vesicle (EV)‐based interventions for acute kidney injury, the multi‐omics characterization of EV cargo has revealed coordinated miRNA–protein networks regulating inflammation, oxidative stress, and tubular survival pathways [675]. Such analyses provide mechanism‐resolved pharmacodynamic markers, enabling not only the monitoring of treatment response but also the optimization of therapeutic design, including EV source selection, cargo composition, and dosing strategies.

Furthermore, a critical implication of this framework is the ability to explain and predict heterogeneity in therapeutic response, including nonresponse and resistance. Variability in clinical outcomes reflects differences in dominant pathogenic pathways, as well as the presence of compensatory or alternative molecular programs that may attenuate therapeutic effects. For example, while SGLT2 inhibitors exert beneficial metabolic and bioenergetic effects, patients whose disease is driven primarily by inflammation‐ or fibrosis‐dominant pathways may exhibit a limited response, as these mechanisms are not directly targeted by SGLT2 inhibition. Multi‐omics thus provides a basis for identifying nonresponder phenotypes characterized by differences between the targeted pathways and the underlying disease biology. More broadly, cross‐omics integration enables the identification of residual or compensatory pathway activation despite treatment, offering insight into mechanisms of therapeutic escape and informing the development of combination or sequential therapeutic strategies. In this context, resistance is no longer viewed as a binary outcome but as a reflection of network‐level adaptation within a biologically stratified disease system.

Collectively, these lines of evidence support a clinically actionable precision framework in which multi‐omics enables treatment optimization, response monitoring, and resistance characterization. By linking therapeutic effects to pathway‐specific molecular reprogramming, this approach transforms renoprotective therapy from empiric intervention into mechanism‐guided precision medicine, where treatment efficacy is defined by the ability to normalize dysregulated networks within biologically stratified patient subgroups.

### Dynamic monitoring and AI‐assisted decision‐making

Longitudinal monitoring after treatment initiation is a highly relevant clinical scenario. In routine practice, therapeutic response is usually inferred from changes in albuminuria or the slope of the eGFR, which may lag behind the molecular response. Serial proteomic, metabolomic, or inflammatory biomarker profiling could provide earlier pharmacodynamic readouts of pathway engagement, such as reduced tubular injury, attenuated inflammatory activation, or normalization of metabolic stress signatures. When integrated with electronic health record variables and longitudinal clinical trajectories, these molecular data may enable dynamic risk updating and support adaptive treatment adjustment. This provides a practical rationale for AI‐assisted models and future digital twin frameworks in precision nephrology.

A forward‐looking layer of precision medicine involves dynamic monitoring and AI‐assisted decision‐making throughout the therapeutic course. Rather than treating risk assessment as a one‐time classification, longitudinal multi‐omics profiling enables the repeated measurement of molecular states that track treatment engagement, residual risk, and shifting pathophysiology over time. Conceptually, this “closed‐loop” model requires biomarkers that are not only prognostic at baseline but also pharmacodynamically responsive, that is, capable of moving in directions that reflect on‐target pathway modulation. In this context, post hoc biomarker analyses embedded within large SGLT2i programs have been especially informative. In CANVAS, canagliflozin attenuated longitudinal increases in plasma TNFR1 and TNFR2 and produced early and sustained reductions in KIM‐1, supporting the notion that anti‐inflammatory and tubular injury pathways are modulated in parallel with clinical renoprotection [676, 677]. These patterns align with the broader mechanistic framing in nephrology reviews that emphasize the effects of SGLT2i extending beyond hemodynamics to multilayer metabolic and inflammatory reprogramming, motivating the use of serial biomarker sampling as a pharmacodynamic readout in future trials [650, 667]. Parallel experimental multi‐omics further reinforces the feasibility of molecular response monitoring. Integrated proteomics–metabolomics in diabetic models revealed that SGLT2i can reshape nucleotide, tryptophan, and nicotinamide‐related metabolic programs while dampening oxidative stress and mitochondrial dysfunction, providing a plausible mechanism through which early biomarker shifts may predict long‐term benefits [674]. Similar logic applies to other emerging interventions in which the therapeutic product is complex and multicomponent. For example, EV‐based renoprotection studies have demonstrated that multi‐omics profiling of miRNA–protein cargo can define mechanism‐of‐action modules (inflammation control, redox balance, and tubular survival), enabling rational optimization and, critically, longitudinal monitoring of whether those modules are engaged *in vivo* [675].

As trial designs increasingly incorporate serial biosampling, the analytic challenge shifts from identifying static predictors to learning time‐evolving response trajectories. This is where AI becomes enabling rather than merely additive: Models that combine repeated laboratory measures, longitudinal omics features, and EHR‐derived covariates can update individualized risk estimates in near real time and recommend adaptive therapy adjustments. Contemporary EHR‐based time series modeling, including recurrent neural network approaches using longitudinal eGFR trajectories, illustrates how temporal structure improves short‐term progression prediction relative to that of static risk models, providing a computational scaffold onto which omics‐derived pharmacodynamic signals can be layered [678]. This trajectory naturally connects to the new digital twin concept, a computational representation that is iteratively updated with incoming data to simulate counterfactual outcomes under alternative treatment strategies. While digital twin implementations in nephrology remain in an early stage of development, systematic reviews in digital medicine suggest the use of dynamically updated patient‐specific models to support precision health management and individualized therapy evaluation across diseases [679]. In kidney medicine specifically, such an approach depends on standardized, multilayer data infrastructures that can harmonize tissue states, circulating biomarkers, and longitudinal clinical metadata.

Initiatives such as the Kidney Precision Medicine Project (KPMP) are therefore pivotal not only because they provide atlases but also because they establish the recruitment, biopsy‐based multi‐omics interrogation, and centralized data harmonization needed for future longitudinal integration at scale. The KPMP is explicitly designed to obtain and evaluate human kidney biopsies in CKD/AKI patients, define disease subgroups, and construct a kidney tissue atlas supported by an integrated coordinating infrastructure, providing foundations that can be extended from “static maps” toward longitudinal, feedback‐enabled systems once serial sampling and follow‐up layers are incorporated. Taken together, these developments suggest a practical roadmap for intelligence‐enabled kidney care: serial multi‐omics defines response modules; validated pharmacodynamic biomarkers quantify on‐target pathway engagement; and AI models integrate trajectories across omics and EHR streams to support adaptive dosing, class switching, or combination strategies—thereby closing the loop from molecular monitoring to actionable decision‐making [667].

### Section summary

**Figure 10:**
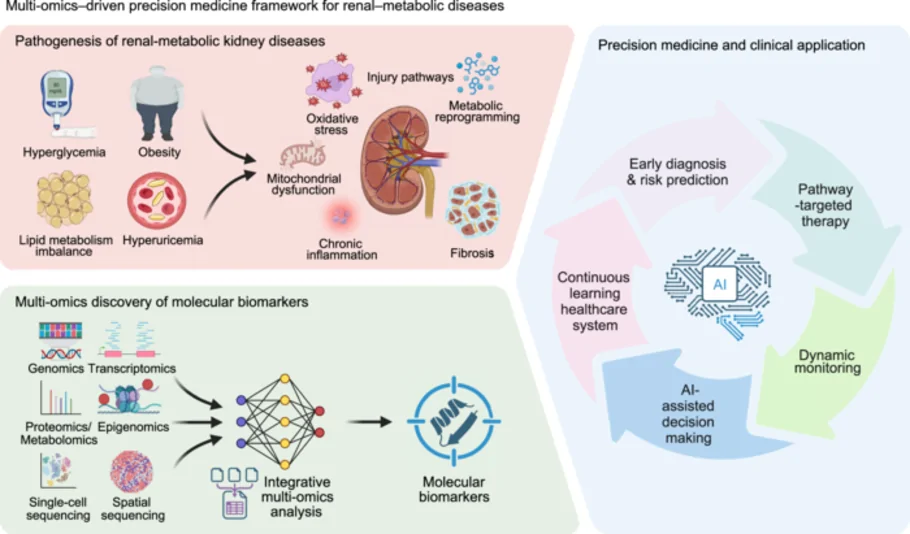
Multi‐omics approaches in precision therapy for renal–metabolic diseases. Metabolic disorders such as hyperglycemia, obesity, lipid imbalance, and hyperuricemia drive kidney injury through interconnected processes such as metabolic reprogramming, mitochondrial dysfunction, oxidative stress, chronic inflammation, and fibrosis. Multi‐omics technologies (genomics, transcriptomics, proteomics, metabolomics, epigenomics, and single‐cell sequencing) enable integrative analyses that identify molecular biomarkers and disease‐related pathways. These discoveries support precision medicine strategies, where biomarker‐guided early diagnosis and risk prediction inform pathway‐targeted therapies. The continuous monitoring of molecular and clinical indicators, combined with artificial intelligence–assisted decision‐making, enables a dynamic, learning healthcare system to optimize treatment for patients with renal–metabolic diseases.

Taken together, multi‐omics research in renal–metabolic diseases provides a clinically relevant framework that extends from molecular discovery to precision disease management. In diabetic kidney disease, obesity/metabolic syndrome–associated CKD, and hyperuricemia‐related nephropathy, similar clinical phenotypes may arise from distinct molecular mechanisms, including metabolic dysfunction, inflammation, mitochondrial injury, endothelial damage, and fibrosis. Multi‐omics can therefore support early molecular detection, mechanism‐based risk stratification, pathway‐guided therapeutic selection, and longitudinal response monitoring. When integrated with AI and real‐world clinical data, this framework may further enable adaptive decision‐making and continuously optimized precision nephrology (Figure 10).

## CHALLENGES AND FUTURE DIRECTIONS OF MULTI‐OMICS‐DRIVEN PRECISION MEDICINE

Therapeutic practice has historically adhered to a universal paradigm [680, 681], despite evolving conceptual frameworks across biomedicine, philosophy, and sociology [682, 683, 684, 685]. This traditional paradigm, which is based on imprecise diagnosis and generalized treatments, is facing increasing challenges [681], particularly given the increasing mortality caused by increasingly subclassified chronic diseases [686].

The 1999 WHO diabetes classification marked the beginning of this paradigm shift [687]. Subsequent subclassifications and evolving treatment guidelines have further highlighted that clinically similar diseases may arise from diverse etiologies and therefore require fundamentally different medical interventions [688, 689]. Omics technologies underpin these advances in clarifying disease and treatment mechanisms at the molecular level. Today, empowered by rapidly advancing AI, the integration of multi‐omics is laying solid groundwork for unraveling disease pathogenesis and enabling precision medicine.

**Figure 11:**
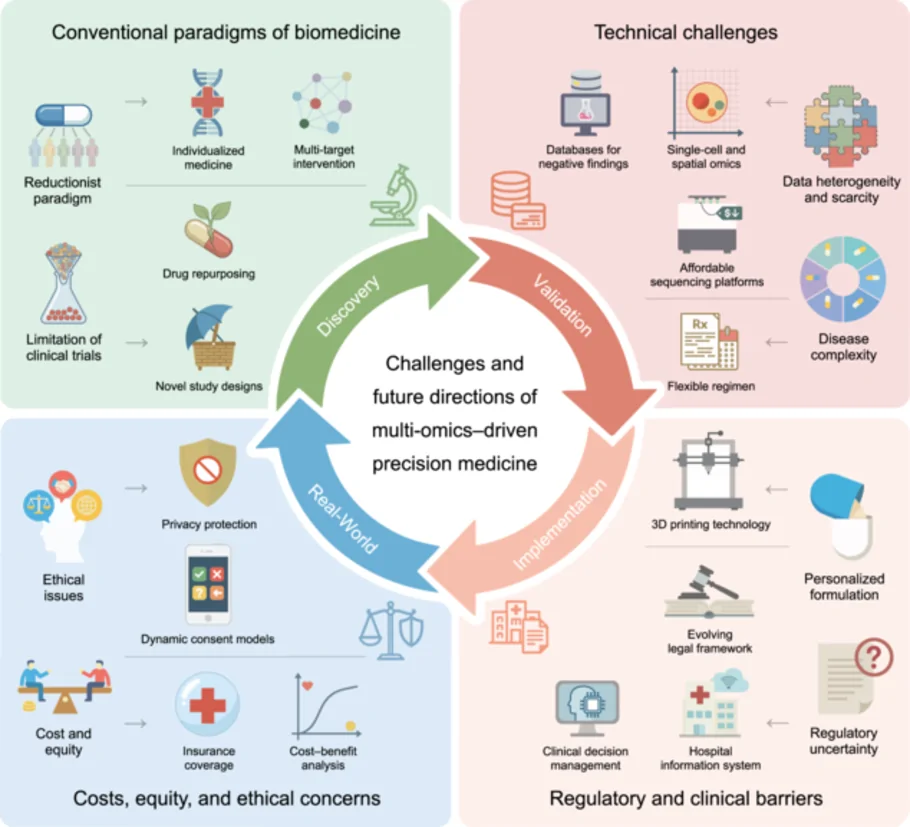
Challenges and future directions of multi‐omics‐driven precision medicine. Despite substantial achievements and considerable potential, the widespread clinical application of multi‐omics‐driven precision medicine remains limited by several obstacles. These obstacles stem from limitations inherent in conventional drug development and clinical trial paradigms, as well as technical challenges such as data heterogeneity and scarcity and the inherent complexity of polygenic diseases. Its clinical translation is further complicated by regulatory and legal barriers, intellectual property issues, the governance of AI‐assisted decision‐making, and individualized pharmaceutical preparation. Moreover, equity concerns arising from high costs, alongside pressing demands for updated ethical frameworks, particularly regarding privacy, data ownership, and informed consent, add further layers of complexity.

Nonetheless, despite considerable achievements in MODPM, significant technical, clinical, economic, and ethical barriers continue to obstruct its translation into routine practice (Figure 11).

### Paradigm shifts catalyzed by multi‐omics‐driven precision medicine

Multi‐omics studies have achieved substantial progress in precision medicine across diverse diseases, particularly in dose personalization and targeted treatment. For instance, warfarin, an oral anticoagulant with a narrow therapeutic window and pronounced pharmacokinetic variability, necessitates long‐term therapeutic drug monitoring [690, 691]. Insights from multi‐omics have revealed the underlying molecular mechanisms of this heterogeneity, leading to the development of user‐friendly testing kits—advances that have demonstrably enhanced therapeutic efficacy and safety profiles [692, 693]. However, optimizing specific therapies can only partially advance the goal of precision medicine. The increasing complexity of clinical needs requires a fundamental re‐evaluation of conventional drug development frameworks. Multi‐omics approaches not only identify existing limitations but also actively promote paradigm shifts across drug discovery, clinical trial design, and translational feedback loops.

Despite its name, the traditional paradigm of “rational” drug design remains an evolving construct that requires continuous refinement. Multi‐omics reveals that diseases frequently involve dysregulation across multiple biological pathways [694]. Furthermore, it shows that pathogenesis‐related proteins exist as ensembles of microconformations, which may exhibit various responses to a given ligand [695]. This systems‐level complexity highlights the heterogeneity of patient populations and challenges the prevailing reductionist paradigm. Consequently, therapeutic strategies are increasingly shifting from “perfect target blockade” toward modulating disease‐associated networks. The success of sorafenib, a multikinase inhibitor, reaffirms this principle, as its therapeutic effects are partly derived from the inhibition of multiple targets [696]. This network perspective may also explain the *in vivo* inefficacy of certain lead compounds with high *in vitro* target affinity. Moreover, off‐target effects play a considerable role in the therapeutic activity of some ostensibly selective agents. For instance, the clinical effects of imatinib extend beyond *BCR‐ABL* inhibition, involving additional targets and mechanisms [697]. From a safety standpoint, accumulating evidence challenges the assumption that single‐target agents are intrinsically safer or more effective than multitarget drugs [698]. Paradoxically, excessively high affinity for a single target may narrow the therapeutic window and heighten the risk of acquired resistance [699]. In summary, multi‐omics provides in‐depth mechanistic insight into disease pathophysiology and facilitates the integration of clinical evidence, multitarget strategies, and combination regimens into the drug development continuum, thereby strengthening the scientific foundation for precision medicine.

Randomized controlled trials, long regarded as the gold standard for evaluating drug efficacy and safety, are undergoing critical reexamination even as they benefit from multi‐omics integration. Historically, symptom‐based disease classifications have often led to the inadvertent inclusion of molecularly heterogeneous nonresponders to treatments in large trial cohorts. Such variability may dilute treatment effects and obscure the benefit of interventions targeted at specific disease subtypes. An example is everolimus, an mTOR inhibitor initially evaluated in an unselected cohort of patients with metastatic urothelial cancer. Although it demonstrated no superiority over placebo, a subset of patients exhibited responses, including one case with durable remission [700]. Subsequent multi‐omics analyses linked these responses to *TSC1* mutations and dysfunction of the corresponding protein [701]. This insight, underscoring the value of identifying responsive subpopulations, partially contributed to the approval of everolimus for various cancers irrespective of *TSC1* status. Without multi‐omics studies of exceptional responders, a potentially valuable therapy for a molecularly defined bladder cancer subset might have been prematurely discarded. This case underscores the imperative need for biomarker refinement, patient stratification, and in‐depth exceptional responder analyses in clinical trials [702, 703]. Emerging trial methods, such as crossover, adaptive, basket, and N‐of‐1 approaches [703], offer statistical efficiency with smaller sample sizes and align with this evolving framework.

Furthermore, multi‐omics may rescue compounds from discontinuation because of failure in conventional clinical trials. Teplizumab, an anti‐CD3 monoclonal antibody, failed to meet its primary endpoints in a phase Ⅲ trial for type 1 diabetes, with no significant reduction in insulin requirements or glycated hemoglobin levels [704]. However, subsequent multi‐omics analyses revealed the sustained modulation of immune pathways and long‐term preservation of C‐peptide levels—a surrogate marker of islet β‐cell function. These insights, together with those of confirmatory trials, supported the eventual approval of teplizumab for delaying the progression of type 1 diabetes [705]. This cycle, from clinical variability to omics study and back to a refined indication, epitomizes the iterative optimization of the multi‐omics–guided paradigm. This approach offers a cost‐effective strategy to accelerate drug development and address persistent unmet medical needs.

### Technical challenges of multi‐omics‐driven precision medicine

Despite the remarkable achievements and considerable potential of MODPM, its widespread clinical implementation remains constrained by technological challenges, especially the heterogeneity and scarcity of multi‐omics data and the complexity of treating polygenic diseases.

#### Data heterogeneity and scarcity

Multi‐omics‐driven precision medicine is substantially constrained by heterogeneous and scarce data, leading to statistical barriers and results with limited applicability [706, 707]. Overcoming these obstacles is essential for improving reproducibility, generalizability, and clinical implementation.

Data heterogeneity, a primary challenge, frequently originates from diverse platforms and laboratories using varied instruments, protocols, and workflows. Such technical variability, often manifesting as batch effects, may introduce noise and lead to the curse of dimensionality and overfitting [360]. This may diminish statistical power, undermine data reproducibility and cross‐study comparability, and even result in misleading conclusions [203, 706]. In a clinical trial, inconsistent transcriptomic protocols misclassified the risk status of 162 cancer patients, of whom 28 received unnecessary chemotherapy [708]. This underscores the need to establish unified standard operating procedures, good laboratory practices, and statistical methodologies in multi‐omics research to ensure data precision and reproducibility.

Another limitation lies in the scarcity of available multi‐omics data sources. High‐quality, multidimensional data sets are scarce and are largely generated within a limited number of prominent research institutions or clinical centers, thereby introducing sampling bias, such as the overrepresentation of Caucasian populations. This lack of diversity compromises the validity of findings and limits their broader applicability [50]. Furthermore, despite the identification of numerous biomarkers using multi‐omics approaches, only some have been rigorously validated, particularly regarding disease causality and specificity. Such a lack of rigorous validation may compromise their reliability for clinical application [7].

To increase the robustness of conclusions given these limitations, the application of multi‐omics data in clinical practice must be interpreted alongside real‐world evidence. The integration of multi‐omics data with deep phenotypic data from standardized electronic health records is crucial for identifying gene–environment interactions. However, effective integration strategies remain an open question [50, 709, 710].

#### Complexity of polygenic disease

Multi‐omics‐driven precision medicine can yield modest benefits in polygenic diseases or in cases without targetable alterations. Its clinical application, however, is constrained by patient tolerability, drug resistance due to incomplete treatment, and the reductionist interpretation of the “precision” concept.

The pathophysiological architecture of many diseases defies simple explanation: They may result from the cumulative effect of multiple mutations rather than a single alteration, or, in some cases, they exhibit no clearly identifiable driver at all. For instance, tumor genomics has revealed the frequent coexistence of multiple mutations within a single tumor [711], posing clinical dilemmas regarding target selection. Moreover, the systematic underreporting of unsuccessful precision therapy cases obscures valuable negative evidence, hindering the identification of optimal intervention targets despite the availability of extensive multi‐omics data. In parallel, efforts should be made to promote the inclusion of negative cases in databases and peer‐reviewed literature [712].

In treating polygenic disease, patient tolerability to medications also imposes critical constraints, demanding well‐designed regimens even for combinations of highly selective targeted agents. A clinical trial compared half‐dose dual‐targeted therapy with conventional chemotherapy in patients whose advanced solid tumors contained at least two actionable targets [713]. Although the combination group achieved longer overall survival, this benefit was associated with a significantly higher incidence of adverse events [713]. More critically, incomplete precision therapy that targets only a subset of mutations may exert selective pressure within the disease microenvironment. This may induce resistance in residual cells and, under cytokine stimulation, potentially increase their survival and proliferation [711, 714]. In oncology, such consequences can be fatal. Quinidine is another typical example, a drug that shows highly variable clinical responses in precision therapy for Dravet syndrome [715], whereas phenytoin, despite its pharmacological contraindications, has shown unexpected efficacy and tolerability in some patients [716]. These observations highlight that current precision medicine may not always outperform nonprecision approaches. Furthermore, therapeutic decisions must integrate molecular patterns with clinical realities rather than mechanically subordinate real‐world evidence to dogmatism.

“Precision” is not a binary concept, nor is precision medicine merely molecular targeting. Instead, it represents a dynamic, patient‐centered, and evolving endeavor. Consequently, therapeutic individualization demands multidimensional calibrations of pathophysiological conditions, treatment tolerability, patient preferences, and clinical evidence. Multi‐omics‐driven precision therapies encompass not only drug selection, dosage, combination, and routes of administration but also surgery, alternative treatments, and well‐defined therapeutic purposes (e.g., cure, symptom control, or quality‐of‐life improvement). Such a comprehensive perspective facilitates the clinical translation of precision medicine, delivering meaningful benefits to patients in need.

#### Facilitating the clinical adoption of omics via technological advancements

Despite the aforementioned challenges, recent technological advancements are actively bridging the gap between multi‐omics research and routine clinical practice. Driven by advances in efficiency, reliability, and automation, particularly microfluidic technologies [717], single‐cell and spatial multi‐omics have emerged as accessible tools suitable for clinical use. These platforms enable the dissection of cellular heterogeneity and spatial organization within diseased tissues, revealing rare but clinically actionable clones or microenvironments that might be masked by bulk assays in complex diseases. By extracting high‐resolution biological information from limited clinical samples, they may overcome the data scarcity inherent in many clinical settings [48].

Parallel advances in analytical and computational technologies address another translational bottleneck—the prioritization of clinically relevant alterations from massive multi‐omics data sets. Hierarchical frameworks based on established actionability, such as the European Society for Medical Oncology Scale for Clinical Actionability of Molecular Targets (ESCAT), have been developed. This system stratifies genomic alterations from tier Ⅰ (eligible for clinical intervention) to tier Ⅴ (no clinical benefit) and tier Ⅹ (insufficient evidence for clinical actionability), serving as a decision‐making framework for researchers and clinicians regarding whether to pursue further investigation on a given alteration. Consequently, unnecessary expenditures of time and financial resources are prevented, thereby promoting the clinical translation of multi‐omics technologies [718].

Additional technological enablers include (1) advancing networking and cloud computing, which permits complex multi‐omics data analysis without reliance on dedicated local computational infrastructure; (2) standardized data management workflows that integrate batch‐effect correction algorithms (e.g., ComBat) into routine laboratory systems [719]; and (3) portable and affordable sequencing devices, such as nanopore platforms, that facilitate rapid onsite multi‐omics profiling [720].

Collectively, these technological advancements are reshaping the feasibility landscape for clinical multi‐omics. As costs continue to decrease, workflows become automated, and interpretative frameworks mature, the transition from research‐only applications to routine diagnostic tools is accelerating. Nevertheless, the successful clinical adoption of MODPM requires concurrent investments in regulatory pathways, the training ‌of medical personnel‌, and precision pharmaceutical formulation, which extend beyond purely technological considerations but are equally essential.

### Regulatory, operational, and manufacturing barriers to multi‐omics‐driven precision medicine

Translating MODPM from bench to bedside requires not only technological breakthroughs but also the deliberate redesign of clinical workflows, regulatory pathways, and drug manufacturing systems—each of which presents critical barriers discussed below.

To address these barriers in a clinical context, consider a typical hospital attempting to implement MODPM for an oncology patient. After multi‐omics profiling and AI‐assisted dose optimization, the physician prescribes drugs in a combined dosage form at personalized doses. However, the electronic health records system lacks a module to record AI‐derived recommendations as auditable clinical decisions. The pharmacy dispensary system does not accept variable‐dose prescriptions. Compounding units cannot formulate qualified individualized preparations in time. Consequently, the physician could only revert to the conventional regimen, nullifying the precision approach. Compounding this challenge is the lack of clinical decision support tools that can translate multi‑omics results into actionable therapeutic recommendations, leaving clinicians without clear interpretation pathways. This scenario illustrates that the clinical integration of MODPM is not merely a technical obstacle but also a multidimensional challenge across regulatory policy, intellectual‐property management, workflow governance, and pharmaceutical manufacturing capacity. Bridging these gaps is therefore a prerequisite for enabling the clinical translation of MODPM.

Precision pharmaceutical preparations require regulatory support, especially explicit provisions for off‐label use and individualized formulation. Current regulatory frameworks, which predominantly approve drugs in standardized fixed‐dose forms, inherently limit such personalization. A further concern lies in the intellectual property domain: repurposing approved drugs for precision formulations risks patent litigation and financial disputes. This obstacle, often neglected in related research, can be insurmountable for MODPM. Furthermore, MODPM increasingly relies on AI assistance, which may introduce risks, potentially leading to false conclusions and harmful clinical decisions [721]. This raises critical concerns about responsibility, as there is currently no clear regulatory or legal framework for allocating liability among AI developers, testing laboratories, and prescribing physicians. It also poses challenges for the review of clinical decision‐making workflows, since any output intended for clinical use must undergo rigorous validation.

Moreover, compliance with good manufacturing practices does not guarantee full manufacturing qualifications. Conventional pharmacies and hospital compounding units may lack the capacity to independently execute the entire individualized pharmaceutical preparation workflow. This limitation is particularly evident in the precise formulation of complex dosage forms, such as combination products or modified‐release preparations. Rapidly advancing 3D printing technology offers a solution to this bottleneck, enabling the precise modulation of drug dose, release kinetics, drug combinations, and dosage forms according to individualized prescriptions. A clinical trial demonstrated the use of semisolid extrusion 3D printing to rapidly prepare fixed‐dose combination capsules with robust efficacy and prolonged storage stability [722]. Another recent study reported a substantial reduction in associated costs, facilitating the deployment of 3D printing in hospital pharmacies [723].

### Cost and ethical concerns of multi‐omics‐driven precision medicine

Beyond regulatory and operational hurdles, the clinical translation of MODPM is equally shaped by cost–benefit realities and ethical frameworks, which together determine whether personalized interventions can reach patients equitably and sustainably.

Cost is a major barrier to the widespread adoption of MODPM. At present, multi‐omics technologies remain expensive and time‐consuming, coupled with the need for expert interpretation and patient compliance. These limitations restrict their application to a small subset of patients with specific diseases. This reality raises critical concerns: Wealth inequality has already resulted in unequal access to precision medicine [724], and the overrepresentation of certain ancestries in multi‐omics data sets may prevent non‐Caucasian populations from achieving equal benefits [50].

A systematic comparison of costs and benefits across different stakeholder perspectives further clarifies this potential. From a payer perspective (e.g., insurance or national health system), upfront multi‐omics testing and AI analysis may increase short‐term expenditure, but reduced trial‐and‐error prescribing, fewer adverse drug events, and shortened hospital stays can offset these costs—especially in high‐cost contexts such as cancer and rare diseases. From a patient perspective, although out‐of‐pocket costs may increase if tests remain uninsured, benefits include faster access to effective therapy, the avoidance of ineffective treatments, and potentially improved quality of life. From a health system perspective, the paradigm shift from “try‐until‐response” to “right‐first‐time” could reallocate resources from managing treatment failures to delivering more reliable health care. Quantitatively, although the multi‐omics diagnostic cost for melanoma was reported to be fivefold greater than that of routine sequencing [725], the overall medical cost may remain comparable because of reduced trial‐and‐error and improved efficacy [725, 726]. For example, multi‑omics‑guided therapy was associated with a substantially increased response rate and a significant extension of progression‐free survival. Thus, when the upfront costs of multi‐omics testing are higher, the benefit–cost balance may become neutral or positive when downstream savings are accounted for. However, it should be noted that this favorable balance is most evident in oncology and rare monogenic diseases for which effective targeted therapies exist. For common polygenic conditions (e.g., hypertension), the cost‐effectiveness of MODPM remains uncertain and requires disease‐specific health technology assessment. Accordingly, extending insurance reimbursement to cover these tests and promoting public awareness of their value may serve as pivotal measures to mitigate accessibility disparities. Health technology assessment frameworks, such as the UK's quality‐adjusted life year‐based model and China's centralized procurement policies, may improve the cost‐effectiveness and accessibility of these tests.

Innovations in single‐cell omics are advancing equity in precision medicine: Optimized CITE‐seq antibody titration can improve assay efficiency and reduce reagent waste [727], and the International Human Cell Atlas Consortium is building a comprehensive, multiorigin reference map [728]. AI‐driven multi‐omics simulations also hold promise for addressing these challenges. The integration of AI‐powered multi‐omics platforms, along with emerging organ‐on‐a‐chip techniques and metagenomics studies [729, 730], can facilitate the generation of high‐fidelity, context‐specific data sets, paving the way toward digital twins from the cell to the population scale [50]. Parallel efforts to develop standardized kits for specific omics applications also hold promise for reducing costs and lowering the entry barrier.

Beyond equity and equality, the primary ethical concern of MODPM is privacy, a challenge that requires advances in privacy tools, dynamic consent models, and evolving ethical frameworks. The complexity of multi‐omics data undermines the effectiveness of conventional anonymization measures. The removal of explicit identifiers, for instance, may not guarantee privacy, as transcriptomic profiles remain susceptible to reidentification via eQTLs, enabling linkage attacks [731]. Proteomic data carry similar risks: single amino acid variants in hair proteins have been shown to enable individual identification [732]. Long‐term clinical data storage further amplifies privacy risks. Given that multi‐omics data contain sensitive information on disease susceptibility, drug response, and heritable traits, any leakage may affect not only individuals but also their biological relatives. Cases in which genomic data are transferred to pharmaceutical companies without informed consent have already been documented, and high‐risk genotype carriers may even face insurance denial [733].

Another longstanding controversy surrounds the ownership of multi‐omics data. In practice, data governance is frequently dominated by custodians, leaving data subjects unable to track data use or demand erasure under the right to be forgotten [734]. Blockchain technology offers an innovative solution to these concerns. By providing immutable metadata ensures transparent and traceable privacy protection for sensitive omics information [735]. The GenShare platform exemplifies this approach, integrating two distinct blockchains with homomorphic and hardware‐based encryption to facilitate the secure sharing and statistical analysis of genomic data while safeguarding individual privacy [736]. A more advanced example is Governome, which uses a blockchain‐based management system, allowing data owners to dynamically authorize, instantly revoke, and comprehensively track the use of their omics data [734].

Furthermore, the scale and inherent uncertainty of multi‐omics data also challenge the conventional framework of informed consent. Patients may not fully understand the significance and future implications of omics information, and this lack undermines their capacity for autonomous decision‐making [737]. The emerging dynamic consent model provides a potential solution to this challenge, yet it may also lead to unintended consequences regarding future treatments or the disclosure of incidental findings unrelated to the original condition [738]. Moreover, the volume and complexity of multi‐omics data may exceed the capacity of conventional ethics review processes to conduct timely evaluations. Delaying the resolution of these privacy and consent issues may directly slow the clinical translation of MODPM, as institutional review boards and hospital ethics committees currently lack standardized protocols for multi‐omics studies, creating a critical implementation bottleneck.

## CONCLUSION

Multi‐omics‐driven precision medicine has moved beyond a conceptual ambition and is now reshaping how disease mechanisms, patient stratification, therapeutic targeting, and treatment monitoring are understood across multiple clinical domains. Its true value lies not in the accumulation of more data layers but in the integration of those layers into a coherent framework that connects molecular heterogeneity to interpretable and actionable clinical decisions. Across drug development, oncology, autoimmune disease, cardiometabolic disease, and renal–metabolic disease, the common theme is the same: Multi‐omics becomes most powerful when it is linked to mechanism, context, and outcome rather than treated as a purely descriptive technology.

The next phase of MODPM will depend on several converging advances: higher‐quality and more diverse multimodal cohorts, stronger standards for data generation and external validation, more interpretable and biologically faithful AI models, and clinical workflows that can incorporate longitudinal learning without compromising safety, equity, or privacy. If these challenges are met, MODPM will not simply refine existing precision medicine paradigms but will help establish a more adaptive, mechanistic, and continuously learning model of medicine.

## AUTHOR CONTRIBUTIONS

**Huibo Li:** Conceptualization; supervision; writing—original draft; writing—review and editing; visualization; project administration; funding acquisition. **Zhe Zhao:** Writing—original draft; writing—review and editing; visualization; funding acquisition. **Yifan Zhang:** Writing—original draft; writing—review and editing; visualization. **Xin Zhou:** Writing—original draft; writing—review and editing; visualization. **Gaofei Hu:** Writing—original draft; writing—review and editing; visualization. **Min Zeng:** Writing—original draft; writing—review and editing; visualization. **Zhanqun Yang:** Writing—original draft; writing—review and editing; visualization. **Zixuan Zhao:** Writing—original draft; writing—review and editing; visualization. **Yao Ma:** Writing—original draft; writing—review and editing; visualization. **Wei Hu:** Writing—original draft; writing—review and editing; visualization. **Yuxuan Sun:** Writing—original draft; writing—review and editing; visualization. **Meng Su:** Writing—original draft; writing—review and editing; visualization. **Jun Li:** Writing—review and editing; visualization; funding acquisition. **Matthew Whiteman:** Writing—original draft; visualization. **Wei Fu:** Conceptualization; writing—original draft; writing—review and editing. **Chao Zhong:** Conceptualization; writing—original draft; writing—review and editing; funding acquisition. **Lemin Zheng:** Conceptualization; writing—original draft; writing—review and editing; funding acquisition. **Long Chen:** Conceptualization; writing—original draft; writing—review and editing; funding acquisition. **Hairong Lv:** Conceptualization; writing—original draft; writing—review and editing; visualization; supervision; funding acquisition. **Rongsheng Zhao:** Conceptualization; writing—original draft; writing—review and editing; funding acquisition. **Yi Zhun Zhu:** Conceptualization; supervision; writing—original draft; writing—review and editing; funding acquisition. All authors have read the final manuscript and approved it for publication.

## CONFLICT OF INTEREST STATEMENT

The authors declare no conflicts of interest.

## ETHICS STATEMENT

No animals or humans were involved in this study.

## FIGURE GENERATION STATEMENT

All figures in this manuscript were designed, revised, and finalized by the authors using BioRender.com, Microsoft PowerPoint, and AI‐assisted visualization tools where applicable. The authors reviewed and edited all the figure contents to ensure scientific accuracy. No copyrighted third‐party images were reproduced without permission.

## Data Availability

Data sharing is not applicable to this article as no new data were created or analyzed in this study. Supplementary materials (graphical abstract, slides, videos, Chinese translated version and update materials) may be found in the online DOI or iMeta Science http://www.imeta.science/.

## References

[1] National Human Genome Research Institute . 2026. Precision Medicine, March 5, 2026. https://www.genome.gov/genetics-glossary/Precision-Medicine

[2] Wang, Wen‐jian , and Teng Zhang . 2017. “Integration of Traditional Chinese Medicine and Western Medicine in the Era of Precision Medicine.” Journal of Integrative Medicine 15: 1–7. 10.1016/S2095-4964(17)60314-5 28088253

[3] Moeini, Reihaneh , Zahra Memariani , Parvin Pasalar , and Narjes Gorji . 2017. “Historical Root of Precision Medicine: An Ancient Concept Concordant With the Modern Pharmacotherapy.” DARU Journal of Pharmaceutical Sciences 25: 7. 10.1186/s40199-017-0173-1 28372571 PMC5379719

[4] Obama, Barack . 2026, Remarks by the President on Precision Medicine, March 5, 2026, whitehouse.gov., https://obamawhitehouse.archives.gov/the-press-office/2015/01/30/remarks-president-precision-medicine

[5] Ramaswami, Ramya , Ronald Bayer , and Sandro Galea . 2018. “Precision Medicine From a Public Health Perspective.” Annual Review of Public Health 39: 153–168. 10.1146/annurev-publhealth-040617-014158 29166244

[6] Le, Jiayuan , Yating Dian , Deze Zhao , Ziyu Guo , Zehao Luo , Xiang Chen , Furong Zeng , and Guangtong Deng . 2025. “Single‐Cell Multi‐Omics in Cancer Immunotherapy: From Tumor Heterogeneity to Personalized Precision Treatment.” Molecular Cancer 24: 221. 10.1186/s12943-025-02426-3 40855431 PMC12376342

[7] Jiang, Ziming , Haoxuan Zhang , Yibo Gao , and Yingli Sun . 2025. “Multi‐Omics Strategies for Biomarker Discovery and Application in Personalized Oncology.” Molecular Biomedicine 6: 115. 10.1186/s43556-025-00340-0 41269529 PMC12638490

[8] Kreitmaier, Peter , Georgia Katsoula , and Eleftheria Zeggini . 2023. “Insights From Multi‐Omics Integration in Complex Disease Primary Tissues.” Trends in Genetics 39: 46–58. 10.1016/j.tig.2022.08.005 36137835

[9] Hsu, Chou‐Yi , Shavan Askar , Samer Saleem Alshkarchy , Priya Priyadarshini Nayak , Kassem A. L. Attabi , Mohammad Ahmar Khan , J. Albert Mayan , et al. 2025. “AI‐driven Multi‐Omics Integration in Precision Oncology: Bridging the Data Deluge to Clinical Decisions.” Clinical and Experimental Medicine 26: 29. 10.1007/s10238-025-01965-9 41266662 PMC12634751

[10] Guan, Angel , and Camelia Quek . 2025. “Single‐Cell Multi‐Omics: Insights Into Therapeutic Innovations to Advance Treatment in Cancer.” International Journal of Molecular Sciences 26: 2447. 10.3390/ijms26062447 40141092 PMC11942442

[11] Hasin, Yehudit , Marcus Seldin , and Aldons Lusis . 2017. “Multi‐Omics Approaches to Disease.” Genome Biology 18: 83. 10.1186/s13059-017-1215-1 28476144 PMC5418815

[12] Mani, Srinivasan , Seema R. Lalani , and Mohan Pammi . 2025. “Genomics and Multiomics in the Age of Precision Medicine.” Pediatric Research 97: 1399–1410. 10.1038/s41390-025-04021-0 40185865 PMC12106083

[13] Luo, Yan , Nan Zhang , Jiannan Yang , Mengyao Cui , Kelvin K. F. Tsoi , Gregory Y. H. Lip , Tong Liu , and Qingpeng Zhang . 2026. “AI‐Based Multiomics Profiling Reveals Complementary Omics Contributions to Personalized Prediction of Cardiovascular Disease.” Nature Communications 17: 2269. 10.1038/s41467-026-68956-6 PMC12966374 41629312

[14] Camps, Jordi , Andrea Jiménez‐Franco , Raquel García‐Pablo , Jorge Joven , and Meritxell Arenas . 2025. “Artificial Intelligence‐Driven Integration of Multi‐Omics and Radiomics: A New Hope for Precision Cancer Diagnosis and Prognosis.” Biochimica Et Biophysica Acta—Molecular Basis of Disease 1871: 167841. 10.1016/j.bbadis.2025.167841 40216368

[15] Xu, Yu , Scott C. Ritchie , Yujian Liang , Paul R. H. J. Timmers , Maik Pietzner , Loïc Lannelongue , Samuel A. Lambert , et al. 2023. “An Atlas of Genetic Scores to Predict Multi‐Omic Traits.” Nature 616: 123–131. 10.1038/s41586-023-05844-9 36991119 PMC10323211

[16] Julkunen, Heli , Anna Cichońska , Mika Tiainen , Harri Koskela , Kristian Nybo , Valtteri Mäkelä , Jussi Nokso‐Koivisto , et al. 2023. “Atlas of Plasma NMR Biomarkers for Health and Disease in 118,461 Individuals From the UK Biobank.” Nature Communications 14: 604. 10.1038/s41467-023-36231-7 PMC9898515 36737450

[17] Teleanu, Maria‐Veronica , Annika Schneider , Claudia R. Ball , Mathias Felix Leber , Christoph Stange , Eva Krieghoff‐Henning , Katja Beck , et al. 2025. “Celebrating Ulrik Ringborg: Multi‐Omics‐Based Patient Stratification for Precision Cancer Treatment.” Biomolecules 15: 693. 10.3390/biom15050693 40427586 PMC12108785

[18] Chen, Chongyang , Jing Wang , Donghui Pan , Xinyu Wang , Yuping Xu , Junjie Yan , Lizhen Wang , et al. 2023. “Applications of Multi‐Omics Analysis in Human Diseases.” MedComm 4: e315. 10.1002/mco2.315 37533767 PMC10390758

[19] Chen, Hang , Ying Shi , Meiling Yuan , Huihui Li , and Xiaowei Liu . 2026. “Integrating Multi‐Omics and Artificial Intelligence Fuels Advanced Target Identification and Drug Discovery.” Biotechnology Advances 87: 108785. 10.1016/j.biotechadv.2025.108785 41461245

[20] Jameson, J. Larry , and Dan L. Longo . 2015. “Precision Medicine‐‐Personalized, Problematic, and Promising.” New England Journal of Medicine 372: 2229–2234. 10.1056/NEJMsb1503104 26014593

[21] Ugochukwu, Ezinwanne Jane , Jennifer Chinwe Edom , Faith Olanrewaju Omotayo , Agatha Adaeze Amaechi , Chinedu Benneth Obetta , Chibueze Anosike , AbdulMuminu Isah , and Chukwuemeka Michael Ubaka . 2025. “Bridging the Gap: Understanding the Perspective of Healthcare Professional Students Towards Precision Medicine in a Nigerian Tertiary Institution (A Cross‐Sectional Study).” BMC Medical Education 25: 63. 10.1186/s12909-025-06651-8 39806360 PMC11730482

[22] Huang, Haiyin , Peilan Yang , Jingjing Xue , Jie Tang , Liyu Ding , Ying Ma , Jie Wang , et al. 2014. “Evaluating the Individualized Treatment of Traditional Chinese Medicine: A Pilot Study of N‐of‐1 Trials.” Evidence‐Based Complementary and Alternative Medicine 2014: 148730. 10.1155/2014/148730 25477988 PMC4244929

[23] Hamamoto, Ryuji , Takafumi Koyama , Nobuji Kouno , Tomohiro Yasuda , Shuntaro Yui , Kazuki Sudo , Makoto Hirata , et al. 2022. “Introducing AI to the Molecular Tumor Board: One Direction Toward the Establishment of Precision Medicine Using Large‐Scale Cancer Clinical and Biological Information.” Experimental Hematology & Oncology 11: 82. 10.1186/s40164-022-00333-7 36316731 PMC9620610

[24] Collins, Francis S. , and Harold Varmus . 2015. “A New Initiative on Precision Medicine.” New England Journal of Medicine 372: 793–795. 10.1056/NEJMp1500523 25635347 PMC5101938

[25] Yang, Tiantian , and Zhiqian Chen . 2026. “MOTGNN: Interpretable Graph Neural Networks for Multi‐Omics Disease Classification.” arXiv. 10.48550/arXiv.2508.07465

[26] Karczewski, Konrad J. , and Michael P. Snyder . 2018. “Integrative Omics for Health and Disease.” Nature Reviews Genetics 19: 299–310. 10.1038/nrg.2018.4 PMC5990367 29479082

[27] Liu, Yi , Benjamin Elsworth , Pau Erola , Valeriia Haberland , Gibran Hemani , Matt Lyon , Jie Zheng , et al. 2021. “EpiGraphDB: A Database and Data Mining Platform for Health Data Science.” Bioinformatics 37: 1304–1311. 10.1093/bioinformatics/btaa961 33165574 PMC8189674

[28] Ahmed, Zeeshan , Khalid Mohamed , Saman Zeeshan , and XinQi Dong . 2020. “Artificial Intelligence With Multi‐Functional Machine Learning Platform Development for Better Healthcare and Precision Medicine.” Database 2020: baaa010. 10.1093/database/baaa010 32185396 PMC7078068

[29] Matboli, Marwa , Shaimaa H. Gadallah , Wafaa M. Rashed , Amany Helmy Hasanin , Nada Essawy , Hala M. Ghanem , and Sanaa Eissa . 2021. “mRNA‐miRNA‐lncRNA Regulatory Network in Nonalcoholic Fatty Liver Disease.” International Journal of Molecular Sciences 22: 6770. 10.3390/ijms22136770 34202571 PMC8269036

[30] Williams, Cameron G. , Hyun Jae Lee , Takahiro Asatsuma , Roser Vento‐Tormo , and Ashraful Haque . 2022. “An Introduction to Spatial Transcriptomics for Biomedical Research.” Genome Medicine 14: 68. 10.1186/s13073-022-01075-1 35761361 PMC9238181

[31] Huang, Bowen , Yingjia Chen , and Shuqiang Yuan . 2024. “Application of Spatial Transcriptomics in Digestive System Tumors.” Biomolecules 15: 21. 10.3390/biom15010021 39858416 PMC11761220

[32] Tiwari, Ashutosh , Dyah Ika Widodo , Dyah Ika Krisnawati , Kai‐Yi Tzou , and Tsung‐Rong Kuo . 2026. “Machine Learning for Extracellular Vesicles Enables Diagnostic and Therapeutic Nanobiotechnology.” Journal of Nanobiotechnology 24: 153. 10.1186/s12951-025-03952-4 41546050 PMC12895957

[33] Hong, Chuan , Liang Liang , Qianyu Yuan , Kelly Cho , Katherine P. Liao , Michael J. Pencina , David C. Christiani , and Tianxi Cai . 2023. “Semi‐Supervised Calibration of Noisy Event Risk (SCANER) With Electronic Health Records.” Journal of Biomedical Informatics 144: 104425. 10.1016/j.jbi.2023.104425 37331495 PMC10478159

[34] Hübschmann, Daniel , Simon Kreutzfeldt , Benjamin Roth , Katrin Glocker , Janine Schoop , Lena Oeser , Steffen Hausmann , et al. 2026. “The Knowledge Connector Decision Support System for Multiomics‐Based Precision Oncology.” Nature Communications 17: 742. 10.1038/s41467-026-68333-3 PMC12820158 41554728

[35] Fu, Jia , Mengjie Fang , Ling Wu , Xiaodong Li , Francesco De Cobelli , Diego Palumbo , Xuebin Xie , et al. 2025. “Development, Advancement, and Clinical Integration of Artificial Intelligence Technology in Gastric Cancer.” Chinese Medical Journal 138: 3332–3350. 10.1097/cm9.0000000000003922 41400327 PMC12721780

[36] Gan, Xiangfeng , Jianzhong He , Wei Zhang , Wenzeng Chen , Shijiancong Liu , Wenhao Li , Xiaohui Duan , et al. 2025. “Attention‐Guided Framework for Integrative Omics and Temporal Dynamics in Predicting Major Pathological Response in Neoadjuvant Immunochemotherapy for NSCLC.” Journal for ImmunoTherapy of Cancer 13: e010541. 10.1136/jitc-2025-012526 41135954 PMC12557778

[37] Aronson, Samuel J. , and Heidi L. Rehm . 2015. “Building the Foundation for Genomics in Precision Medicine.” Nature 526: 336–342. 10.1038/nature15816 26469044 PMC5669797

[38] Perna, Giampaolo , Eleonora Pinto , Alessandro Spiti , Tatiana Torti , Michele Cucchi , and Daniela Caldirola . 2024. “Foundations for a Personalized Psycho‐Oncology: The State of the Art.” Journal of Personalized Medicine 14: 892. 10.3390/jpm14090892 39338146 PMC11433554

[39] Topol, Eric J . 2019. “High‐Performance Medicine: The Convergence of Human and Artificial Intelligence.” Nature Medicine 25: 44–56. 10.1038/s41591-018-0300-7 30617339

[40] Ji, Yanrong , Zhihan Zhou , Han Liu , and Ramana V. Davuluri . 2021. “DNABERT: Pre‐Trained Bidirectional Encoder Representations From Transformers Model for DNA‐Language in Genome.” Bioinformatics 37: 2112–2120. 10.1093/bioinformatics/btab083 33538820 PMC11025658

[41] Jumper, John , Richard Evans , Alexander Pritzel , Tim Green , Michael Figurnov , Olaf Ronneberger , Kathryn Tunyasuvunakool , et al. 2021. “Highly Accurate Protein Structure Prediction With AlphaFold.” Nature 596: 583–589. 10.1038/s41586-021-03819-2 34265844 PMC8371605

[42] Mehr, Ramit , Michal Sternberg‐Simon , Miri Michaeli , and Yishai Pickman . 2012. “Models and Methods for Analysis of Lymphocyte Repertoire Generation, Development, Selection and Evolution.” Immunology Letters 148: 11–22. 10.1016/j.imlet.2012.08.002 22902400

[43] Yu, Hengshi , Weizhou Qian , Yuxuan Song , and Joshua D. Welch . 2025. “Perturbnet Predicts Single‐Cell Responses to Unseen Chemical and Genetic Perturbations.” Molecular Systems Biology 21: 960–982. 10.1038/s44320-025-00131-3 40640612 PMC12322087

[44] Gao, Yicheng , Zhiting Wei , Kejing Dong , Ke Chen , Jingya Yang , Guohui Chuai , and Qi Liu . 2024. “Toward Subtask‐Decomposition‐Based Learning and Benchmarking for Predicting Genetic Perturbation Outcomes and Beyond.” Nature Computational Science 4: 773–785. 10.1038/s43588-024-00698-1 39333790

[45] He, Siyu , Yuefei Zhu , Daniel Naveed Tavakol , Haotian Ye , Yeh‐Hsing Lao , Zixian Zhu , Cong Xu , et al. 2026. “Squidiff: Predicting Cellular Development and Responses to Perturbations Using a Diffusion Model.” Nature Methods 23: 65–77. 10.1038/s41592-025-02877-y 41184550 PMC12872408

[46] Bunne, Charlotte , Yusuf Roohani , Yanay Rosen , Ankit Gupta , Xikun Zhang , Marcel Roed , Theo Alexandrov , et al. 2024. “How to Build the Virtual Cell With Artificial Intelligence: Priorities and Opportunities.” Cell 187: 7045–7063. 10.1016/j.cell.2024.11.015 39672099 PMC12148494

[47] Denny, Joshua C. , and Francis S. Collins . 2021. “Precision Medicine in 2030—Seven Ways to Transform Healthcare.” Cell 184: 1415–1419. 10.1016/j.cell.2021.01.015 33740447 PMC9616629

[48] Vandereyken, Katy , Alejandro Sifrim , Bernard Thienpont , and Thierry Voet . 2023. “Methods and Applications for Single‐Cell and Spatial Multi‐Omics.” Nature Reviews Genetics 24: 494–515. 10.1038/s41576-023-00580-2 PMC9979144 36864178

[49] Scannell, Jack W. , Alex Blanckley , Helen Boldon , and Brian Warrington . 2012. “Diagnosing the Decline in Pharmaceutical R&D Efficiency.” Nature Reviews Drug Discovery 11: 191–200. 10.1038/nrd3681 22378269

[50] Alemu, Robel , Nigussie T. Sharew , Yodit Y. Arsano , Muktar Ahmed , Fasil Tekola‐Ayele , Tesfaye B. Mersha , and Azmeraw T. Amare . 2025. “Multi‐Omics Approaches for Understanding Gene‐Environment Interactions in Noncommunicable Diseases: Techniques, Translation, and Equity Issues.” Human Genomics 19: 8. 10.1186/s40246-025-00718-9 39891174 PMC11786457

[51] Manolio, Teri A. , Rex L. Chisholm , Brad Ozenberger , Dan M. Roden , Marc S. Williams , Richard Wilson , David Bick , et al. 2013. “Implementing Genomic Medicine in the Clinic: The Future Is Here.” Genetics in Medicine 15: 258–267. 10.1038/gim.2012.157 23306799 PMC3835144

[52] Mardis, Elaine R . 2017. “DNA Sequencing Technologies: 2006–2016.” Nature Protocols 12: 213–218. 10.1038/nprot.2016.182 28055035

[53] Goodwin, Sara , John D. McPherson , and W. Richard McCombie . 2016. “Coming of Age: Ten Years of Next‐Generation Sequencing Technologies.” Nature Reviews Genetics 17: 333–351. 10.1038/nrg.2016.49 PMC10373632 27184599

[54] De Coster, Wouter , Matthias H. Weissensteiner , and Fritz J. Sedlazeck . 2021. “Towards Population‐Scale Long‐Read Sequencing.” Nature Reviews Genetics 22: 572–587. 10.1038/s41576-021-00367-3 PMC8161719 34050336

[55] Clark, Michael J. , Rui Chen , Hugo Y. K. Lam , Konrad J. Karczewski , Rong Chen , Ghia Euskirchen , Atul J. Butte , and Michael Snyder . 2011. “Performance Comparison of Exome DNA Sequencing Technologies.” Nature Biotechnology 29: 908–914. 10.1038/nbt.1975 PMC4127531 21947028

[56] Tsaousis, Georgios N. , Eirini Papadopoulou , Angela Apessos , Konstantinos Agiannitopoulos , Georgia Pepe , Stavroula Kampouri , Nikolaos Diamantopoulos , et al. 2019. “Analysis of Hereditary Cancer Syndromes by Using a Panel of Genes: Novel and Multiple Pathogenic Mutations.” BMC Cancer 19: 535. 10.1186/s12885-019-5756-4 31159747 PMC6547505

[57] Auton, Adam , Gonçalo R. Abecasis , David M. Altshuler , Richard M. Durbin , Gonçalo R. Abecasis , David R. Bentley , Aravinda Chakravarti , et al. 2015. “A Global Reference for Human Genetic Variation.” Nature 526: 68–74. 10.1038/nature15393 26432245 PMC4750478

[58] Karczewski, Konrad J. , Laurent C. Francioli , Grace Tiao , Beryl B. Cummings , Jessica Alföldi , Qingbo Wang , Ryan L. Collins , et al. 2020. “The Mutational Constraint Spectrum Quantified From Variation in 141,456 Humans.” Nature 581: 434–443. 10.1038/s41586-020-2308-7 32461654 PMC7334197

[59] Wu, Lucia R. , Sherry X. Chen , Yalei Wu , Abhijit A. Patel , and David Yu Zhang . 2017. “Multiplexed Enrichment of Rare DNA Variants via Sequence‐Selective and Temperature‐Robust Amplification.” Nature Biomedical Engineering 1: 714–723. 10.1038/s41551-017-0126-5 PMC5969535 29805844

[60] Dai, Peng , Lucia Ruojia Wu , Sherry Xi Chen , Michael Xiangjiang Wang , Lauren Yuxuan Cheng , Jinny Xuemeng Zhang , Pengying Hao , et al. 2021. “Calibration‐Free NGS Quantitation of Mutations Below 0.01% VAF.” Nature Communications 12: 6123. 10.1038/s41467-021-26308-6 PMC8531361 34675197

[61] Logsdon, Glennis A. , Mitchell R. Vollger , and Evan E. Eichler . 2020. “Long‐Read Human Genome Sequencing and Its Applications.” Nature Reviews Genetics 21: 597–614. 10.1038/s41576-020-0236-x PMC7877196 32504078

[62] Liu, Tianyuan , and Ana Conesa . 2025. “Profiling the Epigenome Using Long‐Read Sequencing.” Nature Genetics 57: 27–41. 10.1038/s41588-024-02038-5 39779955

[63] Weinstein, John N. , Eric A. Collisson , Gordon B. Mills , Kenna R Mills Shaw , Brad A. Ozenberger , Kyle Ellrott , Ilya Shmulevich , Chris Sander , and Joshua M. Stuart . 2013. “The Cancer Genome Atlas Pan‐Cancer Analysis Project.” Nature Genetics 45: 1113–1120. 10.1038/ng.2764 24071849 PMC3919969

[64] Alexandrov, Ludmil B. , Serena Nik‐Zainal , David C. Wedge , Samuel A. J. R. Aparicio , Sam Behjati , Andrew V. Biankin , Graham R. Bignell , et al. 2013. “Signatures of Mutational Processes in Human Cancer.” Nature 500: 415–421. 10.1038/nature12477 23945592 PMC3776390

[65] Xu, Xue , Yuan Zhou , Xiaowen Feng , Xiong Li , Mohammad Asad , Derek Li , Bo Liao , et al. 2020. “Germline Genomic Patterns Are Associated With Cancer Risk, Oncogenic Pathways, and Clinical Outcomes.” Science Advances 6: eaba4905. 10.1126/sciadv.aba4905 33246949 PMC7695479

[66] Relling, Mary V. , and William E. Evans . 2015. “Pharmacogenomics in the Clinic.” Nature 526: 343–350. 10.1038/nature15817 26469045 PMC4711261

[67] Wang, Monica . 2023. “Pharmacogenomics in the Clinic.” Nature Reviews Nephrology 19: 277. 10.1038/s41581-023-00709-w 36964228

[68] Wan, Jonathan C. M. , Charles Massie , Javier Garcia‐Corbacho , Florent Mouliere , James D. Brenton , Carlos Caldas , Simon Pacey , Richard Baird , and Nitzan Rosenfeld . 2017. “Liquid Biopsies Come of Age: Towards Implementation of Circulating Tumour DNA.” Nature Reviews Cancer 17: 223–238. 10.1038/nrc.2017.7 28233803

[69] Cohen, Joshua D. , Lu Li , Yuxuan Wang , Christopher Thoburn , Bahman Afsari , Ludmila Danilova , Christopher Douville , et al. 2018. “Detection and Localization of Surgically Resectable Cancers With a Multi‐Analyte Blood Test.” Science 359: 926–930. 10.1126/science.aar3247 29348365 PMC6080308

[70] Ignatiadis, Michail , George W. Sledge , and Stefanie S. Jeffrey . 2021. “Liquid Biopsy Enters the Clinic—Implementation Issues and Future Challenges.” Nature Reviews Clinical Oncology 18: 297–312. 10.1038/s41571-020-00457-x 33473219

[71] Wang, Jingjing , Fang Ye , Haoxi Chai , Yujia Jiang , Teng Wang , Xia Ran , Qimin Xia , et al. 2025. “Advances and Applications in Single‐Cell and Spatial Genomics.” Science China Life Sciences 68: 1226–1282. 10.1007/s11427-024-2770-x 39792333

[72] Feinberg, Andrew P . 2018. “The Key Role of Epigenetics in Human Disease Prevention and Mitigation.” New England Journal of Medicine 378: 1323–1334. 10.1056/NEJMra1402513 29617578 PMC11567374

[73] Dor, Yuval , and Howard Cedar . 2018. “Principles of DNA Methylation and Their Implications for Biology and Medicine.” Lancet 392: 777–786. 10.1016/s0140-6736(18)31268-6 30100054

[74] Jakovcevski, Mira , and Schahram Akbarian . 2012. “Epigenetic Mechanisms in Neurological Disease.” Nature Medicine 18: 1194–1204. 10.1038/nm.2828 PMC3596876 22869198

[75] Suzuki, Miho M. , and Adrian Bird . 2008. “DNA Methylation Landscapes: Provocative Insights From Epigenomics.” Nature Reviews Genetics 9: 465–476. 10.1038/nrg2341 18463664

[76] Robertson, Keith D . 2005. “DNA Methylation and Human Disease.” Nature Reviews Genetics 6: 597–610. 10.1038/nrg1655 16136652

[77] Derbala, David , Abel Garnier , Eric Bonnet , Jean‐François Deleuze , and Jörg Tost . 2024. “Whole‐Genome Bisulfite Sequencing Protocol for the Analysis of Genome‐Wide DNA Methylation and Hydroxymethylation Patterns at Single‐Nucleotide Resolution.” Methods in Molecular Biology 2842: 353–382. 10.1007/978-1-0716-4051-7_18 39012605

[78] Liu, Yibin , Paulina Siejka‐Zielińska , Gergana Velikova , Ying Bi , Fang Yuan , Marketa Tomkova , Chunsen Bai , et al. 2019. “Bisulfite‐Free Direct Detection of 5‐Methylcytosine and 5‐Hydroxymethylcytosine at Base Resolution.” Nature Biotechnology 37: 424–429. 10.1038/s41587-019-0041-2 30804537

[79] Vaisvila, Romualdas , V.K. Chaithanya Ponnaluri , Zhiyi Sun , Bradley W. Langhorst , Lana Saleh , Shengxi Guan , Nan Dai , et al. 2021. “Enzymatic Methyl Sequencing Detects DNA Methylation at Single‐Base Resolution From Picograms of DNA.” Genome Research 31: 1280–1289. 10.1101/gr.266551.120 34140313 PMC8256858

[80] Dai, Qing , Chang Ye , Iryna Irkliyenko , Yiding Wang , Hui‐Lung Sun , Yun Gao , Yushuai Liu , et al. 2024. “Ultrafast Bisulfite Sequencing Detection of 5‐Methylcytosine in DNA and RNA.” Nature Biotechnology 42: 22. 10.1038/s41587-023-02034-w PMC11217147 38168991

[81] Dai, Qing , Tanner Baldwin , Ruitu Lyu , Bryan Daniels , Chang Ye , Chen Cao , Chenyou Zhu , et al. 2025. “Ultra‐Mild Bisulfite Outperforms Existing Methods for 5‐Methylcytosine Detection With Low Input DNA.” Nature Communications 16: 9939. 10.1038/s41467-025-66033-y PMC12615686 41233368

[82] Kohli, Rahul M. , and Yi Zhang . 2013. “TET Enzymes, TDG and the Dynamics of DNA Demethylation.” Nature 502: 472–479. 10.1038/nature12750 24153300 PMC4046508

[83] Song, Chun‐Xiao , Keith E. Szulwach , Ye Fu , Qing Dai , Chengqi Yi , Xuekun Li , Yujing Li , et al. 2011. “Selective Chemical Labeling Reveals the Genome‐Wide Distribution of 5‐Hydroxymethylcytosine.” Nature Biotechnology 29: 68–72. 10.1038/nbt.1732 PMC3107705 21151123

[84] Song, Chun‐Xiao , Keith E. Szulwach , Qing Dai , Ye Fu , Shi‐Qing Mao , Li Lin , Craig Street , et al. 2013. “Genome‐Wide Profiling of 5‐Formylcytosine Reveals Its Roles in Epigenetic Priming.” Cell 153: 678–691. 10.1016/j.cell.2013.04.001 23602153 PMC3657391

[85] Xie, Yalun , Yafen Wang , Zhiyong He , Wei Yang , Boshi Fu , Guangrong Zou , Xiong Zhang , Jinguo Huang , and Xiang Zhou . 2020. “Selective Chemical Labeling and Sequencing of 5‐Carboxylcytosine in DNA at Single‐Base Resolution.” Analytical Chemistry 92: 12710–12715. 10.1021/acs.analchem.0c03201 32803958

[86] Fabyanic, Emily B. , Peng Hu , Qi Qiu , Kiara N. Berríos , Daniel R. Connolly , Tong Wang , Jennifer Flournoy , et al. 2024. “Joint Single‐Cell Profiling Resolves 5mC and 5hmC and Reveals Their Distinct Gene Regulatory Effects.” Nature Biotechnology 42: 960–974. 10.1038/s41587-023-01909-2 PMC11525041 37640946

[87] Bai, Dongsheng , Xiaoting Zhang , Huifen Xiang , Zijian Guo , Chenxu Zhu , and Chengqi Yi . 2025. “Simultaneous Single‐Cell Analysis of 5mC and 5hmC With SIMPLE‐seq.” Nature Biotechnology 43: 85–96. 10.1038/s41587-024-02148-9 38336903

[88] Xu, Jing , Hangyu Chen , Jingang Yang , Yanmin Yang , Yuan Wu , Jun Zhang , Jiansong Yuan , et al. 2025. “Inflammation‐Related 5‐Hydroxymethylation Signatures as Markers for Clinical Presentations of Coronary Artery Disease.” Cardiovascular Diabetology 24: 237. 10.1186/s12933-025-02749-x 40551101 PMC12183871

[89] Kuerban, Subinuer , Hangyu Chen , Long Chen , Lei Zhang , Xuehui Li , Baixin Zhen , Hong Xiao , et al. 2025. “cfDNA Hydroxymethylcytosine Profiling for Detection Metastasis and Recurrence of Esophageal Squamous Cell Carcinoma.” World Journal of Surgical Oncology 23: 90. 10.1186/s12957-025-03747-9 40089765 PMC11909818

[90] Chen, Hang‐Yu , Wei‐Long Zhang , Lei Zhang , Ping Yang , Fang Li , Ze‐Ruo Yang , Jing Wang , et al. 2021. “5‐Hydroxymethylcytosine Profiles of cfDNA Are Highly Predictive of R‐CHOP Treatment Response in Diffuse Large B Cell Lymphoma Patients.” Clinical Epigenetics 13: 33. 10.1186/s13148-020-00973-8 33573703 PMC7879534

[91] Liu, M. C. , G. R. Oxnard , E. A. Klein , C. Swanton , M. V. Seiden , Minetta C. Liu , Geoffrey R. Oxnard , et al. 2020. “Sensitive and Specific Multi‐Cancer Detection and Localization Using Methylation Signatures in Cell‐Free DNA.” Annals of Oncology 31: 745–759. 10.1016/j.annonc.2020.02.011 33506766 PMC8274402

[92] Strahl, Brian D. , and C. David Allis . 2000. “The Language of Covalent Histone Modifications.” Nature 403: 41–45. 10.1038/47412 10638745

[93] Tan, Minjia , Chao Peng , Kristin A. Anderson , Peter Chhoy , Zhongyu Xie , Lunzhi Dai , Jeongsoon Park , et al. 2014. “Lysine Glutarylation Is a Protein Posttranslational Modification Regulated by SIRT5.” Cell Metabolism 19: 605–617. 10.1016/j.cmet.2014.03.014 24703693 PMC4108075

[94] Zhang, Zhihong , Minjia Tan , Zhongyu Xie , Lunzhi Dai , Yue Chen , and Yingming Zhao . 2011. “Identification of Lysine Succinylation as a New Post‐Translational Modification.” Nature Chemical Biology 7: 58–63. 10.1038/nchembio.495 21151122 PMC3065206

[95] Barski, Artem , Suresh Cuddapah , Kairong Cui , Tae‐Young Roh , Dustin E. Schones , Zhibin Wang , Gang Wei , Iouri Chepelev , and Keji Zhao . 2007. “High‐Resolution Profiling of Histone Methylations in the Human Genome.” Cell 129: 823–837. 10.1016/j.cell.2007.05.009 17512414

[96] Johnson, David S. , Ali Mortazavi , Richard M. Myers , and Barbara Wold . 2007. “Genome‐Wide Mapping of In Vivo protein‐DNA Interactions.” Science 316: 1497–1502. 10.1126/science.1141319 17540862

[97] Skene, Peter J. , and Steven Henikoff . 2017. “An Efficient Targeted Nuclease Strategy for High‐Resolution Mapping of DNA Binding Sites.” eLife 6: e21856. 10.7554/eLife.21856 28079019 PMC5310842

[98] Kaya‐Okur, Hatice S. , Steven J. Wu , Christine A. Codomo , Erica S. Pledger , Terri D. Bryson , Jorja G. Henikoff , Kami Ahmad , and Steven Henikoff . 2019. “CUT&Tag for Efficient Epigenomic Profiling of Small Samples and Single Cells.” Nature Communications 10: 1930. 10.1038/s41467-019-09982-5 PMC6488672 31036827

[99] Buenrostro, Jason D. , Paul G. Giresi , Lisa C. Zaba , Howard Y. Chang , and William J. Greenleaf . 2013. “Transposition of Native Chromatin for Fast and Sensitive Epigenomic Profiling of Open Chromatin, DNA‐Binding Proteins and Nucleosome Position.” Nature Methods 10: 1213–1218. 10.1038/nmeth.2688 24097267 PMC3959825

[100] Bartosovic, Marek , Mukund Kabbe , and Gonçalo Castelo‐Branco . 2021. “Single‐Cell CUT&Tag Profiles Histone Modifications and Transcription Factors in Complex Tissues.” Nature Biotechnology 39: 825–835. 10.1038/s41587-021-00869-9 PMC7611252 33846645

[101] Buenrostro, Jason D. , Beijing Wu , Ulrike M. Litzenburger , Dave Ruff , Michael L. Gonzales , Michael P. Snyder , Howard Y. Chang , and William J. Greenleaf . 2015. “Single‐Cell Chromatin Accessibility Reveals Principles of Regulatory Variation.” Nature 523: 486–490. 10.1038/nature14590 26083756 PMC4685948

[102] Stark, Rory , Marta Grzelak , and James Hadfield . 2019. “RNA Sequencing: The Teenage Years.” Nature Reviews Genetics 20: 631–656. 10.1038/s41576-019-0150-2 31341269

[103] Wang, Zhong , Mark Gerstein , and Michael Snyder . 2009. “RNA‐Seq: A Revolutionary Tool for Transcriptomics.” Nature Reviews Genetics 10: 57–63. 10.1038/nrg2484 PMC2949280 19015660

[104] Levin, Joshua Z. , Moran Yassour , Xian Adiconis , Chad Nusbaum , Dawn Anne Thompson , Nir Friedman , Andreas Gnirke , and Aviv Regev . 2010. “Comprehensive Comparative Analysis of Strand‐Specific RNA Sequencing Methods.” Nature Methods 7: 709–715. 10.1038/Nmeth.1491 20711195 PMC3005310

[105] Adiconis, Xian , Diego Borges‐Rivera , Rahul Satija , David S. DeLuca , Michele A. Busby , Aaron M. Berlin , Andrey Sivachenko , et al. 2013. “Comparative Analysis of RNA Sequencing Methods for Degraded or Low‐Input Samples.” Nature Methods 10: 623–629. 10.1038/nmeth.2483 23685885 PMC3821180

[106] Bray, Nicolas L. , Harold Pimentel , Páll Melsted , and Lior Pachter . 2016. “Near‐Optimal Probabilistic RNA‐seq Quantification.” Nature Biotechnology 34: 525–527. 10.1038/nbt.3519 27043002

[107] Garnett, Mathew J. , Elena J. Edelman , Sonja J. Heidorn , Chris D. Greenman , Anahita Dastur , King Wai Lau , Patricia Greninger , et al. 2012. “Systematic Identification of Genomic Markers of Drug Sensitivity in Cancer Cells.” Nature 483: 570–575. 10.1038/nature11005 22460902 PMC3349233

[108] Jiang, Peng , Sanju Sinha , Kenneth Aldape , Sridhar Hannenhalli , Cenk Sahinalp , and Eytan Ruppin . 2022. “Big Data in Basic and Translational Cancer Research.” Nature Reviews Cancer 22: 625–639. 10.1038/s41568-022-00502-0 36064595 PMC9443637

[109] Yang, Zhanqun , Ying Liu , Mengzhu Zheng , Hui Li , Ruoyao Cui , Pan Wang , and Tianhui He , et al. 2025. “Malignant Ascites Enhance γδ T Cell Cytotoxicity Toward Ovarian Cancer Via Chemokine‐Mediated Recruitment.” Cancer Biology & Medicine 22: 639–643. 10.20892/j.issn.2095-3941.2025.0072 40476592 PMC12240187

[110] Stuart, Tim , and Rahul Satija . 2019. “Integrative Single‐Cell Analysis.” Nature Reviews Genetics 20: 257–272. 10.1038/s41576-019-0093-7 30696980

[111] Chu, Xiaojing , Xiangjie Li , Yu Zhang , Guohui Dang , Yuhui Miao , Wenbin Xu , Jinyu Wang , Zemin Zhang , and Sijin Cheng . 2024. “Integrative Single‐Cell Analysis of Human Colorectal Cancer Reveals Patient Stratification With Distinct Immune Evasion Mechanisms.” Nature Cancer 5: 1409–1426. 10.1038/s43018-024-00807-z 39147986

[112] Svensson, Valentine , Roser Vento‐Tormo , and Sarah A. Teichmann . 2018. “Exponential Scaling of Single‐Cell RNA‐seq in the Past Decade.” Nature Protocols 13: 599–604. 10.1038/nprot.2017.149 29494575

[113] Zhang, Fan , Kevin Wei , Kamil Slowikowski , Chamith Y. Fonseka , Deepak A. Rao , Stephen Kelly , Susan M. Goodman , et al. 2019. “Defining Inflammatory Cell States in Rheumatoid Arthritis Joint Synovial Tissues by Integrating Single‐Cell Transcriptomics and Mass Cytometry.” Nature Immunology 20: 928–942. 10.1038/s41590-019-0378-1 31061532 PMC6602051

[114] Rodriques, Samuel G. , Robert R. Stickels , Aleksandrina Goeva , Carly A. Martin , Evan Murray , Charles R. Vanderburg , Joshua Welch , et al. 2019. “Slide‐Seq: A Scalable Technology for Measuring Genome‐Wide Expression at High Spatial Resolution.” Science 363: 1463–1467. 10.1126/science.aaw1219 30923225 PMC6927209

[115] Rao, Anjali , Dalia Barkley , Gustavo S.S. , and Itai Yanai . 2021. “Exploring Tissue Architecture Using Spatial Transcriptomics.” Nature 596: 211–220. 10.1038/s41586-021-03634-9 34381231 PMC8475179

[116] Wang, Mingyue , Qinan Hu , Tianhang Lv , Yuhang Wang , Qing Lan , Rong Xiang , Zhencheng Tu , et al. 2022. “High‐Resolution 3D Spatiotemporal Transcriptomic Maps of Developing Drosophila Embryos and Larvae.” Developmental Cell 57: 1271–1283. 10.1016/j.devcel.2022.04.006 35512700

[117] Longo, Sophia K. , Margaret G. Guo , Andrew L. Ji , and Paul A. Khavari . 2021. “Integrating Single‐Cell and Spatial Transcriptomics to Elucidate Intercellular Tissue Dynamics.” Nature Reviews Genetics 22: 627–644. 10.1038/s41576-021-00370-8 PMC9888017 34145435

[118] Cang, Zixuan , Yanxiang Zhao , Axel A. Almet , Adam Stabell , Raul Ramos , Maksim V. Plikus , Scott X. Atwood , and Qing Nie . 2023. “Screening Cell‐Cell Communication in Spatial Transcriptomics via Collective Optimal Transport.” Nature Methods 20: 218–228. 10.1038/s41592-022-01728-4 36690742 PMC9911355

[119] Crosetto, Nicola , Magda Bienko , and Alexander van Oudenaarden . 2015. “Spatially Resolved Transcriptomics and Beyond.” Nature Reviews Genetics 16: 57–66. 10.1038/nrg3832 25446315

[120] Moor, Andreas E. , and Shalev Itzkovitz . 2017. “Spatial Transcriptomics: Paving the Way for Tissue‐Level Systems Biology.” Current Opinion in Biotechnology 46: 126–133. 10.1016/j.copbio.2017.02.004 28346891

[121] Zhuang, Xiaowei . 2021. “Spatially Resolved Single‐Cell Genomics and Transcriptomics by Imaging.” Nature Methods 18: 18–22. 10.1038/s41592-020-01037-8 33408406 PMC9805800

[122] Ament, Isabelle Heifetz , Nicole DeBruyne , Feng Wang , and Lan Lin . 2025. “Long‐Read RNA Sequencing: A Transformative Technology for Exploring Transcriptome Complexity in Human Diseases.” Molecular Therapy 33: 883–894. 10.1016/j.ymthe.2024.11.025 39563027 PMC11897757

[123] Foord, Careen , Justine Hsu , Julien Jarroux , Wen Hu , Natan Belchikov , Shaun Pollard , Yi He , Anoushka Joglekar , and Hagen U. Tilgner . 2023. “The Variables on RNA Molecules: Concert or Cacophony? Answers in Long‐Read Sequencing.” Nature Methods 20: 20–24. 10.1038/s41592-022-01715-9 36635536

[124] Abbott, Jamie A. , Christopher S. Francklyn , and Susan M. Robey‐Bond . 2014. “Transfer RNA and Human Disease.” Frontiers in Genetics 5: 158. 10.3389/fgene.2014.00158 24917879 PMC4042891

[125] Padhiar, Nigam H. , Upendra Katneni , Anton A. Komar , Yuri Motorin , and Chava Kimchi‐Sarfaty . 2024. “Advances in Methods for tRNA Sequencing and Quantification.” Trends in Genetics 40: 276–290. 10.1016/j.tig.2023.11.001 38123442 PMC12376271

[126] Zheng, Guanqun , Yidan Qin , Wesley C. Clark , Qing Dai , Chengqi Yi , Chuan He , Alan M. Lambowitz , and Tao Pan . 2015. “Efficient and Quantitative High‐Throughput tRNA Sequencing.” Nature Methods 12: 835–837. 10.1038/nmeth.3478 26214130 PMC4624326

[127] Cozen, Aaron E. , Erin Quartley , Andrew D. Holmes , Eva Hrabeta‐Robinson , Eric M. Phizicky , and Todd M. Lowe . 2015. “ARM‐seq: AlkB‐Facilitated RNA Methylation Sequencing Reveals a Complex Landscape of Modified tRNA Fragments.” Nature Methods 12: 879–884. 10.1038/nmeth.3508 26237225 PMC4553111

[128] Lucas, Morghan C. , Leszek P. Pryszcz , Rebeca Medina , Ivan Milenkovic , Noelia Camacho , Virginie Marchand , Yuri Motorin , Lluís Ribas de Pouplana , and Eva Maria Novoa . 2024. “Quantitative Analysis of tRNA Abundance and Modifications by Nanopore RNA Sequencing.” Nature Biotechnology 42: 72–86. 10.1038/s41587-023-01743-6 PMC10791586 37024678

[129] Lowe, Todd M. , and Sean R. Eddy . 1997. “tRNAscan‐SE: A Program for Improved Detection of Transfer RNA Genes in Genomic Sequence.” Nucleic Acids Research 25: 955–964. 10.1093/nar/25.5.955 9023104 PMC146525

[130] Chan, Patricia P. , Brian Y. Lin , Allysia J. Mak , and Todd M. Lowe . 2021. “tRNAscan‐SE 2.0: Improved Detection and Functional Classification of Transfer RNA Genes.” Nucleic Acids Research 49: 9077–9096. 10.1093/nar/gkab688 34417604 PMC8450103

[131] Behrens, Andrew , Geraldine Rodschinka , and Danny D. Nedialkova . 2021. “High‐Resolution Quantitative Profiling of tRNA Abundance and Modification Status in Eukaryotes by mim‐tRNAseq.” Molecular Cell 81: 1802–1815.e7. 10.1016/j.molcel.2021.01.028 33581077 PMC8062790

[132] Murillo‐Recio, Marina , Ignacio Miguel Martínez de Lejarza Samper , Cristina Tuñí i Domínguez , Lluís Ribas de Pouplana , and Adrian Gabriel Torres . 2022. “tRNAstudio: Facilitating the Study of Human Mature tRNAs From Deep Sequencing Datasets.” Bioinformatics 38: 2934–2936. 10.1093/bioinformatics/btac198 35561195

[133] Sordyl, Dominik , Etienne Boileau , Agata Bernat , Satyabrata Maiti , Sunandan Mukherjee , S Naeim Moafinejad , Masoud Amiri Farsani , et al. 2026. “MODOMICS: A Database of RNA Modifications and Related Information. 2025 Update and 20th Anniversary.” Nucleic Acids Research 54: D219–D225. 10.1093/nar/gkaf1284 41277531 PMC12807697

[134] Hu, Yanping , Shuangyong Yan , Haohao Yan , Jingping Su , Zhongqiu Cui , Junling Li , Shengjun Wang , et al. 2025. “PacBio Full‐Length Transcriptome Analysis Reveals the Role of tRNA‐like Structures in RNA Processing.” Cellular Signalling 125: 111515. 10.1016/j.cellsig.2024.111515 39571702

[135] Vilfan, Igor D. , Yu‐Chih Tsai , Tyson A. Clark , Jeffrey Wegener , Qing Dai , Chengqi Yi , Tao Pan , Stephen W. Turner , and Jonas Korlach . 2013. “Analysis of RNA Base Modification and Structural Rearrangement by Single‐Molecule Real‐Time Detection of Reverse Transcription.” Journal of Nanobiotechnology 11: 8. 10.1186/1477-3155-11-8 23552456 PMC3623877

[136] Scheepbouwer, Chantal , Ernesto Aparicio‐Puerta , Cristina Gomez‐Martin , Heleen Verschueren , Monique van Eijndhoven , Laurine E. Wedekind , Stavros Giannoukakos , et al. 2023. “ALL‐tRNAseq Enables Robust tRNA Profiling in Tissue Samples.” Genes & Development 37: 243–257. 10.1101/gad.350233.122 36810209 PMC10111867

[137] Goodarzi, Hani , Hoang C. B. Nguyen , Steven Zhang , Brian D. Dill , Henrik Molina , and Sohail F. Tavazoie . 2016. “Modulated Expression of Specific tRNAs Drives Gene Expression and Cancer Progression.” Cell 165: 1416–1427. 10.1016/j.cell.2016.05.046 27259150 PMC4915377

[138] Huang, Shi‐qiong , Bao Sun , Zong‐ping Xiong , Yan Shu , Hong‐hao Zhou , Wei Zhang , Jing Xiong , and Qing Li . 2018. “The Dysregulation of tRNAs and tRNA Derivatives in Cancer.” Journal of Experimental & Clinical Cancer Research 37: 101. 10.1186/s13046-018-0745-z 29743091 PMC5944149

[139] Madrer, Nimrod , Shani Vaknine‐Treidel , Tamara Zorbaz , Yonat Tzur , Estelle R. Bennett , Paz Drori , Nitzan Suissa , et al. 2025. “Pre‐Symptomatic Parkinson's Disease Blood Test Quantifying Repetitive Sequence Motifs in Transfer RNA Fragments.” Nature Aging 5: 868–882. 10.1038/s43587-025-00851-z 40216989 PMC12092246

[140] Lv, Xinxin , Ruorui Zhang , Shanshan Li , and Xin Jin . 2024. “tRNA Modifications and Dysregulation: Implications for Brain Diseases.” Brain Sciences 14: 633. 10.3390/brainsci14070633 39061374 PMC11274612

[141] Zhang, Jie , Yingfei Xi , Qiuping Fei , Jun Xu , and Jinxing Hu . 2023. “Identification of tRNA‐derived RNAs in Adipose Tissue From Overweight Type 2 Diabetes Mellitus Patients and Their Potential Biological Functions.” Frontiers in Endocrinology 14: 1139157. 10.3389/fendo.2023.1139157 37484941 PMC10358832

[142] Arroyo, Maria Nicol , Jonathan Alex Green , Miriam Cnop , and Mariana Igoillo‐Esteve . 2021. “tRNA Biology in the Pathogenesis of Diabetes: Role of Genetic and Environmental Factors.” International Journal of Molecular Sciences 22: 496. 10.3390/ijms22020496 33419045 PMC7825315

[143] Aebersold, Ruedi , and Matthias Mann . 2016. “Mass‐Spectrometric Exploration of Proteome Structure and Function.” Nature 537: 347–355. 10.1038/nature19949 27629641

[144] Epstein, Lois B. , Diana M. Smith , Neil M. Matsui , Huu M. Tran , Chrissy Sullivan , Ines Raineri , Alma L. Burlingame , et al. 1996. “Identification of Cytokine‐Regulated Proteins in Normal and Malignant Cells by the Combination of Two‐Dimensional Polyacrylamide Gel Electrophoresis, Mass Spectrometry, Edman Degradation and Immunoblotting and Approaches to the Analysis of Their Functional Roles.” Electrophoresis 17: 1655–1670. 10.1002/elps.1150171103 8982598

[145] Deng, Jingjing , Hediye Erdjument‐Bromage , and Thomas A. Neubert . 2019. “Quantitative Comparison of Proteomes Using SILAC.” Current Protocols in Protein Science 95: e74. 10.1002/cpps.74 30238645 PMC6342620

[146] Yan, Jing , Bei Xie , Ye Tian , Li Huang , Shuli Zou , Zhiheng Peng , Zhuan Liu , and Linjing Li . 2022. “iTRAQ‐Based Proteome Profiling of Differentially Expressed Proteins in Insulin‐Resistant Human Hepatocellular Carcinoma.” Frontiers in Cell and Developmental Biology 10: 836041. 10.3389/fcell.2022.836041 35281088 PMC8914942

[147] Thompson, Andrew , Jürgen Schäfer , Karsten Kuhn , Stefan Kienle , Josef Schwarz , Günter Schmidt , Thomas Neumann , and Christian Hamon . 2003. “Tandem Mass Tags: A Novel Quantification Strategy for Comparative Analysis of Complex Protein Mixtures by MS/MS.” Analytical Chemistry 75: 1895–1904. 10.1021/ac0262560 12713048

[148] Välikangas, Tommi , Tomi Suomi , and Laura L. Elo . 2018. “A Systematic Evaluation of Normalization Methods in Quantitative Label‐Free Proteomics.” Briefings in Bioinformatics 19: 1–11. 10.1093/bib/bbw095 27694351 PMC5862339

[149] Wilhelm, Mathias , Judith Schlegl , Hannes Hahne , Amin Moghaddas Gholami , Marcus Lieberenz , Mikhail M. Savitski , Emanuel Ziegler , et al. 2014. “Mass‐Spectrometry‐Based Draft of the Human Proteome.” Nature 509: 582–587. 10.1038/nature13319 24870543

[150] Giansanti, Piero , Patroklos Samaras , Yangyang Bian , Chen Meng , Andrea Coluccio , Martin Frejno , Hannah Jakubowsky , et al. 2022. “Mass Spectrometry‐Based Draft of the Mouse Proteome.” Nature Methods 19: 803–811. 10.1038/s41592-022-01526-y 35710609 PMC7613032

[151] Guo, Tiannan , and Ruedi Aebersold . 2023. “Recent Advances of Data‐Independent Acquisition Mass Spectrometry‐Based Proteomics.” Proteomics 23: e2200011. 10.1002/pmic.202200011 37039310

[152] Wik, Lotta , Niklas Nordberg , John Broberg , Johan Björkesten , Erika Assarsson , Sara Henriksson , Ida Grundberg , et al. 2021. “Proximity Extension Assay in Combination With Next‐Generation Sequencing for High‐Throughput Proteome‐Wide Analysis.” Molecular & Cellular Proteomics 20: 100168. 10.1016/j.mcpro.2021.100168 34715355 PMC8633680

[153] Assarsson, Erika , Martin Lundberg , Göran Holmquist , Johan Björkesten , Stine Bucht Thorsen , Daniel Ekman , Anna Eriksson , et al. 2014. “Homogenous 96‐Plex PEA Immunoassay Exhibiting High Sensitivity, Specificity, and Excellent Scalability.” PLOS One 9: e95192. 10.1371/journal.pone.0095192 24755770 PMC3995906

[154] Martínez‐Moreno, Julio Manuel , Adrián Llamas‐Urbano , Nuria Barbarroja , and Carlos Pérez‐Sánchez . 2025. “Proteomics by qPCR Using the Proximity Extension Assay (PEA).” In Protein Arrays: Methods and Protocols, 129–142. Springer US. 10.1007/978-1-0716-4595-6_10 40601148

[155] Sissala, Noora , Haris Babačić , Isabelle R. Leo , Xiaofang Cao , Jenny Forshed , Lars E. Eriksson , Janne Lehtiö , et al. 2025. “Comparative Evaluation of Olink Explore 3072 and Mass Spectrometry With Peptide Fractionation for Plasma Proteomics.” Communications Chemistry 8: 327. 10.1038/s42004-025-01753-2 41188494 PMC12586489

[156] Bennett, Hayley M. , William Stephenson , Christopher M. Rose , and Spyros Darmanis . 2023. “Single‐Cell Proteomics Enabled by Next‐Generation Sequencing or Mass Spectrometry.” Nature Methods 20: 363–374. 10.1038/s41592-023-01791-5 36864196

[157] Mund, Andreas , Andreas‐David Brunner , and Matthias Mann . 2022. “Unbiased Spatial Proteomics With Single‐Cell Resolution in Tissues.” Molecular Cell 82: 2335–2349. 10.1016/j.molcel.2022.05.022 35714588

[158] Leduc, Andrew , R. Gray Huffman , Joshua Cantlon , Saad Khan , and Nikolai Slavov . 2022. “Exploring Functional Protein Covariation Across Single Cells Using nPOP.” Genome Biology 23: 261. 10.1186/s13059-022-02817-5 36527135 PMC9756690

[159] Editorial . 2023. “Single‐Cell Proteomics: Challenges and Prospects.” Nature Methods 20: 317–318. 10.1038/s41592-023-01828-9 36899170

[160] Weng, Yicheng , Wendong Chen , Qian Kong , Ruixiang Wang , Ruxin Zeng , An He , Yanjun Liu , et al. 2024. “DeKinomics Pulse‐Chases Kinase Functions in Living Cells.” Nature Chemical Biology 20: 615–623. 10.1038/s41589-023-01497-x 38167916

[161] Cho, Kelvin F. , Tess C. Branon , Namrata D. Udeshi , Samuel A. Myers , Steven A. Carr , and Alice Y. Ting . 2020. “Proximity Labeling in Mammalian Cells With TurboID and split‐TurboID.” Nature Protocols 15: 3971–3999. 10.1038/s41596-020-0399-0 33139955

[162] Roux, Kyle J. , Dae In Kim , Brian Burke , and Danielle G. May . 2018. “BioID: A Screen for Protein‐Protein Interactions.” Current Protocols in Protein Science 91: 19.23.1–19.23.5. 10.1002/cpps.51 PMC6028010 29516480

[163] Wu, Jhen‐Wei , Chueh‐Wen Wang , Wei Yang Hong , Anna C. C. Jang , and Yu‐Chiuan Chang . 2024. “Detecting Native Protein–Protein Interactions by APEX2 Proximity Labeling in Drosophila Tissues.” Bio‐protocol 14: e5090. 10.21769/BioProtoc.5090 39512889 PMC11540050

[164] Li, Kexin , Xiao Xie , Rui Gao , Zhaoming Chen , Mingdong Yang , Zhihui Wen , Yicheng Weng , et al. 2025. “Spatiotemporal Protein Interactome Profiling Through Condensation‐Enhanced Photocrosslinking.” Nature Chemistry 17: 111–123. 10.1038/s41557-024-01663-1 39501047

[165] Huang, Zongyu , Ziqi Liu , Xiao Xie , Ruxin Zeng , Zujie Chen , Linghao Kong , Xinyuan Fan , and Peng R. Chen . 2021. “Bioorthogonal Photocatalytic Decaging‐Enabled Mitochondrial Proteomics.” Journal of the American Chemical Society 143: 18714–18720. 10.1021/jacs.1c09171 34709827

[166] Zhou, Nan , Yan Zhang , Peng R. Chen , and Xinyuan Fan . 2025. “Time‐Resolved Photocatalytic Proximity Labeling Uncovers ER Proteome Dynamics Underlying UPR‐to‐Apoptosis Transition.” Proceedings of the National Academy of Sciences of the United States of America 122: e2503115122. 10.1073/pnas.2503115122 40768357 PMC12358923

[167] Patti, Gary J. , Oscar Yanes , and Gary Siuzdak . 2012. “Metabolomics: The Apogee of the Omics Trilogy.” Nature Reviews Molecular Cell Biology 13: 263–269. 10.1038/nrm3314 22436749 PMC3682684

[168] Wishart, David S . 2019. “Metabolomics for Investigating Physiological and Pathophysiological Processes.” Physiological Reviews 99: 1819–1875. 10.1152/physrev.00035.2018 31434538

[169] Schrimpe‐Rutledge, Alexandra C. , Simona G. Codreanu , Stacy D. Sherrod , and John A. McLean . 2016. “Untargeted Metabolomics Strategies‐Challenges and Emerging Directions.” Journal of the American Society for Mass Spectrometry 27: 1897–1905. 10.1007/s13361-016-1469-y 27624161 PMC5110944

[170] Roberts, Lee D. , Amanda L. Souza , Robert E. Gerszten , and Clary B. Clish . 2012. “Targeted Metabolomics.” Current Protocols in Molecular Biology 30: 30.2.1–30.2.24. 10.1002/0471142727.mb3002s98 PMC3334318 22470063

[171] Dunn, Warwick B. , David Broadhurst , Paul Begley , Eva Zelena , Sue Francis‐McIntyre , Nadine Anderson , Marie Brown , et al. 2011. “Procedures for Large‐Scale Metabolic Profiling of Serum and Plasma Using Gas Chromatography and Liquid Chromatography Coupled to Mass Spectrometry.” Nature Protocols 6: 1060–1083. 10.1038/nprot.2011.335 21720319

[172] Larive, Cynthia K. , Gregory A. Barding , and Meredith M. Dinges . 2015. “NMR Spectroscopy for Metabolomics and Metabolic Profiling.” Analytical Chemistry 87: 133–146. 10.1021/ac504075g 25375201

[173] Cheng, Jing , Wenxian Lan , Guangyong Zheng , and Xianfu Gao . 2018. “Metabolomics: A High‐Throughput Platform for Metabolite Profile Exploration.” Methods in Molecular Biology 1754: 265–292. 10.1007/978-1-4939-7717-8_16 29536449

[174] Cui, Liang , Haitao Lu , and Yie Hou Lee . 2018. “Challenges and Emergent Solutions for LC‐MS/MS Based Untargeted Metabolomics in Diseases.” Mass Spectrometry Reviews 37: 772–792. 10.1002/mas.21562 29486047

[175] Yuan, Zhiyuan , Qiming Zhou , Lesi Cai , Lin Pan , Weiliang Sun , Shiwei Qumu , Si Yu , et al. 2021. “SEAM Is a Spatial Single Nuclear Metabolomics Method for Dissecting Tissue Microenvironment.” Nature Methods 18: 1223–1232. 10.1038/s41592-021-01276-3 34608315

[176] Delafiori, Jeany , Mohammed Shahraz , Andreas Eisenbarth , Volker Hilsenstein , Bernhard Drotleff , Alberto Bailoni , Bishoy Wadie , et al. 2025. “HT SpaceM: A High‐Throughput and Reproducible Method for Small‐Molecule Single‐Cell Metabolomics.” Cell 188: 6028–6043.e11. 10.1016/j.cell.2025.08.015 40914160

[177] Johnson, Caroline H. , Julijana Ivanisevic , and Gary Siuzdak . 2016. “Metabolomics: Beyond Biomarkers and Towards Mechanisms.” Nature Reviews Molecular Cell Biology 17: 451–459. 10.1038/nrm.2016.25 PMC5729912 26979502

[178] Wishart, David S . 2016. “Emerging Applications of Metabolomics in Drug Discovery and Precision Medicine.” Nature Reviews Drug Discovery 15: 473–484. 10.1038/nrd.2016.32 26965202

[179] Cheng, Jie , Jinxin Yan , Ying Liu , Jiangzhou Shi , Haoyu Wang , Hanyang Zhou , Yinglin Zhou , et al. 2023. “Cancer‐Cell‐Derived Fumarate Suppresses the Anti‐Tumor Capacity of CD8(+) T Cells in the Tumor Microenvironment.” Cell Metabolism 35: 961–978.e10. 10.1016/j.cmet.2023.04.017 37178684

[180] Ho, Ping‐Chih , Jessica Dauz Bihuniak , Andrew N. Macintyre , Matthew Staron , Xiaojing Liu , Robert Amezquita , Yao‐Chen Tsui , et al. 2015. “Phosphoenolpyruvate Is a Metabolic Checkpoint of Anti‐Tumor T Cell Responses.” Cell 162: 1217–1228. 10.1016/j.cell.2015.08.012 26321681 PMC4567953

[181] Li, Xiaoyun , Mathias Wenes , Pedro Romero , Stanley Ching‐Cheng Huang , Sarah‐Maria Fendt , and Ping‐Chih Ho . 2019. “Navigating Metabolic Pathways to Enhance Antitumour Immunity and Immunotherapy.” Nature Reviews Clinical Oncology 16: 425–441. 10.1038/s41571-019-0203-7 30914826

[182] Wang, Thomas J. , Martin G. Larson , Ramachandran S. Vasan , Susan Cheng , Eugene P. Rhee , Elizabeth McCabe , Gregory D. Lewis , et al. 2011. “Metabolite Profiles and the Risk of Developing Diabetes.” Nature Medicine 17: 448–453. 10.1038/nm.2307 PMC3126616 21423183

[183] Wang, Pan , Jihong Ma , Wenjing Li , Qilong Wang , Yinan Xiao , Yuening Jiang , Xiaoyang Gu , et al. 2023. “Profiling the Metabolome of Uterine Fluid for Early Detection of Ovarian Cancer.” Cell Reports Medicine 4: 101061. 10.1016/j.xcrm.2023.101061 37267943 PMC10313936

[184] Weiss, Robert H. , and Kyoungmi Kim . 2011. “Metabolomics in the Study of Kidney Diseases.” Nature Reviews Nephrology 8: 22–33. 10.1038/nrneph.2011.152 22025087

[185] Griffin, Julian L. , Helen Atherton , John Shockcor , and Luigi Atzori . 2011. “Metabolomics as a Tool for Cardiac Research.” Nature Reviews Cardiology 8: 630–643. 10.1038/nrcardio.2011.138 21931361

[186] Sender, Ron , Shai Fuchs , and Ron Milo . 2016. “Are We Really Vastly Outnumbered? Revisiting the Ratio of Bacterial to Host Cells in Humans.” Cell 164: 337–340. 10.1016/j.cell.2016.01.013 26824647

[187] Cani, Patrice D . 2018. “Human Gut Microbiome: Hopes, Threats and Promises.” Gut 67: 1716–1725. 10.1136/gutjnl-2018-316723 29934437 PMC6109275

[188] Xu, Jing , and Yuejin Yang . 2021. “Gut Microbiome and Its Meta‐Omics Perspectives: Profound Implications for Cardiovascular Diseases.” Gut Microbes 13: 1936379. 10.1080/19490976.2021.1936379 34170211 PMC8237965

[189] Sequeira, João C. , Vítor Pereira , M. Madalena Alves , M. Alcina Pereira , Miguel Rocha , and Andreia F. Salvador . 2024. “MOSCA 2.0: A Bioinformatics Framework for Metagenomics, Metatranscriptomics and Metaproteomics Data Analysis and Visualization.” Molecular Ecology Resources 24: e13996. 10.1111/1755-0998.13996 39099161

[190] Quince, Christopher , Alan W. Walker , Jared T. Simpson , Nicholas J. Loman , and Nicola Segata . 2017. “Shotgun Metagenomics, From Sampling to Analysis.” Nature Biotechnology 35: 833–844. 10.1038/nbt.3935 28898207

[191] Callahan, Benjamin J. , Paul J. McMurdie , Michael J. Rosen , Andrew W. Han , Amy Jo A. Johnson , and Susan P. Holmes . 2016. “DADA2: High‐Resolution Sample Inference From Illumina Amplicon Data.” Nature Methods 13: 581–583. 10.1038/nmeth.3869 27214047 PMC4927377

[192] Lloyd‐Price, Jason , Cesar Arze , Ashwin N. Ananthakrishnan , Melanie Schirmer , Julian Avila‐Pacheco , Tiffany W. Poon , Elizabeth Andrews , et al. 2019. “Multi‐Omics of the Gut Microbial Ecosystem in Inflammatory Bowel Diseases.” Nature 569: 655–662. 10.1038/s41586-019-1237-9 31142855 PMC6650278

[193] Proctor, Lita M. , Heather H. Creasy , Jennifer M. Fettweis , Jason Lloyd‐Price , Anup Mahurkar , Wenyu Zhou , Gregory A. Buck , et al. 2019. “The Integrative Human Microbiome Project.” Nature 569: 641–648. 10.1038/s41586-019-1238-8 31142853 PMC6784865

[194] Wang, Yunhao , Yue Zhao , Audrey Bollas , Yuru Wang , and Kin Fai Au . 2021. “Nanopore Sequencing Technology, Bioinformatics and Applications.” Nature Biotechnology 39: 1348–1365. 10.1038/s41587-021-01108-x PMC8988251 34750572

[195] Buetas, Elena , Marta Jordán‐López , Andrés López‐Roldán , Giuseppe D'Auria , Llucia Martínez‐Priego , Miguel Carda‐Diéguez , and Alex Mira . 2024. “Full‐Length 16S rRNA Gene Sequencing by PacBio Improves Taxonomic Resolution in Human Microbiome Samples.” BMC Genomics 25: 310. 10.1186/s12864-024-10213-5 38528457 PMC10964587

[196] Valdés‐Mas, Rafael , Avner Leshem , Danping Zheng , Yotam Cohen , Lara Kern , Niv Zmora , Yiming He , et al. 2025. “Metagenome‐Informed Metaproteomics of the Human Gut Microbiome, Host, and Dietary Exposome Uncovers Signatures of Health and Inflammatory Bowel Disease.” Cell 188: 1062–1083. 10.1016/j.cell.2024.12.016 39837331

[197] Zhao, Yuchao , Jian Tan , Luoyun Fang , and Linshu Jiang . 2024. “Harnessing Meta‐Omics to Unveil and Mitigate Methane Emissions in Ruminants: Integrative Approaches and Future Directions.” Science of the Total Environment 951: 175732. 10.1016/j.scitotenv.2024.175732 39182764

[198] Wahlström, Annika , Sama I. Sayin , Hanns‐Ulrich Marschall , and Fredrik Bäckhed . 2016. “Intestinal Crosstalk Between Bile Acids and Microbiota and Its Impact on Host Metabolism.” Cell Metabolism 24: 41–50. 10.1016/j.cmet.2016.05.005 27320064

[199] Parada Venegas, Daniela , Marjorie K. De la Fuente , Glauben Landskron , María Julieta González , Rodrigo Quera , Gerard Dijkstra , Hermie J. M. Harmsen , Klaas Nico Faber , and Marcela A. Hermoso . 2019. “Short Chain Fatty Acids (SCFAs)‐Mediated Gut Epithelial and Immune Regulation and Its Relevance for Inflammatory Bowel Diseases.” Frontiers in Immunology 10: 277. 10.3389/fimmu.2019.00277 30915065 PMC6421268

[200] Jayakrishnan, Thejus T. , Naseer Sangwan , Shimoli V. Barot , Nicole Farha , Arshiya Mariam , Shao Xiang , Federico Aucejo , et al. 2024. “Multi‐Omics Machine Learning to Study Host‐Microbiome Interactions in Early‐Onset Colorectal Cancer.” npj Precision Oncology 8: 146. 10.1038/s41698-024-00647-1 39020083 PMC11255257

[201] Zhu, Yan , Yiteng Tang , Xin Qi , and Xiong Zhu . 2026. “Transformer Models, Graph Networks, and Generative AI in Gut Microbiome Research: A Narrative Review.” Bioengineering 13: 144. 10.3390/bioengineering13020144 41749685 PMC12938703

[202] Wang, Yiwen , and Kim‐Anh LêCao . 2020. “Managing Batch Effects in Microbiome Data.” Briefings in Bioinformatics 21: 1954–1970. 10.1093/bib/bbz105 31776547

[203] Yu, Ying , Yuanbang Mai , Yuanting Zheng , and Leming Shi . 2024. “Assessing and Mitigating Batch Effects in Large‐Scale Omics Studies.” Genome Biology 25: 254. 10.1186/s13059-024-03401-9 39363244 PMC11447944

[204] Zheng, Wenshan , Shijie Zhao , Yehang Yin , Huidan Zhang , David M. Needham , Ethan D. Evans , Chengzhen L. Dai , et al. 2022. “High‐Throughput, Single‐Microbe Genomics With Strain Resolution, Applied to a Human Gut Microbiome.” Science 376: eabm1483. 10.1126/science.abm1483 35653470

[205] Ntekas, Ioannis , Lena Takayasu , David W. McKellar , Benjamin Grodner , Chase Holdener , Peter Schweitzer , Young Seo Park , et al. 2026. “Spatial Transcriptomics Maps Host–Gut Microbiome Biogeography at High Resolution.” Nature Microbiology 11: 1193–1204. 10.1038/s41564-026-02286-7 PMC13171632 41792309

[206] Dama, Adam C. , Kevin S. Kim , Danielle M. Leyva , Annamarie P. Lunkes , Noah S. Schmid , Kenan Jijakli , and Paul A. Jensen . 2023. “BacterAI Maps Microbial Metabolism Without Prior Knowledge.” Nature Microbiology 8: 1018–1025. 10.1038/s41564-023-01376-0 37142775

[207] Huang, Yiming , Ravi U. Sheth , Shijie Zhao , Lucas A. Cohen , Kendall Dabaghi , Thomas Moody , Yiwei Sun , et al. 2023. “High‐Throughput Microbial Culturomics Using Automation and Machine Learning.” Nature Biotechnology 41: 1424–1433. 10.1038/s41587-023-01674-2 PMC10567565 36805559

[208] Hu, Jian , Xiangjie Li , Kyle Coleman , Amelia Schroeder , Nan Ma , David J. Irwin , Edward B. Lee , Russell T. Shinohara , and Mingyao Li . 2021. “SpaGCN: Integrating Gene Expression, Spatial Location and Histology to Identify Spatial Domains and Spatially Variable Genes by Graph Convolutional Network.” Nature Methods 18: 1342–1351. 10.1038/s41592-021-01255-8 34711970

[209] Dong, Kangning , and Shihua Zhang . 2022. “Deciphering Spatial Domains From Spatially Resolved Transcriptomics With an Adaptive Graph Attention Auto‐Encoder.” Nature Communications 13: 1739. 10.1038/s41467-022-29439-6 PMC8976049 35365632

[210] Zitnik, Marinka , Francis Nguyen , Bo Wang , Jure Leskovec , Anna Goldenberg , and Michael M. Hoffman . 2019. “Machine Learning for Integrating Data in Biology and Medicine: Principles, Practice, and Opportunities.” Information Fusion 50: 71–91. 10.1016/j.inffus.2018.09.012 30467459 PMC6242341

[211] Guo, Fei , Renchu Guan , Yaohang Li , Qi Liu , Xiaowo Wang , Can Yang , and Jianxin Wang . 2025. “Foundation Models in Bioinformatics.” National Science Review 12: nwaf028. 10.1093/nsr/nwaf028 40078374 PMC11900445

[212] Zhou, Zhihan , Yanrong Ji , Weijian Li , Pratik Dutta , Ramana Davuluri , and Han Liu . 2023. “Dnabert‐2: Efficient Foundation Model and Benchmark for Multi‐Species Genome.” *arXiv*, 10.48550/arXiv.2306.15006

[213] Lin, Zeming , Halil Akin , Roshan Rao , Brian Hie , Zhongkai Zhu , Wenting Lu , Nikita Smetanin , et al. 2023. “Evolutionary‐Scale Prediction of Atomic‐Level Protein Structure With a Language Model.” Science 379: 1123–1130. 10.1126/science.ade2574 36927031

[214] Brixi, Garyk , Matthew G. Durrant , Jerome Ku , Mohsen Naghipourfar , Michael Poli , Gwanggyu Sun , Greg Brockman , et al. 2026. “Genome Modelling and Design Across All Domains of Life With Evo 2.” Nature 652: 1349–1361. 10.1038/s41586-026-10176-5 41781614 PMC13128491

[215] Akiyama, Manato , and Yasubumi Sakakibara . 2022. “Informative RNA Base Embedding for RNA Structural Alignment and Clustering by Deep Representation Learning.” NAR Genomics and Bioinformatics 4: lqac012. 10.1093/nargab/lqac012 35211670 PMC8862729

[216] Cai, Hongmin , Boan Ji , Shangyan Cai , Yi Liao , Jiazhou Chen , and Weitian Huang . 2026. “GE2Hist: Generating Histology Images From Single‐Cell Gene Expression via Cross‐Modal Generative Network.” *Medical Image Computing and Computer Assisted Intervention—MICCAI 2025*: 240–250. 10.1007/978-3-032-05141-7_24

[217] He, Xiujing , Xiaowei Liu , Fengli Zuo , Hubing Shi , and Jing Jing . 2023. “Artificial Intelligence‐Based Multi‐Omics Analysis Fuels Cancer Precision Medicine.” Seminars in Cancer Biology 88: 187–200. 10.1016/j.semcancer.2022.12.009 36596352

[218] Carrillo‐Perez, Francisco , Marija Pizurica , Michael G. Ozawa , Hannes Vogel , Robert B. West , Christina S. Kong , Luis Javier Herrera , Jeanne Shen , and Olivier Gevaert . 2023. “Synthetic Whole‐Slide Image Tile Generation With Gene Expression Profile‐Infused Deep Generative Models.” Cell Reports Methods 3: 100534. 10.1016/j.crmeth.2023.100534 37671024 PMC10475789

[219] Cui, Haotian , Chloe Wang , Hassaan Maan , Kuan Pang , Fengning Luo , Nan Duan , and Bo Wang . 2024. “scGPT: Toward Building a Foundation Model for Single‐Cell Multi‐Omics Using Generative AI.” Nature Methods 21: 1470–1480. 10.1038/s41592-024-02201-0 38409223

[220] Bunne, Charlotte , Stefan G. Stark , Gabriele Gut , Jacobo Sarabia Del Castillo , Mitch Levesque , Kjong‐Van Lehmann , Lucas Pelkmans , Andreas Krause , and Gunnar Rätsch . 2023. “Learning Single‐Cell Perturbation Responses Using Neural Optimal Transport.” Nature Methods 20: 1759–1768. 10.1038/s41592-023-01969-x 37770709 PMC10630137

[221] Varrone, Marco , Daniele Tavernari , Albert Santamaria‐Martínez , Logan A. Walsh , and Giovanni Ciriello . 2024. “CellCharter Reveals Spatial Cell Niches Associated With Tissue Remodeling and Cell Plasticity.” Nature Genetics 56: 74–84. 10.1038/s41588-023-01588-4 38066188

[222] Wang, Jun , Yuanhua Huang , and Ole Winther . 2025. “SpatialFormer: Universal Spatial Representation Learning From Subcellular Molecular to Multicellular Landscapes.” *bioRxiv*, 10.1101/2025.01.18.633701 42533054

[223] Lin, Yuxiang , Ling Luo , Ying Chen , Xushi Zhang , Zihui Wang , Wenxian Yang , Mengsha Tong , and Rongshan Yu . 2024. “St‐align: A Multimodal Foundation Model for Image‐Gene Alignment in Spatial Transcriptomics.” *arXiv*, 10.48550/arXiv.2411.16793

[224] Shaban, Muhammad , Yuzhou Chang , Huaying Qiu , Yao Yu Yeo , Andrew H. Song , Guillaume Jaume , Yuchen Wang , et al. 2025. “A Foundation Model for Spatial Proteomics.” *arXiv*, 10.48550/arXiv.2506.03373

[225] Liu, Linjing , Wei Li , Fang Wang , Yiming Li , Long‐Kai Huang , Ka‐Chun Wong , Fan Yang , and Jianhua Yao . 2025. “A Pre‐Trained Large Generative Model for Translating Single‐Cell Transcriptomes to Proteomes.” Nature Biomedical Engineering 10: 1672–1691. 10.1038/s41551-025-01528-z 41193888

[226] Pang, Jiangshuan , Ping Qiu , Youzhe He , Baolong Li , Yiting Deng , Jun Wang , Adi Lin , et al. 2025. “OmniCell: Unified Foundation Modeling of Single‐Cell and Spatial Transcriptomics for Cellular and Molecular Insights.” *bioRxiv*. 10.64898/2025.12.29.696804

[227] Pang, Kuan , Yanay Rosen , Kasia Kedzierska , Ziyuan He , Abhejit Rajagopal , Claire E. Gustafson , Grace Huynh , and Jure Leskovec . 2025. “PULSAR: A Foundation Model for Multi‐scale and Multicellular Biology.” *bioRxiv*. 10.1101/2025.11.24.685470

[228] Shen, Ronglai , Adam B. Olshen , and Marc Ladanyi . 2009. “Integrative Clustering of Multiple Genomic Data Types Using a Joint Latent Variable Model With Application to Breast and Lung Cancer Subtype Analysis.” Bioinformatics 25: 2906–2912. 10.1093/bioinformatics/btp543 19759197 PMC2800366

[229] Wang, Bo , Aziz M. Mezlini , Feyyaz Demir , Marc Fiume , Zhuowen Tu , Michael Brudno , Benjamin Haibe‐Kains , and Anna Goldenberg . 2014. “Similarity Network Fusion for Aggregating Data Types on a Genomic Scale.” Nature Methods 11: 333–337. 10.1038/nmeth.2810 24464287

[230] Argelaguet, Ricard , Damien Arnol , Danila Bredikhin , Yonatan Deloro , Britta Velten , John C. Marioni , and Oliver Stegle . 2020. “MOFA+: A Statistical Framework for Comprehensive Integration of Multi‐Modal Single‐Cell Data.” Genome Biology 21: 111. 10.1186/s13059-020-02015-1 32393329 PMC7212577

[231] Singh, Amrit , Casey P. Shannon , Benoît Gautier , Florian Rohart , Michaël Vacher , Scott J. Tebbutt , and Kim‐Anh Lê Cao . 2019. “DIABLO: An Integrative Approach for Identifying Key Molecular Drivers From Multi‐Omics Assays.” Bioinformatics 35: 3055–3062. 10.1093/bioinformatics/bty1054 30657866 PMC6735831

[232] Wang, Tongxin , Wei Shao , Zhi Huang , Haixu Tang , Jie Zhang , Zhengming Ding , and Kun Huang . 2021. “MOGONET Integrates Multi‐Omics Data Using Graph Convolutional Networks Allowing Patient Classification and Biomarker Identification.” Nature Communications 12: 3445. 10.1038/s41467-021-23774-w PMC8187432 34103512

[233] Gayoso, Adam , Zoë Steier , Romain Lopez , Jeffrey Regier , Kristopher L. Nazor , Aaron Streets , and Nir Yosef . 2021. “Joint Probabilistic Modeling of Single‐Cell Multi‐Omic Data With totalVI.” Nature Methods 18: 272–282. 10.1038/s41592-020-01050-x 33589839 PMC7954949

[234] Hao, Yuhan , Stephanie Hao , Erica Andersen‐Nissen , William M. Mauck , Shiwei Zheng , Andrew Butler , Maddie J. Lee , et al. 2021. “Integrated Analysis of Multimodal Single‐Cell Data.” Cell 184: 3573–3587. 10.1016/j.cell.2021.04.048 34062119 PMC8238499

[235] Ashuach, Tal , Mariano I. Gabitto , Rohan V. Koodli , Giuseppe‐Antonio Saldi , Michael I. Jordan , and Nir Yosef . 2023. “MultiVI: Deep Generative Model for the Integration of Multimodal Data.” Nature Methods 20: 1222–1231. 10.1038/s41592-023-01909-9 37386189 PMC10406609

[236] Cao, Zhi‐Jie , and Ge Gao . 2022. “Multi‐Omics Single‐Cell Data Integration and Regulatory Inference With Graph‐Linked Embedding.” Nature Biotechnology 40: 1458–1466. 10.1038/s41587-022-01284-4 PMC9546775 35501393

[237] Schuster, Viktoria , Emma Dann , Anders Krogh , and Sarah A. Teichmann . 2024. “multiDGD: A Versatile Deep Generative Model for Multi‐Omics Data.” Nature Communications 15: 10031. 10.1038/s41467-024-53340-z PMC11579284 39567490

[238] Wang, Feng‐ao , Zhenfeng Zhuang , Feng Gao , Ruikun He , Shaoting Zhang , Liansheng Wang , Junwei Liu , and Yixue Li . 2024. “TMO‐Net: An Explainable Pretrained Multi‐Omics Model for Multi‐Task Learning in Oncology.” Genome Biology 25: 149. 10.1186/s13059-024-03293-9 38845006 PMC11157742

[239] Wu, Kevin E. , Kathryn E. Yost , Howard Y. Chang , and James Zou . 2021. “BABEL Enables Cross‐Modality Translation Between Multiomic Profiles at Single‐Cell Resolution.” Proceedings of the National Academy of Sciences 118: e2023070118. 10.1073/pnas.2023070118 PMC8054007 33827925

[240] Cao, Yichuan , Xiamiao Zhao , Songming Tang , Qun Jiang , Sijie Li , Siyu Li , and Shengquan Chen . 2024. “Scbutterfly: A Versatile Single‐Cell Cross‐Modality Translation Method via Dual‐Aligned Variational Autoencoders.” Nature Communications 15: 2973. 10.1038/s41467-024-47418-x PMC10998864 38582890

[241] Lotfollahi, Mohammad , F. Alexander Wolf , and Fabian J. Theis . 2019. “scGen Predicts Single‐Cell Perturbation Responses.” Nature Methods 16: 715–721. 10.1038/s41592-019-0494-8 31363220

[242] Roohani, Yusuf , Kexin Huang , and Jure Leskovec . 2024. “Predicting Transcriptional Outcomes of Novel Multigene Perturbations With GEARS.” Nature biotechnology 42: 927–935. 10.1038/s41587-023-01905-6 PMC11180609 37592036

[243] Dong, Mingze , Bao Wang , Jessica Wei , Antonio H. de O. Fonseca , Curtis J. Perry , Alexander Frey , Feriel Ouerghi , et al. 2023. “Causal Identification of Single‐Cell Experimental Perturbation Effects With CINEMA‐OT.” Nature Methods 20: 1769–1779. 10.1038/s41592-023-02040-5 37919419 PMC10630139

[244] Muneer, Amgad , Muhammad Waqas , Maliazurina B. Saad , Eman Showkatian , Rukhmini Bandyopadhyay , Hui Xu , Wentao Li , et al. 2026. “From Classical Machine Learning to Emerging Foundation Models: Review on Multimodal Data Integration for Cancer Research.” Artificial Intelligence Review 59: 119. 10.1007/s10462-026-11522-9

[245] Chen, Sijie , Yanting Luo , Haoxiang Gao , Fanhong Li , Yixin Chen , Jiaqi Li , Renke You , et al. 2022. “hECA: the Cell‐Centric Assembly of a Cell Atlas.” iScience 25: 104318. 10.1016/j.isci.2022.104318 35602947 PMC9114628

[246] Xi, Xi , Yixin Chen , Xinze Wu , Minsheng Hao , Jiaqi Li , Haiyang Bian , Qiuchen Meng , et al. 2025. “hECA v2.0: An AI‐Ready Ensemble Cell Atlas of Single‐Cell RNA and ATAC Sequencing Data.” Scientific Data 13: 110. 10.1038/s41597-025-06426-2 41398179 PMC12852668

[247] Shen, Hongru , Jilei Liu , Jiani Hu , Xilin Shen , Chao Zhang , Dan Wu , Mengyao Feng , et al. 2023. “Generative Pretraining From Large‐Scale Transcriptomes for Single‐Cell Deciphering.” iScience 26: 106536. 10.1016/j.isci.2023.106536 37187700 PMC10176267

[248] Hao, Minsheng , Jing Gong , Xin Zeng , Chiming Liu , Yucheng Guo , Xingyi Cheng , Taifeng Wang , et al. 2024. “Large‐Scale Foundation Model on Single‐Cell Transcriptomics.” Nature Methods 21: 1481–1491. 10.1038/s41592-024-02305-7 38844628

[249] Chen, Xiaoyang , Keyi Li , Xuejian Cui , Zian Wang , Qun Jiang , Jiacheng Lin , Zhen Li , et al. 2025. “EpiAgent: Foundation Model for Single‐Cell Epigenomics.” Nature Methods 22: 2316–2327. 10.1038/s41592-025-02822-z 40999099

[250] Liu, Guole , Yongbing Zhao , Yingying Zhao , Tianyu Wang , Quanyou Cai , Xiaotao Wang , Ziyi Wen , et al. 2025. “SCARF: Single Cell ATAC‐seq and RNA‐seq Foundation model.” *bioRxiv*. 10.1101/2025.04.07.647689

[251] Schaar, Anna C. , Alejandro Tejada‐Lapuerta , Giovanni Palla , Robert Gutgesell , Lennard Halle , Mariia Minaeva , Larsen Vornholz , et al. 2024. “Nicheformer: A Foundation Model for Single‐Cell and Spatial Omics.” *SSRN* 10.2139/ssrn.4803291 PMC12695652 41168487

[252] Long, Yahui , Kok Siong Ang , Mengwei Li , Kian Long Kelvin Chong , Raman Sethi , Chengwei Zhong , Hang Xu , et al. 2023. “Spatially Informed Clustering, Integration, and Deconvolution of Spatial Transcriptomics With GraphST.” Nature Communications 14: 1155. 10.1038/s41467-023-36796-3 PMC9977836 36859400

[253] Ren, Honglei , Benjamin L. Walker , Zixuan Cang , and Qing Nie . 2022. “Identifying Multicellular Spatiotemporal Organization of Cells With SpaceFlow.” Nature Communications 13: 4076. 10.1038/s41467-022-31739-w PMC9283532 35835774

[254] Long, Yahui , Kok Siong Ang , Raman Sethi , Sha Liao , Yang Heng , Lynn van Olst , Shuchen Ye , et al. 2024. “Deciphering Spatial Domains From Spatial Multi‐Omics With SpatialGlue.” Nature Methods 21: 1658–1667. 10.1038/s41592-024-02316-4 38907114 PMC11399094

[255] Zhou, Yuansheng , Xue Xiao , Lei Dong , Chen Tang , Guanghua Xiao , and Lin Xu . 2025. “Cooperative Integration of Spatially Resolved Multi‐Omics Data With COSMOS.” Nature Communications 16: 27. 10.1038/s41467-024-55204-y PMC11696235 39747840

[256] Xiao, Yongjun , Dian Meng , Xinlei Huang , Yanran Liu , Shiwei Ruan , Ziyue Qiao , and Xubin Zheng . 2025. “GROVER: Graph‐Guided Representation of Omics and Vision With Expert Regulation for Adaptive Spatial Multi‐Omics Fusion.” *arXiv*. 10.48550/arXiv.2511.11730

[257] Blampey, Quentin , Hakim Benkirane , Nadège Bercovici , Kevin Mulder , Grégoire Gessain , Florent Ginhoux , Fabrice André , and Paul‐Henry Cournède . 2025. “Novae: A Graph‐Based Foundation Model for Spatial Transcriptomics Data.” Nature Methods 22: 2539–2550. 10.1038/s41592-025-02899-6 41372623

[258] Wang, Chloe , Haotian Cui , Andrew Zhang , Ronald Xie , Hani Goodarzi , and Wang Bo . 2025. “scGPT‐Spatial: Continual Pretraining of Single‐Cell Foundation Model for Spatial Transcriptomics.” *bioRxiv*. 10.1101/2025.02.05.636714

[259] Zhao, Suyuan , Yizhen Luo , Ganbo Yang , Yan Zhong , Hao Zhou , and Zaiqing Nie . 2025. “Stofm: A Multi‐Scale Foundation Model for Spatial Transcriptomics.” *arXiv*, 10.48550/arXiv.2507.11588

[260] Cao, Shenghao , Kaiyuan Yang , Jiabei Cheng , Jiachen Li , Hong‐Bin Shen , Xiaoyong Pan , and Ye Yuan . 2024. “stFormer: A Foundation Model for Spatial Transcriptomics.” *bioRxiv*, 10.1101/2024.09.27.615337

[261] Park, Jeongbin , Sumin Kim , Jiwon Kim , Dongjoo Lee , Sungwoo Bae , Haenara Shin , Daeseung Lee , and Hongyoon Choi . 2024. “CELLama: Foundation Model for Single Cell and Spatial Transcriptomics by Cell Embedding Leveraging Language Model Abilities.” Advanced Science 13: e13210. 10.1002/advs.202513210 PMC12866849 41243712

[262] Lu, Ming Y. , Bowen Chen , Drew F. K. Williamson , Richard J. Chen , Ivy Liang , Tong Ding , Guillaume Jaume , et al. 2024. “A Visual‐Language Foundation Model for Computational Pathology.” Nature Medicine 30: 863–874. 10.1038/s41591-024-02856-4 PMC11384335 38504017

[263] Ding, Tong , Sophia J. Wagner , Andrew H. Song , Richard J. Chen , Ming Y. Lu , Andrew Zhang , Anurag J. Vaidya , et al. 2025. “A Multimodal Whole‐Slide Foundation Model for Pathology.” Nature Medicine 31: 3749–3761. 10.1038/s41591-025-03982-3 PMC12618242 41193692

[264] Jiang, Rui , Xiaoxu Yin , Pengshuai Yang , Lingchao Cheng , Juan Hu , Jiao Yang , Ying Wang , et al. 2024. “A Transformer‐Based Weakly Supervised Computational Pathology Method for Clinical‐Grade Diagnosis and Molecular Marker Discovery of Gliomas.” Nature Machine Intelligence 6: 876–891. 10.1038/s42256-024-00868-w

[265] Chen, Weiqing , Pengzhi Zhang , Tu N. Tran , Yiwei Xiao , Shengyu Li , Vrutant V. Shah , Hao Cheng , et al. 2025. “A Visual–Omics Foundation Model to Bridge Histopathology With Spatial Transcriptomics.” Nature Methods 22: 1568–1582. 10.1038/s41592-025-02707-1 40442373 PMC12240810

[266] Vaidya, Anurag , Andrew Zhang , Guillaume Jaume , Andrew H. Song , Tong Ding , Sophia J. Wagner , Ming Y. Lu , et al. 2025. “Molecular‐Driven Foundation Model for Oncologic Pathology.” *arXiv*. 10.48550/arXiv.2501.16652

[267] Yan, Rui , Xueyuan Zhang , Zihang Jiang , Baizhi Wang , Xiuwu Bian , Fei Ren , and S Kevin Zhou . 2025. “Pathway‐Aware Multimodal Transformer (PAMT): Integrating Pathological Image and Gene Expression for Interpretable Cancer Survival Analysis.” IEEE Transactions on Pattern Analysis and Machine Intelligence 4: 896–913. 10.1109/TPAMI.2025.3611531 40966149

[268] Liu, Junzhuo , Markus Eckstein , Zhixiang Wang , Friedrich Feuerhake , and Dorit Merhof . 2026. “Spatial Transcriptomics Expression Prediction From Histopathology Based on Cross‐Modal Mask Reconstruction and Contrastive Learning.” Medical Image Analysis 108: 103889. 10.1016/j.media.2025.103889 41330094

[269] Zhang, Pengzhi , Weiqing Chen , Tu N. Tran , Minghao Zhou , Kaylee N. Carter , Ibrahem Kandel , Shengyu Li , et al. 2025. “Thor: A Platform for Cell‐Level Investigation of Spatial Transcriptomics and Histology.” Nature Communications 16: 7178. 10.1038/s41467-025-62593-1 PMC12325965 40764306

[270] Carrillo‐Perez, Francisco , Marija Pizurica , Yuanning Zheng , Tarak Nath Nandi , Ravi Madduri , Jeanne Shen , and Olivier Gevaert . 2025. “Generation of Synthetic Whole‐Slide Image Tiles of Tumours From RNA‐Sequencing Data via Cascaded Diffusion Models.” Nature Biomedical Engineering 9: 320–332. 10.1038/s41551-024-01193-8 38514775

[271] Klein, Dominik , Jonas Simon Fleck , Daniil Bobrovskiy , Lea Zimmermann , Sören Becker , Alessandro Palma , Leander Dony , et al. 2025. “CellFlow Enables Generative Single‐Cell Phenotype Modeling With Flow Matching.” *bioRxiv*. 10.1101/2025.04.11.648220

[272] Zhu, Ouyang , and Jun Li . 2025. “Scouter Predicts Transcriptional Responses to Genetic Perturbations With Large Language Model Embeddings.” Nature Computational Science 6: 21–28. 10.1038/s43588-025-00912-8 41350457 PMC12855003

[273] Tang, Xiangru , Zhuoyun Yu , Jiapeng Chen , Yan Cui , Daniel Shao , Weixu Wang , and Fang Wu , et al. 2025. “CellForge: Agentic Design of ‘Virtual Cell Models’.” *arXiv*. 10.48550/arXiv.2508.02276

[274] Dalla‐Torre, Hugo , Liam Gonzalez , Javier Mendoza‐Revilla , Nicolas Lopez Carranza , Adam Henryk Grzywaczewski , Francesco Oteri , Christian Dallago , et al. 2025. “Nucleotide Transformer: Building and Evaluating Robust Foundation Models for Human Genomics.” Nature Methods 22: 287–297. 10.1038/s41592-024-02523-z 39609566 PMC11810778

[275] Avsec, Žiga , Vikram Agarwal , Daniel Visentin , Joseph R. Ledsam , Agnieszka Grabska‐Barwinska , Kyle R. Taylor , Yannis Assael , et al. 2021. “Effective Gene Expression Prediction From Sequence by Integrating Long‐Range Interactions.” Nature Methods 18: 1196–1203. 10.1038/s41592-021-01252-x 34608324 PMC8490152

[276] Nguyen, Eric , Michael Poli , Marjan Faizi , Armin Thomas , Michael Wornow , Callum Birch‐Sykes , Stefano Massaroli , et al. 2023. “HyenaDNA: Long‐Range Genomic Sequence Modeling at Single Nucleotide Resolution.” Advances in Neural Information Processing Systems 36: 43177–43201. 10.52202/075280-1872

[277] Nguyen, Eric , Michael Poli , Matthew G. Durrant , Brian Kang , Dhruva Katrekar , David B. Li , Liam J. Bartie , et al. 2024. “Sequence Modeling and Design From Molecular to Genome Scale With Evo.” Science 386: eado9336. 10.1126/science.ado9336 39541441 PMC12057570

[278] Avsec, Žiga , Natasha Latysheva , Jun Cheng , Guido Novati , Kyle R. Taylor , Tom Ward , Clare Bycroft , et al. 2026. “Advancing Regulatory Variant Effect Prediction With AlphaGenome.” Nature 649: 1206–1218. 10.1038/s41586-025-10014-0 41606153 PMC12851941

[279] Lin, Adi , Bin Xie , Cheng Ye , Cheng Wang , Duoyuan Chen , Ercheng Wang , Fanfeng Lu , et al. 2025. “Genos: A Human‐Centric Genomic Foundation Model.” GigaScience 14: giaf132. 10.1093/gigascience/giaf132 41122975 PMC12755919

[280] Angermueller, Christof , Heather J. Lee , Wolf Reik , and Oliver Stegle . 2017. “DeepCpG: Accurate Prediction of Single‐Cell DNA Methylation States Using Deep Learning.” Genome Biology 18: 67. 10.1186/s13059-017-1189-z 28395661 PMC5387360

[281] Deng, Yuzhong , Jianxiong Tang , Jiyang Zhang , Jianxiao Zou , Que Zhu , and Shicai Fan . 2023. “GraphCpG: Imputation of Single‐Cell Methylomes Based on Locus‐Aware Neighboring Subgraphs.” Bioinformatics 39: btad533. 10.1093/bioinformatics/btad533 37647650 PMC10516632

[282] Zhou, Jiyun , Chongyuan Luo , Hanqing Liu , Matthew G Heffel , Richard E. Straub , Joel E. Kleinman , Thomas M. Hyde , et al. 2024. “scMeFormer: A Transformer‐Based Deep Learning Model for Imputing DNA Methylation States in Single Cells Enhances the Detection of Epigenetic Alterations in Schizophrenia.” *bioRxiv*, 10.1101/2024.01.25.577200 PMC11960545 39986279

[283] Gao, Zijing , Qiao Liu , Wanwen Zeng , Rui Jiang , and Wing Hung Wong . 2024. “EpiGePT: A Pretrained Transformer‐Based Language Model for Context‐Specific Human Epigenomics.” Genome Biology 25: 310. 10.1186/s13059-024-03449-7 39696471 PMC11657395

[284] Fu, Xi , Shentong Mo , Alejandro Buendia , Anouchka P. Laurent , Anqi Shao , Maria del Mar Alvarez‐Torres , Tianji Yu , et al. 2025. “A Foundation Model of Transcription Across Human Cell Types.” Nature 637: 965–973. 10.1038/s41586-024-08391-z 39779852 PMC11754112

[285] Quake, Stephen R . 2021. “The Cell as a Bag of RNA.” Trends In Genetics 37: 1064–1068. 10.1016/j.tig.2021.08.003 34462156

[286] Zhang, Ruiyi , Yunan Luo , Jianzhu Ma , Ming Zhang , and Sheng Wang . 2022. “Scpretrain: Multi‐Task Self‐Supervised Learning for Cell‐Type Classification.” Bioinformatics 38: 1607–1614. 10.1093/bioinformatics/btac007 34999749

[287] Yang, Fan , Wenchuan Wang , Fang Wang , Yuan Fang , Duyu Tang , Junzhou Huang , Hui Lu , and Jianhua Yao . 2022. “scBERT as a Large‐Scale Pretrained Deep Language Model for Cell Type Annotation of Single‐Cell RNA‐seq Data.” Nature Machine Intelligence 4: 852–866. 10.1038/s42256-022-00534-z

[288] Theodoris, Christina V. , Ling Xiao , Anant Chopra , Mark D. Chaffin , Zeina R. Al Sayed , Matthew C. Hill , Helene Mantineo , et al. 2023. “Transfer Learning Enables Predictions in Network Biology.” Nature 618: 616–624. 10.1038/s41586-023-06139-9 37258680 PMC10949956

[289] Zeng, Yuansong , Jiancong Xie , Ningyuan Shangguan , Zhuoyi Wei , Wenbing Li , Yun Su , Shuangyu Yang , et al. 2025. “CellFM: A Large‐Scale Foundation Model Pre‐Trained on Transcriptomics of 100 Million Human Cells.” Nature Communications 16: 4679. 10.1038/s41467-025-59926-5 PMC12092794 40393991

[290] Wen, Hongzhi , Wenzhuo Tang , Xinnan Dai , Jiayuan Ding , Wei Jin , Yuying Xie , and Jiliang Tang . 2023. “Cellplm: Pre‐training of Cell Language Model Beyond Single Cells.” *bioRxiv*, 10.1101/2023.10.03.560734

[291] Bian, Haiyang , Yixin Chen , Xiaomin Dong , Chen Li , Minsheng Hao , Sijie Chen , Jinyi Hu , et al. 2024. “scMulan: A Multitask Generative Pre‐Trained Language Model for Single‐Cell Analysis.” Research in Computational Molecular Biology 2024: 479–482. 10.1007/978-1-0716-3989-4_57

[292] Dong, Mingze , Abhinav Adduri , Dhruv Gautam , Christopher Carpenter , Rohan Shah , Chiara Ricci‐Tam , Yuval Kluger , Dave P. Burke , and Yusuf H. Roohani . 2026. “Stack: In‐Context Learning of Single‐Cell Biology.” *bioRxiv*. 10.64898/2026.01.09.698608

[293] Levine, Daniel , Syed Asad Rizvi , Sacha Lévy , Nazreen Pallikkavaliyaveetil , David Zhang , Xingyu Chen , Sina Ghadermarzi , et al. 2024. “Cell2Sentence: Teaching Large Language Models the Language of Biology.” *bioRxiv*. 10.1101/2023.09.11.557287

[294] Rizvi, Syed Asad , Daniel Levine , Aakash Patel , Shiyang Zhang , Eric Wang , Curtis Jamison Perry , Ivan Vrkic , et al. 2026. “Scaling Large Language Models for Next‐Generation Single‐Cell Analysis.” *bioRxiv*. 10.1101/2025.04.14.648850

[295] Gao, Yicheng , Weixu Wang , Yuheng Zhao , Kejing Dong , Caihua Shan , Weizhong Zheng , Till Richter , et al. 2025. “Language May be All Omics Needs: Harmonizing Multimodal Data for Omics Understanding With CellHermes.” *bioRxiv*. 10.1101/2025.11.07.687322

[296] Chen, Yiqun , and James Zou . 2024. “Genepert: Leveraging Genept Embeddings for Gene Perturbation Prediction.” *bioRxiv*. 10.1101/2024.10.27.620513

[297] Elnaggar, Ahmed , Michael Heinzinger , Christian Dallago , Ghalia Rehawi , Yu Wang , Llion Jones , Tom Gibbs , et al. 2022. “ProtTrans: Toward Understanding the Language of Life Through Self‐Supervised Learning.” IEEE Transactions on Pattern Analysis and Machine Intelligence 44: 7112–7127. 10.1109/tpami.2021.3095381 34232869

[298] Rives, Alexander , Joshua Meier , Tom Sercu , Siddharth Goyal , Zeming Lin , Jason Liu , Demi Guo , et al. 2021. “Biological Structure and Function Emerge From Scaling Unsupervised Learning to 250 Million Protein Sequences.” Proceedings of the National Academy of Sciences of the United States of America 118: e2016239118. 10.1073/pnas.2016239118 33876751 PMC8053943

[299] Baek, Minkyung , Frank DiMaio , Ivan Anishchenko , Justas Dauparas , Sergey Ovchinnikov , Gyu Rie Lee , Jue Wang , et al. 2021. “Accurate Prediction of Protein Structures and Interactions Using a Three‐Track Neural Network.” Science 373: 871–876. 10.1126/science.abj8754 34282049 PMC7612213

[300] Hayes, Thomas , Roshan Rao , Halil Akin , Nicholas J. Sofroniew , Deniz Oktay , Zeming Lin , Robert Verkuil , et al. 2025. “Simulating 500 Million Years of Evolution With a Language Model.” Science 387: 850–858. 10.1126/science.ads0018 39818825

[301] Ferruz, Noelia , Steffen Schmidt , and Birte Höcker . 2022. “ProtGPT2 Is a Deep Unsupervised Language Model for Protein Design.” Nature Communications 13: 4348. 10.1038/s41467-022-32007-7 PMC9329459 35896542

[302] Zhang, Ningyu , Zhen Bi , Xiaozhuan Liang , Siyuan Cheng , Haosen Hong , Shumin Deng , Jiazhang Lian , Qiang Zhang , and Huajun Chen . 2022. “Ontoprotein: Protein Pretraining With Gene Ontology Embedding.” *arXiv* 10.48550/arXiv.2201.11147

[303] Queen, Owen , Yepeng Huang , Robert Calef , Valentina Giunchiglia , Tianlong Chen , George Dasoulas , LeAnn Tai , et al. 2025. “Procyon: A Multimodal Foundation Model for Protein Phenotypes.” *bioRxiv*. 10.1101/2024.12.10.627665

[304] Jiang, Kaiyi , Zhaoqing Yan , Matteo Di Bernardo , Samantha R. Sgrizzi , Lukas Villiger , Alisan Kayabolen , B. J. Kim , et al. 2025. “Rapid In Silico Directed Evolution by a Protein Language Model With EVOLVEpro.” Science 387: eadr6006. 10.1126/science.adr6006 39571002

[305] Li, Wei , Fan Yang , Fang Wang , Yu Rong , Linjing Liu , Bingzhe Wu , Han Zhang , and Jianhua Yao . 2024. “scPROTEIN: A Versatile Deep Graph Contrastive Learning Framework for Single‐Cell Proteomics Embedding.” Nature Methods 21: 623–634. 10.1038/s41592-024-02214-9 38504113

[306] Qiu, Shizheng , Jirui Guo , Zhishuai Zhang , Haozheng Liang , Huanyu You , Yang Hu , Guiyou Liu , and Yadong Wang . 2025. “MetaboLM: A Metabolomic Language Model for Multi‐Disease Early Prediction and Risk Stratification.” Nature Communications 16: 11272. 10.1038/s41467-025-66163-3 PMC12717070 41339308

[307] Wu, Dan‐Ni , Joey Jen , Erickson Fajiculay , Min‐Fen Hsu , Ming‐Chu Chang , Jen‐Chen Yeh , Karen Sargsyan , et al. 2026. “PanMETAI—A High Performance Tabular Foundation Model for Accurate Pancreatic Cancer Diagnosis via NMR Metabolomics.” Nature Communications 17: 1595. 10.1038/s41467-026-69426-9 PMC12905243 41688460

[308] Lu, Yuxing . 2025. “Knowledge Graph and Large Language Model for Metabolomics.” Proceedings of the AAAI Conference on Artificial Intelligence 39: 29281–29282. 10.1609/aaai.v39i28.35218

[309] Zhang, Haohong , Yuli Zhang , Zixin Kang , Jiayun Xiong , Ronghua Yang , and Kang Ning . 2026. “MGM as a Large‐Scale Pretrained Foundation Model for Microbiome Analyses in Diverse Contexts.” Advanced Science 13: e13333. 10.1002/advs.202513333 41580987 PMC13116254

[310] Medearis, Nicholas A. , Siyao Zhu , and Ali R. Zomorrodi . 2026. “BiomeGPT: A Foundation Model for the Human Gut Microbiome.” *bioRxiv*. 10.64898/2026.01.05.697599

[311] Pope, Quintin , Rohan Varma , Christine Tataru , Maude M. David , and Xiaoli Fern . 2025. “Learning A Deep Language Model for Microbiomes: The Power of Large Scale Unlabeled Microbiome Data.” PLOS Computational Biology 21: e1011353. 10.1371/journal.pcbi.1011353 40334224 PMC12058177

[312] Gollwitzer, Arvid E. , Adrián Noriega de la Colina , Deepak A. Subramanian , and Giovanni Traverso . “FINGERS‐7B: A Multi‐Omic Foundation Model for Precision Biomarker Discovery.” *ICLR 2026 Workshop on Foundation Models for Science: Real‐World Impact and Science‐First Design*. https://openreview.net/forum?id=fVqvRQ6XRV

[313] Fang, Chao , Fangming Yang , Hao Hou , Huahui Ren , Huanzi Zhong , Huinan Xu , Jiahao Zhang , et al. 2026. “Genos‐m: A Foundation Model for Human‐Associated Microbial Genomes.” *bioRxiv*. 10.64898/2026.05.21.726868

[314] Munsamy, Geraldene , Gavin Ayres , Carla Greco , Daniel Anderson , Srijani Sridhar , William Chow , Aaron W. Kollasch , et al. 2026. “Scaling Laws and Architectural Frontiers in Metagenomic Foundation Models.” Generative AI in Genomics (Gen2): Barriers and Frontiers. https://openreview.net/forum?id=1ZtaoMcOTN

[315] Gupta, Ankit , Zoe Wefers , Konstantin Kahnert , Jan N. Hansen , Mohini K. Misra , Will Leineweber , Anthony Cesnik , et al. 2025. “SubCell: Proteome‐Aware Vision Foundation Models for Microscopy Capture Single‐Cell Biology.” *bioRxiv*. 10.1101/2024.12.06.627299

[316] Ozturk, Efe , Felix G. Rivera Moctezuma , and Ahmet F. Coskun . 2025. “MetaboFM: A Foundation Model for Spatial Metabolomics.” *bioRxiv*. 10.1101/2025.10.23.684227

[317] Mataraso, Samson J. , Camilo A. Espinosa , David Seong , S. Momsen Reincke , Eloise Berson , Jonathan D. Reiss , Yeasul Kim , et al. 2025. “A Machine Learning Approach to Leveraging Electronic Health Records for Enhanced Omics Analysis.” Nature Machine Intelligence 7: 293–306. 10.1038/s42256-024-00974-9 PMC11847705 40008295

[318] Paverd, Hania , Konstantinos Zormpas‐Petridis , Hannah Clayton , Sarah Burge , and Mireia Crispin‐Ortuzar . 2024. “Radiology and Multi‐Scale Data Integration for Precision Oncology.” npj Precision Oncology 8: 158. 10.1038/s41698-024-00656-0 39060351 PMC11282284

[319] Boehm, Kevin M. , Pegah Khosravi , Rami Vanguri , Jianjiong Gao , and Sohrab P. Shah . 2021. “Harnessing Multimodal Data Integration to Advance Precision Oncology.” Nature Reviews Cancer 22: 114–126. 10.1038/s41568-021-00408-3 34663944 PMC8810682

[320] Lipkova, Jana , Richard J. Chen , Bowen Chen , Ming Y. Lu , Matteo Barbieri , Daniel Shao , Anurag J. Vaidya , et al. 2022. “Artificial Intelligence for Multimodal Data Integration in Oncology.” Cancer Cell 40: 1095–1110. 10.1016/j.ccell.2022.09.012 36220072 PMC10655164

[321] Zhao, Mengyuan , Xinyue Tang , Jiawei Li , Cheng Liang , Jijun Tang , and Fei Guo . 2026. “MultiPert: An Adversarial Alignment and Dual Attention Framework for Single‐Cell Multi‐Omics Perturbation Prediction.” PLOS Computational Biology 22: e1014054. 10.1371/journal.pcbi.1014054 41811907 PMC12998955

[322] Ji, Yuge , Mohammad Lotfollahi , F. Alexander Wolf , and Fabian J. Theis . 2021. “Machine Learning for Perturbational Single‐Cell Omics.” Cell Systems 12: 522–537. 10.1016/j.cels.2021.05.016 34139164

[323] Gandhi, Shreshth , Farnoosh Javadi , Valentine Svensson , Umair Khan , Matthew G. Jones , John Yu , Daniele Merico , Hani Goodarzi , and Nima Alidoust . 2025. “Tahoe‐x1: Scaling perturbation‐Trained Single‐Cell Foundation Models to 3 billion Parameters.” *bioRxiv*, 10.1101/2025.10.23.683759

[324] Cui, Haotian , Alejandro Tejada‐Lapuerta , Maria Brbić , Julio Saez‐Rodriguez , Simona Cristea , Hani Goodarzi , Mohammad Lotfollahi , Fabian J. Theis , and Bo Wang . 2025. “Towards Multimodal Foundation Models in Molecular Cell Biology.” Nature 640: 623–633. 10.1038/s41586-025-08710-y 40240854

[325] Wu, You , and Lei Xie . 2025. “AI‐Driven Multi‐Omics Integration for Multi‐Scale Predictive Modeling of Genotype‐Environment‐Phenotype Relationships.” Computational and Structural Biotechnology Journal 27: 265–277. 10.1016/j.csbj.2024.12.030 39886532 PMC11779603

[326] Huang, Tinglin , Tianyu Liu , Mehrtash Babadi , Rex Ying , and Wengong Jin . 2025. “STPath: A Generative Foundation Model for Integrating Spatial Transcriptomics and Whole‐Slide Images.” npj Digital Medicine 8: 659. 10.1038/s41746-025-02020-3 41238784 PMC12618518

[327] Fallahpour, Adibvafa , Andrew Magnuson , Purav Gupta , Shihao Ma , Jack Naimer , Arnav Shah , Haonan Duan , et al. 2025. “BioReason: Incentivizing Multimodal Biological Reasoning Within a DNA‐LLM Model.” Advances in Neural Information Processing Systems 38: 111644–111671. 10.48550/arXiv.2505.23579

[328] Toussaint, Philipp A. , Florian Leiser , Scott Thiebes , Matthias Schlesner , Benedikt Brors , and Ali Sunyaev . 2024. “Explainable Artificial Intelligence for Omics Data: A Systematic Mapping Study.” Briefings in Bioinformatics 25: bbad453. 10.1093/bib/bbad453 PMC10729786 38113073

[329] Kuenzi, Brent M. , Jisoo Park , Samson H. Fong , Kyle S. Sanchez , John Lee , Jason F. Kreisberg , Jianzhu Ma , and Trey Ideker . 2020. “Predicting Drug Response and Synergy Using a Deep Learning Model of Human Cancer Cells.” Cancer Cell 38: 672–684.e6. 10.1016/j.ccell.2020.09.014 33096023 PMC7737474

[330] Wei, Zhijian , Runze Ma , Zichen Wang , Zhongmin Li , Shuotong Song , and Shuangjia Zheng . 2025. “VCWorld: A Biological World Model for Virtual Cell Simulation.” *arXiv*, 10.48550/arXiv.2512.00306

[331] Chevalley, Mathieu , Yusuf H. Roohani , Arash Mehrjou , Jure Leskovec , and Patrick Schwab . 2025. “A Large‐Scale Benchmark for Network Inference From Single‐Cell Perturbation Data.” Communications Biology 8: 412. 10.1038/s42003-025-07764-y 40069299 PMC11897147

[332] Yiu, Taylor , Bin Chen , Haoyu Wang , Genyi Feng , Qiangqiang Fu , and Huijing Hu . 2025. “Transformative Advances in Single‐Cell Omics: A Comprehensive Review of Foundation Models, Multimodal Integration and Computational Ecosystems.” Journal of Translational Medicine 23: 1176. 10.1186/s12967-025-07091-0 41146276 PMC12560279

[333] Kato, Takahiro , Daisuke Komura , Binay Panda , and Shumpei Ishikawa . 2025. “Medicine for Artificial Intelligence: Applying a Medical Framework to AI Anomalies.” Frontiers in Artificial Intelligence 8: 1698717. 10.3389/frai.2025.1698717 41141908 PMC12546293

[334] Wouters, Olivier J. , Martin McKee , and Jeroen Luyten . 2020. “Estimated Research and Development Investment Needed to Bring a New Medicine to Market, 2009‐2018.” JAMA 323: 844–853. 10.1001/jama.2020.1166 32125404 PMC7054832

[335] Philippidis, Alex . 2025. “Top 10 Best‐Selling Drugs: Total Aggregate Value of the Drugs Generating the Highest Sales in 2024 Rose 6.5% vs. 2023, and Jumped 53% Over Five years.” Genetic Engineering & Biotechnology News 45: 14–15. 10.1089/gen.45.07.07

[336] Brown, Dean G. , and Heike J. Wobst . 2021. “A Decade of FDA‐Approved Drugs (2010–2019): Trends and Future Directions.” Journal of Medicinal Chemistry 64: 2312–2338. 10.1021/acs.jmedchem.0c01516 33617254

[337] Seoane‐Vazquez, Enrique , Rosa Rodriguez‐Monguio , Sheryl L. Szeinbach , and Jay Visaria . 2008. “Incentives for Orphan Drug Research and Development in the United States.” Orphanet Journal of Rare Diseases 3: 33. 10.1186/1750-1172-3-33 19087348 PMC2631478

[338] Ringel, Michael S. , Jack W. Scannell , Mathias Baedeker , and Ulrik Schulze . 2020. “Breaking Eroom's Law.” Nature Reviews Drug Discovery 19: 833–834. 10.1038/d41573-020-00059-3 32300238

[339] Khanna, Ish . 2012. “Drug Discovery in Pharmaceutical Industry: Productivity Challenges and Trends.” Drug Discovery Today 17: 1088–1102. 10.1016/j.drudis.2012.05.007 22627006

[340] Morabito, Aurelia , Giulia De Simone , Roberta Pastorelli , Laura Brunelli , and Manuela Ferrario . 2025. “Algorithms and Tools for Data‐Driven Omics Integration to Achieve Multilayer Biological Insights: A Narrative Review.” Journal of Translational Medicine 23: 425. 10.1186/s12967-025-06446-x 40211300 PMC11987215

[341] Alaseem, Ali M. , Glowi Alasiri , Arockia Babu Marianesan , Thakur Gurjeet Singh , Prawez Alam , Mohammad Fareed , and Nisha Bansal . 2025. “Target‐Specific Potency and Drug‐Ability Profile of Flavonoids Against Lung Cancer: An Integrative Multi‐Omics Approach for Lead Identification.” Drug Development Research 86: e70131. 10.1002/ddr.70131 40741887

[342] Keshri, Vivek , and Jyothi Belurappa . 2025. “Multi‐omics Applications in Drug Discovery and Development.” In Multi‐Omics in Biomedical Sciences and Environmental Sustainability: Applications and Recent Advances, 143–158. Springer Nature Singapore. 10.1007/978-981-96-7067-3_5

[343] Nguyen, Nhan , Danyel Jennen , and Jos Kleinjans . 2022. “Omics Technologies to Understand Drug Toxicity Mechanisms.” Drug Discovery Today 27: 103348. 10.1016/j.drudis.2022.103348 36089240

[344] Glaab, Enrico , Armin Rauschenberger , Rita Banzi , Chiara Gerardi , Paula Garcia , and Jacques Demotes . 2021. “Biomarker Discovery Studies for Patient Stratification Using Machine Learning Analysis of Omics Data: a Scoping Review.” BMJ Open 11: e053674. 10.1136/bmjopen-2021-053674 PMC8650485 34873011

[345] Wang, Richard C. , and Zhixiang Wang . 2023. “Precision Medicine: Disease Subtyping and Tailored Treatment.” Cancers 15: 3837. 10.3390/cancers15153837 37568653 PMC10417651

[346] Matthews, Holly , James Hanison , and Niroshini Nirmalan . 2016. “ ‘Omics’‐Informed Drug and Biomarker Discovery: Opportunities, Challenges and Future Perspectives.” Proteomes 4: 28. 10.3390/proteomes4030028 28248238 PMC5217350

[347] Zielinski, Jessica M. , Jason J. Luke , Silvia Guglietta , and Carsten Krieg . 2021. “High Throughput Multi‐Omics Approaches for Clinical Trial Evaluation and Drug Discovery.” Frontiers in Immunology 12: 590742. 10.3389/fimmu.2021.590742 33868223 PMC8044891

[348] Mandal, Soma , Mee'nal Moudgil , and Sanat K. Mandal . 2009. “Rational Drug Design.” European Journal of Pharmacology 625: 90–100. 10.1016/j.ejphar.2009.06.065 19835861

[349] Zheng, Wei , Natasha Thorne , and John C. McKew . 2013. “Phenotypic Screens as a Renewed Approach for Drug Discovery.” Drug Discovery Today 18: 1067–1073. 10.1016/j.drudis.2013.07.001 23850704 PMC4531371

[350] Plenge, Robert M. , Edward M. Scolnick , and David Altshuler . 2013. “Validating Therapeutic Targets Through Human Genetics.” Nature Reviews Drug Discovery 12: 581–594. 10.1038/nrd4051 23868113

[351] Minikel, Eric Vallabh , Jeffery L. Painter , Coco Chengliang Dong , and Matthew R. Nelson . 2024. “Refining the Impact of Genetic Evidence on Clinical Success.” Nature 629: 624–629. 10.1038/s41586-024-07316-0 38632401 PMC11096124

[352] Norton, Nadine , Duanxiang Li , Mark J. Rieder , Jill D. Siegfried , Evadnie Rampersaud , Stephan Züchner , Steve Mangos , et al. 2011. “Genome‐Wide Studies of Copy Number Variation and Exome Sequencing Identify Rare Variants in BAG3 as a Cause of Dilated Cardiomyopathy.” American Journal of Human Genetics 88: 273–282. 10.1016/j.ajhg.2011.01.016 21353195 PMC3059419

[353] ClinicalTrials.gov . March 10, 2026. “A Phase 1 AAV Gene Therapy Trial Evaluating Safety and Preliminary Efficacy of RP‐A701 in Subjects With BAG3 Dilated Cardiomyopathy.” ClinicalTrials.gov., NCT07137338. https://clinicaltrials.gov/study/NCT07137338

[354] Chen, Robert , Áine Duffy , and Ron Do . 2026. “Genomics of Drug Target Prioritization for Complex Diseases.” Nature Reviews Genetics 27: 231–245. 10.1038/s41576-025-00904-4 PMC12816132 41198831

[355] Yang, Jing , Jin Xu , Wei Wang , Bo Zhang , Xianjun Yu , and Si Shi . 2023. “Epigenetic Regulation in the Tumor Microenvironment: Molecular Mechanisms and Therapeutic Targets.” Signal Transduction and Targeted Therapy 8: 210. 10.1038/s41392-023-01480-x 37217462 PMC10203321

[356] Vijaykrishnaraj, M. , Prakash Patil , Sudeep D. Ghate , Adithi K. Bhandary , and Vikram M. Haridas . 2023. “Efficacy of HDAC Inhibitors and Epigenetic Modulation in the Amelioration of Synovial Inflammation, Cellular Invasion, and Bone Erosion in Rheumatoid Arthritis Pathogenesis.” International Immunopharmacology 122: 110644. 10.1016/j.intimp.2023.110644 37454631

[357] Kim, Mirang . 2019. “DNA Methylation: A Cause and Consequence of Type 2 Diabetes.” Genomics & Informatics 17: e38. 10.5808/GI.2019.17.4.e38 31896238 PMC6944052

[358] Rasooly, Danielle , Alexandre C. Pereira , and Jacob Joseph . 2025. “Drug Discovery and Development for Heart Failure Using Multi‐Omics Approaches.” International Journal of Molecular Sciences 26: 2703. 10.3390/ijms26062703 40141349 PMC11943351

[359] Tarazona, Sonia , Fernando García‐Alcalde , Joaquín Dopazo , Alberto Ferrer , and Ana Conesa . 2011. “Differential Expression in RNA‐seq: A Matter of Depth.” Genome Research 21: 2213–2223. 10.1101/gr.124321.111 21903743 PMC3227109

[360] Loers, Jens Uwe , and Vanessa Vermeirssen . 2024. “A Single‐Cell Multimodal View on Gene Regulatory Network Inference From Transcriptomics and Chromatin Accessibility Data.” Briefings in Bioinformatics 25: bbae382. 10.1093/bib/bbae382 39207727 PMC11359808

[361] Davis, Andrew M. , Simon J. Teague , and Gerard J. Kleywegt . 2003. “Application and Limitations of X‐Ray Crystallographic Data in Structure‐Based Ligand and Drug Design.” Angewandte Chemie International Edition 42: 2718–2736. 10.1002/anie.200200539 12820253

[362] Lee, Yie Hou , Hwee Tong Tan , and Maxey C. M. Chung . 2010. “Subcellular Fractionation Methods and Strategies for Proteomics.” Proteomics 10: 3935–3956. 10.1002/pmic.201000289 21080488

[363] Qin, Jinfang , Shun Zhang , Yongyi Pan , Xiaocan Lei , Zhenwei Guo , Qin Xie , Kongwei Huang , and Peng Huo . 2025. “Progress and Application of Activity‐Based Protein Profiling for the Discovery of Natural Product Targets.” Molecular Diversity 30: 3175–3190. 10.1007/s11030-025-11361-w 40986277

[364] Attwood, Misty M. , Doriano Fabbro , Aleksandr V. Sokolov , Stefan Knapp , and Helgi B. Schiöth . 2021. “Trends in Kinase Drug Discovery: Targets, Indications and Inhibitor Design.” Nature Reviews Drug Discovery 20: 839–861. 10.1038/s41573-021-00252-y 34354255

[365] Li, Yang , Mahlon Collins , Jiyan An , Rachel Geiser , Tony Tegeler , Kristine Tsantilas , Krystine Garcia , Patrick Pirrotte , and Robert Bowser . 2016. “Immunoprecipitation and Mass Spectrometry Defines an Extensive RBM45 Protein–Protein Interaction Network.” Brain Research 1647: 79–93. 10.1016/j.brainres.2016.02.047 26979993 PMC5152586

[366] Lee, Wen‐Chien , and Kelvin H. Lee . 2004. “Applications of Affinity Chromatography in Proteomics.” Analytical Biochemistry 324: 1–10. 10.1016/j.ab.2003.08.031 14654038

[367] Hanson, Caroline M. , Dina L. Bai , and Jarrod A. Marto . 2026. “Navigating Challenges in Mass Spectrometry Analysis of Endogenous and Synthetic Protein Modifications.” Biomolecules 16: 367. 10.3390/biom16030367 41897302 PMC13024198

[368] Ganapathy‐Kanniappan, Shanmugasundaram , and Jean‐Francois H. Geschwind . 2013. “Tumor Glycolysis as a Target for Cancer Therapy: Progress and Prospects.” Molecular Cancer 12: 152. 10.1186/1476-4598-12-152 24298908 PMC4223729

[369] Ruiz, Matthieu , François Labarthe , Annik Fortier , Bertrand Bouchard , Julie Thompson Legault , Virginie Bolduc , Odile Rigal , et al. 2017. “Circulating Acylcarnitine Profile in Human Heart Failure: a Surrogate of Fatty Acid Metabolic Dysregulation in Mitochondria and Beyond.” American Journal of Physiology‐Heart and Circulatory Physiology 313: H768–H781. 10.1152/ajpheart.00820.2016 28710072

[370] Pan, Shanshan , Luan Yin , Jie Liu , Jie Tong , Zichuan Wang , Jiahui Zhao , Xuesong Liu , et al. 2024. “Metabolomics‐Driven Approaches for Identifying Therapeutic Targets in Drug Discovery.” MedComm 5: e792. 10.1002/mco2.792 39534557 PMC11555024

[371] Xu, Xiaoqian , Yuzhou Bao , Qi Zhang , Lixia Wang , and Hao Wang . 2026. “sc‐eQTL Unveil Immunogenetic Architecture of Polycystic Ovary Syndrome.” Scientific Reports 16: 4462. 10.1038/s41598-025-34726-5 41495349 PMC12864734

[372] Maria, Maleeha , Negar Pouyanfar , Tiit Örd , and Minna U. Kaikkonen . 2022. “The Power of Single‐Cell RNA Sequencing in eQTL Discovery.” Genes 13: 502. 10.3390/genes13030502. https://www.mdpi.com/2073-4425/13/3/502 35328055 PMC8949403

[373] Du, Yanhong , Yao Gao , Xinzhe Du , Ting An , Hong Zhang , Binhong Wang , Hongbao Cao , et al. 2025. “Further Elucidation of GMPPB as a Risk Gene for Depression Through Integrative Multi‐Omics Analyses.” Journal of Affective Disorders 384: 23–46. 10.1016/j.jad.2025.05.013 40334866

[374] Liu, Jingjing , Wanghui Jing , Tianyu Wang , Zhe Hu , and Haitao Lu . 2023. “Functional Metabolomics Revealed the Dual‐Activation of cAMP‐AMP Axis Is a Novel Therapeutic Target of Pancreatic Cancer.” Pharmacological Research 187: 106554. 10.1016/j.phrs.2022.106554 36379357

[375] Zhang, Anzhuo , Jiawei Zou , Yue Xi , Lianchong Gao , Fulan Deng , Yujun Liu , Pengfei Gao , et al. 2024. “Single‐Cell Technology for Drug Discovery and Development.” Frontiers in Drug Discovery 4: 1459962. 10.3389/fddsv.2024.1459962

[376] Whitehouse, Peter , Sam Gandy , Vikas Saini , Daniel R. George , Eric B. Larson , G. Caleb Alexander , Jerry Avorn , et al. 2022. “Making the Case for Accelerated Withdrawal of Aducanumab.” Journal of Alzheimer's Disease 87: 1003–1007. 10.3233/jad-220262 35404287

[377] G. Wyatt, Paul , Ian H. Gilbert , Kevin D. Read , and Alan H. Fairlamb . 2011. “Target Validation: Linking Target and Chemical Properties to Desired Product Profile.” Current Topics in Medicinal Chemistry 11: 1275–1283. 10.2174/156802611795429185 21401506 PMC3182078

[378] Hu, Qing , Yuan Xiong , Guang‐Hao Zhu , Ya‐Ni Zhang , Yi‐Wen Zhang , Ping Huang , and Guang‐Bo Ge . 2022. “The SARS‐CoV‐2 Main Protease (Mpro): Structure, Function, and Emerging Therapies for COVID‐19.” MedComm 3: e151. 10.1002/mco2.151 35845352 PMC9283855

[379] de Mol, Nico J. , Malcolm B. Gillies , and Marcel J. E. Fischer . 2002. “Experimental and Calculated Shift in pKa Upon Binding of Phosphotyrosine Peptide to the SH2 Domain of p56lck.” Bioorganic & Medicinal Chemistry 10: 1477–1482. 10.1016/S0968-0896(01)00407-2 11886810

[380] Xu, Xinyu , Jeremy Shonberg , Jonas Kaindl , Mary J. Clark , Anne Stößel , Luis Maul , Daniel Mayer , et al. 2023. “Constrained Catecholamines Gain β2AR Selectivity Through Allosteric Effects on Pocket Dynamics.” Nature Communications 14: 2138. 10.1038/s41467-023-37808-y PMC10104803 37059717

[381] Shen, Zhihang , Gustavo Seabra , Jason Brant , Kalyanee Shirlekar , Loic Deleyrolle , Benjamin Lewis , and Chenglong Li . 2025. “Discovery of PRMT5 N‐Terminal TIM Barrel Ligands From Machine‐Learning‐Based Virtual Screening.” ACS Omega 10: 1156–1163. 10.1021/acsomega.4c08661 39829491 PMC11740121

[382] Kamya, Petrina , Ivan V. Ozerov , Frank W. Pun , Kyle Tretina , Tatyana Fokina , Shan Chen , Vladimir Naumov , et al. 2024. “PandaOmics: An AI‐Driven Platform for Therapeutic Target and Biomarker Discovery.” Journal of Chemical Information and Modeling 64: 3961–3969. 10.1021/acs.jcim.3c01619 38404138 PMC11134400

[383] Niazi, Sarfaraz K . 2025. “Quantum Mechanics Paradox in Protein Structure Prediction: Intrinsically Linked to Sequence yet Independent of It.” Computational and Structural Biotechnology Reports 2: 100039. 10.1016/j.csbr.2025.100039

[384] Cabeza de Vaca, Israel , Boris Trapkov , Ling Shen , Duy Duc Vo , Xiaoqun Zhang , Yunting Yang , Mitra Pezeshki , et al. 2025. “Ultra‐Large Virtual Screening Unveils Potent Agonists of the Neuromodulatory Orphan Receptor GPR139.” Nature Communications 17: 129. 10.1038/s41467-025-66845-y PMC12775434 41365886

[385] Nilles, Julie , Dirk Theile , Johanna Weiss , Walter E. Haefeli , and Stephanie Ruez . 2024. “Lack of CYP3A4 Protein Induction Despite mRNA Induction in Primary Hepatocytes Exposed to Rifabutin as a Possible Explanation for Its Low Interaction Risk In Vivo.” Archives of Toxicology 98: 2541–2556. 10.1007/s00204-024-03763-w 38713375

[386] Aliferis, Constantin F. , Alexander Statnikov , and Ioannis Tsamardinos . 2006. “Challenges in the Analysis of Mass‐Throughput Data: A Technical Commentary From the Statistical Machine Learning Perspective.” Cancer Informatics 2: 117693510600200. 10.1177/117693510600200004 PMC2675497 19458765

[387] Thirman, Hannah L. , Madeline J. Grider‐Hayes , Laura C. Geben , Rebecca A. Ihrie , Lauren E. Brown , John A. Porco , and Jonathan M. Irish . 2025. “Connecting Chemical Structure to Single Cell Signaling Profiles.” Communications Biology 8: 1137. 10.1038/s42003-025-08545-3 40745386 PMC12313981

[388] Walters, W.Patrick , Matthew T. Stahl , and Mark A. Murcko . 1998. “Virtual Screening—An Overview.” Drug Discovery Today 3: 160–178. 10.1016/S1359-6446(97)01163-X

[389] Zhou, Guangfeng , Domnita‐Valeria Rusnac , Hahnbeom Park , Daniele Canzani , Hai Minh Nguyen , Lance Stewart , Matthew F. Bush , et al. 2024. “An Artificial Intelligence Accelerated Virtual Screening Platform for Drug Discovery.” Nature Communications 15: 7761. 10.1038/s41467-024-52061-7 PMC11377542 39237523

[390] Wu, Xiaofeng , Menchus Quan , Marco Hadisurya , Jianzhong Hu , Yi‐Kai Liu , Yuxin Zhuang , Li Li , et al. 2024. “Monitoring Drug Metabolic Pathways Through Extracellular Vesicles in Mouse Plasma.” PNAS Nexus 3: pgae023. 10.1093/pnasnexus/pgae023 38312223 PMC10833468

[391] Lipinski, Christopher A . 2004. “Lead‐ and Drug‐Like Compounds: The Rule‐Of‐Five Revolution.” Drug Discovery Today: Technologies 1: 337–341. 10.1016/j.ddtec.2004.11.007 24981612

[392] Lipinski, Christopher A . 2016. “Rule of Five in 2015 and Beyond: Target and Ligand Structural Limitations, Ligand Chemistry Structure and Drug Discovery Project Decisions.” Advanced Drug Delivery Reviews 101: 34–41. 10.1016/j.addr.2016.04.029 27154268

[393] Wang, Zhengyu , Bo‐Syong Pan , Rajesh Kumar Manne , Jungang Chen , Dongwen Lv , Minmin Wang , Phuc Tran , et al. 2025. “CD36‐Mediated Endocytosis of Proteolysis‐Targeting Chimeras.” Cell 188: 3219–3237.e18. 10.1016/j.cell.2025.03.036 40250420 PMC12167158

[394] Bofill, Andreu , Xavier Jalencas , Tudor I. Oprea , and Jordi Mestres . 2019. “The Human Endogenous Metabolome as a Pharmacology Baseline for Drug Discovery.” Drug Discovery Today 24: 1806–1820. 10.1016/j.drudis.2019.06.007 31226432 PMC7748399

[395] Wang, Zhongping , Li Xu , Xinping Zhu , Weigang Cui , Yan Sun , Hisao Nishijo , Yuwen Peng , and Ruixi Li . 2010. “Demethylation of Specific Wnt/β‐Catenin Pathway Genes and Its Upregulation in Rat Brain Induced by Prenatal Valproate Exposure.” Anatomical Record 293: 1947–1953. 10.1002/ar.21232 20734317

[396] Kageyama, Takashi , Masaru Nakamura , Akinori Matsuo , Yasuomi Yamasaki , Yoshinobu Takakura , Mitsuru Hashida , Yoshikatsu Kanai , et al. 2000. “The 4F2hc/LAT1 Complex Transports l‐DOPA Across the Blood–Brain Barrier.” Brain Research 879: 115–121. 10.1016/S0006-8993(00)02758-X 11011012

[397] Soares‐da‐Silva, Patrício , and Maria Paula Serrão . 2004. “High‐ and Low‐Affinity Transport of L‐Leucine and l‐DOPA by the Hetero Amino Acid Exchangers LAT1 and LAT2 in LLC‐PK1 Renal Cells.” American Journal of Physiology‐Renal Physiology 287: F252–F261. 10.1152/ajprenal.00030.2004 15271688

[398] Gori, Sadakatali S. , Ajit G. Thomas , Arindom Pal , Robyn Wiseman , Dana V. Ferraris , Run‐duo Gao , Ying Wu , et al. 2022. “D‐DOPA Is a Potent, Orally Bioavailable, Allosteric Inhibitor of Glutamate Carboxypeptidase II.” Pharmaceutics 14: 2018. 10.3390/pharmaceutics14102018 36297453 PMC9608075

[399] Pinto, Miguel , Tiago Barros Silva , Vilma A. Sardão , Rui Simões , Bárbara Albuquerque , Paulo J. Oliveira , Maria João Valente , et al. 2024. “Cellular and Mitochondrial Toxicity of Tolcapone, Entacapone, and New Nitrocatechol Derivatives.” ACS Pharmacology & Translational Science 7: 1637–1649. 10.1021/acsptsci.4c00124 38751615 PMC11091965

[400] Denayer, Tinneke , Thomas Stöhr , and Maarten Van Roy . 2014. “Animal Models in Translational Medicine: Validation and Prediction.” New Horizons in Translational Medicine 2: 5–11. 10.1016/j.nhtm.2014.08.001

[401] Izadi, Hooman , Hui Wang , Christian Atsriku , Zef Konst , Adam Levinson , Joseph Piccotti , Steven Pirie‐Shepherd , et al. 2023. “Sgr‐2921, a Potent CDC7 Inhibitor, Demonstrates Significant Anti‐Leukemic Responses in Patient‐Derived AML Models Representing Difficult‐to‐Treat Disease.” Blood 142: 2801. 10.1182/blood-2023-181826

[402] Philippidis, Alex . 2025. “Schrödinger Halts Cancer Drug Program After Two Patients Die in Phase I Trial.” GEN Edge 7: 559–562. 10.1089/genedge.7.1.103

[403] Huang, Xinxin , Xu Zhu , Fangyan Fu , Junzhen Song , Jiyu Zeng , Shanshan Huang , and Feng Yue . 2025. “Multi‐Omics Analysis of Plasma and CSF in Spontaneous Diabetic Cynomolgus Monkeys: Unravelling and Validating the Key Molecular Markers That Predict the Preclinical Pathological Formation of Alzheimer's Disease.” Computers in Biology and Medicine 196: 110849. 10.1016/j.compbiomed.2025.110849 40753947

[404] Perez, Jesenia M. , Jolene M. Duda , Joohyun Ryu , Mihir Shetty , Subina Mehta , Pratik D. Jagtap , Andrew C. Nelson , et al. 2025. “Investigating Proteogenomic Divergence in Patient‐Derived Xenograft Models of Ovarian Cancer.” Scientific Reports 15: 813. 10.1038/s41598-024-84874-3 39755759 PMC11700199

[405] Moshref, Maryam , Jerry Hung‐Hao Lo , Andrew McKay , Julien Camperi , Joseph Schroer , Norikiyo Ueno , Shu Wang , et al. 2024. “Assessing a Single‐Cell Multi‐Omic Analytic Platform to Characterize Ex Vivo‐Engineered T‐Cell Therapy Products.” Frontiers in Bioengineering and Biotechnology 12: 1417070. 10.3389/fbioe.2024.1417070 39229457 PMC11368872

[406] Zhao, Ning , Yongjing Liu , Yunzhen Wei , Zichuang Yan , Qiang Zhang , Cheng Wu , Zhiqiang Chang , and Yan Xu . 2016. “Optimization of Cell Lines as Tumour Models by Integrating Multi‐Omics Data.” Briefings in Bioinformatics 18: 515–529. 10.1093/bib/bbw082 27694350

[407] Liu, Caihong , Liangbin Zhou , Cheng Jiang , Rocky S.Tuan , Zhong Alan Li , and Chen‐zhong Li . 2025. “Embracing Human Relevance: the FDA's Historic Move Beyond Animal Testing Mandates.” Cell Organoid 1: 9410013. 10.26599/CO.2025.9410013

[408] Müller, Nico Dario . 2024. “Beyond Anthropocentrism: The Moral and Strategic Philosophy Behind Russell and Burch's 3Rs in Animal Experimentation.” Science and Engineering Ethics 30: 44. 10.1007/s11948-024-00504-1 39261332 PMC11390778

[409] Zhao, Rui , Qiushi Feng , Yangyang Xia , Lingzi Liao , and Shang Xie . 2025. “Iteration of Tumor Organoids in Drug Development: Simplification and Integration.” Pharmaceuticals 18: 1540. 10.3390/ph18101540 41155654 PMC12566727

[410] Ma, Wanting , Zhenglin Dong , Zhu'anzhen Zheng , Long Bai , Xingcai Zhang , and Jiacan Su . 2025. “Multiscale Construction, Evaluation, and Application of Organoids.” Advanced Science 12: e08534. 10.1002/advs.202508534 41214890 PMC12713053

[411] Kim, Chae‐Young , Soon‐Chan Kim , Rumi Shin , Min Jung Kim , Seung‐Yong Jeong , Ji Won Park , and Ja‐Lok Ku . 2026. “Integrative Multi‐Omics of Matched Primary and Liver Metastatic Colorectal Cancer Organoids Identifies Putative Molecular Targets.” Translational Oncology 63: 102592. 10.1016/j.tranon.2025.102592 41192148 PMC12637260

[412] Goodwin, Richard J. A. , Stefan J. Platz , Jorge S. Reis‐Filho , and Simon T. Barry . 2024. “Accelerating Drug Development Using Spatial Multi‐Omics.” Cancer Discovery 14: 620–624. 10.1158/2159-8290.Cd-24-0101 38571424

[413] Li, Xuexin , Lu Pan , Weiyuan Li , Bingyang Liu , Chunjie Xiao , Valerie Chew , Xuan Zhang , et al. 2025. “Deciphering Immune Predictors of Immunotherapy Response: A Multiomics Approach at the Pan‐Cancer Level.” Cell Reports Medicine 6: 101992. 10.1016/j.xcrm.2025.101992 40054456 PMC12047473

[414] Ran, Qiang , Mengjun Huang , Lijuan Wang , Yanyan Li , Wenhui Wu , Xia Liu , Juan Chen , et al. 2025. “Integrated Bioinformatics and Multi‐Omics to Investigate the Mechanism of Rhododendron Molle Flos‐Induced Hepatotoxicity.” Journal of Ethnopharmacology 341: 119308. 10.1016/j.jep.2024.119308 39746411

[415] Al‐Btoosh, Sakha , Ryan F. Donnelly , and Stephen A. Kelly . 2026. “Microbes and Medicines: Interrelationships Between Pharmaceuticals and the Gut Microbiome.” Gut Microbes 18: 2604867. 10.1080/19490976.2025.2604867 41431379 PMC12758310

[416] Takahashi, Yugo , Qingbo S. Wang , Takanori Hasegawa , Ho Namkoong , Fumitaka Inoue , Koichi Fukunaga , Seiya Imoto , Satoru Miyano , and Yukinori Okada , Covid‐Task Force Japan . 2025. “JOB: Japan Omics Browser Provides Integrative Visualization of Multi‐Omics Data.” BMC Genomics 26: 451. 10.1186/s12864-025-11639-1 40335902 PMC12057183

[417] Blatkiewicz, Małgorzata , Marta Szyszka , Szymon Hryhorowicz , Joanna Suszyńska‐Zajczyk , Andrea Porzionato , Adam Plewiński , Ludwik K. Malendowicz , and Marcin Rucinski . 2025. “Identification of Conserved Canonical Marker Genes in Human and Mouse Adrenal Glands Using Visium Spatial Transcriptomics.” Histochemistry and Cell Biology 164: 2. 10.1007/s00418-025-02446-6 41442035 PMC12738614

[418] Wu, Jichun , He Nie , Hui Wang , Faping Li , Yanqiu Ma , Xiaojing Yang , Dong Wang , et al. 2025. “Integrative Multi‐Omics Identifies S100A8/IGFBP5/CTSK/S100P as Dual Diagnostic Biomarkers and Therapeutic Targets in Crohn's Disease: From Computational Discovery to Preclinical Validation.” International Immunopharmacology 165: 115500. 10.1016/j.intimp.2025.115500 40929958

[419] Bates, Susan E . 2020. “Epigenetic Therapies for Cancer.” New England Journal of Medicine 383: 650–663. 10.1056/NEJMra1805035 32786190

[420] Rayad, Noha , Ekram A. Chowdhury , and Guy M. L. Meno‐Tetang . 2025. “The Impact of QSP Modeling on the Design and Optimization of Gene Therapy Approaches.” CPT: Pharmacometrics & Systems Pharmacology 14: 1760–1764. 10.1002/psp4.70131 41220317 PMC12625082

[421] Caracciolo, Giulio . 2015. “Liposome–Protein Corona in a Physiological Environment: Challenges and Opportunities for Targeted Delivery of Nanomedicines.” Nanomedicine: Nanotechnology, Biology and Medicine 11: 543–557. 10.1016/j.nano.2014.11.003 25555353

[422] Zhang, Zhongheng , Lin Chen , Hongjie Shen , Jing Wang , Jie Yang , Suibi Yang , Weimin Zhang , et al. 2025. “Deriving Consensus Sepsis Clusters via Goal‐Directed Subgroup Identification in Multi‐Omics Study.” Nature Communications 16: 10328. 10.1038/s41467-025-65271-4 PMC12644673 41285725

[423] Freidlin, Boris , and Edward L. Korn . 2014. “Biomarker Enrichment Strategies: Matching Trial Design to Biomarker Credentials.” Nature Reviews Clinical Oncology 11: 81–90. 10.1038/nrclinonc.2013.218 24281059

[424] Hamamoto, Ryuji , Ken Takasawa , Hidenori Machino , Kazuma Kobayashi , Satoshi Takahashi , Amina Bolatkan , Norio Shinkai , et al. 2022. “Application of Non‐Negative Matrix Factorization in Oncology: One Approach for Establishing Precision Medicine.” Briefings in Bioinformatics 23: bbac246. 10.1093/bib/bbac246 35788277 PMC9294421

[425] Yang, Mu , Stuart Matan‐Lithwick , Yanling Wang , Philip L. De Jager , David A. Bennett , and Daniel Felsky . 2023. “Multi‐Omic Integration via Similarity Network Fusion to Detect Molecular Subtypes of Ageing.” Brain Communications 5: fcad110. 10.1093/braincomms/fcad110 37082508 PMC10110975

[426] Hänzelmann, Sonja , Robert Castelo , and Justin Guinney . 2013. “GSVA: Gene Set Variation Analysis for Microarray and RNA‐Seq Data.” BMC Bioinformatics 14: 7. 10.1186/1471-2105-14-7 23323831 PMC3618321

[427] Yang, Hai , Rui Chen , Dongdong Li , and Zhe Wang . 2021. “Subtype‐GAN: A Deep Learning Approach for Integrative Cancer Subtyping of Multi‐Omics Data.” Bioinformatics 37: 2231–2237. 10.1093/bioinformatics/btab109 33599254

[428] Zhang, Ge , Zeyu Wang , Zhuang Tong , Zhen Qin , Chang Su , Demin Li , Shuai Xu , et al. 2024. “AI Hybrid Survival Assessment for Advanced Heart Failure Patients With Renal Dysfunction.” Nature Communications 15: 6756. 10.1038/s41467-024-50415-9 PMC11310499 39117613

[429] Administration, Food and Drug . March 13, 2026. *Clinical Trial Endpoints for the Approval of Cancer Drugs and Biologics*, FDA. https://www.fda.gov/regulatory-information/search-fda-guidance-documents/clinical-trial-endpoints-approval-cancer-drugs-and-biologics

[430] Antoine, Alison , Katia Desroziers , Julien Dupin , David Pérol , and Rémy Choquet . 2025. “Enhancing Confidence in Complex Health Technology Assessments by Using Real‐World Evidence: Highlighting Existing Strategies for Effective Drug Evaluation.” BMC Medical Research Methodology 25: 248. 10.1186/s12874-025-02683-2 41184774 PMC12581489

[431] Kummar, Shivaani , Larry Rubinstein , Robert Kinders , Ralph E. Parchment , Martin E. Gutierrez , Anthony J. Murgo , Jay Ji , et al. 2008. “Phase 0 Clinical Trials: Conceptions and Misconceptions.” Cancer Journal 14: 133–137. 10.1097/PPO.0b013e318172d6f3 18536551 PMC7185299

[432] Perez de Souza, Leonardo , Saleh Alseekh , Federico Scossa , and Alisdair R. Fernie . 2021. “Ultra‐High‐Performance Liquid Chromatography High‐Resolution Mass Spectrometry Variants for Metabolomics Research.” Nature Methods 18: 733–746. 10.1038/s41592-021-01116-4 33972782

[433] Wen, Dingsheng , Boyu Chen , Mingtong Deng , Shaoyu Wu , and Shuilin Xie . 2025. “Plasma Protein Binding, Biostability, Metabolite Profiling, and CYP450 Phenotype of TPB15 Across Different Species: A Novel Smoothened Inhibitor for TNBC Therapy.” Pharmaceutics 17: 423. 10.3390/pharmaceutics17040423 40284418 PMC12030497

[434] Ji, Yuan , and Sue‐Jane Wang . 2013. “Modified Toxicity Probability Interval Design: A Safer and More Reliable Method Than the 3 + 3 Design for Practical Phase I Trials.” Journal of Clinical Oncology 31: 1785–1791. 10.1200/jco.2012.45.7903 23569307 PMC3641699

[435] Guo, Xing Gang , Zhi Heng Wang , Wei Dong , Xu Dong He , FuChen Liu , and Hui Liu . 2018. “Specific CYP450 Genotypes in the Chinese Population Affect Sorafenib Toxicity in HBV/HCV‐associated Hepatocellular Carcinoma Patients.” Biomedical and Environmental Sciences 31: 586–595. 10.3967/bes2018.080 30231963

[436] Zhang, Miao , Xiaolei Zhou , Zhenzhen Li , TianYu Zhao , Yu Miao , Sen Liu , Qiaoqiao Han , et al. 2025. “Decitabine Regulates the Resistance of HCC to Sorafenib Through Demethylation.” Clinical Epigenetics 17: 120. 10.1186/s13148-025-01925-w 40624511 PMC12235873

[437] Doroshow, James H. , and Shivaani Kummar . 2009. “Role of Phase 0 Trials in Drug Development.” Future Medicinal Chemistry 1: 1375–1380. 10.4155/fmc.09.117 21426052

[438] Amghar, Mariam , Tobias Rausch , Hilal Özgür , Mareike Roscher , Ulrike Bauder‐Wüst , Frank Bruchertseifer , Alfred Morgenstern , et al. 2026. “Exploratory Case Series of Circulating Tumor DNA Dynamics During Tandem Therapy in Metastatic Castration‐Resistant Prostate Cancer.” EJNMMI Research 16: 43. 10.1186/s13550-026-01388-x 41670929 PMC13000081

[439] Singh, Sonia , Adnan A. Jaigirdar , Flora Mulkey , Joyce Cheng , Salaheldin S. Hamed , Yangbing Li , Jiang Liu , et al. 2021. “FDA Approval Summary: Lurbinectedin for the Treatment of Metastatic Small Cell Lung Cancer.” Clinical Cancer Research 27: 2378–2382. 10.1158/1078-0432.Ccr-20-3901 33288660 PMC9532454

[440] Liu, Ian T. T. , Aaron S. Kesselheim , and Edward R. Scheffer Cliff . 2024. “Clinical Benefit and Regulatory Outcomes of Cancer Drugs Receiving Accelerated Approval.” JAMA 331: 1471–1479. 10.1001/jama.2024.2396 38583175 PMC11000139

[441] Yamazaki, Makoto , Manami Miyake , Hiroko Sato , Naoya Masutomi , Naohisa Tsutsui , Klaus‐Peter Adam , Danny C. Alexander , et al. 2013. “Perturbation of Bile Acid Homeostasis Is an Early Pathogenesis Event of Drug Induced Liver Injury in Rats.” Toxicology and Applied Pharmacology 268: 79–89. 10.1016/j.taap.2013.01.018 23360887

[442] Ellinger‐Ziegelbauer, Heidrun , Melanie Adler , Alexander Amberg , Arnd Brandenburg , John J. Callanan , Susan Connor , Michael Fountoulakis , et al. 2011. “The Enhanced Value of Combining Conventional and ‘Omics’ Analyses in Early Assessment of Drug‐Induced Hepatobiliary Injury.” Toxicology and Applied Pharmacology 252: 97–111. 10.1016/j.taap.2010.09.022 20888850

[443] Li, Huishan , Yan Wang , Jianfeng Zhu , Hehui Chen , Tianshu Liu , and Luoyan Ai . 2025. “Mechanistic Insights Into PD‐1 Inhibitor‐Associated Immune Thrombocytopenia: A Case Report Integrating Longitudinal Transcriptomic Profiling.” Journal for ImmunoTherapy of Cancer 13: e013108. 10.1136/jitc-2025-013108 41475844 PMC12766837

[444] Corrigan‐Curay, Jacqueline , Leonard Sacks , and Janet Woodcock . 2018. “Real‐World Evidence and Real‐World Data for Evaluating Drug Safety and Effectiveness.” JAMA 320: 867–868. 10.1001/jama.2018.10136 30105359

[445] Nassani, Rayan , Yahya Bokhari , and Bahauddeen M. Alrfaei . 2023. “Molecular Signature to Predict Quality of Life and Survival With Glioblastoma Using Multiview Omics Model.” PLOS One 18: e0287448. 10.1371/journal.pone.0287448 37972206 PMC10653472

[446] Luo, Qiang , Yuxiao Chen , Dawei Liu , Xinlin Wu , Jun Yang , Haiguo Yu , Hongmei Song , et al. 2025. “Integrative Pharmacovigilance and AI‐Based Framework Uncovers Potential Drug Triggers in Juvenile Idiopathic Arthritis.” Frontiers in Immunology 16: 1653003. 10.3389/fimmu.2025.1653003 41256847 PMC12620426

[447] Golder, Su , Dongfang Xu , Karen O'Connor , Yunwen Wang , Mahak Batra , and Graciela Gonzalez Hernandez . 2025. “Leveraging Natural Language Processing and Machine Learning Methods for Adverse Drug Event Detection in Electronic Health/Medical Records: A Scoping Review.” Drug Safety 48: 321–337. 10.1007/s40264-024-01505-6 39786481 PMC11903561

[448] Iida, Midori , Yurika Kuniki , Kenta Yagi , Mitsuhiro Goda , Satoko Namba , Jun‐ichi Takeshita , Ryusuke Sawada , et al. 2024. “A Network‐Based Trans‐Omics Approach for Predicting Synergistic Drug Combinations.” Communications Medicine 4: 154. 10.1038/s43856-024-00571-2 39075184 PMC11286857

[449] Colibăşanu, Daiana , Vlad Groza , Maria Antonietta Occhiuzzi , Fedora Grande , Mihai Udrescu , and Lucreția Udrescu . 2025. “Drug Repositioning Pipeline Integrating Community Analysis in Drug‐Drug Similarity Networks and Automated ATC Community Labeling to Foster Molecular Docking Analysis.” Frontiers in Bioinformatics 5: 1666716. 10.3389/fbinf.2025.1666716 41210919 PMC12589059

[450] Hakariya, Hayase , Akihiko Ozaki , and Tetsuya Tanimoto . 2025. “US FDA–Accelerated Approvals and Subsequent Withdrawals: Influence on Japanese Clinical Oncology Practice Guidelines.” Investigational New Drugs 43: 311–317. 10.1007/s10637-025-01524-9 40178688 PMC12048449

[451] Selby, Chris , Lisa R. Yacko , and Ashley E. Glode . 2019. “Gemtuzumab Ozogamicin: Back again.” Journal of the Advanced Practitioner in Oncology 10: 68–82. 10.6004/jadpro.2019.10.1.6 31308990 PMC6605703

[452] Dagogo‐Jack, Ibiayi , and Alice T. Shaw . 2018. “Tumour Heterogeneity and Resistance to Cancer Therapies.” Nature Reviews Clinical Oncology 15: 81–94. 10.1038/nrclinonc.2017.166 29115304

[453] Newell, Felicity , Peter A. Johansson , James S. Wilmott , Katia Nones , Vanessa Lakis , Antonia L. Pritchard , Serigne N. Lo , et al. 2022. “Comparative Genomics Provides Etiologic and Biological Insight Into Melanoma Subtypes.” Cancer Discovery 12: 2856–2879. 10.1158/2159-8290.CD-22-0603 36098958 PMC9716259

[454] Wang, Rui , Jingyun Li , Xin Zhou , Yunuo Mao , Wendong Wang , Shuai Gao , Wei Wang , et al. 2022. “Single‐Cell Genomic and Transcriptomic Landscapes of Primary and Metastatic Colorectal Cancer Tumors.” Genome Medicine 14: 93. 10.1186/s13073-022-01093-z 35974387 PMC9380328

[455] Gui, Chengcheng , Henry S. Walch , Kirin D. Mueller , Lillian A. Boe , Anna Skakodub , Emily Miao , Ishaani Khatri , et al. 2025. “Genomics Correlates of Brain Metastasis and Progression in Colorectal Cancer.” Neuro‐Oncology 27: 3121–3131. 10.1093/neuonc/noaf198 40891323 PMC12916729

[456] Ho, Shamaine Wei Ting , Taotao Sheng , Manjie Xing , Wen Fong Ooi , Chang Xu , Raghav Sundar , Kie Kyon Huang , et al. 2023. “Regulatory Enhancer Profiling of Mesenchymal‐Type Gastric Cancer Reveals Subtype‐Specific Epigenomic Landscapes and Targetable Vulnerabilities.” Gut 72: 226–241. 10.1136/gutjnl-2021-326483 35817555

[457] Bian, Shuhui , Yicheng Wang , Yuan Zhou , Wendong Wang , Limei Guo , Lu Wen , Wei Fu , Xin Zhou , and Fuchou Tang . 2023. “Integrative Single‐Cell Multiomics Analyses Dissect Molecular Signatures of Intratumoral Heterogeneities and Differentiation States of Human Gastric Cancer.” National Science Review 10: nwad094. 10.1093/nsr/nwad094 37347037 PMC10281500

[458] Hohmann, Lennart , Deborah F. Nacer , Mattias Aine , Yasin Memari , Daniella Black , Ramsay Bowden , Helen R. Davies , et al. 2025. “Molecular Profiling of the Basal‐Like Intrinsic Molecular Subtype in Primary ER‐positive HER2‐negative Breast Cancer.” Genome Medicine 17: 146. 10.1186/s13073-025-01576-9 41327380 PMC12667147

[459] Miyakoshi, Jun , Kouya Shiraishi , Akifumi Mochizuki , Akiko Tateishi , Yukiko Shimoda‐Igawa , Masahiro Torasawa , Hanako Ono , et al. 2025. “Chromosome 15q15 Deletion Drives Brain Metastasis in NSCLC.” Journal of Thoracic Oncology 21: 103511. 10.1016/j.jtho.2025.11.001 41207477

[460] Vis, Daniel J. , Sander A. L. Palit , Marie Corradi , Edwin Cuppen , Niven Mehra , Martijn P. Lolkema , Lodewyk F. A. Wessels , et al. 2025. “Whole Genome Sequencing of 378 Prostate Cancer Metastases Reveals Tissue Selectivity for Mismatch Deficiency With Potential Therapeutic Implications.” Genome Medicine 17: 24. 10.1186/s13073-025-01445-5 40114169 PMC11927350

[461] Fu, Yifan , Jinxin Tao , Tao Liu , Yueze Liu , Jiangdong Qiu , Dan Su , Ruobing Wang , et al. 2024. “Unbiasedly Decoding the Tumor Microenvironment With Single‐Cell Multiomics Analysis in Pancreatic Cancer.” Molecular Cancer 23: 140. 10.1186/s12943-024-02050-7 38982491 PMC11232163

[462] Ma, Yao , Lijian Wang , Ziyue Zhang , Yifei Zhao , Zimin Zhao , Renjie Luo , Junwei Wang , et al. 2025. “Comprehensive Single‐Cell Transcriptome Landscape of Metastatic Colorectal Cancer Identifies Budding‐Potential Cells and Their Interactions With Cancer‐Associated Fibroblasts.” npj Precision Oncology 9: 408. 10.1038/s41698-025-01187-y 41298776 PMC12749751

[463] Parsons, Adrienne , Esther Sauras Colón , Meghana Manjunath , Hanyun Zhang , Julia Chen , Milos Spasic , Beyza Koca , et al. 2025. “Cell Populations in Human Breast Cancers Are Molecularly and Biologically Distinct With Age.” Nature Aging 5: 2546–2563. 10.1038/s43587-025-00984-1 41188599 PMC12705435

[464] Guo, De‐Zhen , Xin Zhang , Sen‐Quan Zhang , Shi‐Yu Zhang , Xiang‐Yu Zhang , Jia‐Yan Yan , San‐Yuan Dong , et al. 2024. “Single‐Cell Tumor Heterogeneity Landscape of Hepatocellular Carcinoma: Unraveling the Pro‐Metastatic Subtype and Its Interaction Loop With Fibroblasts.” Molecular Cancer 23: 157. 10.1186/s12943-024-02062-3 39095854 PMC11295380

[465] Marteau, Valentin , Niloofar Nemati , Kristina Handler , Deeksha Raju , Alexander Kirchmair , Dietmar Rieder , Erika Kvalem Soto , et al. 2026. “Single‐Cell Integration and Multi‐Modal Profiling Reveals Phenotypes and Spatial Organization of Neutrophils in Colorectal Cancer.” Cancer Cell 44: 146–165.e14. 10.1016/j.ccell.2025.12.003 41529665

[466] Cheng, Yixin , Guanyin Huang , Xuefei Liu , Chun Wu , Jingru Lian , Jing Cao , Lianhui Duan , et al. 2026. “Fusion of Tumor Cells With Lipid‐Associated Macrophages Drives Metastatic Progression of Breast Cancer.” Cancer Research 86: 661–678. 10.1158/0008-5472.CAN-25-0261 41342370

[467] Chen, Min‐Min , Qinhang Gao , Huiheng Ning , Kang Chen , Yang Gao , Min Yu , Chao‐Qun Liu , et al. 2025. “Integrated Single‐Cell and Spatial Transcriptomics Uncover Distinct Cellular Subtypes Involved in Neural Invasion in Pancreatic Cancer.” Cancer Cell 43: 1656–1676.e10. 10.1016/j.ccell.2025.06.020 40680743

[468] Liu, Zedao , Zhenlin Yang , Junqi Wu , Wenjie Zhang , Yuxuan Sun , Chao Zhang , Guangyu Bai , et al. 2025. “A Single‐Cell Atlas Reveals Immune Heterogeneity in Anti‐PD‐1‐Treated Non‐Small Cell Lung Cancer.” Cell 188: 3081–3096.e19. 10.1016/j.cell.2025.03.018 40147443

[469] Zhang, Yao , Yanhua Du , Jiaxin Wang , Danshu Wang , Judong Li , Jing Zhang , Yizhou Zhao , et al. 2026. “Single‐Cell Analysis of Chemotherapy‐Induced Remodeling Reveals CD276‐Driven Basal‐Like Chemoresistance in Pancreatic Cancer.” Gastroenterology 170: 769–786. 10.1053/j.gastro.2025.09.043 41701125

[470] Li, Yuanfang , Yongqiang Zheng , Jiaqian Huang , Run‐Cong Nie , Qi‐Nian Wu , Zhijun Zuo , Shuqiang Yuan , et al. 2025. “CAF‐Macrophage Crosstalk in Tumour Microenvironments Governs the Response to Immune Checkpoint Blockade in Gastric Cancer Peritoneal Metastases.” Gut 74: 350–363. 10.1136/gutjnl-2024-333617 39537239 PMC11874311

[471] Li, Juan , Aijing Liu , Weili Duan , Yanru Li , Xue Kong , Tiantian Wang , Dun Niu , et al. 2025. “Ubiquitin‐Specific Protease 48 Drives Malignant Progression of Colorectal Cancer by Suppressing Autophagy Through Stabilizing Sequestosome 1.” Cell Death & Differentiation 33: 1004–1019. 10.1038/s41418-025-01617-1 41299086 PMC13156323

[472] Chowdhury, Nayela N. , Dana K. Mitchell , Kadri Kangro , Kierra Eldridge , Sara Abrahams , Francesca Ferraresso , Lih J. Juang , et al. 2025. “Depletion of Fibrinogen Suppresses Growth of Primary Tumors and Metastasis of Pancreatic Ductal Adenocarcinoma.” Gastroenterology 170: 753–768. 10.1053/j.gastro.2025.09.024 41432649 PMC12731649

[473] Cai, Tingting , Tao Feng , Wei Zhang , Qintao Ge , Liangju Peng , Yue Wang , Jindong Xie , et al. 2025. “Neutrophil Extracellular Traps‐STC1 Positive Feedback Loop Promotes Immune Evasion and Metastasis in Bladder Cancer.” Journal for ImmunoTherapy of Cancer 13: e012736. 10.1136/jitc-2025-012736 41314976 PMC12666199

[474] Yang, Jie , Chenggong Liao , Xiaohua Liang , Yuan Ke , Ying Sun , Minmin Huang , Meirui Qian , et al. 2025. “CD147 Promotes NSCLC Metastasis by Inducing Secretory Autophagy‐Dependent Exosome Secretion via TRIM56‐mediated Ubiquitination and Degradation of GCN2.” Cell Death & Differentiation 33: 1152–1174. 10.1038/s41418-025-01636-y 41413248 PMC13247162

[475] Wang, Qilong , Yuening Jiang , Meiyu Bi , Gang Wang , Yinan Xiao , Hailian Zhao , Yuhan Guo , et al. 2025. “In Vivo Proteomic Labeling Reveals Diverse Proteomes for Therapeutic Targets.” Molecular Cell 85: 4651–4666.e9. 10.1016/j.molcel.2025.11.008 41330386

[476] Zhao, Jiangnan , Mo Shen , Xia Xu , Shunxian Ji , Shumin Xu , Lei Xu , Ying Yang , et al. 2025. “Proteomics and Single Cell Profiling Identify Keratin Driven Preexisting Immunity Influences Lung Squamous Carcinoma Neoadjuvant Therapy.” Cancer Letters 633: 218000. 10.1016/j.canlet.2025.218000 40850567

[477] Zhang, Hangyu , Libing Hong , Zhen Dong , Shan Xin , Bo Lin , Jinlin Cheng , Weihong Tian , et al. 2025. “Spatially Resolved C1QC(+) macrophage‐CD4(+) T Cell Niche in Colorectal Cancer Microenvironment: Implications for Immunotherapy Response.” Cell Discovery 11: 60. 10.1038/s41421-025-00811-2 40593467 PMC12219098

[478] Cheng, Yifei , Xiaofang Chen , Li Feng , Zhicheng Yang , Liyun Xiao , Bin Xiang , Xiaodong Wang , et al. 2025. “Stromal Architecture and Fibroblast Subpopulations With Opposing Effects on Outcomes in Hepatocellular Carcinoma.” Cell Discovery 11: 1. 10.1038/s41421-024-00747-z 39870619 PMC11772884

[479] Chen, Bo , Karrie M. Kiang , Fangkun Liu , Chuntao Li , Xizhe Li , Chen Weiwei , Xin Fu , et al. 2026. “Neutrophil Extracellular Trap Reprograms Cancer Metabolism to Form a Metastatic Niche Promoting Non‐Small Cell Lung Cancer Brain Metastasis.” Advanced Science 13: e08478. 10.1002/advs.202508478 41250997 PMC12850224

[480] Li, Yini , Tongtong Tian , Qian Yu , Huiqin Jiang , Te Liu , Hao Wang , Ran Huo , et al. 2026. “Elaidic Acid Suppresses Hepatocellular Carcinoma Growth Through Modulating the Production of Intestinal Ligilactobacillus Murinus‐Derived Spermidine.” International Journal of Biological Sciences 22: 1542–1559. 10.7150/ijbs.122392 41608620 PMC12839069

[481] Wu, Di , Chunbin Zhu , Haoqi Pan , He Xu , Jin Xu , Yang Liu , Sikai Wang , et al. 2026. “Integrated Screens Reveal That Guanine Nucleotide Depletion, Which Is Irreversible via Targeting IMPDH2, Inhibits Pancreatic Cancer and Potentiates KRAS Inhibition.” Gut: gutjnl‐2025‐336235. 10.1136/gutjnl-2025-336235 PMC13691714 41494804

[482] Yuan, Qihang , Yushan Sun , Yue Zhang , Chen Chen , Chenye Bu , Xiaolong Hua , Lejia Sun , et al. 2026. “Intratumoral P. Copri Reprograms MARCO+ Tumor‐Associated Macrophages by Depleting Glycerophosphocholine to Drive Colorectal Cancer Progression.” Cancer Research 86: 2414–2428. 10.1158/0008-5472.CAN-25-3400 41591361

[483] Chianese, Ugo , Chiara Papulino , Gerardo Saggese , Ahmad Ali , Marianna Ciotola , Enza Lonardo , Mirko Cortese , et al. 2026. “Glycolytic Heterogeneity Drives Metabolic‐Targeted Therapy in Pancreatic Ductal Adenocarcinoma.” Signal Transduction and Targeted Therapy 11: 25. 10.1038/s41392-025-02546-8 41554705 PMC12816621

[484] Gu, Jian , Yu Li , Qiuyang Chen , Ziyan Song , Qufei Qian , Yuan Liang , Tianning Huang , et al. 2026. “Tumor‐Produced Ammonia Is Metabolized by Regulatory T Cells to Further Impede Anti‐Tumor Immunity.” Cell 189: 418–434. 10.1016/j.cell.2025.11.034 41448182

[485] Liang, Lei , Cheng Kong , Jinming Li , Guang Liu , Jinwang Wei , Guan Wang , Qinying Wang , et al. 2024. “Distinct Microbes, Metabolites, and the Host Genome Define the Multi‐Omics Profiles in Right‐Sided and Left‐Sided Colon Cancer.” Microbiome 12: 274. 10.1186/s40168-024-01987-7 39731152 PMC11681701

[486] Jia, Yan , Li Yuan , Weijia Wen , Linna Chen , Xueyuan Zhao , Qiong Wu , Yan Liao , et al. 2025. “Circulating Proteins and Metabolites Panel for Noninvasive Preoperative Diagnosis of Epithelial Ovarian Cancer.” BMC Medicine 23: 492. 10.1186/s12916-025-04341-2 40846961 PMC12374407

[487] Heidegger, Isabel , Maria Frantzi , Stefan Salcher , Piotr Tymoszuk , Agnieszka Martowicz , Enrique Gomez‐Gomez , Ana Blanca , et al. 2025. “Prediction of Clinically Significant Prostate Cancer by a Specific Collagen‐Related Transcriptome, Proteome, and Urinome Signature.” European Urology Oncology 8: 652–662. 10.1016/j.euo.2024.05.014 38851995

[488] Tao, Yuhuan , Shaozhen Xing , Shuai Zuo , Pengfei Bao , Yunfan Jin , Yu Li , Mingyang Li , et al. 2023. “Cell‐Free Multi‐Omics Analysis Reveals Potential Biomarkers in Gastrointestinal Cancer Patients’ Blood.” Cell Reports Medicine 4: 101281. 10.1016/j.xcrm.2023.101281 37992683 PMC10694666

[489] Zhang, Hang , Fan Yang , Ying Xu , Shen Zhao , Yi‐Zhou Jiang , Zhi‐Ming Shao , and Yi Xiao . 2025. “Multimodal Integration Using a Machine Learning Approach Facilitates Risk Stratification in HR+/HER2‐ Breast Cancer.” Cell Reports Medicine 6: 101924. 10.1016/j.xcrm.2024.101924 39848244 PMC11866502

[490] Chang, Ya‐Hsuan , Tzu‐Chan Hong , Kuen‐Tyng Lin , Yi‐Jing Hsiao , Hsiang‐En Hsu , Juanilita T. Waniwan , Rodrigo Espinoza Silva , et al. 2026. “Integrative Proteogenomics Maps Multifactorial Aetiology, Progression and Therapeutic Vulnerabilities in Gastric Cancer.” Gut 75: 886–904. 10.1136/gutjnl-2025-337247 41617485

[491] Yu, Yanxi , Yan You , Yuxin Duan , Meiqing Kang , Baoyong Zhou , Jian Yang , Kunli Yin , et al. 2026. “Multi‐Omics Approaches for Identifying the PANoptosis Signature and Prognostic Model via a Multimachine‐Learning Computational Framework for Intrahepatic Cholangiocarcinoma.” Hepatology 83: 466–483. 10.1097/HEP.0000000000001352 40233411 PMC12904247

[492] Chauhan, Pradeep S. , Irfan Alahi , Savar Sinha , Elisa M. Ledet , Ryan Mueller , Jessica Linford , Alexander L. Shiang , et al. 2025. “Genomic and Epigenomic Analysis of Plasma Cell‐Free DNA Identifies Stemness Features Associated With Worse Survival in Lethal Prostate Cancer.” Clinical Cancer Research 31: 151–163. 10.1158/1078-0432.CCR-24-1658 39177583 PMC11743868

[493] Woo, Seonjeong , Beodeul Kang , Sung Hwan Lee , Jung Sun Kim , Haeyoun Kang , Seok Jeong Yang , Chansik An , et al. 2025. “Biology‐Driven Stratification of Advanced Biliary Tract Cancer Treated With Nab‐Paclitaxel Plus Gemcitabine‐Cisplatin: A Prospective Observational Cohort Study.” Hepatology. 10.1097/HEP.0000000000001650 PMC13480321 41416898

[494] Fu, Zile , Yuanli Song , Fen Liu , Lv Chen , Shangli Cai , Peng Cui , Guoqiang Wang , et al. 2026. “Integrative Proteogenomic Analysis Provides Molecular Insights and Clinical Significance in Gallbladder Cancer.” Cancer Cell 44: 405–423.e13. 10.1016/j.ccell.2025.12.014 41512870

[495] Zhang, Pei , Dan Liu , Tonghui Yu , Yanlin Zhang , Lu Zhong , Xiao Ouyang , Qin Xia , and Lei Dong . 2025. “Integrated Pathway Analysis Identifies Prognostically Relevant Subtypes of Glioblastoma Characterized by Abnormalities in Multi‐Omics.” Clinical and Translational Medicine 15: e70517. 10.1002/ctm2.70517 41292185 PMC12647366

[496] Li, Binghua , Yunzheng Li , Huajun Zhou , Yanchao Xu , Yajuan Cao , Chunxiao Cheng , Jin Peng , et al. 2024. “Multiomics Identifies Metabolic Subtypes Based on Fatty Acid Degradation Allocating Personalized Treatment in Hepatocellular Carcinoma.” Hepatology 79: 289–306. 10.1097/HEP.0000000000000553 37540187 PMC10789383

[497] Xu, Shouping , Keda Yu , Lei Liu , Qin Wang , Xiaohui Wu , Yihai Chen , Guozheng Li , et al. 2026. “Proteogenomic Characterization Reveals Subtype‐Specific Therapeutic Potential for HER2‐Low Breast Cancer.” Advanced Science 13: e13086. 10.1002/advs.202513086 41454718 PMC12948274

[498] Liu, Qian , Jing Zhang , Chenchen Guo , Mengcheng Wang , Chenfei Wang , Yilv Yan , Liangdong Sun , et al. 2024. “Proteogenomic Characterization of Small Cell Lung Cancer Identifies Biological Insights and Subtype‐Specific Therapeutic Strategies.” Cell 187: 184–203. 10.1016/j.cell.2023.12.004 38181741

[499] Xiao, Yuling , Hang Zhang , Yi Xiao , Ying Wang , Jing Zhang , Qi Hua , Pengchen Hu , et al. 2025. “High‐Precision Immune‐Related Plasma Proteomics Profiling Predicts Response to Immunotherapy in Patients With Triple‐Negative Breast Cancer.” Cancer Biology & Medicine 22: 854. 10.20892/j.issn.2095-3941.2025.0038 40613791 PMC12302270

[500] Zou, Xin , Yujun Liu , Miaochen Wang , Jiawei Zou , Yi Shi , Xianbin Su , Juan Xu , et al. 2023. “scCURE Identifies Cell Types Responding to Immunotherapy and Enables Outcome Prediction.” Cell Reports Methods 3: 100643. 10.1016/j.crmeth.2023.100643 37989083 PMC10694528

[501] Cao, Ailing , Yaobin Lin , Shaoxing Guan , Youhao Chen , Wenyu Zhai , Yuheng Zhou , Shoucheng Feng , et al. 2026. “Baseline Multi‐Omics Signatures Could Predict Therapeutic Response to Neoadjuvant anti‐PD‐1 Immunochemotherapy in Non‐Small‐Cell Lung Cancer.” Clinical and Translational Medicine 16: e70579. 10.1002/ctm2.70579 41499358 PMC12778419

[502] Wang, Shanshan , Zhengguang Guo , Boyu Sun , Kai Liu , Jiashuo Chao , Ziyu Xun , Yunchao Wang , et al. 2025. “Dynamic Urinary Proteomics Integrates Single‐Cell and Spatial Transcriptomics to Reveal Tumour Microenvironment and Predict Immunotherapy Response in Biliary Tract Cancer.” Gut 75: 1411–1424. 10.1136/gutjnl-2025-335513 PMC13311962 41151791

[503] Zheng, Liang , Haoming Xu , Shuyuan Wang , Meili Ma , Fang Hu , Lei Cheng , Jun Lu , et al. 2026. “Multi‐Omics Dynamic Profiling Reveals Predictive Biomarkers for First‐Line Immunochemotherapy in Extensive‐Stage Small‐Cell Lung Cancer.” Journal of Translational Medicine 24: 353. 10.1186/s12967-026-07778-y 41654892 PMC12977650

[504] Zhou, Yaoyao , Ziyun Liu , Cheng Gong , Jie Zhang , Jing Zhao , Xia Zhang , Xiangyu Liu , et al. 2024. “Targeting Treatment Resistance: Unveiling the Potential of RNA Methylation Regulators and TG‐101,209 in Pan‐Cancer Neoadjuvant Therapy.” Journal of Experimental & Clinical Cancer Research 43: 232. 10.1186/s13046-024-03111-x 39160604 PMC11331809

[505] Zhao, Yuheng , Chunxian Ou , Huimin Huang , Yujue Wang , Jiaxi Shen , Yaoyi Li , Qingmei He , et al. 2026. “IDH1 Reprograms Nucleotide Metabolism by Inducing Chromatin Remodeling and DHODH Transcription to Drive Chemoresistance in Nasopharyngeal Carcinoma.” Cancer Research 86: 2004–2023. 10.1158/0008-5472.CAN-25-2313 41570327

[506] Efthymiou, Georgios , Eugénie Lohmann , Claudio Montenegro , Paraskevi Kousteridou , Pierre Bertrand , Julia Pereira , Heather S. Carr , et al. 2025. “The Extracellular Matrix Drives Guanylate Production and Protects Pancreatic Cancer Cells From Oxaliplatin‐Induced DNA Damage.” Science Advances 11: eadu2276. 10.1126/sciadv.adu2276 41385622 PMC12700215

[507] Holt, Matthew V. , Yongchao Dou , Meggie N. Young , Alexander B. Saltzman , Meenakshi Anurag , Jonathan T. Lei , Antrix Jain , et al. 2025. “Proteogenomic Characterization Unveils Biomarkers Associated With Chemoresistance in Muscle‐Invasive Bladder Cancer.” Cell Reports Medicine 6: 102255. 10.1016/j.xcrm.2025.102255 40749681 PMC12432383

[508] Roudko, Vladimir , Diane Marie Del Valle , Emir Radkevich , Geoffrey Kelly , Xie Hui , Manishkumar Patel , Edgar Gonzalez‐Kozlova , et al. 2025. “Immunological Biomarkers of Response and Resistance to Treatment With Cabozantinib and Nivolumab in Recurrent Endometrial Cancer.” Journal for ImmunoTherapy of Cancer 13: e010541. 10.1136/jitc-2024-010541 40010771 PMC11865721

[509] Cho, Judy H. , and Marc Feldman . 2015. “Heterogeneity of Autoimmune Diseases: Pathophysiologic Insights From Genetics and Implications for New Therapies.” Nature Medicine 21: 730–738. 10.1038/nm.3897 PMC5716342 26121193

[510] Bogdanos, Dimitrios P. , Daniel S. Smyk , Eirini I. Rigopoulou , Maria G. Mytilinaiou , Michael A. Heneghan , Carlo Selmi , and M. Eric Gershwin . 2012. “Twin Studies in Autoimmune Disease: Genetics, Gender and Environment.” Journal of Autoimmunity 38: J156–J169. 10.1016/j.jaut.2011.11.003 22177232

[511] Baysoy, Alev , Zhiliang Bai , Rahul Satija , and Rong Fan . 2023. “The Technological Landscape and Applications of Single‐Cell Multi‐Omics.” Nature Reviews Molecular Cell Biology 24: 695–713. 10.1038/s41580-023-00615-w 37280296 PMC10242609

[512] Chiou, Joshua , Ryan J. Geusz , Mei‐Lin Okino , Jee Yun Han , Michael Miller , Rebecca Melton , Elisha Beebe , et al. 2021. “Interpreting Type 1 Diabetes Risk With Genetics and Single‐Cell Epigenomics.” Nature 594: 398–402. 10.1038/s41586-021-03552-w 34012112 PMC10560508

[513] Demela, Pietro , Nicola Pirastu , and Blagoje Soskic . 2023. “Cross‐Disorder Genetic Analysis of Immune Diseases Reveals Distinct Gene Associations That Converge on Common Pathways.” Nature Communications 14: 2743. 10.1038/s41467-023-38389-6 PMC10182075 37173304

[514] Yu, Lu , Zhenhui Chen , Shengqi Yin , Qiqing Guo , Yuchuan Chen , Jiaying Li , Yafang Wang , et al. 2025. “Gut‐Derived Lactobacillus From Exceptional Responders Mitigates Chemoradiotherapy‐Induced Intestinal Injury through Methionine‐Driven Epigenetic Modulation.” iMeta 4: e70043. 10.1002/imt2.70043 40469520 PMC12130557

[515] Westra, Harm‐Jan , Marta Martínez‐Bonet , Suna Onengut‐Gumuscu , Annette Lee , Yang Luo , Nikola Teslovich , Jane Worthington , et al. 2018. “Fine‐Mapping and Functional Studies Highlight Potential Causal Variants for Rheumatoid Arthritis and Type 1 Diabetes.” Nature Genetics 50: 1366–1374. 10.1038/s41588-018-0216-7 30224649 PMC6364548

[516] Nistico, L . 1996. “The CTLA‐4 Gene Region of Chromosome 2q33 Is Linked to, and Associated With, Type 1 Diabetes. Belgian Diabetes Registry.” Human Molecular Genetics 5: 1075–1080. 10.1093/hmg/5.7.1075 8817351

[517] Jostins, Luke , Stephan Ripke , Rinse K. Weersma , Richard H. Duerr , Dermot P. McGovern , Ken Y. Hui , James C. Lee , et al. 2012. “Host‐Microbe Interactions Have Shaped the Genetic Architecture of Inflammatory Bowel Disease.” Nature 491: 119–124. 10.1038/nature11582 23128233 PMC3491803

[518] Lincoln, Matthew R. , Noah Connally , Pierre‐Paul Axisa , Christiane Gasperi , Mitja Mitrovic , David van Heel , Cisca Wijmenga , et al. 2024. “Genetic Mapping Across Autoimmune Diseases Reveals Shared Associations and Mechanisms.” Nature Genetics 56: 838–845. 10.1038/s41588-024-01732-8 38741015 PMC13095137

[519] Hu, Nan , Xiangning Qiu , Yongqi Luo , Jun Yuan , Yaping Li , Wenzhi Lei , Guiying Zhang , et al. 2008. “Abnormal Histone Modification Patterns in Lupus CD4+ T Cells.” Journal of Rheumatology 35: 804–810. http://www.jrheum.org/content/35/5/804.abstract 18398941

[520] Garaud, Soizic , Christelle Le Dantec , Sandrine Jousse‐Joulin , Catherine Hanrotel‐Saliou , Alain Saraux , Rizgar A. Mageed , Pierre Youinou , and Yves Renaudineau . 2009. “IL‐6 Modulates CD5 Expression in B Cells From Patients With Lupus by Regulating DNA Methylation.” Journal of Immunology 182: 5623–5632. 10.4049/jimmunol.0802412 19380809

[521] Feng, Ruoqing , Lena Spieth , Lu Liu , Stefan Berghoff , Jonas Franz , Qian Liu , Zhen Wang , et al. 2025. “Single‐Cell Spatial Transcriptomic Profiling Defines a Pathogenic Inflammatory Niche in Chronic Active Multiple Sclerosis Lesions.” Immunity 58: 2989–3005. 10.1016/j.immuni.2025.10.003 41167189

[522] Balasubramanian, Preetha , Uthra Balaji , Marina Silva Santos , Jeanine Baisch , Cynthia Smitherman , Lynnette Walters , Paola Sparagana , et al. 2025. “Single‐Cell RNA Profiling of Blood CD4(+) T Cells Identifies Distinct Helper and Dysfunctional Regulatory Clusters in Children With SLE.” Nature Immunology 26: 2100–2111. 10.1038/s41590-025-02297-2 41120754 PMC13034613

[523] Jäkel, Sarah , Eneritz Agirre , Ana Mendanha Falcão , David van Bruggen , Ka Wai Lee , Irene Knuesel , Dheeraj Malhotra , et al. 2019. “Altered Human Oligodendrocyte Heterogeneity in Multiple Sclerosis.” Nature 566: 543–547. 10.1038/s41586-019-0903-2 30747918 PMC6544546

[524] Zhu, Chenxi , Jiayi Xu , Siyu He , Qian Niu , Yi Liu , Xin Guo , Huifang Hu , et al. 2025. “A Longitudinal Study Reveals Metabolomic Markers for Individuals at Risk, Disease Severity, and Treatment Response in Rheumatoid Arthritis.” Advanced Science 12: e04414. 10.1002/advs.202504414 40801465 PMC12520550

[525] Xu, Henan , Xiao Zhang , Xin Wang , Bo Li , Hang Yu , Yuan Quan , Yan Jiang , et al. 2024. “Cellular Spermine Targets JAK Signaling to Restrain Cytokine‐Mediated Autoimmunity.” Immunity 57: 1796–1811.e8. 10.1016/j.immuni.2024.05.025 38908373

[526] Dai, Xiaofeng , Yuting Fan , and Xing Zhao . 2025. “Systemic Lupus Erythematosus: Updated Insights on the Pathogenesis, Diagnosis, Prevention and Therapeutics.” Signal Transduction and Targeted Therapy 10: 102. 10.1038/s41392-025-02168-0 40097390 PMC11914703

[527] Hosang, Leon , Roger Cugota Canals , Felicia Joy van der Flier , Jacqueline Hollensteiner , Rolf Daniel , Alexander Flügel , and Francesca Odoardi . 2022. “The Lung Microbiome Regulates Brain Autoimmunity.” Nature 603: 138–144. 10.1038/s41586-022-04427-4 35197636

[528] Lin, Xu , Zuxiang Yu , Yang Liu , Changzhou Li , Hui Hu , Jia‐Chun Hu , Mian Liu , et al. 2025. “Gut‐X Axis.” iMeta 4: e270. 10.1002/imt2.270 40027477 PMC11865426

[529] Goswami, Pawan Kumar , and Sandip Chatterjee . 2025. “Biological Multi‐Omics Approaches to Next‐Generation Biomarkers in Immune‐Related Disorders and Malignancies: an Overview.” Clinical and Translational Oncology 28: 1570–1585. 10.1007/s12094-025-04128-0 41324821

[530] Akif, Adnan , Mohammad Fahim Kadir , and Md Rabiul Islam . 2026. “Interleukins in Major Depressive Disorder: Lessons From Autoimmune Diseases and Pathways to Clinical Translation.” CNS Neuroscience & Therapeutics 32: e70791. 10.1002/cns.70791 41700330 PMC12910255

[531] Ageeva, Tatyana , Albert Rizvanov , and Yana Mukhamedshina . 2024. “NF‐κB and JAK/STAT Signaling Pathways as Crucial Regulators of Neuroinflammation and Astrocyte Modulation in Spinal Cord Injury.” Cells 13: 581. 10.3390/cells13070581 38607020 PMC11011519

[532] Klarquist, Jared , Rachel Cantrell , Maria A. Lehn , Kristin Lampe , Cassandra M. Hennies , Kasper Hoebe , and Edith M. Janssen . 2020. “Type I IFN Drives Experimental Systemic Lupus Erythematosus by Distinct Mechanisms in CD4 T Cells and B Cells.” ImmunoHorizons 4: 140–152. 10.4049/immunohorizons.2000005 32161059 PMC7294741

[533] Trzupek, Dominik , Mercede Lee , Fiona Hamey , Linda S. Wicker , John A. Todd , and Ricardo C. Ferreira . 2021. “Single‐Cell Multi‐Omics Analysis Reveals IFN‐driven Alterations in T Lymphocytes and Natural Killer Cells in Systemic Lupus Erythematosus.” Wellcome Open Research 6: 149. 10.12688/wellcomeopenres.16883.1 35509371 PMC9046903

[534] Masuo, Yuki , Akinori Murakami , Rinko Akamine , Osamu Iri , Shunsuke Uno , Koichi Murata , Kohei Nishitani , et al. 2025. “Stem‐Like and Effector Peripheral Helper T Cells Comprise Distinct Subsets in Rheumatoid Arthritis.” Science Immunology 10: eadt3955. 10.1126/sciimmunol.adt3955 40815671

[535] Xia, Xuyang , Chenjia He , Zhinan Xue , Yuelan Wang , Yun Qin , Zhixiang Ren , Yupeng Huang , et al. 2025. “Single Cell Immunoprofile of Synovial Fluid in Rheumatoid Arthritis With TNF/JAK Inhibitor Treatment.” Nature Communications 16: 2152. 10.1038/s41467-025-57361-0 PMC11880340 40038288

[536] Zhang, Fan , Anna Helena Jonsson , Aparna Nathan , Nghia Millard , Michelle Curtis , Qian Xiao , Maria Gutierrez‐Arcelus , et al. 2023. “Deconstruction of Rheumatoid Arthritis Synovium Defines Inflammatory Subtypes.” Nature 623: 616–624. 10.1038/s41586-023-06708-y 37938773 PMC10651487

[537] Goto, Manaka , Hideyuki Takahashi , Ryochi Yoshida , Takahiro Itamiya , Masahiro Nakano , Yasuo Nagafuchi , Hiroaki Harada , et al. 2024. “Age‐Associated CD4(+) T Cells With B Cell‐Promoting Functions Are Regulated by ZEB2 in Autoimmunity.” Science Immunology 9: eadk1643. 10.1126/sciimmunol.adk1643 38330141

[538] Gjurgjaj, Altea , and Cecilia Domínguez Conde . 2025. “Narrowing Down Key Players in Autoimmunity via Single‐Cell Multiomics.” European Journal of Immunology 55: e51233. 10.1002/eji.202451233 40534591 PMC12177686

[539] Sun, Shao‐Cong , Jae‐Hoon Chang , and Jin Jin . 2013. “Regulation of Nuclear Factor‐κB in Autoimmunity.” Trends in Immunology 34: 282–289. 10.1016/j.it.2013.01.004 23434408 PMC3664242

[540] Zarrin, Ali A. , Katherine Bao , Patrick Lupardus , and Domagoj Vucic . 2021. “Kinase Inhibition in Autoimmunity and Inflammation.” Nature Reviews Drug Discovery 20: 39–63. 10.1038/s41573-020-0082-8 33077936 PMC7569567

[541] Patel, Chirag H. , and Jonathan D. Powell . 2025. “More TOR: the Expanding Role of mTOR in Regulating Immune Responses.” Immunity 58: 1629–1645. 10.1016/j.immuni.2025.06.010 40592340

[542] Ahmed, Anees , and Gregory F. Sonnenberg . 2026. “Interleukin‐23 Biology Linking Mucosal Immunity to Autoimmune Diseases and Cancer.” Trends in Immunology 47: 397–408. 10.1016/j.it.2025.12.005 41483965

[543] Luo, Qiong , Yijun Liu , Ke Shi , Xuecheng Shen , Yaqi Yang , Xuejiao Liang , Liangliang Lu , et al. 2023. “An Autonomous Activation of Interleukin‐17 Receptor Signaling Sustains Inflammation and Promotes Disease Progression.” Immunity 56: 2006–2020.e6. 10.1016/j.immuni.2023.06.012 37473759

[544] Li, Qi , Xiaoyu Li , Yunwei Lou , Yingying Xing , Wei Yan , Yuan Chen , Guohong Hu , et al. 2026. “The IL17REL Gene Encodes a Decoy Receptor of IL‐17 Family Cytokines to Control Gut Inflammation.” Nature Immunology 27: 225–235. 10.1038/s41590-025-02368-4 41495316

[545] Franke, Andre , Tobias Balschun , Christian Sina , David Ellinghaus , Robert Häsler , Gabriele Mayr , Mario Albrecht , et al. 2010. “Genome‐Wide Association Study for Ulcerative Colitis Identifies Risk Loci at 7q22 and 22q13 (IL17REL).” Nature Genetics 42: 292–294. 10.1038/ng.553 20228798

[546] Zhuang, Yan , Yongcui Yan , Zheng Wen , Xiaoquan Rao , Jiangang Jiang , Huihui Li , and Dao Wen Wang . 2026. “Rapamycin Preserves Cardiac Function in Autoimmune Myocarditis by Reprogramming Cxcl9(+) Macrophages via the mTORC1‐C/EBPβ‐OSM Axis.” Redox Biology 89: 103970. 10.1016/j.redox.2025.103970 41412038 PMC12771342

[547] Gieseck, Richard L. , Mark S. Wilson , and Thomas A. Wynn . 2018. “Type 2 Immunity in Tissue Repair and Fibrosis.” Nature Reviews Immunology 18: 62–76. 10.1038/nri.2017.90 28853443

[548] Pisetsky, David S. , Amanda M. Eudy , Jennifer L. Rogers , Ru‐Rong Ji , Katherine T. Martucci , Camilla Svensson , and Peter E. Lipsky . 2025. “Pain in Systemic Lupus Erythematosus: Emerging Insights and Paradigms.” Nature Reviews Rheumatology 21: 626–639. 10.1038/s41584-025-01290-1 40858989

[549] Cortez, Victor S. , Sara Viragova , Satoshi Koga , Meizi Liu , Claire E. O'Leary , Roberto R. Ricardo‐Gonzalez , Andrew W. Schroeder , et al. 2025. “IL‐25‐induced Memory Type 2 Innate Lymphoid Cells Enforce Mucosal Immunity.” Cell 188: 6220–6235.e22. 10.1016/j.cell.2025.08.017 40914159 PMC13056343

[550] Annunziato, Francesco , Chiara Romagnani , and Sergio Romagnani . 2015. “The 3 Major Types of Innate and Adaptive Cell‐Mediated Effector Immunity.” Journal of Allergy and Clinical Immunology 135: 626–635. 10.1016/j.jaci.2014.11.001 25528359

[551] Vieujean, Sophie , Vipul Jairath , Laurent Peyrin‐Biroulet , Marla Dubinsky , Marietta Iacucci , Fernando Magro , and Silvio Danese . 2025. “Understanding the Therapeutic Toolkit for Inflammatory Bowel Disease.” Nature Reviews Gastroenterology & Hepatology 22: 371–394. 10.1038/s41575-024-01035-7 39891014

[552] Gronke, Konrad , Pedro P. Hernández , Jakob Zimmermann , Christoph S. N. Klose , Michael Kofoed‐Branzk , Fabian Guendel , Mario Witkowski , et al. 2019. “Interleukin‐22 Protects Intestinal Stem Cells against Genotoxic Stress.” Nature 566: 249–253. 10.1038/s41586-019-0899-7 30700914 PMC6420091

[553] Ahmed, Anees , Ann M. Joseph , Jordan Zhou , Veronika Horn , Jazib Uddin , Mengze Lyu , Jeremy Goc , et al. 2024. “CTLA‐4‐Expressing ILC3s Restrain Interleukin‐23‐Mediated Inflammation.” Nature 630: 976–983. 10.1038/s41586-024-07537-3 38867048 PMC11298788

[554] Cadinu, Paolo , Kisha N. Sivanathan , Aditya Misra , Rosalind J. Xu , Davide Mangani , Evan Yang , Joseph M. Rone , et al. 2024. “Charting the Cellular Biogeography in Colitis Reveals Fibroblast Trajectories and Coordinated Spatial Remodeling.” Cell 187: 2010–2028.e30. 10.1016/j.cell.2024.03.013 38569542 PMC11017707

[555] Hu, Xiaoyi , Jing li , Maorong Fu , Xia Zhao , and Wei Wang . 2021. “The JAK/STAT Signaling Pathway: From Bench to Clinic.” Signal Transduction and Targeted Therapy 6: 402. 10.1038/s41392-021-00791-1 34824210 PMC8617206

[556] Song, Yi , Jian Li , and Yuzhang Wu . 2024. “Evolving Understanding of Autoimmune Mechanisms and New Therapeutic Strategies of Autoimmune Disorders.” Signal Transduction and Targeted Therapy 9: 263. 10.1038/s41392-024-01952-8 39362875 PMC11452214

[557] Theofilopoulos, Argyrios N. , Dwight H. Kono , and Roberto Baccala . 2017. “The Multiple Pathways to Autoimmunity.” Nature Immunology 18: 716–724. 10.1038/ni.3731 28632714 PMC5791156

[558] Tran, Thao , Felicia Wee , Craig Ryan Joseph , Wanqiu Zhang , Jeffrey Chun Tatt Lim , Zhen Wei Neo , Li Yen Chong , et al. 2025. “An Integrated Approach for Analyzing Spatially Resolved Multi‐Omics Datasets From the Same Tissue Section.” Frontiers in Molecular Biosciences 12: 1614288. 10.3389/fmolb.2025.1614288 40735471 PMC12304548

[559] Kuo, David , Jennifer Ding , Ian S. Cohn , Fan Zhang , Kevin Wei , Deepak A. Rao , Cristina Rozo , et al. 2019. “HBEGF(+) Macrophages in Rheumatoid Arthritis Induce Fibroblast Invasiveness.” Science Translational Medicine 11: eaau8587. 10.1126/scitranslmed.aau8587 31068444 PMC6726376

[560] Falcão, Ana Mendanha , David van Bruggen , Sueli Marques , Mandy Meijer , Sarah Jäkel , Eneritz Agirre , Samudyata , et al. 2018. “Disease‐Specific Oligodendrocyte Lineage Cells Arise in Multiple Sclerosis.” Nature Medicine 24: 1837–1844. 10.1038/s41591-018-0236-y PMC6544508 30420755

[561] Mayassi, Toufic , Chenhao Li , Åsa Segerstolpe , Eric M. Brown , Rebecca Weisberg , Toru Nakata , Hiroshi Yano , et al. 2024. “Spatially Restricted Immune and Microbiota‐Driven Adaptation of the Gut.” Nature 636: 447–456. 10.1038/s41586-024-08216-z 39567686 PMC11816900

[562] Hao, Gaoyang , Yi Fan , Zhuohan Yu , Yanchi Su , Haoran Zhu , Fuzhou Wang , Xingjian Chen , et al. 2025. “Topological Identification and Interpretation for Single‐Cell Epigenetic Regulation Elucidation in Multi‐Tasks Using scAGDE.” Nature Communications 16: 1691. 10.1038/s41467-025-57027-x PMC11830825 39956806

[563] Joulia, Régis , Sara Patti , William J. Traves , Lola Loewenthal , Laura Yates , Simone A. Walker , Franz Puttur , et al. 2025. “A Single‐Cell Spatial Chart of the Airway Wall Reveals Proinflammatory Cellular Ecosystems and Their Interactions in Health and Asthma.” Nature Immunology 26: 920–933. 10.1038/s41590-025-02161-3 40399607 PMC12133579

[564] Shi, Jialu , Wenjun Mao , Yuqing Song , Yuxin Wang , Lili Zhang , Yan Xu , Huiwen Gu , et al. 2025. “Butyrate Alleviates Food Allergy by Improving Intestinal Barrier Integrity Through Suppressing Oxidative Stress‐Mediated Notch Signaling.” iMeta 4: e70024. 10.1002/imt2.70024 40469511 PMC12130580

[565] Sun, Lele , Pengcheng Huai , Zhenzhen Wang , Qing Zhao , Yingjie Lin , Tingting Liu , Xiaotong Xue , et al. 2025. “TSC22 Domain Family Member 3 Links Natural Killer Cells to CD8+ T Cell‐Mediated Drug Hypersensitivity.” Signal Transduction and Targeted Therapy 10: 196. 10.1038/s41392-025-02300-0 40544157 PMC12182574

[566] Song, Woogil , Eunyoung Emily Lee , Seongwan Park , Baekgyu Choi , Min‐Gang Kim , Seo Yoon Ban , Se Rim Choi , et al. 2025. “Type 1 Interferon Signature and Allograft Inflammatory Factor‐1 Contribute to Refractoriness to TNF Inhibition in Ankylosing Spondylitis.” Nature Communications 16: 5531. 10.1038/s41467-025-60445-6 PMC12214652 40593526

[567] Wan, Yisong Y. , and Richard A. Flavell . 2008. “TGF‐β and Regulatory T Cell in Immunity and Autoimmunity.” Journal of Clinical Immunology 28: 647–659. 10.1007/s10875-008-9251-y 18792765 PMC2837280

[568] Barnabei, Laura , Emmanuel Laplantine , William Mbongo , Frédéric Rieux‐Laucat , and Robert Weil . 2021. “NF‐κB: At the Borders of Autoimmunity and Inflammation.” Frontiers in Immunology 12: 716469. 10.3389/fimmu.2021.716469 34434197 PMC8381650

[569] Thomas, Tom , Matthias Friedrich , Charlotte Rich‐Griffin , Mathilde Pohin , Devika Agarwal , Julia Pakpoor , Carl Lee , et al. 2024. “A Longitudinal Single‐Cell Atlas of Anti‐Tumour Necrosis Factor Treatment in Inflammatory Bowel Disease.” Nature Immunology 25: 2152–2165. 10.1038/s41590-024-01994-8 39438660 PMC11519010

[570] Mitchell, Patrick S. , Rachael K. Parkin , Evan M. Kroh , Brian R. Fritz , Stacia K. Wyman , Era L. Pogosova‐Agadjanyan , Amelia Peterson , et al. 2008. “Circulating microRNAs as Stable Blood‐Based Markers for Cancer Detection.” Proceedings of the National Academy of Sciences 105: 10513–10518. 10.1073/pnas.0804549105 PMC2492472 18663219

[571] Gjermeni, Erind , Raluca Fiebiger , Linnaeus Bundalian , Antje Garten , Torsten Schöneberg , Diana Le Duc , and Matthias Blüher . 2025. “The Impact of Dietary Interventions on Cardiometabolic Health.” Cardiovascular Diabetology 24: 234. 10.1186/s12933-025-02766-w 40450314 PMC12125738

[572] Tahir, Usman A. , and Robert E. Gerszten . 2023. “Molecular Biomarkers for Cardiometabolic Disease: Risk Assessment in Young Individuals.” Circulation Research 132: 1663–1673. 10.1161/CIRCRESAHA.123.322000 37289904 PMC10320574

[573] Joshi, Abhishek , Marieke Rienks , Konstantinos Theofilatos , and Manuel Mayr . 2021. “Systems Biology in Cardiovascular Disease: A Multiomics Approach.” Nature Reviews Cardiology 18: 313–330. 10.1038/s41569-020-00477-1 33340009

[574] Peterson, Linda R. , and Robert J. Gropler . 2020. “Metabolic and Molecular Imaging of the Diabetic Cardiomyopathy.” Circulation Research 126: 1628–1645. 10.1161/CIRCRESAHA.120.315899 32437305 PMC8696978

[575] Tenenbaum, Alexander , Enrique Z. Fisman , Ehud Schwammenthal , Yehuda Adler , Michal Benderly , Michael Motro , and Joseph Shemesh . 2003. “Increased Prevalence of Left Ventricular Hypertrophy in Hypertensive Women With Type 2 Diabetes Mellitus.” Cardiovascular Diabetology 2: 14. 10.1186/1475-2840-2-14 14633284 PMC317343

[576] Wu, Teng , Tongsheng Huang , Honglin Ren , Conghui Shen , Jiang Qian , Xinlu Fu , Shangyuan Liu , et al. 2025. “Metabolic Coordination Structures Contribute to Diabetic Myocardial Dysfunction.” Circulation Research 136: 946–967. 10.1161/CIRCRESAHA.124.326044 40190276

[577] Murtaza, Ghulam , Hafeez Ul Hassan Virk , Muhammad Khalid , Carl J. Lavie , Hector Ventura , Debabrata Mukherjee , Vijay Ramu , et al. 2019. “Diabetic Cardiomyopathy ‐ A Comprehensive Updated Review.” Progress in Cardiovascular Diseases 62: 315–326. 10.1016/j.pcad.2019.03.003 30922976

[578] Gilbert, Jack A. , Martin J. Blaser , J. Gregory Caporaso , Janet K. Jansson , Susan V. Lynch , and Rob Knight . 2018. “Current Understanding of the Human Microbiome.” Nature Medicine 24: 392–400. 10.1038/nm.4517 PMC7043356 29634682

[579] Jin, Jing‐yu , Xin‐yu Yang , Ru Feng , Meng‐liang Ye , Hui Xu , Jing‐yue Wang , Jia‐chun Hu , et al. 2025. “Gut Microbiota‐Derived Metabolites Orchestrate Metabolic Reprogramming in Diabetic Cardiomyopathy: Mechanisms and Therapeutic Frontiers.” FASEB Journal 39: e71004. 10.1096/fj.202501579RR 40899744 PMC12406765

[580] Wang, Yu‐Chen , Yen Chin Koay , Calvin Pan , Zhiqiang Zhou , Wilson Tang , Jennifer Wilcox , Xinmin S. Li , et al. 2024. “Indole‐3‐Propionic Acid Protects Against Heart Failure With Preserved Ejection Fraction.” Circulation Research 134: 371–389. 10.1161/CIRCRESAHA.123.322381 38264909 PMC10923103

[581] Zhang, Han , Junbao Du , Yaqian Huang , Chaoshu Tang , and Hongfang Jin . 2023. “Hydrogen Sulfide Regulates Macrophage Function in Cardiovascular Diseases.” Antioxidants & Redox Signaling 38: 45–56. 10.1089/ars.2022.0075 35658575

[582] Tao, Zhipeng , and Yao Wang . 2025. “The Health Benefits of Dietary Short‐Chain Fatty Acids in Metabolic Diseases.” Critical Reviews in Food Science and Nutrition 65: 1579–1592. 10.1080/10408398.2023.2297811 38189336

[583] Krautkramer, Kimberly A. , Jing Fan , and Fredrik Bäckhed . 2021. “Gut Microbial Metabolites as Multi‐Kingdom Intermediates.” Nature Reviews Microbiology 19: 77–94. 10.1038/s41579-020-0438-4 32968241

[584] Avlas, Orna , Reut Fallach , Asher Shainberg , Eyal Porat , and Edith Hochhauser . 2011. “Toll‐Like Receptor 4 Stimulation Initiates an Inflammatory Response That Decreases Cardiomyocyte Contractility.” Antioxidants & Redox Signaling 15: 1895–1909. 10.1089/ars.2010.3728 21126202

[585] Chen, Sifan , Ayana Henderson , Michael C. Petriello , Kymberleigh A. Romano , Mary Gearing , Ji Miao , Mareike Schell , et al. 2019. “Trimethylamine N‐Oxide Binds and Activates PERK to Promote Metabolic Dysfunction.” Cell Metabolism 30: 1141–1151. 10.1016/j.cmet.2019.08.021 31543404

[586] Zheng, Hong , Xi Zhang , Chen Li , Die Wang , Yuying Shen , Jiahui Lu , Liangcai Zhao , Xiaokun Li , and Hongchang Gao . 2024. “BCAA Mediated Microbiota‐Liver‐Heart Crosstalk Regulates Diabetic Cardiomyopathy Via FGF21.” Microbiome 12: 157. 10.1186/s40168-024-01872-3 39182099 PMC11344321

[587] Depommier, Clara , Amandine Everard , Céline Druart , Dominique Maiter , Jean‐Paul Thissen , Audrey Loumaye , Michel P. Hermans , et al. 2021. “Serum Metabolite Profiling Yields Insights Into Health Promoting Effect of A. Muciniphila in Human Volunteers With a Metabolic Syndrome.” Gut Microbes 13: 1994270. 10.1080/19490976.2021.1994270 34812127 PMC8632301

[588] Yang, Caixin , Ruiting Lan , Lijun Zhao , Ji Pu , Dalong Hu , Jing Yang , Huimin Zhou , et al. 2024. “Prevotella Copri Alleviates Hyperglycemia and Regulates Gut Microbiota and Metabolic Profiles in Mice.” mSystems 9: e0053224. 10.1128/msystems.00532-24 38934548 PMC11265406

[589] Guo, Chuansheng , Shujun Xie , Zhexu Chi , Jinhua Zhang , Yangyang Liu , Li Zhang , Mingzhu Zheng , et al. 2016. “Bile Acids Control Inflammation and Metabolic Disorder through Inhibition of NLRP3 Inflammasome.” Immunity 45: 802–816. 10.1016/j.immuni.2016.09.008 27692610

[590] Ridlon, Jason M. , Saravanan Devendran , João Mp Alves , Heidi Doden , Patricia G. Wolf , Gabriel V. Pereira , Lindsey Ly , et al. 2020. “The ‘In Vivolifestyle’ of Bile Acid 7α‐Dehydroxylating Bacteria: Comparative Genomics, Metatranscriptomic, and Bile Acid Metabolomics Analysis of a Defined Microbial Community in Gnotobiotic Mice.” Gut Microbes 11: 381–404. 10.1080/19490976.2019.1618173 31177942 PMC7524365

[591] Yao, Weiyi , Xinting Hu , and Xin Wang . 2024. “Crossing Epigenetic Frontiers: the Intersection of Novel Histone Modifications and Diseases.” Signal Transduction and Targeted Therapy 9: 232. 10.1038/s41392-024-01918-w 39278916 PMC11403012

[592] Kuppe, Christoph , Ricardo O. Ramirez Flores , Zhijian Li , Sikander Hayat , Rebecca T. Levinson , Xian Liao , Monica T. Hannani , et al. 2022. “Spatial Multi‐Omic Map of Human Myocardial Infarction.” Nature 608: 766–777. 10.1038/s41586-022-05060-x 35948637 PMC9364862

[593] Serrano‐Gómez, Gerard , Francisca Yañez , Zaida Soler , Marc Pons‐Tarin , Luis Mayorga , Claudia Herrera‐deGuise , Natalia Borruel , et al. 2025. “Microbiome Multi‐Omics Analysis Reveals Novel Biomarkers and Mechanisms Linked With CD Etiopathology.” Biomarker Research 13: 85. 10.1186/s40364-025-00802-1 40524271 PMC12172205

[594] Pechlaner, Raimund , Stefan Kiechl , and Manuel Mayr . 2016. “Potential and Caveats of Lipidomics for Cardiovascular Disease.” Circulation 134: 1651–1654. 10.1161/CIRCULATIONAHA.116.025092 27756782

[595] Li, Leyuan , Zhibin Ning , Kai Cheng , Xu Zhang , Caitlin M. A. Simopoulos , and Daniel Figeys . 2022. “iMetaLab Suite: A One‐Stop Toolset for Metaproteomics.” iMeta 1: e25. 10.1002/imt2.25 38868572 PMC10989937

[596] Wang, Hu , Jiaxing Wang , Hao Cui , Chenyu Fan , Yuzhou Xue , Huiying Liu , Hui Li , et al. 2024. “Inhibition of Fatty Acid Uptake by TGR5 Prevents Diabetic Cardiomyopathy.” Nature Metabolism 6: 1161–1177. 10.1038/s42255-024-01036-5 PMC11199146 38698281

[597] Tahir, Usman A. , and Robert E. Gerszten . 2020. “Omics and Cardiometabolic Disease Risk Prediction.” Annual Review of Medicine 71: 163–175. 10.1146/annurev-med-042418-010924 31986080

[598] Jiang, Xuanwei , Guangrui Yang , Meng Chen , Nannan Feng , Lan Xu , Xihao Du , Chunlai Zeng , and Victor W. Zhong . 2025. “Multiomics Insight Into Disease Trajectories of Cardiometabolic Diseases and Cancer.” Nature Communications 17: 813. 10.1038/s41467-025-67510-0 PMC12824212 41408044

[599] Martin, Alicia R. , Masahiro Kanai , Yoichiro Kamatani , Yukinori Okada , Benjamin M. Neale , and Mark J. Daly . 2019. “Clinical Use of Current Polygenic Risk Scores May Exacerbate Health Disparities.” Nature Genetics 51: 584–591. 10.1038/s41588-019-0379-x 30926966 PMC6563838

[600] Rosenberg, Noah A. , Lucy Huang , Ethan M. Jewett , Zachary A. Szpiech , Ivana Jankovic , and Michael Boehnke . 2010. “Genome‐Wide Association Studies in Diverse Populations.” Nature Reviews Genetics 11: 356–366. 10.1038/nrg2760 PMC3079573 20395969

[601] Abul‐Husn, Noura S. , Kandamurugu Manickam , Laney K. Jones , Eric A. Wright , Dustin N. Hartzel , Claudia Gonzaga‐Jauregui , Colm O'Dushlaine , et al. 2016. “Genetic Identification of Familial Hypercholesterolemia Within a Single U.S. Health Care System.” Science 354: aaf7000. 10.1126/science.aaf7000 28008010

[602] Lotta, Luca A. , Robert A. Scott , Stephen J. Sharp , Stephen Burgess , Jian'an Luan , Therese Tillin , Amand F. Schmidt , et al. 2016. “Genetic Predisposition to an Impaired Metabolism of the Branched‐Chain Amino Acids and Risk of Type 2 Diabetes: A Mendelian Randomisation Analysis.” PLOS Medicine 13: e1002179. 10.1371/journal.pmed.1002179 27898682 PMC5127513

[603] Patanè, Salvatore . 2020. “Long‐Term Changes in Gut Microbial Metabolite TMAO, CHD Risk, and Its Complex Regulatory Network.” Journal of the American College of Cardiology 75: 3100–3101. 10.1016/j.jacc.2020.03.077 32553264

[604] Wang, Zeneng , Elizabeth Klipfell , Brian J. Bennett , Robert Koeth , Bruce S. Levison , Brandon DuGar , Ariel E. Feldstein , et al. 2011. “Gut Flora Metabolism of Phosphatidylcholine Promotes Cardiovascular Disease.” Nature 472: 57–63. 10.1038/nature09922 21475195 PMC3086762

[605] Chechi, Kanta , Rima Chakaroun , Antonis Myridakis , Sofia K. Forslund‐Startceva , Sebastien Fromentin , Trine Nielsen , Judith Aron‐Wisneswky , et al. 2026. “A Gut Microbiome‐Kidney‐Heart Axis Predictive of Future Cardiovascular Diseases.” Nature Communications 17: 3477. 10.1038/s41467-026-69405-0 PMC13079829 41786749

[606] Talmor‐Barkan, Yeela , Noam Bar , Aviv A. Shaul , Nir Shahaf , Anastasia Godneva , Yuval Bussi , Maya Lotan‐Pompan , et al. 2022. “Metabolomic and Microbiome Profiling Reveals Personalized Risk Factors for Coronary Artery Disease.” Nature Medicine 28: 295–302. 10.1038/s41591-022-01686-6 PMC12365913 35177859

[607] Lam, Maggie P. Y. , Peipei Ping , and Elizabeth Murphy . 2016. “Proteomics Research in Cardiovascular Medicine and Biomarker Discovery.” Journal of the American College of Cardiology 68: 2819–2830. 10.1016/j.jacc.2016.10.031 28007144 PMC5189682

[608] Lindsey, Merry L. , Manuel Mayr , Aldrin V. Gomes , Christian Delles , D. Kent Arrell , Anne M. Murphy , Richard A. Lange , et al. 2015. “Transformative Impact of Proteomics on Cardiovascular Health and Disease: A Scientific Statement From the American Heart Association.” Circulation 132: 852–872. 10.1161/CIR.0000000000000226 26195497

[609] Nurmohamed, Nick S. , Jordan M. Kraaijenhof , Manuel Mayr , Stephen J. Nicholls , Wolfgang Koenig , Alberico L. Catapano , and Erik S. G. Stroes . 2023. “Proteomics and Lipidomics in Atherosclerotic Cardiovascular Disease Risk Prediction.” European Heart Journal 44: 1594–1607. 10.1093/eurheartj/ehad161 36988179 PMC10163980

[610] Unterhuber, Matthias , Karl‐Patrik Kresoja , Karl‐Philipp Rommel , Christian Besler , Andrea Baragetti , Nora Klöting , Uta Ceglarek , et al. 2021. “Proteomics‐Enabled Deep Learning Machine Algorithms Can Enhance Prediction of Mortality.” Journal of the American College of Cardiology 78: 1621–1631. 10.1016/j.jacc.2021.08.018 34649700

[611] Nurmohamed, Nick S. , João P. Belo Pereira , Renate M. Hoogeveen , Jeffrey Kroon , Jordan M. Kraaijenhof , Farahnaz Waissi , Nathalie Timmerman , et al. 2022. “Targeted Proteomics Improves Cardiovascular Risk Prediction in Secondary Prevention.” European Heart Journal 43: 1569–1577. 10.1093/eurheartj/ehac055 35139537 PMC9020984

[612] Versnjak, Jakob , Titus Kuehne , Pauline Fahjen , Nina Jovanovic , Ulrike Löber , Gabriele G. Schiattarella , Nicola Wilck , et al. 2025. “Deep Phenotyping of Heart Failure With Preserved Ejection Fraction through Multi‐Omics Integration.” European Journal of Heart Failure 27: 3243–3259. 10.1002/ejhf.70041 40977229 PMC12803610

[613] Fruman, David A. , Honyin Chiu , Benjamin D. Hopkins , Shubha Bagrodia , Lewis C. Cantley , and Robert T. Abraham . 2017. “The PI3K Pathway in Human Disease.” Cell 170: 605–635. 10.1016/j.cell.2017.07.029 28802037 PMC5726441

[614] Ren, Bin‐cheng , Yu‐fei Zhang , Shan‐shan Liu , Xiao‐jing Cheng , Xin Yang , Xiao‐guang Cui , Xin‐rui Zhao , et al. 2020. “Curcumin Alleviates Oxidative Stress and Inhibits Apoptosis in Diabetic Cardiomyopathy via Sirt1‐Foxo1 and PI3K‐Akt Signalling Pathways.” Journal of Cellular and Molecular Medicine 24: 12355–12367. 10.1111/jcmm.15725 32961025 PMC7687015

[615] Thomas, M. C. , J. Söderlund , M. Lehto , V.‐P. Mäkinen , J. L. Moran , M. E. Cooper , C. Forsblom , and P.‐H. Groop . 2011. “Soluble Receptor for AGE (RAGE) Is a Novel Independent Predictor of All‐Cause and Cardiovascular Mortality in Type 1 Diabetes.” Diabetologia 54: 2669–2677. 10.1007/s00125-011-2186-5 21607631

[616] Würtz, Peter , Aki S. Havulinna , Pasi Soininen , Tuulia Tynkkynen , David Prieto‐Merino , Therese Tillin , Anahita Ghorbani , et al. 2015. “Metabolite Profiling and Cardiovascular Event Risk: A Prospective Study of 3 Population‐Based Cohorts.” Circulation 131: 774–785. 10.1161/CIRCULATIONAHA.114.013116 25573147 PMC4351161

[617] Osório, Thiago Guimarães , Estefania Pavesi , Khalil Abou El‐Ardat , Needa Qureshi , Leanne Cassidy , Terrence Lee St. John , Nicole Sirotin , and Bartlomiej Piechowski‐Jozwiak . 2025. “Single‐Cell Multi‐Omics for Precision Cardiovascular and Longevity Medicine: From Methods to Clinical Translation.” Frontiers in Aging 6: 1656727. 10.3389/fragi.2025.1656727 41393100 PMC12696164

[618] Mosquera, Jose Verdezoto , Gaëlle Auguste , Doris Wong , Adam W. Turner , Chani J. Hodonsky , Astrid Catalina Alvarez‐Yela , Yipei Song , et al. 2023. “Integrative Single‐Cell Meta‐Analysis Reveals Disease‐Relevant Vascular Cell States and Markers in Human Atherosclerosis.” Cell Reports 42: 113380. 10.1016/j.celrep.2023.113380 37950869 PMC12335892

[619] Bajinka, Ousman , Lamarana Jallow , Yanjun Zhang , Xiaoxia Feng , Xianquan Zhan , and Na Li . 2025. “The Use of Predictive, Preventive, and Personalized Medical Approaches to Optimize Hypertension Management.” EPMA Journal 16: 785–804. 10.1007/s13167-025-00427-2 41311994 PMC12647437

[620] Collier, David J. , Mike Taylor , Thomas Godec , Julian Shiel , Rebecca James , Yasmin Chowdury , Patrizia Ebano , et al. 2024. “Personalized Antihypertensive Treatment Optimization With Smartphone‐Enabled Remote Precision Dosing of Amlodipine During the COVID‐19 Pandemic (PERSONAL‐CovidBP Trial).” Journal of the American Heart Association 13: e030749. 10.1161/JAHA.123.030749 38323513 PMC11010092

[621] Ferrannini, Ele , and Anna Solini . 2012. “SGLT2 Inhibition in Diabetes Mellitus: Rationale and Clinical Prospects.” Nature Reviews Endocrinology 8: 495–502. 10.1038/nrendo.2011.243 22310849

[622] Lundberg, Emma , and Georg H. H. Borner . 2019. “Spatial Proteomics: A Powerful Discovery Tool for Cell Biology.” Nature Reviews Molecular Cell Biology 20: 285–302. 10.1038/s41580-018-0094-y 30659282

[623] Chen, Xing , Luying Zhang , and Wen Chen . 2025. “Global, Regional, and National Burdens of Type 1 and Type 2 Diabetes Mellitus in Adolescents From 1990 to 2021, With Forecasts to 2030: A Systematic Analysis of the Global Burden of Disease Study 2021.” BMC Medicine 23: 48. 10.1186/s12916-025-03890-w 39876009 PMC11776159

[624] Ng, Marie , Emmanuela Gakidou , Justin Lo , Yohannes Habtegiorgis Abate , Cristiana Abbafati , Nasir Abbas , Mohammadreza Abbasian , et al. 2025. “Global, Regional, and National Prevalence of Adult Overweight and Obesity, 1990–2021, With Forecasts to 2050: A Forecasting Study for the Global Burden of Disease Study 2021.” Lancet 405: 813–838. 10.1016/S0140-6736(25)00355-1 40049186 PMC11920007

[625] Stafford, Lauryn K. , Anna Gage , Yvonne Yiru Xu , Madeleine Conrad , Ismael Barreras Beltran , Edward J. Boyko , Bruce B. Duncan , et al. 2025. “Global, Regional, and National Cascades of Diabetes Care, 2000‐23: A Systematic Review and Modelling Analysis Using Findings From the Global Burden of Disease Study.” Lancet Diabetes & Endocrinology 13: 924–934. 10.1016/s2213-8587(25)00217-7 40934935 PMC13537427

[626] Xie, Zhaomin , Chaolun Yu , Qingmei Cui , Xirui Zhao , Juncheng Zhuang , Shiqun Chen , Haixia Guan , and Jie Li . 2025. “Global Burden of the Key Components of Cardiovascular‐Kidney‐Metabolic Syndrome.” Journal of the American Society of Nephrology 36: 1572–1584. 10.1681/asn.0000000658 40042920 PMC12342072

[627] Choi, Ye Ji , Gabriel Richard , Guanshi Zhang , Jeffrey B. Hodgin , Dawit S. Demeke , Yingbao Yang , Jennifer A. Schaub , et al. 2024. “Attenuated Kidney Oxidative Metabolism in Young Adults With Type 1 Diabetes.” Journal of Clinical Investigation 134: e183984. 10.1172/jci183984 39436695 PMC11645151

[628] Fioretti, Francesco , Jeffrey M. Testani , Maria Clarissa Tio , Bertram Pitt , and Javed Butler . 2025. “Aldosterone and Aldosterone Modulation in Cardio‐Kidney Diseases.” Journal of the American College of Cardiology 86: 354–373. 10.1016/j.jacc.2025.06.012 40738563

[629] Lee, Lauren E. , Tomohito Doke , Dhanunjay Mukhi , and Katalin Susztak . 2024. “The Key Role of Altered Tubule Cell Lipid Metabolism in Kidney Disease Development.” Kidney International 106: 24–34. 10.1016/j.kint.2024.02.025 38614389 PMC11193624

[630] Herrington, William G. , Parminder K. Judge , Morgan E. Grams , and Christoph Wanner . 2026. “Chronic Kidney Disease.” Lancet 407: 90–104. 10.1016/s0140-6736(25)01942-7 41314225

[631] Bavanandan, Sunita , Irene L. Noronha , Irma Tchokhonelidze , and Adrian Liew . 2025. “Metabolic Disorder‐Associated Kidney Disease and Diabetic Kidney Disease in Developing Countries.” Kidney International 108: 380–387. 10.1016/j.kint.2025.03.034 40582409

[632] Eckardt, Kai‐Uwe , Seth L. Alper , Corinne Antignac , Anthony J. Bleyer , Dominique Chauveau , Karin Dahan , Constantinos Deltas , et al. 2015. “Autosomal Dominant Tubulointerstitial Kidney Disease: Diagnosis, Classification, and Management‐‐A KDIGO Consensus Report.” Kidney International 88: 676–683. 10.1038/ki.2015.28 25738250

[633] Knol, Martine G. E. , Vera C. Wulfmeyer , Roman‐Ulrich Müller , and Markus M. Rinschen . 2024. “Amino Acid Metabolism in Kidney Health and Disease.” Nature Reviews Nephrology 20: 771–788. 10.1038/s41581-024-00872-8 39198707

[634] Li, Jinxi , Ting Xiang , Fengping Zhang , Li Feng , Yiting Wu , Fan Guo , Lingzhi Li , Ping Fu , and Liang Ma . 2025. “Tubular ACSM3 Deficiency Impairs Medium‐Chain Fatty Acid Metabolism and Aggravates Kidney Fibrosis.” Proceedings of the National Academy of Sciences of the United States of America 122: e2505752122. 10.1073/pnas.2505752122 40953271 PMC12478119

[635] Li, Shen , Hongbo Liu , Hailong Hu , Eunji Ha , Praveena Prasad , Brenita C. Jenkins , Ujjalkumar Subhash Das , et al. 2025. “Human Genetics Identify Convergent Signals in Mitochondrial LACTB‐mediated Lipid Metabolism in Cardiovascular‐Kidney‐Metabolic Syndrome.” Cell Metabolism 37: 154–168.e7. 10.1016/j.cmet.2024.10.007 39561766 PMC11972450

[636] Basso, Paulo José , Vinicius Andrade‐Oliveira , and Niels Olsen Saraiva Câmara . 2021. “Targeting Immune Cell Metabolism in Kidney Diseases.” Nature Reviews Nephrology 17: 465–480. 10.1038/s41581-021-00413-7 33828286

[637] Fan, Xiaoting , Meilin Yang , Yating Lang , Shangwei Lu , Zhijuan Kong , Ying Gao , Ning Shen , Dongdong Zhang , and Zhimei Lv . 2024. “Mitochondrial Metabolic Reprogramming in Diabetic Kidney Disease.” Cell Death & Disease 15: 442. 10.1038/s41419-024-06833-0 38910210 PMC11194272

[638] Kishi, Seiji , Hajime Nagasu , Kengo Kidokoro , and Naoki Kashihara . 2024. “Oxidative Stress and the Role of Redox Signalling in Chronic Kidney Disease.” Nature Reviews Nephrology 20: 101–119. 10.1038/s41581-023-00775-0 37857763

[639] Martinez Leon, Victor , Rachel Hilburg , and Katalin Susztak . 2026. “Mechanisms of Diabetic Kidney Disease and Established and Emerging Treatments.” Nature Reviews Endocrinology 22: 21–35. 10.1038/s41574-025-01171-3 PMC13714763 40935879

[640] Hu, Hongtu , Weiwei Li , Yiqun Hao , Zhuan Peng , Zhengping Zou , Jiali Wei , Ying Zhou , Wei Liang , and Yun Cao . 2024. “The SGLT2 Inhibitor Dapagliflozin Ameliorates Renal Fibrosis in Hyperuricemic Nephropathy.” Cell Reports Medicine 5: 101690. 10.1016/j.xcrm.2024.101690 39168099 PMC11384938

[641] Francis, Anna , Meera N. Harhay , Albert C. M. Ong , Sri Lekha Tummalapalli , Alberto Ortiz , Agnes B. Fogo , Danilo Fliser , et al. 2024. “Chronic Kidney Disease and the Global Public Health Agenda: an International Consensus.” Nature Reviews Nephrology 20: 473–485. 10.1038/s41581-024-00820-6 38570631

[642] Bikbov, Boris , Caroline A. Purcell , Andrew S. Levey , Mari Smith , Amir Abdoli , Molla Abebe , Oladimeji M. Adebayo , et al. 2020. “Global, Regional, and National Burden of Chronic Kidney Disease, 1990–2017: A Systematic Analysis for the Global Burden of Disease Study 2017.” Lancet 395: 709–733. 10.1016/S0140-6736(20)30045-3 32061315 PMC7049905

[643] Aklilu, Abinet M . 2023. “Diagnosis of Chronic Kidney Disease and Assessing Glomerular Filtration Rate.” Medical Clinics of North America 107: 641–658. 10.1016/j.mcna.2023.03.001 37258004

[644] Zoccali, Carmine , Patrick B. Mark , Pantelis Sarafidis , Rajiv Agarwal , Marcin Adamczak , Rodrigo Bueno de Oliveira , Ziad A. Massy , et al. 2023. “Diagnosis of Cardiovascular Disease in Patients With Chronic Kidney Disease.” Nature Reviews Nephrology 19: 733–746. 10.1038/s41581-023-00747-4 37612381

[645] Neuen, Brendon L. , Emily K. Yeung , Janani Rangaswami , and Muthiah Vaduganathan . 2025. “Combination Therapy as a New Standard of Care in Diabetic and Non‐Diabetic Chronic Kidney Disease.” Nephrology Dialysis Transplantation 40: i59–i69. 10.1093/ndt/gfae258 PMC11795647 39907542

[646] Liu, Zhihua , Yahui Zhao , Pengzhou Kong , Yuhao Liu , Jing Huang , Enwei Xu , Wenqing Wei , et al. 2023. “Integrated Multi‐Omics Profiling Yields a Clinically Relevant Molecular Classification for Esophageal Squamous Cell Carcinoma.” Cancer Cell 41: 181–195.e9. 10.1016/j.ccell.2022.12.004 36584672

[647] Xing, Xiaohua , En Hu , Jiahe Ouyang , Xianyu Zhong , Fei Wang , Kaixin Liu , Linsheng Cai , et al. 2023. “Integrated Omics Landscape of Hepatocellular Carcinoma Suggests Proteomic Subtypes for Precision Therapy.” Cell Reports Medicine 4: 101315. 10.1016/j.xcrm.2023.101315 38091986 PMC10783603

[648] Iacucci, Marietta , Giovanni Santacroce , Irene Zammarchi , Yasuharu Maeda , Rocío Del Amor , Pablo Meseguer , Bisi Bode Kolawole , et al. 2024. “Artificial Intelligence and Endo‐Histo‐Omics: New Dimensions of Precision Endoscopy and Histology in Inflammatory Bowel Disease.” Lancet Gastroenterology & Hepatology 9: 758–772. 10.1016/s2468-1253(24)00053-0 38759661

[649] Eales, James M. , Xiao Jiang , Xiaoguang Xu , Sushant Saluja , Artur Akbarov , Eddie Cano‐Gamez , Michelle T. McNulty , et al. 2021. “Uncovering Genetic Mechanisms of Hypertension through Multi‐Omic Analysis of the Kidney.” Nature Genetics 53: 630–637. 10.1038/s41588-021-00835-w 33958779

[650] Lu, Yao‐Qi , and Yirong Wang . 2024. “Multi‐Omic Analysis Reveals Genetic Determinants and Therapeutic Targets of Chronic Kidney Disease and Kidney Function.” International Journal of Molecular Sciences 25: 6033. 10.3390/ijms25116033 38892221 PMC11172763

[651] Rinschen, Markus M. , and Julio Saez‐Rodriguez . 2021. “The Tissue Proteome in the Multi‐Omic Landscape of Kidney Disease.” Nature Reviews Nephrology 17: 205–219. 10.1038/s41581-020-00348-5 33028957

[652] Fernandes, Marco , and Holger Husi . 2017. “Establishment of a Integrative Multi‐Omics Expression Database CKDdb in the Context of Chronic Kidney Disease (CKD).” Scientific Reports 7: 40367. 10.1038/srep40367 28079125 PMC5227717

[653] Lake, Blue B. , Rajasree Menon , Seth Winfree , Qiwen Hu , Ricardo Melo Ferreira , Kian Kalhor , Daria Barwinska , et al. 2023. “An Atlas of Healthy and Injured Cell States and Niches in the Human Kidney.” Nature 619: 585–594. 10.1038/s41586-023-05769-3 37468583 PMC10356613

[654] Lake, Blue B. , Ricardo Melo Ferreira , Jens Hansen , Rajasree Menon , Jeannine Basta , Heather Thiessen Philbrook , Stephanie Reinert , et al. 2025. Cellular and Spatial Drivers of Unresolved Injury and Functional Decline in the Human Kidney.” *bioRxiv*. 10.1101/2025.09.26.678707

[655] Wilson, Parker C. , Yoshiharu Muto , Haojia Wu , Anil Karihaloo , Sushrut S. Waikar , and Benjamin D. Humphreys . 2022. “Multimodal Single Cell Sequencing Implicates Chromatin Accessibility and Genetic Background in Diabetic Kidney Disease Progression.” Nature Communications 13: 5253. 10.1038/s41467-022-32972-z PMC9448792 36068241

[656] Lv, Zhimei , Jinxiu Hu , Hong Su , Qun Yu , Yating Lang , Meilin Yang , Xiaoting Fan , et al. 2025. “TRAIL Induces Podocyte PANoptosis Via Death Receptor 5 in Diabetic Kidney Disease.” Kidney International 107: 317–331. 10.1016/j.kint.2024.10.026 39571905

[657] Tuechler, Nadine , Mira Lea Burtscher , Martin Garrido‐Rodriguez , Muzamil Majid Khan , Dénes Türei , Christian Tischer , Sarah Kaspar , et al. 2025. “Dynamic Multi‐Omics and Mechanistic Modeling Approach Uncovers Novel Mechanisms of Kidney Fibrosis Progression.” Molecular Systems Biology 21: 1030–1065. 10.1038/s44320-025-00116-2 40473842 PMC12322177

[658] Abedini, Amin , Jonathan Levinsohn , Konstantin A. Klötzer , Bernhard Dumoulin , Ziyuan Ma , Julia Frederick , Poonam Dhillon , et al. 2024. “Single‐Cell Multi‐Omic and Spatial Profiling of Human Kidneys Implicates the Fibrotic Microenvironment in Kidney Disease Progression.” Nature Genetics 56: 1712–1724. 10.1038/s41588-024-01802-x 39048792 PMC11592391

[659] Li, Haikuo , Dian Li , Nicolas Ledru , Qiao Xuanyuan , Haojia Wu , Amish Asthana , Lori N. Byers , et al. 2024. “Transcriptomic, Epigenomic, and Spatial Metabolomic Cell Profiling Redefines Regional Human Kidney Anatomy.” Cell Metabolism 36: 1105–1125.e10. 10.1016/j.cmet.2024.02.015 38513647 PMC11081846

[660] Jung, Chan‐Young , and Tae‐Hyun Yoo . 2022. “Pathophysiologic Mechanisms and Potential Biomarkers in Diabetic Kidney Disease.” Diabetes & Metabolism Journal 46: 181–197. 10.4093/dmj.2021.0329 35385633 PMC8987689

[661] McDonnell, Thomas , Rosamonde E. Banks , Maarten W. Taal , Nicolas Vuilleumier , and Philip A. Kalra . 2025. “Personalized Care in CKD: Moving Beyond Traditional Biomarkers.” Nephron 149: 339–357. 10.1159/000543640 39848232 PMC12136532

[662] Tofte, Nete , Morten Lindhardt , Katarina Adamova , Stephan J. L. Bakker , Joachim Beige , Joline W. J. Beulens , Andreas L. Birkenfeld , et al. 2020. “Early Detection of Diabetic Kidney Disease by Urinary Proteomics and Subsequent Intervention With Spironolactone to Delay Progression (PRIORITY): A Prospective Observational Study and Embedded Randomised Placebo‐Controlled Trial.” Lancet Diabetes & Endocrinology 8: 301–312. 10.1016/s2213-8587(20)30026-7 32135136

[663] Davies, Elin , Andrew Chetwynd , Garry McDowell , Anirudh Rao , and Louise Oni . 2024. “The Current Use of Proteomics and Metabolomics in Glomerulonephritis: A Systematic Literature Review.” Journal of Nephrology 37: 1209–1225. 10.1007/s40620-024-01923-w 38689160 PMC11405440

[664] Govender, Melanie A. , Jean‐Tristan Brandenburg , June Fabian , and Michèle Ramsay . 2021. “The Use of ‘Omics for Diagnosing and Predicting Progression of Chronic Kidney Disease: A Scoping Review.” Frontiers in Genetics 12: 682929. 10.3389/fgene.2021.682929 34819944 PMC8606569

[665] Barutta, Federica , Stefania Bellini , Silvia Canepa , Marilena Durazzo , and Gabriella Gruden . 2021. “Novel Biomarkers of Diabetic Kidney Disease: Current Status and Potential Clinical Application.” Acta Diabetologica 58: 819–830. 10.1007/s00592-020-01656-9 33528734

[666] McLarnon, Thomas , Steven Watterson , Sean McCallion , Eamonn Cooper , Andrew R. English , Ying Kuan , David S. Gibson , et al. 2025. “Sendotypes Predict Worsening Renal Function in Chronic Kidney Disease Patients.” Clinical and Translational Medicine 15: e70279. 10.1002/ctm2.70279 40147025 PMC11949504

[667] Hill, Claire , Ione Avila‐Palencia , Alexander Peter Maxwell , Ruth F. Hunter , and Amy Jayne McKnight . 2022. “Harnessing the Full Potential of Multi‐Omic Analyses to Advance the Study and Treatment of Chronic Kidney Disease.” Frontiers in Nephrology 2: 923068. 10.3389/fneph.2022.923068 37674991 PMC10479694

[668] Koshino, Akihiko , Hiddo J. L. Heerspink , Niels Jongs , Sunil V. Badve , Clare Arnott , Bruce Neal , Meg Jardine , et al. 2025. “Canagliflozin and Iron Metabolism in the CREDENCE Trial.” Nephrology Dialysis Transplantation 40: 696–706. 10.1093/ndt/gfae198 PMC11960735 39304530

[669] Ingelfinger, Julie R. , and Clifford J. Rosen . 2019. “Clinical Credence—SGLT2 Inhibitors, Diabetes, and Chronic Kidney Disease.” New England Journal of Medicine 380: 2371–2373. 10.1056/NEJMe1904740 30990261

[670] Oshima, Megumi , Brendon L. Neuen , JingWei Li , Vlado Perkovic , David M. Charytan , Dick de Zeeuw , Robert Edwards , et al. 2020. “Early Change in Albuminuria With Canagliflozin Predicts Kidney and Cardiovascular Outcomes: A PostHoc Analysis From the CREDENCE Trial.” Journal of the American Society of Nephrology 31: 2925–2936. 10.1681/asn.2020050723 32998938 PMC7790219

[671] Wheeler, David C. , Bergur V. Stefansson , Mikhail Batiushin , Oleksandr Bilchenko , David Z. I. Cherney , Glenn M. Chertow , Walter Douthat , et al. 2020. “The Dapagliflozin and Prevention of Adverse Outcomes in Chronic Kidney Disease (DAPA‐CKD) Trial: Baseline Characteristics.” Nephrology Dialysis Transplantation 35: 1700–1711. 10.1093/ndt/gfaa234 PMC7538235 32862232

[672] Heerspink, Hiddo J. L. , Niels Jongs , Glenn M. Chertow , Anna Maria Langkilde , John J. V. McMurray , Ricardo Correa‐Rotter , Peter Rossing , et al. 2021. “Effect of Dapagliflozin on the Rate of Decline in Kidney Function in Patients With Chronic Kidney Disease With and Without Type 2 Diabetes: A Prespecified Analysis From the DAPA‐CKD Trial.” Lancet Diabetes & Endocrinology 9: 743–754. 10.1016/s2213-8587(21)00242-4 34619108

[673] Wheeler, David C. , Bergur V. Stefánsson , Niels Jongs , Glenn M. Chertow , Tom Greene , Fan Fan Hou , John J. V. McMurray , et al. 2021. “Effects of Dapagliflozin on Major Adverse Kidney and Cardiovascular Events in Patients With Diabetic and Non‐Diabetic Chronic Kidney Disease: A Prespecified Analysis From the DAPA‐CKD Trial.” Lancet Diabetes & Endocrinology 9: 22–31. 10.1016/s2213-8587(20)30369-7 33338413

[674] Lu, Yong‐Ping , Ze‐Yu Zhang , Hong‐Wei Wu , Li‐Jing Fang , Bo Hu , Chun Tang , Yi‐Qing Zhang , et al. 2022. “SGLT2 Inhibitors Improve Kidney Function and Morphology by Regulating Renal Metabolic Reprogramming in Mice With Diabetic Kidney Disease.” Journal of Translational Medicine 20: 420. 10.1186/s12967-022-03629-8 36104729 PMC9476562

[675] Lundy, David J. , Barbara Szomolay , and Chia‐Te Liao . 2024. “Systems Approaches to Cell Culture‐Derived Extracellular Vesicles for Acute Kidney Injury Therapy: Prospects and Challenges.” Function 5: zqae012. 10.1093/function/zqae012 38706963 PMC11065115

[676] Waijer, Simke W. , Taha Sen , Clare Arnott , Bruce Neal , Jos G. W. Kosterink , Kenneth W. Mahaffey , Chirag R. Parikh , et al. 2022. “Association Between TNF Receptors and KIM‐1 With Kidney Outcomes in Early‐Stage Diabetic Kidney Disease.” Clinical Journal of the American Society of Nephrology 17: 251–259. 10.2215/cjn.08780621 34876454 PMC8823939

[677] Sen, Taha , Jingwei Li , Brendon L. Neuen , Bruce Neal , Clare Arnott , Chirag R. Parikh , Steven G. Coca , et al. 2021. “Effects of the SGLT2 Inhibitor Canagliflozin on Plasma Biomarkers TNFR‐1, TNFR‐2 and KIM‐1 in the CANVAS Trial.” Diabetologia 64: 2147–2158. 10.1007/s00125-021-05512-5 34415356 PMC8423682

[678] Ferguson, Thomas , Pietro Ravani , Manish M. Sood , Alix Clarke , Paul Komenda , Claudio Rigatto , and Navdeep Tangri . 2022. “Development and External Validation of a Machine Learning Model for Progression of CKD.” Kidney International Reports 7: 1772–1781. 10.1016/j.ekir.2022.05.004 35967110 PMC9366291

[679] Shen, Mei‐di , Si‐bing Chen , and Xiang‐dong Ding . 2024. “The Effectiveness of Digital Twins in Promoting Precision Health Across the Entire Population: A Systematic Review.” npj Digital Medicine 7: 145. 10.1038/s41746-024-01146-0 38831093 PMC11148028

[680] Koshland, Daniel E . 1995. “The Key–Lock Theory and the Induced Fit Theory.” Angewandte Chemie International Edition in English 33: 2375–2378. 10.1002/anie.199423751

[681] Akhoon, Neha . 2021. “Precision Medicine: A New Paradigm in Therapeutics.” International Journal of Preventive Medicine 12: 12. 10.4103/ijpvm.IJPVM_375_19 34084309 PMC8106271

[682] Godlee, F. 2011. “Who Should Define Disease?” BMJ 342: d2974. 10.1136/bmj.d2974

[683] Ereshefsky, Marc . 2009. “Defining ‘Health’ and ‘Disease’.” Studies in History and Philosophy of Science Part C: Studies in History and Philosophy of Biological and Biomedical Sciences 40: 221–227. 10.1016/j.shpsc.2009.06.005 19720330

[684] Bircher, Johannes . 2005. “Towards a Dynamic Definition of Health and Disease.” Medicine, Health Care and Philosophy 8: 335–341. 10.1007/s11019-005-0538-y 16283496

[685] Cooper, Rachel . 2002. “Disease.” Studies in History and Philosophy of Science Part C: Studies in History and Philosophy of Biological and Biomedical Sciences 33: 263–282. 10.1016/S0039-3681(02)00018-3

[686] Bauer, Ursula E. , Peter A. Briss , Richard A. Goodman , and Barbara A. Bowman . 2014. “Prevention of Chronic Disease in the 21st Century: Elimination of the Leading Preventable Causes of Premature Death and Disability in the USA.” Lancet 384: 45–52. 10.1016/S0140-6736(14)60648-6 24996589

[687] Wild, S. H. , and C. D. Byrne . 2014. “Commentary: Sub‐Types of Diabetes—What's New and What's Not.” International Journal of Epidemiology 42: 1600–1602. 10.1093/ije/dyt201 24415600

[688] Misra, Shivani , Robert Wagner , Bige Ozkan , Martin Schön , Magdalena Sevilla‐Gonzalez , Katsiaryna Prystupa , Caroline C. Wang , et al. 2023. “Precision Subclassification of Type 2 Diabetes: A Systematic Review.” Communications Medicine 3: 138. 10.1038/s43856-023-00360-3 37798471 PMC10556101

[689] Sugandh, F. N. U. , Maria Chandio , F. N. U. Raveena , Lakshya Kumar , F. N. U. Karishma , Sundal Khuwaja , Unaib Ahmed Memon , et al. 2023. “Advances in the Management of Diabetes Mellitus: A Focus on Personalized Medicine.” Cureus 15: e43697. 10.7759/cureus.43697 37724233 PMC10505357

[690] Holford, Nicholas H. G . 1986. “Clinical Pharmacokinetics and Pharmacodynamics of Warfarin.” Clinical Pharmacokinetics 11: 483–504. 10.2165/00003088-198611060-00005 3542339

[691] Kuruvilla, Mariamma , and Cheryle Gurk‐Turner . 2001. “A Review of Warfarin Dosing and Monitoring.” Baylor University Medical Center Proceedings 14: 305–306. 10.1080/08998280.2001.11927781 16369639 PMC1305837

[692] Fung, Erik , Nikolaos Patsopoulos , Steven Belknap , Daniel O'Rourke , John Robb , Jeffrey Anderson , Nicholas Shworak , and Jason Moore . 2012. “Effect of Genetic Variants, Especially CYP2C9 and VKORC1, on the Pharmacology of Warfarin.” Seminars in Thrombosis and Hemostasis 38: 893–904. 10.1055/s-0032-1328891 23041981 PMC4134937

[693] Lefferts, Joel A. , Mary C. Schwab , Uday B. Dandamudi , Hong‐Kee Lee , Lionel D. Lewis , and Gregory J. Tsongalis . 2010. “Warfarin Genotyping Using Three Different Platforms.” American Journal of Translational Research 2: 441–446. https://pmc.ncbi.nlm.nih.gov/articles/PMC2923866/ 20733952 PMC2923866

[694] Jin, Lv , Xiao‐Yu Zuo , Wei‐Yang Su , Xiao‐Lei Zhao , Man‐Qiong Yuan , Li‐Zhen Han , Xiang Zhao , Ye‐Da Chen , and Shao‐Qi Rao . 2014. “Pathway‐Based Analysis Tools for Complex Diseases: A Review.” Genomics, Proteomics & Bioinformatics 12: 210–220. 10.1016/j.gpb.2014.10.002 PMC4411419 25462153

[695] Kenakin, T . 2002. “The Ligand Paradox Between Affinity and Efficacy: Can You be There and Not Make a Difference?” Trends in Pharmacological Sciences 23: 275–280. 10.1016/S0165-6147(02)02036-9 12084633

[696] Giordano, S. , and A. Petrelli . 2008. “From Single‐ to Multi‐Target Drugs in Cancer Therapy: When Aspecificity Becomes an Advantage.” Current Medicinal Chemistry 15: 422–432. 10.2174/092986708783503212 18288997

[697] Sadri, Arash . 2023. “Is Target‐Based Drug Discovery Efficient? Discovery and ‘Off‐Target’ Mechanisms of All Drugs.” Journal of Medicinal Chemistry 66: 12651–12677. 10.1021/acs.jmedchem.2c01737 37672650

[698] L. Bolognesi, M . 2013. “Polypharmacology in a Single Drug: Multitarget Drugs.” Current Medicinal Chemistry 20: 1639–1645. 10.2174/0929867311320130004 23410164

[699] Makhoba, Xolani H. , Claudio Viegas Jr. , Rebamang A. Mosa , Flávia PD Viegas , and Ofentse J. Pooe . 2020. “Potential Impact of the Multi‐Target Drug Approach in the Treatment of Some Complex Diseases.” Drug Design, Development and Therapy 14: 3235–3249. 10.2147/DDDT.S257494 32884235 PMC7440888

[700] Milowsky, Matthew I. , Gopa Iyer , Ashley M. Regazzi , Hikmat Al‐Ahmadie , Scott R. Gerst , Irina Ostrovnaya , Lan L. Gellert , et al. 2013. “Phase II Study of Everolimus in Metastatic Urothelial Cancer.” BJU International 112: 462–470. 10.1111/j.1464-410X.2012.11720.x 23551593 PMC4020005

[701] Iyer, Gopa , Aphrothiti J. Hanrahan , Matthew I. Milowsky , Hikmat Al‐Ahmadie , Sasinya N. Scott , Manickam Janakiraman , Mono Pirun , et al. 2012. “Genome Sequencing Identifies a Basis for Everolimus Sensitivity.” Science 338: 221. 10.1126/science.1226344 22923433 PMC3633467

[702] Saad, Everardo D. , Xavier Paoletti , Tomasz Burzykowski , and Marc Buyse . 2017. “Precision Medicine Needs Randomized Clinical Trials.” Nature Reviews Clinical Oncology 14: 317–323. 10.1038/nrclinonc.2017.8 28169302

[703] Fountzilas, Elena , Apostolia M. Tsimberidou , Henry Hiep Vo , and Razelle Kurzrock . 2022. “Clinical Trial Design in the Era of Precision Medicine.” Genome Medicine 14: 101. 10.1186/s13073-022-01102-1 36045401 PMC9428375

[704] Thakkar, Simran , Aditi Chopra , Lakshmi Nagendra , Sanjay Kalra , and Saptarshi Bhattacharya . 2023. “Teplizumab in Type 1 Diabetes Mellitus: An Updated Review.” touchREVIEWS in Endocrinology 19: 7. 10.17925/ee.2023.19.2.7 PMC10769466 38187075

[705] Mathieu, Chantal , Emily K. Sims , Lucienne Chatenoud , Eddie A. James , Mark A. Atkinson , and Kevan C. Herold . 2025. “Toward Disease‐Modifying Therapies in Type 1 Diabetes: Focus on Teplizumab.” Diabetes Care 49: 365–374. 10.2337/dci25-0066 PMC12925991 41196630

[706] Baião, Ana R. , Zhaoxiang Cai , Rebecca C. Poulos , Phillip J. Robinson , Roger R. Reddel , Qing Zhong , Susana Vinga , and Emanuel Gonçalves . 2025. “A Technical Review of Multi‐Omics Data Integration Methods: From Classical Statistical to Deep Generative Approaches.” Briefings in Bioinformatics 26: bbaf355. 10.1093/bib/bbaf355 40748323 PMC12315550

[707] Si, Tong , and Haijun Gong . 2026. “Challenges and Advances in Bioinformatics and Computational Biology.” Current Issues in Molecular Biology 48: 185. 10.3390/cimb48020185 41751447 PMC12939951

[708] Cardoso, Fatima , Laura J. van't Veer , Jan Bogaerts , Leen Slaets , Giuseppe Viale , Suzette Delaloge , Jean‐Yves Pierga , et al. 2016. “70‐Gene Signature as an Aid to Treatment Decisions in Early‐Stage Breast Cancer.” New England Journal of Medicine 375: 717–729. 10.1056/NEJMoa1602253 27557300

[709] Robinson, Peter N . 2012. “Deep Phenotyping for Precision Medicine.” Human Mutation 33: 777–780. 10.1002/humu.22080 22504886

[710] Wei, Wei‐Qi , and Joshua C. Denny . 2015. “Extracting Research‐Quality Phenotypes From Electronic Health Records to Support Precision Medicine.” Genome Medicine 7: 41. 10.1186/s13073-015-0166-y 25937834 PMC4416392

[711] Brock, Amy , and Sui Huang . 2017. “Precision Oncology: Between Vaguely Right and Precisely Wrong.” Cancer Research 77: 6473–6479. 10.1158/0008-5472.Can-17-0448 29162615

[712] Sisodiya, Sanjay M . 2021. “Precision Medicine and Therapies of the Future.” Epilepsia 62: S90–S105. 10.1111/epi.16539 32776321 PMC8432144

[713] Padakanti, Anudeep , Vidya Sagar Dusi , Suresh V. S. Attili , Palanki Satya Dattatreya , and Rakesh Sharma . 2025. “Feasibility of Half‐Dose Dual‐Targeted Therapy in Advanced Solid Tumors With NGS‐proven Actionable Targets: A Comparative Analysis With OMCT.” Journal of Clinical Oncology 43: e14659. 10.1200/JCO.2025.43.16_suppl.e14659

[714] Schreiber, Stefan , Konrad Aden , Florian Tran , and Philip Rosenstiel . 2026. “Rise of Precision Medicine: Can It Deliver on Its Promise in IBD?” Gut 75: 176–188. 10.1136/gutjnl-2023-330000 PMC12703253 41052915

[715] Xu, Da , Shuang Chen , Jun Yang , Xiufeng Wang , Zhi Fang , and Man Li . 2022. “Precision Therapy With Quinidine of KCNT1‐Related Epileptic Disorders: A Systematic Review.” British Journal of Clinical Pharmacology 88: 5096–5112. 10.1111/bcp.15479 35940594

[716] Zographos, George A. , Sophie J. Russ‐Hall , and Ingrid E. Scheffer . 2022. “Does Long‐Term Phenytoin Have a Place in Dravet Syndrome?” Annals of Clinical and Translational Neurology 9: 2036–2040. 10.1002/acn3.51684 36314457 PMC9735367

[717] Lin, Shujin , Dan Feng , Xiao Han , Ling Li , Yao Lin , and Haibing Gao . 2024. “Microfluidic Platform for Omics Analysis on Single Cells With Diverse Morphology and Size: A Review.” Analytica Chimica Acta 1294: 342217. 10.1016/j.aca.2024.342217 38336406

[718] Aldea, M. , L. Friboulet , S. Apcher , F. Jaulin , F. Mosele , T. Sourisseau , J.‐C. Soria , S. Nikolaev , and F. André . 2023. “Precision Medicine in the Era of Multi‐Omics: Can the Data Tsunami Guide Rational Treatment Decision?” ESMO Open 8: 101642. 10.1016/j.esmoop.2023.101642 37769400 PMC10539962

[719] Horng, Hannah , Apurva Singh , Bardia Yousefi , Eric A. Cohen , Babak Haghighi , Sharyn Katz , Peter B. Noël , Russell T. Shinohara , and Despina Kontos . 2022. “Generalized ComBat Harmonization Methods for Radiomic Features With Multi‐Modal Distributions and Multiple Batch Effects.” Scientific Reports 12: 4493. 10.1038/s41598-022-08412-9 35296726 PMC8927332

[720] Petersen, Lauren M. , Isabella W. Martin , Wayne E. Moschetti , Colleen M. Kershaw , and Gregory J. Tsongalis . 2019. “Third‐Generation Sequencing in the Clinical Laboratory: Exploring the Advantages and Challenges of Nanopore Sequencing.” Journal of Clinical Microbiology 58: e01315–e01319. 10.1128/jcm.01315-19 31619531 PMC6935936

[721] Burger, Thomas . 2026. “Keeping Generative Artificial Intelligence Reliable in Omics Biology.” Patterns 7: 101417. 10.1016/j.patter.2025.101417 41583973 PMC12827736

[722] Denis, Lucas , Anna Kirstine Jørgensen , Bernard Do , Inès Vaz‐Luis , Barbara Pistilli , André Rieutord , Abdul W. Basit , Alvaro Goyanes , and Maxime Annereau . 2024. “Developing an Innovative 3D Printing Platform for Production of Personalised Medicines in a Hospital for the OPERA Clinical Trial.” International Journal of Pharmaceutics 661: 124306. 10.1016/j.ijpharm.2024.124306 38871137

[723] Rioblanc, François , Baptiste Heumel , Daniel Alloh Nammou , Julie Roupret‐Serzec , Patrick Saulnier , and Thomas Storme . 2025. “Opensource Semi‐Solid 3D Printer Designed for Oral Drug Production in Hospital Pharmacies.” International Journal of Pharmaceutics 682: 125998. 10.1016/j.ijpharm.2025.125998 40714168

[724] Boakye Serebour, Tracy , Adam P. Cribbs , Mathew J. Baldwin , Collen Masimirembwa , Zedias Chikwambi , Angeliki Kerasidou , and Sarah J. B. Snelling . 2024. “Overcoming Barriers to Single‐Cell RNA Sequencing Adoption in Low‐ and Middle‐Income Countries.” European Journal of Human Genetics 32: 1206–1213. 10.1038/s41431-024-01564-4 38565638 PMC11499908

[725] Mehra, Tarun , Dominik Menges , Benedict Gosztonyi , Nicola Miglino , Alexander Ring , Laura Boos , Bettina Sobottka , et al. 2025. “Comparative Cost Analysis of a Diagnostic Multi‐Omics Platform for Decision Support in Advanced Cancer—Results From the Tumor Profiler Melanoma Project.” npj Precision Oncology 10: 35. 10.1038/s41698-025-01229-5 41402424 PMC12819374

[726] Pataky, Reka E. , Deirdre Weymann , Ian Bosdet , Stephen Yip , Stirling Bryan , Mohsen Sadatsafavi , Stuart Peacock , and Dean A. Regier . 2024. “Real‐World Cost‐Effectiveness of Panel‐Based Genomic Testing to Inform Therapeutic Decisions for Metastatic Colorectal Cancer.” Journal of Cancer Policy 41: 100496. 10.1016/j.jcpo.2024.100496 39032558

[727] Nettersheim, Felix Sebastian , Sujit Silas Armstrong , Christopher Durant , Rafael Blanco‐Dominguez , Payel Roy , Marco Orecchioni , Vasantika Suryawanshi , and Klaus Ley . 2022. “Titration of 124 Antibodies Using CITE‐Seq on Human PBMCs.” Scientific Reports 12: 20817. 10.1038/s41598-022-24371-7 36460735 PMC9718773

[728] LeMieux, Julianna . 2025. “The Single‐Cell Club Is Rapidly Expanding:As Single‐Cell RNA‐Seq Meets AI, and DNA Sequencing Prices Drop, Projects Are Becoming Much (Much) Larger.” Genetic Engineering & Biotechnology News 45: 28–31. 10.1089/gen.45.07.11

[729] Chiu, Charles Y. , and Steven A. Miller . 2019. “Clinical Metagenomics.” Nature Reviews Genetics 20: 341–355. 10.1038/s41576-019-0113-7 PMC6858796 30918369

[730] Li, Bing , Yuanjun Tang , Zhanya Huang , Lijun Ma , Jiagui Song , and Lixiang Xue . 2025. “Synergistic Innovation in Organ‐On‐A‐Chip and Organoid Technologies: Reshaping the Future of Disease Modeling, Drug Development, and Precision Medicine.” Protein & Cell 17: 686–703. 10.1093/procel/pwaf058 PMC13429468 40653861

[731] Sadhuka, Shuvom , Daniel Fridman , Bonnie Berger , and Hyunghoon Cho . 2023. “Assessing Transcriptomic Reidentification Risks Using Discriminative Sequence Models.” Genome Research 33: 1101–1112. 10.1101/gr.277699.123 37541758 PMC10538488

[732] Fierro‐Monti, Ivo , James C. Wright , Jyoti S. Choudhary , and Juan Antonio Vizcaíno . 2022. “Identifying Individuals Using Proteomics: Are We There Yet?” Frontiers in Molecular Biosciences 9: 1062031. 10.3389/fmolb.2022.1062031 36523653 PMC9744771

[733] Klitzman, Robert , Paul S. Appelbaum , and Wendy K. Chung . 2014. “Should Life Insurers Have Access to Genetic Test Results?” JAMA 312: 1855–1856. 10.1001/jama.2014.13301 25387181 PMC4259574

[734] Zhang, Jingcheng , Yekai Zhou , Yingxuan Ren , Man Ho Au , Ka‐Ho Chow , Lei Chen , Yanmin Zhao , Junhao Su , and Ruibang Luo . 2025. “Toward Owner Governance in Genomic Data Privacy With Governome.” Cell Reports Methods 5: 101171. 10.1016/j.crmeth.2025.101171 40930087 PMC12539250

[735] Wang, Ran , Fangwen Ye , Sisui Tang , Hangning Zhang , Jie He , Xiaotong Zhang , and Cheng Xu . 2025. “Blockchain Technology for Big‐Data Sharing in Material Genome Engineering.” Scientific Data 12: 1813. 10.1038/s41597-025-06092-4 41253894 PMC12627100

[736] Adanur Dedeturk, Beyhan , Ahmet Soran , and Burcu Bakir‐Gungor . 2026. “GenShare: A Blockchain‐Based Genomic Data Sharing Platform.” Distributed Ledger Technologies: Research and Practice 5: 1–21. 10.1145/3743696

[737] Hasner, Mira C. , Mark P. van Opijnen , Filip Y. F. de Vos , Edwin Cuppen , and Marike L. D. Broekman . 2024. “Whole Genome Sequencing in (Recurrent) Glioblastoma: Challenges Related to Informed Consent Procedures and Data Sharing.” Acta Neurochirurgica 166: 266. 10.1007/s00701-024-06158-z 38874628 PMC11178618

[738] Jonsson, Jon J. , and Vigdis Stefansdottir . 2019. “Ethical Issues in Precision Medicine.” Annals of Clinical Biochemistry: International Journal of Laboratory Medicine 56: 628–629. 10.1177/0004563219870824 31370674

